# Identifying diabetes-related important protein targets with few interacting partners with the PageRank algorithm

**DOI:** 10.1098/rsos.140252

**Published:** 2015-04-29

**Authors:** Vince I. Grolmusz

**Affiliations:** PIT Bioinformatics Group, Eötvös University, Pázmány Péter stny 1/c, Budapest, Hungary

**Keywords:** protein interaction database, interactome, PageRank, personalized PageRank, relativized PageRank, target identification

## Abstract

Diabetes is a growing concern for the developed nations worldwide. New genomic, metagenomic and gene-technologic approaches may yield considerable results in the next several years in its early diagnosis, or in advances in therapy and management. In this work, we highlight some human proteins that may serve as new targets in the early diagnosis and therapy. With the help of a very successful mathematical tool for network analysis that formed the basis of the early successes of Google^TM^, Inc., we analyse the human protein–protein interaction network gained from the IntAct database with a mathematical algorithm. The novelty of our approach is that the new protein targets suggested do not have many interacting partners (so, they are not hubs or super-hubs), so their inhibition or promotion probably will not have serious side effects. We have identified numerous possible protein targets for diabetes therapy and/or management; some of these have been well known for a long time (these validate our method), some of them appeared in the literature in the last 12 months (these show the cutting edge of the algorithm), and the remainder are still unknown to be connected with diabetes, witnessing completely new hits of the method.

## 2. Introduction

Every day dozens of scientific publications appear in the world in each important area of biology and medicine [1]. Lots of relevant results get published in every field, and most of them go unnoticed by the majority of researchers even if they are working on the very same area. Electronically maintained databases of the literature help scientists to follow the new developments of each area.

Knowledge related to the structure, function and interactions of proteins are represented, organized, cleaned, filtered, converted and published regularly in numerous databases. These plentiful information sources could transform health sciences in the coming years if appropriate tools for automatic processing of the databases become widely available.

One goal of scientists specializing in human diseases is to find new possible protein targets of intervention for a given disease. It is well known that the human genome contains around 21 000 genes that encode more than 100 000 proteins [2]. From this set of proteins, one source [3] lists 364 protein and nucleic acid targets for the 1540 approved drugs, while another paper [4] mentions only 133 targets for FDA approved drugs. Thus, very few proteins are targeted by the drugs approved today, and clearly the number of targeted proteins needs to be increased dramatically in the coming years.

For this goal, concentrated efforts are needed: from hundreds or thousands of possible targets, smaller, more focused sets need to be chosen for these concentrated efforts.

In this paper, we describe a new method that is based on the PageRank computation of the Google web-search engine, which is capable of identifying new, relevant protein targets for possible effects on diabetes. These proteins may serve as the targets of more focused efforts in the future.

### 2.1 The protein interaction graph and PageRank

The overall protein concentration of the cell is very high; about 20–30% of the cytosol [5] consists of proteins, and it is proven that a great proportion of proteins work in interaction with other proteins in the cell. The usual way of describing these interactions is the interaction graph: the vertices of the graph are the proteins, and two vertices are connected by an edge if they would interact. In the case of directed interactions (e.g. in metabolic processes or signalling networks), the graph edges can also be directed.

It is a natural idea to assign importance to those vertices that are connected to many other vertices; these vertices are called ‘hubs’. It has turned out, however, that this choice—in numerous aspects—is a too simplistic solution; it is not robust against errors in the data. From the alternative definitions of importance that appeared in 1998 [6,7], the PageRank algorithm [7] proved to be more applicable, robust [8], and useful; the search engine of Google also used this method for finding the most important hits in a web search (a short review of the definition of PageRank is given in §5.5). In biological networks, we can especially make use of the robustness of PageRank [8], as protein interaction data may contain numerous false positives and false negatives, and if small changes in the data would imply large changes in PageRank, then the whole concept of PageRank computation would be useless. We remark that the—otherwise very appealing—method of Kleinberg [6] is not robust [9]; this was one of the reasons for the success of the PageRank algorithm.

We have proposed previously to use PageRank for the analysis of protein interaction networks [10]. In that work, the PageRank algorithm was applied to finding relevant nodes in directed graphs, corresponding to metabolic networks of different organisms and also for undirected graphs with personalization.

It is well known that high-degree nodes in a network (sometimes called hubs, hub-nodes, commodity-nodes) have large PageRanks [11,12]; and, on the other hand, large-degree nodes are usually of vital importance in numerous biochemical processes [13]. Inhibiting or promoting the activity or the production of these proteins with a large number of interactions may have numerous unwanted side-effects, as their modifications (inhibition or promotion) would influence many other processes and proteins in the cell. Therefore, these large-degree nodes usually are not viable candidates for protein targets, so we intended to discover low-degree nodes in the network having importance in the molecular mechanisms of diabetes.

In Bánky *et al.* [12], based on Grolmusz [14], we introduced a measure of importance for the nodes of directed graphs that are essentially computed by dividing the PageRank of the node by its degree (see §5.5 for details). Therefore, the small-degree nodes are compensated, and one can find small-degree and relevant nodes, as well.

We apply a somewhat similar method in this work: we divided the personalized PageRanks of the nodes of the human interactome (an undirected graph) by their degrees; and reviewed the vertices with the highest PageRank/degree values.

## 3. Results

Our results are represented in tables 1 and 2 and the electronic supplementary material, table S3.

**Table 1.** The list of the five nodes with the highest personalized PageRank. The first four vertices are of enormous degrees, each of them are interacting with hundreds of other proteins. The fifth protein has a high PageRank as it was the element of the set of diabetes-related proteins that we personalized to. This table shows that the highest personalized PageRank nodes would not be very interesting for finding new target proteins for diabetes research.

PageRank	NodeID	degree	name
1846.97	P01106	732	Myc proto-oncogene protein: MYC
1752.83	P62993	678	growth factor receptor-bound protein 2: GRB2
1439.11	P19320	629	vascular cell adhesion protein 1: VCAM1
1260.13	P08238	491	heat shock protein HSP 90-beta: HSP90AB1
1251.87	Q96RG2	32	PAS-kinase: PASK; personalized

**Table 2.** The list of the 19 nodes with the highest PageRank/degree values; we considered here only those vertices that we have not personalized to. The remarks for each row are given in the Discussion section.

NodeID	PageRank	degree	PR/Deg	name
P47211	397.29	1	397.29	galanin receptor type 1
O43603	397.29	1	397.29	galanin receptor type 2
O75325	302.59	1	302.59	leucine-rich repeat neuronal protein 2
P37288	518.14	2	259.07	vasopressin V1a receptor
Q8IWW8	487.05	2	243.53	alcohol dehydrogenase iron-containing protein 1
Q9BZL3	184.67	1	184.67	small integral membrane protein 3
P00736	550.25	3	183.42	complement C1r subcomponent
P18505	319.38	2	159.69	GABA(A) receptor subunit beta-1
P09871	147.34	1	147.34	complement C1s subcomponent, C1 esterase
P16118	344.56	3	114.85	6-phosphofructo-2-kinase
P55317	393.93	4	98.48	hepatocyte nuclear factor 3-alpha
P62341	95.12	1	95.12	selenoprotein T
Q9NZ43	188.62	2	94.31	vesicle transport protein USE1
Q96HH6	188.62	2	94.31	transmembrane protein 19
Q9Y2Y9	239.08	3	79.69	Krueppel-like factor 13 NSLP1 BTEB3
P43694	65.78	1	65.78	transcription factor GATA-4
Q9UGH3	64.64	1	64.64	sodium-dependent vitamin C transporter 2
P40199	315.28	5	63.06	carcinoembryonic antigen-rel. cell adh. mol. 6
P14778	375.55	6	62.59	interleukin-1 receptor type 1

Table 1 shows that even the personalized PageRank would identify very large-degree nodes (hubs) with the highest scores that have too many interacting partners and, therefore, are virtually useless for possible interventions.

Table 2 lists the best hits found in this work, and we review them one-by-one in the next section. The whole set of results is given in the electronic supplementary material, table S3.

## 4. Discussion

In this section, we review the protein hits with the highest score given in table 2. We will find that some of them have well-known connections with diabetes, so these proteins should have been included in the list of diabetes-related proteins constructed from the UniProt database (electronic supplementary material, table S2); the others are clearly interesting hits with at most one or two references that connect them to diabetes, or without such connections. The full list of hits is given in electronic supplementary material, table S3.

P47211 Galanin receptor type 1: connection with diabetes is well known (e.g. [15–17]).
O43603 Galanin receptor type 2: connection with diabetes is well known.
O75325 Leucine-rich repeat neuronal protein 2: we have not found any direct connections with diabetes; however, it is over-amplified in malignant gliomas as shown by the UniProt source. Malignant gliomas and diabetes have numerous references (e.g. [18]).
P37288 Vasopressin V1a receptor: connection with diabetes is well known.
Q8IWW8 Alcohol dehydrogenase iron-containing protein 1: we have found only one, but a very interesting reference [19]. The authors selected thoroughbred horses with exceptional racing performance, and performed genetic mapping; they found that the gene *ADHFE1* of protein Q8IWW8 might be involved in increased insulin sensitivity of the horses.
Q9BZL3 Small integral membrane protein 3; we have not found any data concerning the link with diabetes.
P00736 Complement C1r subcomponent, C1R, a serine protease: may be involved in metabolic changes in pregnancy [20].
P18505 GABA(A) receptor subunit beta-1: very recently, one study [21] have found the gene for this protein to be related to a higher incidence of type-2 diabetes in an extended UAE Arab family.
P09871 C1 esterase; its connection with diabetes is well known (e.g. [22]).
P16118 6-Phosphofructo-2-kinase, PFKFB1; Atsumi *et al*. [23] show it may be related to obesity, and Garcia-Herrero *et al*. [24] show it interacts with glucokinase (GCK) that, in turn, acts as a glucose sensor in the pancreatic beta cell and regulates insulin secretion.
P55317 Hepatocyte nuclear factor 3-alpha, FOXA1: Garcia-Herrero *et al*. [24] show the deficiency of transcription factor Neurogenin3 leads to diabetes in humans; and FOXA1 can amplify the auto-regulation of Neurogenin3.
P62341 Selenoprotein T, SELT: we have not found any data concerning the link with diabetes.
Q9NZ43 Vesicle transport protein, USE1: we have not found any data concerning the link with diabetes.
Q96HH6 Transmembrane protein 19: we have not found any data concerning the link with diabetes.
Q9Y2Y9 Krueppel-like factor 13, NSLP1, BTEB3: we have not found any data concerning the link with diabetes.
P43694 Transcription factor GATA-4: it is mostly mentioned in relation to neonatal heart disease, but very recently its connections with early pancreas development have been described [25]. It may have a role in regenerative therapy in type-1 diabetes.
Q9UGH3 Sodium-dependent vitamin C transporter 2, SVCT2: numerous sources show the pivotal role of this protein in diabetes, e.g. [26].
P40199 Carcinoembryonic antigen-related cell adhesion molecule 6, CD66c: we have not found any data concerning the link with diabetes.
P14778 Interleukin-1 receptor type 1, IL1R1: its connection with diabetes is well known (e.g. [27]).

## 5. Material and methods

The methodology of this work comprises the following parts:

— downloading, filtering and pre-processing the human interactions from the IntAct database [28];
— downloading and pre-processing the set of diabetes-related proteins from the UniProt database [29];
— computing the PageRank for the nodes in the human protein interaction graph, personalized to the diabetes-related vertices [10], by the Perl-script at http://uratim.com/pp.zip; and
— post-processing and evaluating the results.

### 5.1 Constructing the human interaction graph

Some tools (e.g. [30,31]) and databases [32] on the World Wide Web produce or contain predicted interactions. Other databases contain only protein interactions inferred from laboratory experiments (e.g. MINT [33], HPRD [34], DIP [35] and IntAct [28]). We have chosen for our present study the rich, laboratory-data based, constantly updated IntAct protein interaction database of the European Bioinformatics Institute.

The binary interactions from the IntAct database were downloaded with the ‘Homo sapiens’; organism filter in MI-TAB 2.5 format [28] on 13 October 2013.

The downloaded data still contained proteins from non-human species in some interactions; these interactions were deleted. We also removed those interactions where at least one of the interacting proteins was not denoted by their UniProt accession number [29]. Next, the isoforms of proteins (e.g. P02545-2) in the table were substituted by their clear UniProt accession numbers (e.g. P02545).

The resulting table contained numerous multiplicities, that is multiple appearances of the same interaction.

We intended to build a graph edge-list from the list of interactions. Physical protein–protein interactions are symmetric relations, in other words the graph that corresponds to the dataset is undirected. Our script that computes the personalized PageRank needs the graph-edges to be given as directed edges, therefore we followed the process below:

— For each pair (*a*,*b*), describing the interaction between proteins identified by their UniProt accession numbers *a* and *b*, we added also the pair (*b*,*a*), even if *a*=*b* was true.
— Then we removed all multiplicities, that is, if (*a*,*b*) was in the list considered above, and *a*≠*b*, then the final table contains (*a*,*b*) and (*b*,*a*) (each once), and if *a*=*b* then it contains (*a*,*a*) only once.

We worked in the resulting undirected graph, with edge-list corresponding to the table constructed: each non-directed edge corresponds to a symmetric pair of directed edges. The edge-list is given in the electronic supplementary material, table S1, with 120 175 directed edges on 11 766 vertices.

### 5.2 The initial list of proteins with a role in diabetes

The UniProt database [29] contains extensive annotations of the proteins deposited. These annotations are based on literature evidence and are very useful tools for fast and reliable retrieval of protein subsets related to some disease or syndrome.

We performed the following search of the UniProt database (on 13 October 2013): ‘(diabetes AND organism: ‘Homo sapiens [9606]’) AND reviewed:yes’, meaning that we were looking for proteins, related to diabetes (both type-1 and type-2); the proteins needed to be human ones, and needed to be the elements of the SwissProt (i.e. the manually reviewed) subset of UniProt. We remark that SwissProt is the strongly controlled subset of UniProt, containing the sequences of proteins that have evidence of their existence at the protein level (i.e. they are not just predicted from their gene sequences). Using this latter restriction is important to assure the quality of the data used in this work.

The query above returned 195 proteins; their annotated list is given in the electronic supplementary material, table S2.

### 5.3 Performing the personalized PageRank computation

The Perl script, downloadable from http://uratim.com/pp.zip [10], was applied to the human protein interaction graph taken from the IntAct database (electronic supplementary material, table S1), with personalization to the proteins marked as ‘related to diabetes’ in UniProt (electronic supplementary material, table S2). The Perl script scales the PageRank values by multiplying all numbers such that the smallest PageRank is set to be 1 (without scaling the numbers would be uncomfortably small: their sum for the 11 766 vertices would have been 1). The parameters of the computation were the following ones in the Perl script: damping_factor=0; personalization_damping_factor=0.15. The PageRank computation is very fast, taking less than 10 s even on a low-end laptop computer.

### 5.4 Post-processing and evaluating the results

From the nature of the PageRank computation with personalization, the nodes that we personalized to have high scores in this measure; so we were looking for proteins that

— were not the elements of the diabetes-related vertex-set from UniProt that we personalized to (electronic supplementary material, table S2); and
— have high value of the PageRank/degree quotient.

The first requirement assures us that we identify either new proteins that are important in diabetes or at least proteins that were not listed as ‘diabetes-related’ in the UniProt database and our electronic supplementary material, table S2; the second requirement assures us that we identify important proteins possibly with only few interacting partners.

The nodes with the highest PageRank are given in table 1, the nodes with the highest PageRank/degree quotient are given in table 2 and the list of all hits are given in the electronic supplementary material, table S3.

### 5.5 Mathematical remarks

In this section, we review some of the mathematical details of the new method of finding important nodes in undirected graphs for those readers with mathematical interest.

The PageRank algorithm [7] can be described by a random walk on the graph. First let us review the simplest case of a random walk: we are given a non-bipartite, connected, undirected graph, *G*, and a player is wandering on the graph according to the rules:

(a) if the player is at a node *v* with *d* connected edges, then she will choose randomly each adjacent edge with the same 1/*d* probability for the next move.

One can show very easily [36] that, after a long time, the probability of being at a given node will be independent of the identity of the starting node and also of the number of steps taken, and will be proportional to the degree of that given node.

For directed graphs, no such statement is true. Page & Brin [7], in the process of designing the Google search engine, suggested a little different random walk on the graph, that would also work well for directed graphs:

— The player at each step will choose with 80% probability step *A* and with 20% probability step *B*:
  (*A*) if the player is at a node *v* with *d* out-going edges, then she will choose randomly each outgoing edge with the same 1/*d* probability for the next move; and
  (*B*) the player teleports to any vertex of the graph, with the same probability (i.e. if the graph has *n* vertices, then the player jumps to any vertex with the same 1/*n* probability).

The probability of being at vertex *v* after a large number of steps is the PageRank of vertex *v* [7].

In the early versions of the Google search engine, PageRank was used for assigning a measure of importance to web pages, and the web pages of higher relevance were shown to the user first, and the less important ones later on in the hit-list of a web search [7].

The personalized PageRank [37] differs from the original PageRank in the teleporting step *B*: let vertex set *V* ′⊂*V* , and let the size of set *V* ′ be equal to *m*≤*n*, then step *B*′ needs to be substituted for *B*:

(*B*′)the player teleports to any vertex of the set *V* ′⊂*V* , with the same probability 1/*m*.

The probability of being at vertex *v* after a large number of steps is the personalized PageRank of vertex *v* [37].

Originally [37], this definition described the importance of web pages related to some personal interest of the user, represented by the set of subjectively interesting web pages *V* ′⊂*V* ; because of this historical reason it is called ‘personalized’ PageRank.

Certainly, in the process, the vertices we have personalized to have high PageRank; we, of course, should not consider those vertices in the search for new, relevant ones.

In Ivan & Grolmusz [10], we have applied the personalized PageRank to a biomedical problem in the following way: we considered the human undirected protein interaction graph, and personalized the PageRank to a proteomics dataset for human melanoma. We received high PageRank vertices, relative to this personalization, of both known and unknown functions; it is very appealing that most of the proteins of the high PageRank vertices with known functions were related to cancers; therefore, we may assume that the high PageRank vertices with unknown functions are also related to cancers in general and melanoma in particular. Since low-concentration proteins cannot be identified in proteomics experiments, this method may also be capable of finding those hidden proteins in a disease.

In [12], we introduced a method for directed graphs that is capable of identifying important nodes of low degree. The method has the following background.

— Let us recall that the limit distribution of a random walk on a connected, non-bipartite, undirected graph converges to the degree distribution of the nodes [36].
— We have shown in [14] that if, in the case of undirected graphs, we compute the PageRank personalized to the degree distribution, then the limit distribution is the degree distribution itself. Therefore, if one computes the PageRank/degree quotient, then we get the same constant for all nodes of the undirected graph.
— If we compute the PageRank/degree quotient in the case of directed graphs, then typically, the results will not be a constant: the larger values will correspond in some sense the relevancy of the node, inherited from the directed structure of the graph. Note that this measure is independent of the degree of the nodes in the undirected graph, so it is believed to compensate small degree nodes against large degree nodes.

In Bánky *et al.* [12], we identified and proposed numerous new protein targets in the directed metabolic networks of pathogenic organisms.

In this work, based on the results of Grolmusz [14], we suggest a unification of the methods of Ivan & Grolmusz [10] and Bánky *et al.* [12] as follows:

— we consider the undirected human interaction graph from IntAct;
— we compute the PageRank, personalized to the set of diabetes-related proteins, taken from UniProt; and
— we have found that the large-degree nodes have very large personalized PageRanks even in this setting (cf. table 1); so we compute the PageRank/degree quotient for each node and would review those that were not personalized to, but have high PageRank/degree quotient (cf. table 2).

## 6. Conclusion

We have performed a refined mathematical algorithm for assigning scores of interest, related to diabetes, in the human interactome, downloaded from the IntAct database. After removing the diabetes-related proteins labelled by UniProt (electronic supplementary material, table S2), we have found proteins with

— well-known relations to diabetes (e.g. galanin receptor type 1 and 2, vasopressin V1a receptor or C1 esterase);
— some very interesting hits with known marginal connections to diabetes (leucine-rich repeat neuronal protein 2, alcohol dehydrogenase iron-containing protein 1, complement C1r subcomponent, 6-phosphofructo-2-kinase, hepatocyte nuclear factor 3-alpha, transcription factor GATA-4); and
— some hits with no known connection to diabetes (small integral membrane protein 3, selenoprotein T, vesicle transport protein, transmembrane protein 19, Krueppel-like factor 13, carcinoembryonic antigen-related cell adhesion molecule 6).

The hits in the first group validate our method. The hits in the second group, with marginal evidence mostly from the last two years, show that our method is capable of finding very recently discovered important proteins in the disease.

The members of the third group show that completely new, still unknown relations between diabetes and these proteins or genes can be searched for with probable success, since other proteins with high score by our method have clear connections.

## Supplementary Material

Table S1 describes the human protein-protein interaction graph used in the analysis;

## Supplementary Material

Table S2 gives the starting/seeding list of diabetes related proteins, originated from annotations of the UniProt database;

## Supplementary Material

Table S3 describes the resulting protein set, with scores and references.

## Supplementary Material

pp.zip

## Data accessibility

Tables S1, S2 and S3 are available as the electronic supplementary material of this article. The Perl script, created by Gábor Iván and first applied and referred to in Ivan & Grolmusz [10] at http://uratim.com/pp.zip, was used here for the PageRank computation and is also available in the electronic supplementary material.

## References

[1] UNESCO Institute for Statistics. 2005 What do bibliometric indicators tell us about world scientific output? UIS Bulletin on Science and Technology Statistics, Issue 2.

[2] Church DM 2011 Modernizing reference genome assemblies. PLoS Biol. 9, e1001091 (doi:10.1371/journal.pbio.1001091) 21750661 10.1371/journal.pbio.1001091 PMC3130012

[3] Zhu F 2012 Therapeutic target database update 2012: a resource for facilitating target-oriented drug discovery. Nucleic Acids Res. 40, D1128–D1136. (doi:10.1093/nar/gkr797) 21948793 10.1093/nar/gkr797 PMC3245130

[4] Sakharkar MK, Li P, Zhong Z, Sakharkar KR 2008 Quantitative analysis on the characteristics of targets with FDA approved drugs. Int. J. Biol. Sci. 4, 15–22. (doi:10.7150/ijbs.4.15) 18167532 10.7150/ijbs.4.15 PMC2140153

[5] Ellis RJ 2001 Macromolecular crowding: obvious but underappreciated. Trends Biochem. Sci. 26, 597–604. (doi:10.1016/S0968-0004(01)01938-7) 11590012 10.1016/s0968-0004(01)01938-7

[6] Kleinberg JM 1998 Authoritative sources in a hyperlinked environment. In Proc. Ninth Annual ACM-SIAM Symposium on Discrete Algorithms, New York, 1998, pp. 668–677. San Francisco, CA: ACM Press.

[7] Brin S, Page L 1998 The anatomy of a large-scale hypertextual web search engine. Comp. Networks ISDN Syst. 30, 107–117. (doi:10.1016/S0169-7552(98)00110-X)

[8] Kamvar S, Haveliwala T 2003 The condition number of the PageRank problem. Technical Report 2003-36. Stanford InfoLab.

[9] Lee HC, Borodin A 2003 Perturbation of the hyperlinked environment. In Computing and Combinatorics: 9th Annual Int. Conf., COCOON 2003, Big Sky, MT, USA, July 25–28, 2003 (eds Warnow T, Zhu B). Notes in Computer Science, no. 2697, pp. 272–283. Springer.

[10] Ivan G, Grolmusz V 2011 When the web meets the cell: using personalized PageRank for analysing protein interaction networks. Bioinformatics 27, 405–407. (doi:10.1093/bioinformatics/btq680) 21149343 10.1093/bioinformatics/btq680

[11] Fortunato S, Boguna M, Flammini A, Menczer F 2008 Approximating PageRank from in-degree. In Algorithms and models for the web-graph, vol. 4936, (Aiello W, Broder A, Janssen J, Milios E). Lecture Notes in Computer Science, pp. 59–71. Berlin, Germany: Springer.

[12] Bánky D, Iván G, Grolmusz V 2013 Equal opportunity for low-degree network nodes: a PageRank-based method for protein target identification in metabolic graphs. PLoS ONE 8, e54204 (doi:10.1371/journal.pone.0054204) 23382878 10.1371/journal.pone.0054204 PMC3558500

[13] Tothmeresz L, Grolmusz V 2013 Characterizing the functional similarity of enzymes with high co-citation in interaction networks. Protein Pept. Lett. 20, 1181–1187. (doi:10.2174/0929866511320100013) 22973847 10.2174/0929866511320100013

[14] Grolmusz V 2015 A note on the PageRank of undirected graphs. Inform. Process. Lett. 115, 633–634. (doi:10.1016/j.ipl.2015.02.015)

[15] Mansouri S, Barde S, Ortsater H, Eweida M, Darsalia V, Langel U, Sjoholm A, Hokfelt T, Patrone C 2013 GalR3 activation promotes adult neural stem cell survival in response to a diabetic milieu. J. Neurochem. 127, 209–220. (doi:10.1111/jnc.12396) 23927369 10.1111/jnc.12396

[16] Miyata S, Yamada N, Kawada T 2012 Possible involvement of hypothalamic nucleobindin-2 in hyperphagic feeding in Tsumura Suzuki obese diabetes mice. Biol. Pharm. Bull. 35, 1784–1793. (doi:10.1248/bpb.b12-00505) 23037168 10.1248/bpb.b12-00505

[17] Fang P, Yu M, Shi M, Zhang Z, Sui Y, Guo L, Bo P 2012 Galanin peptide family as a modulating target for contribution to metabolic syndrome. Gen. Comp. Endocrinol. 179, 115–120. (doi:10.1016/j.ygcen.2012.07.029) 22909974 10.1016/j.ygcen.2012.07.029

[18] Minchenko DO, Kharkova AP, Hubenia OV, Minchenko OH 2013 Insulin receptor, IRS1, IRS2, INSIG1, INSIG2, RRAD, and BAIAP2 gene expressions in glioma U87 cells with ERN1 loss of function: effect of hypoxia and glutamine or glucose deprivation. Endocr. Regul. 47, 15–26. (doi:10.4149/endo_2013_01_15) 23363253 10.4149/endo_2013_01_15

[19] Gu J, Orr N, Park SD, Katz LM, Sulimova G, MacHugh DE, Hill EW 2009 A genome scan for positive selection in thoroughbred horses. PLoS ONE 4, e5767 (doi:10.1371/journal.pone.0005767) 19503617 10.1371/journal.pone.0005767 PMC2685479

[20] Bauersachs S, Mitko K, Ulbrich SE, Blum H, Wolf E 2008 Transcriptome studies of bovine endometrium reveal molecular profiles characteristic for specific stages of estrous cycle and early pregnancy. Exp. Clin. Endocrinol. Diabetes 116, 371–384. (doi:10.1055/s-2008-1076714) 18561091 10.1055/s-2008-1076714

[21] Al Safar HS, Cordell HJ, Jafer O, Anderson D, Jamieson SE, Fakiola M, Khazanehdari K, Tay GK, Blackwell JM 2013 A genome-wide search for type 2 diabetes susceptibility genes in an extended Arab family. Ann. Hum. Genet. 77, 488–503. (doi:10.1111/ahg.12036) 23937595 10.1111/ahg.12036

[22] Van Kirk CA, VanGuilder HD, Young M, Farley JA, Sonntag WE, Freeman WM 2011 Age-related alterations in retinal neurovascular and inflammatory transcripts. Mol. Vis. 17, 1261– 1274. 21633715 PMC3103744

[23] Atsumi T, Nishio T, Niwa H, Takeuchi J, Bando H, Shimizu C, Yoshioka N, Bucala R, Koike T 2005 Expression of inducible 6-phosphofructo-2-kinase/fructose-2,6-bisphosphatase/pfkfb3 isoforms in adipocytes and their potential role in glycolytic regulation. Diabetes 54, 3349–3357. (doi:10.2337/diabetes.54.12.3349) 16306349 10.2337/diabetes.54.12.3349

[24] Garcia-Herrero CM, Galan M, Vincent O, Flandez B, Gargallo M, Delgado-Alvarez E, Blazquez E, Navas MA 2007 Functional analysis of human glucokinase gene mutations causing MODY2: exploring the regulatory mechanisms of glucokinase activity. Diabetologia 50, 325–333. (doi:10.1007/s00125-006-0542-7) 17186219 10.1007/s00125-006-0542-7

[25] Rodriguez-Segui S, Akerman I, Ferrer J 2012 Gata believe it: new essential regulators of pancreas development. J. Clin. Invest. 122, 3469–3471. (doi:10.1172/JCI65751) 23006323 10.1172/JCI65751 PMC3461932

[26] Sangani R 2013 Regulation of vitamin C transporter in the type 1 diabetic mouse bone and bone marrow. Exp. Mol. Pathol. 95, 298–306. (doi:10.1016/j.yexmp.2013.08.007) 23999113 10.1016/j.yexmp.2013.08.007

[27] Hill NJ, Lyons PA, Armitage N, Todd JA, Wicker LS, Peterson LB 2000 NOD Idd5 locus controls insulitis and diabetes and overlaps the orthologous CTLA4/IDDM12 and NRAMP1 loci in humans. Diabetes 49, 1744–1747. (doi:10.2337/diabetes.49.10.1744) 11016460 10.2337/diabetes.49.10.1744

[28] Kerrien S 2007 IntAct–open source resource for molecular interaction data. Nucleic Acids Res. 35, D561–D565. (doi:10.1093/nar/gkl958) 17145710 10.1093/nar/gkl958 PMC1751531

[29] UniProt Consortium. 2010 The Universal Protein Resource (UniProt) in 2010. Nucleic Acids Res. 38, D142–D148. (doi:10.1093/nar/gkp846) 19843607 10.1093/nar/gkp846 PMC2808944

[30] Liao C-S, Lu K, Baym M, Singh R, Berger B 2009 Isorankn: spectral methods for global alignment of multiple protein networks. Bioinformatics 25, i253–i258. (doi:10.1093/bioinformatics/btp203) 19477996 10.1093/bioinformatics/btp203 PMC2687957

[31] Banky D, Ordog R, Grolmusz V 2009 NASCENT: an automatic protein interaction network generation tool for non-model organisms. Bioinformation 3, 361–363. (doi:10.6026/97320630003361) 19707301 10.6026/97320630003361 PMC2720673

[32] Franceschini A 2013 String v9.1: protein–protein interaction networks, with increased coverage and integration. Nucleic Acids Res. 41, D808–D815. (doi:10.1093/nar/gks1094) 23203871 10.1093/nar/gks1094 PMC3531103

[33] Chatraryamontri A, Ceol A, Palazzi LM, Nardelli G, Schneider MV, Castagnoli L, Cesareni G 2007 MINT: the molecular interaction database. Nucleic Acids Res. 35, D572–D574. (doi:10.1093/nar/gkl950) 17135203 10.1093/nar/gkl950 PMC1751541

[34] KeshavaPrasad TS, Kandasamy K, Pandey A 2009 Human protein reference database and human proteinpedia as discovery tools for systems biology. Methods Mol. Biol. 577, 67–79. (doi:10.1007/978-1-60761-232-2_6) 19718509 10.1007/978-1-60761-232-2_6

[35] Salwinski L, Miller CS, Smith AJ, Pettit FK, Bowie JU, Eisenberg D 2004 The database of interacting proteins: 2004 update. Nucleic Acids Res. 32, D449–D451. (doi:10.1093/nar/gkh086) 14681454 10.1093/nar/gkh086 PMC308820

[36] Lovász L 1993 Random walks on graphs: a survey. In Combinatorics, Paul Erdos is Eighty. Bolyai Society Mathematical Studies. Janos Bolyai Mathematical Society.

[37] Page L, Brin S, Motwani R, Winograd T 1999 The PageRank citation ranking: bringing order to the web.

