# Supplementary material for: Identifying diabetes-related important protein targets with few interacting partners with the PageRank algorithm
Source: R Soc Open Sci. 2015 Apr 29;2(4):140252. doi: 10.1098/rsos.140252 (PMC4448867; doi:10.1098/rsos.140252)
Supplement: Table S1 describes the human protein-protein interaction graph used in the analysis; [file rsos140252supp1.docx]

# Table S1

The list of undirected edges from the IntAct database; given in three columns,

Q96CV9 A0AUZ9

Q92731 A0AV96

Q96AV8 A0AVK6

O15198 A0AVT1

P02751 A0FGR8

Q13286 A0FGR8

Q8TD08 A0FGR8

Q96RR4 A0FGR8

A0JLT2 A0JLT2

O95402 A0JLT2

P25963 A0JLT2

P27707 A0JLT2

P28360 A0JLT2

Q75LH2 A0JLT2

Q9BTT4 A0JLT2

Q9BWU1 A0JLT2

Q9NWA0 A0JLT2

Q9NX70 A0JLT2

P54253 A0JNW5

Q9H081 A0M8Q9

Q9Y6K9 A0MZ66

Q99623 A0N4V7

Q9UFF9 A0N4V7

O75381 A0PJ54

P05141 A0PJW6

Q9Y2R0 A0PJW6

P60953 A0S183

P25054 A0S2M1

P36873 A1DRY3

Q9UGN5 A1KXE4

P00533 A1L0T0

P01106 A1L0T0

P12956 A1L0T0

Q13418 A1L0T0

A1X283 A1X283

P62993 A1X283

Q92624 A1XBS5

Q99459 A1YPR0

Q5U5Q3 A2A3R5

Q8NB14 A2RU54

Q96FJ0 A2RU54

P01106 A2RUS2

Q9H492 A2RUS2

P01106 A2VDJ0

P45973 A2VDJ6

Q14145 A3KMH1

Q8WZ19 A4D0S4

Q14197 A4D1E9

Q5VV17 A4D1J9

P00533 A4FU49

P52756 A4FU77

P39748 A4GXA9

P04637 A4KVA4

P51532 A4PIV7

P15336 A4PIV8

Q04724 A4PIV8

P51532 A4PIV9

O75530 A4PIW0

P15336 A4PIW0

Q04724 A4PIW0

Q15022 A4PIW0

Q15910 A4PIW0

O75923 A4UGR9

Q92636 A4UGR9

Q53EZ4 A5D8V6

O14775 A5PKW4

P0CG48 A5PKW4

P23416 A5PKW4

Q96S21 A5PKW4

P49841 A5PL33

P53350 A5PL33

A5YKK6 A5YKK6

O75381 A5YKK6

P02751 A5YKK6

P02768 A5YKK6

P10828 A5YKK6

P19320 A5YKK6

P19793 A5YKK6

P62993 A5YKK6

Q13573 A5YKK6

Q8NDV7 A5YKK6

Q99459 A5YKK6

Q9HCJ0 A5YKK6

Q9UFF9 A5YKK6

Q9UIV1 A5YKK6

Q9UKV8 A5YKK6

Q9UKZ1 A5YKK6

Q9ULM6 A5YKK6

Q9UPQ9 A5YKK6

Q9UPT9 A5YKK6

Q14669 A5YM72

P10276 A6H8Y1

Q9HAU0 A6NC98

Q9GZQ8 A6NCE7

Q9H0Y0 A6NCE7

Q9NT62 A6NCE7

P48729 A6ND36

P84022 A6ND36

Q15796 A6ND36

Q969H0 A6NE52

O00257 A6NEJ1

O00401 A6NGB9

P62993 A6NGB9

P07900 A6NHL2

Q9P2Y5 A6NHL2

Q92731 A6NHQ2

P02751 A6NHR9

P19320 A6NHR9

Q13573 A6NHR9

Q99459 A6NHR9

P48023 A6NJZ7

Q9P0W5 A6NK53

P19320 A6NKE1

O15265 A6NKH4

Q15051 A6NKN8

P62837 A6NLP3

O94782 A6NMY6

P03372 A6NMY6

P16104 A6NMY6

Q70EK8 A6NMY6

Q9H0R8 A6NMY6

P16104 A6NN80

P19320 A6NNE7

Q9UPT9 A6NNY8

P49674 A7E2V4

Q8NB12 A7E2Y1

Q92731 A7E2Y1

P06241 A7KAX9

P31947 A7KAX9

P49407 A7KAX9

P60953 A7KAX9

A7MCY6 A7MCY6

O96018 A7MCY6

Q13137 A7MCY6

Q9UHD2 A7MCY6

Q9UKV8 A7MCY6

Q92731 A8CG34

P62258 A8K0R7

Q9H4A3 A8K0R7

Q9Y3A3 A8K3H8

Q15744 A8K673

P13569 A8K7I4

P08727 A8K8P3

P41208 A8K8P3

P62136 A8K8P3

Q96JH8 A8K8P3

Q99459 A8K8P3

Q8TB68 A8K8V0

O95758 A8K979

O00257 A8MPV3

O00555 A8MQ03

Q8N2Z9 A8MT69

Q15154 A8MTZ0

Q9H0F7 A8MTZ0

P45973 A8MTY0

P62805 A8MW92

P68431 A8MW92

Q92851 A8MW92

Q9H0M0 A8MW92

Q9NX58 A8MW92

P62136 A8MXD5

P08473 A8MY76

Q9NUX5 A8MZ97

O00459 A9UF07

O15357 A9UF07

P22681 A9UF07

P29353 A9UF07

P46108 A9UF07

P62993 A9UF07

Q8TF42 A9UF07

Q96CW1 A9UF07

P37231 A9UHW6

P56524 A9UHW6

P60709 B0I1T2

O15372 B0QY89

O15265 B0YJA4

P19320 B1AKQ8

P62993 B1ALK7

Q8TAF3 B1AM27

P55072 B1AQ61

P04637 B2R5M9

P04637 B2R6D7

Q14197 B2R894

Q969T4 B2RDW1

Q7KZI7 B2RTR1

P01106 B2RTY4

P60953 B2RTY4

Q00005 B2RTY4

P01106 B2RUU2

B2RWN4 B2RWN4

Q08AG7 B2RWN4

Q6NZ67 B2RWN4

Q96CW5 B2RWN4

Q9BVR6 B2RWN4

O95758 B2RWP9

P09874 B2Y833

P11308 B2Y833

P01106 B2ZZ90

Q96SB4 B3KMQ7

Q9NTG7 B3KMS0

P01100 B3KNK9

P16220 B3KQF8

P63279 B3KQF8

O15372 B3KSH1

Q8TAF3 B3KSH1

Q9Y6I4 B3KSH1

O94941 B3KSH4

P50876 B3KSH4

Q13064 B3KSH4

Q6UWE0 B3KSH4

Q86Y13 B3KSH4

Q8TDB6 B3KSH4

Q96CA5 B3KSH4

Q99496 B3KSH4

Q99942 B3KSH4

Q9BWF2 B3KSH4

Q9BYJ4 B3KSH4

Q9BYM8 B3KSH4

Q9C035 B3KSH4

Q9H000 B3KSH4

Q9H4P4 B3KSH4

Q9H992 B3KSH4

Q9NZS9 B3KSH4

Q9ULV8 B3KSH4

Q9Y508 B3KSH4

Q9NZL4 B3KTT5

Q5U5Q3 B3KX72

O95229 B3KY54

Q5U5Q3 B4DHC4

P19320 B4DKZ6

Q06187 B4DMH3

Q7L5N1 B4DN30

Q92905 B4DN30

Q93009 B4DN30

P33993 B4DN52

P19320 B4DNJ6

O75381 B4DP54

Q8N6M0 B4DPD5

Q96FJ0 B4DPD5

Q9Y2R0 B4DRS6

Q15051 B4DRT3

P19320 B4DTG2

Q96EB6 B4DTG2

P19320 B4DVB8

P04637 B4DVN3

P19320 B4DX78

P68400 B4DY08

P38606 B4E040

Q5U5Q3 B4E083

P19320 B4E1G6

P38606 B4E1J8

Q5U5Q3 B4E2W0

P19320 B4E386

Q5U5Q3 B5MCW2

Q96RL1 B5MD87

Q9NWV8 B5MD87

P53350 B5MDQ0

Q5U5Q3 B7Z4B8

Q5U5Q3 B7Z4V2

P19320 B7Z5V2

Q92995 B7Z5W8

Q96RL1 B7Z5W8

P38606 B7Z6L9

B2Y833 B7Z722

Q5U5Q3 B7Z7T5

O00555 B7ZLY3

P28702 B9A6J3

O00555 B9EG76

C3PTT6 C3PTT6

O00206 C3PTT6

O60602 C3PTT6

O60603 C3PTT6

P20340 C3PTT6

P61006 C3PTT6

P61073 C3PTT6

P61626 C3PTT6

Q8N9I0 C3PTT6

Q9BZL6 C3PTT6

Q9Y4E8 C4AM86

Q5U5Q3 C9J9K3

P10588 C9JCN9

P19320 C9JNW5

Q5U5Q3 C9JXB8

P46736 C9JYE9

O00231 D3DR86

O14920 D3DR86

O15027 D3DR86

O15111 D3DR86

O43242 D3DR86

O95816 D3DR86

P04350 D3DR86

P07437 D3DR86

P07900 D3DR86

P08238 D3DR86

P0CG47 D3DR86

P11021 D3DR86

P11142 D3DR86

P12277 D3DR86

P13645 D3DR86

P15924 D3DR86

P17066 D3DR86

P17858 D3DR86

P19838 D3DR86

P25788 D3DR86

P25963 D3DR86

P27708 D3DR86

P28074 D3DR86

P30153 D3DR86

P30154 D3DR86

P31943 D3DR86

P34931 D3DR86

P35579 D3DR86

P38646 D3DR86

P41252 D3DR86

P49368 D3DR86

P51532 D3DR86

P51665 D3DR86

P58107 D3DR86

P60900 D3DR86

P62158 D3DR86

P62191 D3DR86

P62195 D3DR86

P63208 D3DR86

P63261 D3DR86

Q00610 D3DR86

Q00653 D3DR86

Q01201 D3DR86

Q04206 D3DR86

Q05639 D3DR86

Q08380 D3DR86

Q13114 D3DR86

Q13200 D3DR86

Q13748 D3DR86

Q14204 D3DR86

Q15653 D3DR86

Q3ZCQ8 D3DR86

Q8N668 D3DR86

Q92734 D3DR86

Q93008 D3DR86

Q969H0 D3DR86

Q99460 D3DR86

Q99558 D3DR86

Q9BSJ2 D3DR86

Q9BVA1 D3DR86

Q9H1R3 D3DR86

Q9UKB1 D3DR86

Q9UNM6 D3DR86

Q9UQC1 D3DR86

Q9Y230 D3DR86

Q9Y265 D3DR86

Q9Y297 D3DR86

Q9Y6K9 D3DR86

Q5U5Q3 D6REM6

Q5U5Q3 D6RG13

D6RGH6 D6RGH6

Q5U5Q3 E5RGV0

Q5U5Q3 E5RH77

P38606 E7EMK3

P62993 E7EQR4

Q5U5Q3 E7EQV3

P38606 E7ETZ0

P62993 E7EVF4

P19320 E9PAU2

P19320 E9PB61

P62993 E9PBA6

Q5U5Q3 E9PD53

Q5U5Q3 E9PDU5

Q5U5Q3 E9PH82

Q5U5Q3 E9PIN3

Q5U5Q3 E9PLA9

Q5U5Q3 E9PPU1

Q5U5Q3 E9PQD7

P19320 F2Z393

Q5U5Q3 F5GX33

Q5U5Q3 F5GYC1

Q5U5Q3 F5H456

P11441 F6VEM6

Q99459 F8W881

P78362 G3V5S9

P02730 G3XAG3

P19320 H0YHG0

P32121 I6L957

Q9Y230 O00115

Q9Y265 O00115

O75381 O00116

Q676U5 O00116

Q96DB2 O00116

P10242 O00139

P51858 O00139

A0N4V7 O00141

F5H284 O00141

O00303 O00141

O15067 O00141

O75390 O00141

O75828 O00141

O95202 O00141

O95257 O00141

P01833 O00141

P02788 O00141

P04406 O00141

P11940 O00141

P12273 O00141

P12882 O00141

P14866 O00141

P15311 O00141

P16220 O00141

P22234 O00141

P26196 O00141

P26640 O00141

P30049 O00141

P39023 O00141

P43355 O00141

P49321 O00141

P49411 O00141

P50395 O00141

P52272 O00141

P55072 O00141

P60900 O00141

P61247 O00141

Q13347 O00141

Q13435 O00141

Q14697 O00141

Q15008 O00141

Q16543 O00141

Q16658 O00141

Q52LJ0 O00141

Q6NVI1 O00141

Q8TEX9 O00141

Q9H8S9 O00141

Q9NTK5 O00141

Q9NVA4 O00141

Q9UI14 O00141

Q9UQ80 O00141

Q9Y4L1 O00141

P38919 O00148

P61326 O00148

P78362 O00148

P82979 O00148

Q05397 O00148

Q13573 O00148

Q8IY92 O00148

Q92731 O00148

Q99459 O00148

Q9NWV8 O00148

Q9UKV8 O00148

Q9UL18 O00148

P63272 O00151

O00716 O00159

P03372 O00159

P22087 O00159

P29353 O00159

P32121 O00159

P33993 O00159

P49407 O00159

P62993 O00159

Q14240 O00159

Q15051 O00159

Q8IY92 O00159

Q92731 O00159

Q9GZS1 O00159

Q9Y2K6 O00159

Q00978 O00160

O75558 O00161

O76038 O00161

P02751 O00161

P43243 O00161

P46459 O00161

P51116 O00161

P54920 O00161

Q8TAC9 O00161

Q9NYB9 O00161

Q9P2A4 O00161

P13569 O00165

P27694 O00165

P53779 O00165

P62877 O00165

Q14451 O00165

Q86Z02 O00165

Q8TAQ5 O00165

Q9P1U0 O00165

Q15475 O00167

Q93062 O00167

O00408 O00170

P00533 O00170

P08238 O00170

P35869 O00170

P50750 O00170

P61244 O00170

P68400 O00170

Q92985 O00170

Q9Y4P8 O00170

P51858 O00178

Q13568 O00178

Q99459 O00178

P02751 O00186

P39023 O00186

P01023 O00193

P10074 O00193

P21246 O00193

Q14703 O00193

Q75N90 O00193

Q7KZI7 O00193

Q8IY92 O00193

Q8TAF3 O00193

Q8WWI5 O00193

Q9NV58 O00193

Q9P283 O00193

Q9Y6H5 O00193

Q07021 O00198

Q07817 O00198

O95758 O00203

Q13573 O00203

Q96Q35 O00204

O00206 O00206

P58753 O00206

P84022 O00206

Q86XR7 O00206

Q99836 O00206

Q9UHV2 O00206

Q9Y6Y9 O00206

O43157 O00212

O60610 O00212

Q14623 O00212

Q9Y4D1 O00212

P00533 O00213

P05067 O00213

Q07954 O00213

O00214 O00214

Q13137 O00214

Q15583 O00214

O43678 O00217

P40692 O00217

P55040 O00217

Q14197 O00217

P50591 O00220

Q13158 O00220

Q14790 O00220

Q96P48 O00220

D3DR86 O00221

O00221 O00221

O00743 O00221

O15084 O00221

O15111 O00221

O75170 O00221

O75179 O00221

O95816 O00221

P04350 O00221

P07437 O00221

P07814 O00221

P07900 O00221

P08238 O00221

P11021 O00221

P11142 O00221

P19838 O00221

P23246 O00221

P27708 O00221

P28066 O00221

P34931 O00221

P38646 O00221

P41252 O00221

P54136 O00221

P55072 O00221

P61289 O00221

P62158 O00221

P63208 O00221

P78527 O00221

Q00610 O00221

Q00653 O00221

Q04206 O00221

Q04864 O00221

Q07021 O00221

Q13748 O00221

Q3ZCQ8 O00221

Q5H9R7 O00221

Q8IWG5 O00221

Q8IWZ3 O00221

Q8WVZ9 O00221

Q92974 O00221

Q9BZF9 O00221

Q9UKB1 O00221

Q9UPN7 O00221

Q9UQC1 O00221

Q9Y230 O00221

Q9Y6K9 O00221

O00487 O00231

O60383 O00231

O75832 O00231

P02751 O00231

P04406 O00231

P04637 O00231

P19320 O00231

P21246 O00231

P26641 O00231

P51665 O00231

P54257 O00231

P54578 O00231

P61956 O00231

P62195 O00231

P68104 O00231

P84022 O00231

Q04724 O00231

Q05516 O00231

Q13432 O00231

Q13573 O00231

Q13885 O00231

Q14194 O00231

Q15047 O00231

Q15262 O00231

Q15796 O00231

Q5RL73 O00231

Q5S007 O00231

Q5UIP0 O00231

Q7L5N1 O00231

Q96ID5 O00231

Q96LA8 O00231

Q99459 O00231

Q9BQ83 O00231

Q9GZT6 O00231

Q9H7U1 O00231

Q9NPI1 O00231

Q9NWV8 O00231

Q9UKY1 O00231

Q9Y2K6 O00231

Q9Y3C7 O00231

Q9Y4E8 O00231

Q9Y5K5 O00231

O00487 O00232

O75832 O00232

P19320 O00232

P51665 O00232

P54578 O00232

P54727 O00232

P55036 O00232

P60228 O00232

Q13573 O00232

Q16401 O00232

Q92731 O00232

Q99459 O00232

Q9NWV8 O00232

Q9Y2K6 O00232

Q9Y5K5 O00232

P19320 O00233

P62333 O00233

P61077 O00237

P62256 O00237

P62837 O00237

Q96B02 O00237

P43026 O00238

O43914 O00241

P00519 O00254

P06241 O00254

P12931 O00254

P16333 O00254

P19174 O00254

O14757 O00255

P04637 O00255

P35579 O00255

P61964 O00255

Q03164 O00255

Q9BXW9 O00255

Q9H4W6 O00255

Q9Y5Z7 O00255

O43463 O00257

P35226 O00257

P35227 O00257

Q9HC52 O00257

O00257 O00257

P62258 O00257

P63104 O00257

P67870 O00257

P68400 O00257

Q8IXK0 O00257

Q99496 O00257

Q9BYE7 O00257

P10632 O00264

P16435 O00264

P28288 O00264

P30480 O00264

P39656 O00264

P48039 O00264

Q86Y56 O00264

Q9BUP0 O00264

Q9H9B4 O00264

Q9Y3D7 O00264

A0AUL9 O00267

O95219 O00267

P02751 O00267

P16104 O00267

P24928 O00267

P62917 O00267

P62937 O00267

P63272 O00267

Q12789 O00267

Q12907 O00267

Q13123 O00267

Q16643 O00267

Q66LE6 O00267

Q6GVN4 O00267

Q92561 O00267

Q9H3P2 O00267

Q9NRX1 O00267

Q9P0U0 O00267

Q9P2H0 O00267

Q9UHR5 O00267

Q9Y3C7 O00267

O95402 O00268

P20226 O00268

P42858 O00268

P61964 O00268

Q12962 O00268

Q5S007 O00268

Q92830 O00268

Q9Y4A5 O00268

O00273 O00273

O76075 O00273

P13584 O00273

P14866 O00273

P23508 O00273

P54646 O00273

P61981 O00273

P63104 O00273

Q01844 O00273

Q15029 O00273

Q5S007 O00273

Q8WZ42 O00273

Q96NT1 O00273

Q9P2Y5 O00273

Q9Y4E8 O00273

O95782 O00291

P19320 O00291

P42858 O00291

P53675 O00291

Q9H6R3 O00292

P06241 O00294

P62993 O00294

O15116 O00299

P05386 O00299

P10827 O00299

P15336 O00299

P19320 O00299

P20292 O00299

P24539 O00299

P30480 O00299

P32121 O00299

P37198 O00299

P40692 O00299

Q13162 O00299

Q9H0R8 O00299

Q9NR11 O00299

Q9Y6K9 O00299

O00165 O00303

O00571 O00303

O15198 O00303

O60832 O00303

P02751 O00303

P0CG48 O00303

P21127 O00303

P21246 O00303

P30504 O00303

P55884 O00303

P68104 O00303

Q53GS7 O00303

Q7L2H7 O00303

Q8IWF7 O00303

Q92574 O00303

Q92900 O00303

Q969P5 O00303

P21246 O00305

P48023 O00305

Q12788 O00305

Q9Y3C7 O00305

P21579 O00305

P78559 O00305

Q9BX90 O00305

O00308 O00308

O15105 O00308

O43516 O00308

O94855 O00308

P54259 O00308

P78563 O00308

P84022 O00308

Q01860 O00308

Q15796 O00308

Q9BTC0 O00308

Q9BUZ4 O00308

Q9H469 O00308

Q9Y3C5 O00308

O43913 O00311

P11802 O00311

P49736 O00311

P50750 O00311

Q13111 O00311

Q9BTE3 O00311

Q9UBU7 O00311

Q9Y463 O00327

P01112 O00329

P11049 O00329

P16333 O00329

P27986 O00329

P09622 O00330

Q14527 O00338

O00555 O00339

O15265 O00339

O75081 O00339

Q92997 O00339

Q8TAQ5 O00341

Q9BUH8 O00391

O00399 O00399

P08865 O00399

P53350 O00399

O00410 O00401

O15143 O00401

O43516 O00401

O60504 O00401

O94868 O00401

P00738 O00401

P06239 O00401

P16070 O00401

P16333 O00401

P29692 O00401

P46108 O00401

P60953 O00401

P61160 O00401

P62993 O00401

Q15811 O00401

Q8TF74 O00401

Q8WZ75 O00401

Q99828 O00401

Q9H0M0 O00401

Q9H8N7 O00401

Q9Y5X1 O00401

O00170 O00408

O95166 O00410

O95758 O00410

P02751 O00410

P18124 O00410

P19320 O00410

P60520 O00410

Q13573 O00410

Q70EL3 O00410

Q7Z6M2 O00410

Q92560 O00410

Q92769 O00410

Q96EV8 O00410

Q99459 O00410

Q9GZQ8 O00410

Q9H0R8 O00410

Q9H5J8 O00410

Q9Y376 O00410

P51858 O00411

Q14197 O00411

Q8WVZ9 O00411

P14635 O00418

P49748 O00418

Q9UBS0 O00418

O43665 O00422

O75528 O00422

O75964 O00422

O94925 O00422

P04792 O00422

P04844 O00422

P15170 O00422

P17661 O00422

P19784 O00422

P31153 O00422

P31689 O00422

P38919 O00422

P39656 O00422

P46459 O00422

P54577 O00422

P57721 O00422

P60228 O00422

P60520 O00422

P61326 O00422

Q13547 O00422

Q14141 O00422

Q14498 O00422

Q16740 O00422

Q6NXE6 O00422

Q86Y56 O00422

Q8N1F7 O00422

Q969V3 O00422

Q96EK5 O00422

Q9BXB5 O00422

Q9H993 O00422

Q9UBB4 O00422

Q9UMR2 O00422

Q9UPN3 O00422

Q9H9L3 O00423

O95166 O00425

O95503 O00425

P01106 O00425

P02751 O00425

P19525 O00425

P60520 O00425

Q96DB2 O00425

Q9H0R8 O00425

Q9HC52 O00425

Q9UL18 O00425

O00429 O00429

O75381 O00429

P03372 O00429

P10809 O00429

P62256 O00429

Q07817 O00429

Q13573 O00429

Q5S007 O00429

Q96C03 O00429

Q96HS1 O00429

Q99459 O00429

Q9NQG6 O00429

Q9NTG7 O00429

P18077 O00442

P20700 O00442

P22087 O00442

P35606 O00442

P46779 O00442

P47914 O00442

P61254 O00442

P62280 O00442

P62750 O00442

P62910 O00442

Q02543 O00442

Q70EL2 O00442

Q99459 O00442

Q9BUJ2 O00442

Q9HC36 O00442

Q9NR30 O00442

Q9Y3B4 O00442

Q9Y3U8 O00442

O00443 O00443

O95630 O00443

P00533 O00443

P04626 O00443

P32121 O00443

P49407 O00443

Q4VCS5 O00443

O00444 O00444

P31947 O00444

Q12873 O00444

Q5JR59 O00444

Q5VWP2 O00444

Q9HC77 O00444

Q7L622 O00453

Q8WUQ7 O00453

P19525 O00458

Q92963 O00458

A9UF07 O00459

O15357 O00459

P00533 O00459

P01116 O00459

P04626 O00459

P08069 O00459

P08631 O00459

P09619 O00459

P10721 O00459

P12931 O00459

P19320 O00459

P21860 O00459

P22681 O00459

P29353 O00459

P42336 O00459

P42338 O00459

P46108 O00459

P49407 O00459

P62993 O00459

Q05397 O00459

Q8IZP0 O00459

Q9H204 O00459

Q9NY15 O00459

O00463 O00463

O14836 O00463

O43734 O00463

P04632 O00463

P14373 O00463

P25942 O00463

P49069 O00463

Q00987 O00463

Q02223 O00463

Q07955 O00463

Q13287 O00463

Q15326 O00463

Q92956 O00463

Q9BZR9 O00463

Q9Y4K3 O00463

O00555 O00468

P05067 O00468

P30101 O00468

P01106 O00469

Q9H1Y0 O00469

P14921 O00470

P16220 O00470

P31314 O00470

P55854 O00470

O14787 O00471

P05129 O00471

P19022 O00471

P56693 O00471

Q13573 O00471

Q86TW2 O00471

Q99459 O00471

Q9UL17 O00471

A0JLT2 O00472

O95402 O00472

P42568 O00472

P50750 O00472

Q96JC9 O00472

Q9BTT4 O00472

Q9UHB7 O00472

P07711 O00478

Q9NVJ2 O00478

P04155 O00481

O60869 O00482

O43678 O00483

O95166 O00483

P00533 O00483

P04626 O00483

P15735 O00483

Q13418 O00483

Q96Q40 O00483

Q9H0R8 O00483

Q9H492 O00483

Q9UJS0 O00483

Q9UKV8 O00483

P51665 O00487

P54578 O00487

Q16401 O00487

Q5VTR2 O00487

Q75LH2 O00487

Q8NDT2 O00487

Q96CS7 O00487

Q9NWV8 O00487

Q9Y5K5 O00487

O43318 O00488

O95219 O00499

P09467 O00499

P62993 O00499

Q05193 O00499

Q13426 O00499

Q8NFH8 O00499

Q8TB24 O00499

Q9UBW5 O00499

Q9UKG1 O00499

Q9Y2H0 O00499

P10636 O00499

P19447 O00505

P45973 O00505

P46527 O00505

P83916 O00505

Q13255 O00505

Q13547 O00505

Q16236 O00505

Q8IY92 O00505

Q96EB6 O00505

Q9H9J4 O00505

O00506 O00506

O43815 O00506

P61077 O00506

Q08379 O00506

Q13033 O00506

Q5VSL9 O00506

Q8WZ74 O00506

Q9BUL8 O00506

Q9P275 O00506

Q9P289 O00506

Q9P2B4 O00506

Q9Y228 O00506

Q9Y376 O00506

Q9Y3A3 O00506

Q9Y6E0 O00506

Q9Y6K9 O00506

P29353 O00507

P49674 O00507

Q96EB6 O00507

Q9NWV8 O00507

P35222 O00512

Q6P1J9 O00512

Q9BRQ0 O00512

Q9Y3Y4 O00512

P33993 O00515

Q92731 O00515

P08473 O00519

Q9BSQ5 O00522

P19320 O00541

Q14137 O00541

Q92731 O00541

Q96DB2 O00541

A6NFL8 O00555

A6NK53 O00555

A7MD48 O00555

O00339 O00555

O43609 O00555

O60229 O00555

O75525 O00555

O75953 O00555

O94910 O00555

O95153 O00555

O95169 O00555

P07942 O00555

P08708 O00555

P15822 O00555

P22307 O00555

P22695 O00555

P23142 O00555

P41182 O00555

P42679 O00555

P49750 O00555

P62158 O00555

P67870 O00555

P98160 O00555

Q07954 O00555

Q07955 O00555

Q12805 O00555

Q13427 O00555

Q14008 O00555

Q14152 O00555

Q15714 O00555

Q5JRA6 O00555

Q6IAU3 O00555

Q76N89 O00555

Q7Z5H3 O00555

Q7Z7M0 O00555

Q86SJ2 O00555

Q86UR1 O00555

Q86X94 O00555

Q8IXH7 O00555

Q8IZP0 O00555

Q8N1B4 O00555

Q8N2S1 O00555

Q8NBJ7 O00555

Q8TAK6 O00555

Q92824 O00555

Q92832 O00555

Q92871 O00555

Q96A73 O00555

Q96KQ7 O00555

Q99435 O00555

Q9BXJ1 O00555

Q9BZL4 O00555

Q9NZV1 O00555

Q9Y219 O00555

Q9Y4R8 O00555

A4D1P6 O00560

O00560 O00560

O75084 O00560

P19320 O00560

P30203 O00560

P68400 O00566

A0JLT2 O00567

P01106 O00567

P03372 O00567

P19320 O00567

P32121 O00567

P33993 O00567

P68400 O00567

Q16637 O00567

Q92731 O00567

Q96DB2 O00567

Q9UHK0 O00567

Q9UL18 O00567

Q9UPE1 O00567

Q9Y2X3 O00567

A0JLT2 O00571

B2Y833 O00571

O15264 O00571

O43502 O00571

O75554 O00571

O95166 O00571

O95786 O00571

P01106 O00571

P03372 O00571

P06730 O00571

P11940 O00571

P19320 O00571

P23588 O00571

P31483 O00571

P32121 O00571

P33993 O00571

P38919 O00571

P55884 O00571

P60520 O00571

P60709 O00571

P61326 O00571

P63104 O00571

Q00005 O00571

Q04637 O00571

Q06187 O00571

Q09161 O00571

Q13573 O00571

Q14164 O00571

Q15003 O00571

Q15717 O00571

Q5U5Q3 O00571

Q7Z434 O00571

Q7Z6M2 O00571

Q92731 O00571

Q99459 O00571

Q9BTT4 O00571

Q9GZQ8 O00571

Q9H0R8 O00571

Q9H492 O00571

Q9NX70 O00571

Q9UBU9 O00571

P32248 O00585

Q9NRR5 O00585

P46940 O00592

O95229 O00622

P01106 O00622

P40855 O00623

P50542 O00623

P02511 O00626

P48506 O00626

O60341 O00628

O95402 O00629

P01106 O00629

P45973 O00629

P46063 O00629

P63000 O00629

P68400 O00629

P83916 O00629

Q13185 O00629

Q13547 O00629

Q8IY92 O00629

P54253 O00635

Q13546 O00635

Q5VVX9 O00635

P06400 O00716

P41182 O00716

O15084 O00743

O43318 O00743

O75170 O00743

O75663 O00743

P01106 O00743

P60510 O00743

P78318 O00743

Q9UPN7 O00743

O14543 O00746

O95777 O00746

Q14197 O00746

Q15645 O00746

Q9H5I1 O00746

P00533 O00750

P04626 O00750

P09619 O00750

P14373 O00750

P27986 O00750

P31947 O00750

P62993 O00750

Q12873 O00757

P25024 O00762

Q86Y01 O00762

Q96S59 O00762

P01106 O00763

Q00005 O00763

O95166 O00764

P60520 O00764

P63104 O00764

Q9GZQ8 O00764

Q9H492 O00764

O00716 O00767

P00519 O14490

P06241 O14490

P16333 O14490

P19174 O14490

P78352 O14490

O43889 O14494

Q6P587 O14494

P06756 O14495

P08648 O14495

P41182 O14495

A4PIV7 O14497

P41182 O14497

P51531 O14497

P51532 O14497

Q13573 O14497

Q15532 O14497

Q99459 O14497

O14503 O14503

P04637 O14503

P08670 O14503

P19784 O14503

P23142 O14503

P27482 O14503

P62136 O14503

P62140 O14503

P63279 O14503

P67775 O14503

P68400 O14503

Q00987 O14503

Q12873 O14503

Q14995 O14503

Q15047 O14503

Q15910 O14503

Q16537 O14503

Q16649 O14503

Q92753 O14503

Q96BI3 O14503

Q99743 O14503

Q9H0E2 O14503

Q9H469 O14503

Q9NPI1 O14503

Q9UKY1 O14503

P04054 O14508

P10912 O14508

P19235 O14508

P35226 O14508

Q15369 O14508

O60504 O14512

Q14141 O14512

Q8IZP0 O14512

P00519 O14513

P06241 O14513

P25054 O14513

O15372 O14514

O94812 O14514

O76071 O14519

P01876 O14519

P01877 O14519

P04745 O14519

P67870 O14519

Q13547 O14519

Q8TAQ5 O14519

Q92769 O14519

Q99643 O14521

O14522 O14522

Q16849 O14522

Q05516 O14526

O95163 O14530

P21246 O14530

Q12873 O14530

Q15047 O14530

Q16891 O14530

Q99689 O14530

Q9P2H0 O14530

Q9Y2X7 O14530

Q16555 O14531

Q16827 O14531

Q99961 O14531

Q99962 O14531

Q99963 O14531

O60674 O14543

P15822 O14543

P19235 O14543

P20936 O14543

P56159 O14543

Q13526 O14543

Q16659 O14543

Q66K74 O14543

Q6PKC3 O14543

Q96EP0 O14543

Q99665 O14543

Q9UGI8 O14543

Q9UP52 O14543

Q04206 O14544

Q4VCS5 O14544

Q96FK6 O14544

Q13418 O14545

Q8IZL9 O14545

Q00987 O14556

P61981 O14558

Q9UJY1 O14558

P06241 O14559

P63000 O14559

Q16540 O14561

Q9H0P0 O14561

Q13432 O14569

O43264 O14576

O43809 O14576

O95251 O14576

O95793 O14576

P04183 O14576

P09467 O14576

P18754 O14576

P23508 O14576

P38936 O14576

P47736 O14576

Q14203 O14576

Q14451 O14576

Q15796 O14576

Q8NFZ8 O14576

Q96G01 O14576

Q96NL0 O14576

Q9BQ89 O14576

Q9H4B7 O14576

Q9H4H8 O14576

P02751 O14579

P53621 O14579

Q99816 O14579

Q9NTJ5 O14579

O75865 O14582

Q7Z7N5 O14582

Q86SZ2 O14582

P39656 O14593

P52272 O14593

Q14203 O14593

Q16650 O14593

Q96EY7 O14593

P10275 O14595

Q66K74 O14599

O60841 O14602

P60953 O14613

P32121 O14617

P49407 O14617

P51809 O14617

P27487 O14625

Q6V1X1 O14625

P45973 O14628

O95751 O14639

P08670 O14639

P31947 O14639

P62993 O14639

Q04917 O14639

Q12772 O14639

Q12933 O14639

Q13137 O14639

Q8N6Y0 O14639

Q96ED9 O14639

Q9BUH8 O14639

Q9Y3Q8 O14639

P20073 O14640

P25054 O14640

P25788 O14640

P49674 O14640

Q14186 O14640

Q8IW93 O14640

Q96FS4 O14640

Q9Y4D1 O14640

Q5S007 O14640

O14640 O14641

O14641 O14641

O14978 O14641

O43395 O14641

O60504 O14641

O95817 O14641

P04637 O14641

P10074 O14641

P15622 O14641

P26368 O14641

P45974 O14641

P49674 O14641

P49910 O14641

P56545 O14641

P61457 O14641

P84022 O14641

Q12933 O14641

Q14134 O14641

Q15287 O14641

Q155Q3 O14641

Q5S007 O14641

Q7Z7J5 O14641

Q8IYF1 O14641

Q8N4T4 O14641

Q8N5R6 O14641

Q8N684 O14641

Q8TAD8 O14641

Q8TBE0 O14641

Q8WTU0 O14641

Q93062 O14641

Q96A72 O14641

Q96BN8 O14641

Q96BR9 O14641

Q96CG3 O14641

Q96EZ8 O14641

Q96H20 O14641

Q96MT3 O14641

Q96RL1 O14641

Q9BVN2 O14641

Q9BXS5 O14641

Q9H0I2 O14641

Q9NPB6 O14641

Q9NVV9 O14641

Q9NWB1 O14641

Q9NYF0 O14641

Q9UNA4 O14641

A0JLT2 O14645

O14645 O14645

Q96LA8 O14645

Q9NX70 O14645

P63165 O14647

Q8WV44 O14647

Q99497 O14647

P00533 O14654

P01106 O14654

P04626 O14654

P07900 O14654

P46108 O14654

P48039 O14654

P49286 O14654

P62993 O14654

Q00005 O14654

Q13418 O14654

Q13625 O14654

Q5U5Q3 O14654

Q9H492 O14654

P16104 O14656

Q14204 O14656

Q8NFQ8 O14656

Q96NW7 O14656

Q9BT78 O14656

Q9P003 O14656

Q9UBM7 O14656

P16104 O14657

Q05209 O14662

P21926 O14672

P30101 O14672

P12956 O14678

Q15121 O14678

O15550 O14686

P03372 O14686

P61964 O14686

Q13573 O14686

Q14686 O14686

Q15291 O14686

Q15797 O14686

Q6ZW49 O14686

Q9UBL3 O14686

Q9GZM8 O14709

Q9NRI5 O14709

P46939 O14713

P68104 O14713

P22392 O14713

P01616 O14717

P09661 O14717

P58107 O14717

P62318 O14717

P68104 O14717

Q15047 O14717

Q16623 O14717

Q5RL73 O14717

Q5UIP0 O14717

Q6P4R8 O14717

Q7L5N1 O14717

Q9BVJ6 O14717

Q9UPU7 O14717

P10276 O14727

P55211 O14727

P99999 O14727

Q9NQS1 O14727

O43521 O14730

O75386 O14730

P20618 O14730

P22087 O14730

P40616 O14730

P43360 O14730

P43686 O14730

P51571 O14730

P62158 O14730

Q13177 O14730

Q68CJ9 O14730

Q92851 O14730

Q9Y230 O14730

Q92956 O14732

O15519 O14733

O43318 O14733

O60711 O14733

P08238 O14733

P62888 O14733

Q13573 O14733

Q5S007 O14733

Q86Y07 O14733

Q96RU7 O14733

Q99459 O14733

Q99683 O14733

Q9UQF2 O14733

P45983 O14733

P01106 O14734

P05386 O14735

Q13418 O14735

Q15051 O14735

Q86UT6 O14735

Q8NE63 O14735

Q9HCK5 O14735

Q9NR20 O14735

P02751 O14737

P26641 O14737

Q15170 O14737

Q5UIP0 O14737

Q92993 O14737

Q9BVJ6 O14737

Q9P2H0 O14737

O00267 O14744

O14744 O14744

O43852 O14744

O75044 O14744

O95391 O14744

O95863 O14744

P02751 O14744

P04908 O14744

P08621 O14744

P09017 O14744

P19320 O14744

P32121 O14744

P45973 O14744

P49407 O14744

P49674 O14744

P54105 O14744

P62805 O14744

P62993 O14744

P85299 O14744

Q01094 O14744

Q14524 O14744

Q15910 O14744

Q5HYJ3 O14744

Q86U06 O14744

Q8IXH7 O14744

Q8N2W9 O14744

Q8N8U2 O14744

Q8NAV1 O14744

Q8TE85 O14744

Q96AP4 O14744

Q96EZ8 O14744

Q96PV6 O14744

Q9BQA1 O14744

Q9BSG1 O14744

Q9BX10 O14744

Q9C0F3 O14744

Q9H0R8 O14744

Q9H9G7 O14744

Q9HCK5 O14744

Q9NQ92 O14744

Q9NTK5 O14744

Q9UKV8 O14744

Q9UL18 O14744

Q9Y5B0 O14744

P07550 O14745

P07947 O14745

P08473 O14745

P09619 O14745

P13569 O14745

P15311 O14745

P23508 O14745

P26038 O14745

P35240 O14745

P41145 O14745

P41231 O14745

P46937 O14745

P47900 O14745

P60484 O14745

Q01970 O14745

Q6ZVD8 O14745

Q9BXI6 O14745

Q9NWQ8 O14745

Q9ULV1 O14745

Q9Y6Q1 O14745

O60832 O14746

P29590 O14746

Q86US8 O14746

Q92900 O14746

Q9BUR4 O14746

Q9Y230 O14746

Q9Y265 O14746

O00255 O14757

P08238 O14757

P15927 O14757

P31946 O14757

P38398 O14757

P61981 O14757

P67870 O14757

Q01130 O14757

Q06609 O14757

Q13501 O14757

Q13573 O14757

Q13616 O14757

Q8IY92 O14757

Q8TD19 O14757

Q99459 O14757

Q9HAW4 O14757

Q9UJM3 O14757

O00220 O14763

P50591 O14763

Q14790 O14763

Q96P48 O14763

Q9NRR5 O14764

Q9UMX0 O14764

P31314 O14770

P55347 O14770

P01023 O14773

P01106 O14773

O60238 O14775

O75822 O14775

P27694 O14775

P38936 O14775

P49736 O14775

P53602 O14775

P67870 O14775

Q14209 O14775

Q15406 O14775

Q15714 O14775

O00716 O14776

O75554 O14776

P19320 O14776

P42858 O14776

Q8N2W9 O14776

Q9NQ29 O14776

Q9NZC7 O14776

Q9Y2W1 O14776

O14777 O14777

O43264 O14777

O43683 O14777

O75674 O14777

O95229 O14777

P30622 O14777

Q13573 O14777

Q53G59 O14777

Q8N6Y0 O14777

Q99459 O14777

Q9BZD4 O14777

Q9H081 O14777

Q9H3R5 O14777

Q9HBM1 O14777

Q9P1Z2 O14777

Q9UL45 O14777

Q9Y2J4 O14777

Q9Y3C0 O14777

Q99459 O14782

Q9NRI5 O14782

P15692 O14786

P17948 O14786

P19784 O14786

P21333 O14786

O00329 O14787

O95758 O14787

O95988 O14787

Q8TBF2 O14788

P01871 O14791

O14793 O14793

P04626 O14796

Q06830 O14796

Q9UIB8 O14796

P02647 O14798

P78362 O14798

Q8N5W9 O14798

Q9UKY1 O14798

P01106 O14802

P24386 O14802

Q15051 O14810

O14818 O14818

P01106 O14818

P17861 O14818

P19320 O14818

P25786 O14818

P25787 O14818

P25788 O14818

P25789 O14818

P28066 O14818

P31152 O14818

P49720 O14818

P55036 O14818

P55072 O14818

P60900 O14818

Q15051 O14818

Q16659 O14818

Q16665 O14818

Q92530 O14818

Q96SB4 O14818

Q9BQ83 O14818

Q9HC96 O14818

Q9NWV8 O14818

Q9Y244 O14818

Q9Y5K5 O14818

P10276 O14830

O75674 O14832

P28288 O14832

P49190 O14832

Q9H6Y7 O14832

O75888 O14836

P49069 O14836

Q99836 O14836

Q9Y275 O14836

O14862 O14862

P11279 O14862

Q9ULZ3 O14862

O95758 O14867

P48729 O14867

Q9UBE8 O14867

Q9UJX4 O14867

O00442 O14874

P12694 O14874

P40763 O14874

Q52LJ0 O14874

Q9H9S4 O14874

Q9Y376 O14874

O14879 O14879

P02649 O14879

P06753 O14879

P09913 O14879

P09914 O14879

P54725 O14879

Q05D60 O14879

Q13325 O14879

Q15051 O14893

Q16637 O14893

Q96DB2 O14893

Q9UHI6 O14893

Q9UL18 O14893

Q02556 O14896

O00444 O14907

O00159 O14908

O95758 O14908

P17252 O14908

P17643 O14908

P33993 O14908

P35606 O14908

P51571 O14908

P56192 O14908

P57678 O14908

Q01995 O14908

Q13641 O14908

Q6P996 O14908

Q8N1F7 O14908

Q96T76 O14908

Q9P0V3 O14908

Q9UM54 O14908

Q9Y3T9 O14908

P60520 O14910

Q4VCS5 O14910

O14920 O14920

O15111 O14920

O43318 O14920

P04350 O14920

P07437 O14920

P07814 O14920

P07900 O14920

P08238 O14920

P09874 O14920

P11021 O14920

P11142 O14920

P12931 O14920

P21333 O14920

P23396 O14920

P25963 O14920

P34931 O14920

P35580 O14920

P38646 O14920

P46940 O14920

P61626 O14920

P62158 O14920

P63261 O14920

P67775 O14920

Q00610 O14920

Q00839 O14920

Q04206 O14920

Q07021 O14920

Q13748 O14920

Q14145 O14920

Q16543 O14920

Q3ZCQ8 O14920

Q5D1E8 O14920

Q7Z434 O14920

Q86WI3 O14920

Q8N163 O14920

Q92574 O14920

Q92793 O14920

Q92922 O14920

Q99558 O14920

Q9BVA1 O14920

Q9UGK3 O14920

Q9ULZ3 O14920

Q9UQC1 O14920

Q9Y6K9 O14920

Q9Y6Q9 O14920

Q3ZCQ8 O14925

Q9BVV7 O14925

Q9NQG6 O14925

Q9NWV8 O14925

O95166 O14929

P16104 O14929

P49459 O14929

P62805 O14929

P63104 O14929

P68431 O14929

Q13283 O14929

Q92560 O14929

Q9H0Y0 O14929

O43164 O14933

O60260 O14933

O75369 O14933

O95376 O14933

P43490 O14933

P50876 O14933

P52565 O14933

Q13233 O14933

Q6ZMZ0 O14933

Q7Z419 O14933

Q9BZR9 O14933

O00472 O14936

O14910 O14936

O60238 O14936

P11171 O14936

P34741 O14936

P41134 O14936

P51452 O14936

P55198 O14936

P78362 O14936

Q12929 O14936

Q14155 O14936

Q16650 O14936

Q8IYM9 O14936

Q96RL1 O14936

Q9C0A0 O14936

Q9NYY3 O14936

Q9UHC6 O14936

Q9UM82 O14936

Q9Y2J0 O14936

P13569 O14939

P23528 O14939

P62993 O14939

P08574 O14949

P31930 O14949

Q96CV9 O14949

P02751 O14950

P19320 O14950

P35749 O14950

P49407 O14950

P62736 O14950

P62993 O14950

Q00403 O14950

Q14457 O14950

Q70EL2 O14950

P08574 O14957

O14958 O14958

O14960 O14960

A9UHW6 O14964

O43513 O14964

O75674 O14964

O75886 O14964

O95751 O14964

P0CG48 O14964

P62993 O14964

Q15038 O14964

Q53EZ4 O14964

Q5VSY0 O14964

Q8IYI6 O14964

Q8N5R6 O14964

Q8N6Y0 O14964

Q96B67 O14964

Q96KQ7 O14964

Q96LR2 O14964

Q99816 O14964

Q9BUH8 O14964

Q9NZ52 O14964

Q9UJY4 O14964

Q9UMX0 O14964

Q9UPT5 O14964

A0AUL9 O14965

O00743 O14965

O14965 O14965

O15350 O14965

P04179 O14965

P04198 O14965

P42771 O14965

P61026 O14965

P61978 O14965

P68036 O14965

Q01469 O14965

Q12834 O14965

Q13573 O14965

Q96SB4 O14965

P51811 O14967

Q9NR96 O14967

Q9NZC3 O14967

P10644 O14974

P32121 O14974

P33993 O14974

P62993 O14974

P63104 O14974

Q09013 O14974

Q6WCQ1 O14974

Q92905 O14974

P63104 O14975

O60763 O14976

O95967 O14978

P57086 O14978

Q8WV44 O14978

Q96AF5 O14978

Q9H8W4 O14978

Q9NZD8 O14978

Q9UMX1 O14978

Q9Y5B8 O14978

B2Y833 O14979

O14879 O14979

O95166 O14979

P02751 O14979

P09913 O14979

P11021 O14979

P16104 O14979

P17096 O14979

P19320 O14979

P60520 O14979

Q14197 O14979

Q9GZQ8 O14979

Q9H0R8 O14979

Q9H492 O14979

P68400 O14979

O00165 O14980

O15392 O14980

O43566 O14980

O95758 O14980

P01106 O14980

P02751 O14980

P04637 O14980

P08238 O14980

P13569 O14980

P18754 O14980

P19320 O14980

P30304 O14980

P35232 O14980

P38646 O14980

P61925 O14980

P80303 O14980

Q02818 O14980

Q09161 O14980

Q13286 O14980

Q14257 O14980

Q96RS0 O14980

Q9NPQ8 O14980

Q13573 O14981

Q99459 O14981

Q9H6Y7 O14983

Q9UKG1 O14983

O15066 O15013

Q8TAF3 O15013

Q13547 O15014

Q99750 O15015

Q9Y2I6 O15015

P01106 O15018

P25054 O15020

Q92731 O15020

P01106 O15021

Q15797 O15021

O95758 O15027

P01375 O15027

P29353 O15027

P48729 O15027

P49674 O15027

Q15051 O15027

Q96DB2 O15027

Q9BY41 O15027

Q9UBN7 O15027

O60925 O15031

P08581 O15031

P21246 O15031

Q04912 O15031

Q71F23 O15031

Q9H3S1 O15031

P08238 O15033

O00555 O15034

Q9Y6I4 O15034

O95166 O15040

P60520 O15040

Q9BXW4 O15040

Q9H0R8 O15040

A0JLT2 O15042

O95166 O15042

P01106 O15042

P03372 O15042

P52756 O15042

Q13573 O15042

Q86XP3 O15042

Q99459 O15042

Q9H0R8 O15042

Q9NWA0 O15042

Q9NX70 O15042

P10242 O15047

P51610 O15047

P61964 O15047

Q9Y2K6 O15049

Q14781 O15050

P03372 O15054

P67870 O15054

P00519 O15056

P06241 O15056

P31947 O15056

Q9NZM3 O15056

Q9UGN5 O15056

Q15834 O15062

O15013 O15066

P25791 O15066

P61968 O15066

O75323 O15067

Q14145 O15067

Q96RL1 O15067

Q9UGJ0 O15067

Q9NRH1 O15068

Q9UI14 O15068

Q9UL18 O15068

O43303 O15078

Q15051 O15078

Q8IW35 O15078

Q8TAP6 O15078

Q96RK4 O15078

P10242 O15083

Q15051 O15083

O00743 O15084

O15084 O15084

P19338 O15084

P53350 O15084

Q01484 O15084

Q14185 O15084

Q5H9R7 O15084

Q70CQ1 O15084

Q8WZ42 O15084

Q96CW1 O15084

Q96RS0 O15084

Q9H2K2 O15084

Q9UG63 O15084

Q9UK80 O15084

Q9ULJ7 O15084

Q9UM47 O15084

Q9UPN7 O15084

Q9UPS8 O15084

O43157 O15085

O95477 O15085

P00519 O15085

P16333 O15085

P27986 O15085

P32121 O15085

P46108 O15085

P60953 O15085

P61586 O15085

P62993 O15085

Q92905 O15085

Q9Y4D1 O15085

Q14197 O15091

Q7L0Y3 O15091

Q99714 O15091

O00308 O15105

O15169 O15105

O75925 O15105

O95625 O15105

P12956 O15105

P36896 O15105

P62942 O15105

Q15361 O15105

Q8N2W9 O15105

Q92905 O15105

Q9BT81 O15105

Q9HAU4 O15105

Q9NQ87 O15105

Q9UII5 O15105

Q9ULH7 O15105

Q9Y2K7 O15105

O14920 O15111

O15111 O15111

O94832 O15111

P01106 O15111

P04350 O15111

P06753 O15111

P07437 O15111

P07900 O15111

P08238 O15111

P12931 O15111

P19105 O15111

P25963 O15111

P32121 O15111

P35579 O15111

P35580 O15111

P38646 O15111

P46527 O15111

P52907 O15111

P60660 O15111

P62158 O15111

P63261 O15111

P67775 O15111

P78362 O15111

Q00610 O15111

Q04206 O15111

Q05639 O15111

Q13451 O15111

Q13748 O15111

Q15653 O15111

Q16543 O15111

Q7Z406 O15111

Q7Z434 O15111

Q86WI3 O15111

Q92793 O15111

Q99558 O15111

Q99759 O15111

Q9UGK3 O15111

Q9ULZ3 O15111

Q9UQC1 O15111

Q9Y6K9 O15111

Q9Y6Q9 O15111

O43776 O15116

P07900 O15116

P28062 O15116

P28074 O15116

P41182 O15116

P62310 O15116

Q14684 O15116

Q6NUQ4 O15116

Q96B26 O15116

Q96C01 O15116

Q9H270 O15116

Q9UBK9 O15116

Q9UBQ7 O15116

Q9Y333 O15116

Q9Y4Z0 O15116

O60880 O15117

P00519 O15117

P48023 O15117

P62993 O15117

Q13094 O15117

Q86WV1 O15117

Q86WV6 O15118

O15084 O15120

P49915 O15120

Q9P1Y6 O15120

Q9UGH3 O15120

O15260 O15121

O43809 O15121

P11217 O15121

P22061 O15121

P34897 O15121

P53985 O15121

P60228 O15121

Q15393 O15121

Q53HC9 O15121

Q8IVH2 O15121

Q96JH8 O15121

Q9H8S9 O15121

P15692 O15123

P20073 O15123

P38936 O15123

P67870 O15123

Q02763 O15123

Q15051 O15123

P04637 O15126

Q05086 O15126

P63104 O15127

P46527 O15131

Q13309 O15131

P42768 O15143

P78362 O15143

P18206 O15144

P62993 O15144

Q8WYL5 O15144

Q92731 O15144

Q9BR76 O15144

P02751 O15145

P09960 O15145

P11597 O15145

P19320 O15145

P42768 O15145

P42772 O15145

P46379 O15145

P62993 O15145

Q86YJ6 O15145

Q96G74 O15145

Q96P48 O15145

Q99873 O15145

O00505 O15151

O14757 O15151

P04637 O15151

P06400 O15151

P08238 O15151

P10415 O15151

P27348 O15151

P31946 O15151

P48729 O15151

P51948 O15151

P61981 O15151

P62258 O15151

Q00987 O15151

Q04917 O15151

Q3YBA8 O15151

Q6UWE0 O15151

Q7Z4K8 O15151

Q93009 O15151

Q9UER7 O15151

O95758 O15155

Q8N6Y0 O15155

P52292 O15156

Q96G21 O15156

O95602 O15160

P02751 O15160

P10909 O15160

P32121 O15160

P49407 O15160

P51784 O15160

Q13573 O15160

Q8TBZ3 O15160

Q99459 O15160

Q9H2D6 O15160

Q9H5J8 O15160

Q9UL18 O15160

Q9Y2S0 O15160

O43597 O15162

O60269 O15162

O76003 O15162

O94817 O15162

P04196 O15162

P12236 O15162

P17509 O15162

P23142 O15162

P28749 O15162

P29590 O15162

P46109 O15162

P50552 O15162

P78381 O15162

Q00587 O15162

Q05516 O15162

Q14764 O15162

Q14966 O15162

Q15038 O15162

Q15466 O15162

Q15637 O15162

Q16526 O15162

Q16625 O15162

Q16630 O15162

Q16659 O15162

Q5TA45 O15162

Q6UY11 O15162

Q6UY14 O15162

Q86UW9 O15162

Q8N5R6 O15162

Q8TC90 O15162

Q8WV24 O15162

Q8WWR8 O15162

Q92734 O15162

Q92837 O15162

Q96CW7 O15162

Q96D16 O15162

Q96H86 O15162

Q96LJ7 O15162

Q96RN5 O15162

Q9BQ66 O15162

Q9BTL3 O15162

Q9BVI4 O15162

Q9GZM5 O15162

Q9H0B3 O15162

Q9H0I2 O15162

Q9H1Q7 O15162

Q9H7M9 O15162

Q9HB63 O15162

Q9NQX5 O15162

Q9NRQ2 O15162

Q9NRY6 O15162

Q9NTK1 O15162

Q9NVZ3 O15162

Q9NX53 O15162

Q9P0T4 O15162

Q9UBP5 O15162

Q9UHH9 O15162

Q9UQ90 O15162

Q9Y272 O15162

P03372 O15164

Q8WVD5 O15164

Q92731 O15164

Q9BZR9 O15164

O14641 O15169

O14976 O15169

O15105 O15169

O15169 O15169

O95758 O15169

P01106 O15169

P04637 O15169

P25791 O15169

P29590 O15169

P35222 O15169

P35813 O15169

P39687 O15169

P49840 O15169

P49841 O15169

P62136 O15169

P68104 O15169

Q14134 O15169

Q14194 O15169

Q8N4C6 O15169

Q99961 O15169

Q9BVJ6 O15169

Q9H2K2 O15169

Q9NYF0 O15169

Q9UBF9 O15169

Q9Y4X0 O15169

O43924 O15182

O95229 O15182

Q13432 O15182

Q5UIP0 O15182

Q6UVJ0 O15182

Q70CQ1 O15182

P41182 O15197

P55196 O15197

A4FU01 O15198

O00625 O15198

O14686 O15198

O15550 O15198

O75197 O15198

O94907 O15198

O95782 O15198

P01243 O15198

P05108 O15198

P26572 O15198

P27540 O15198

P47897 O15198

P48556 O15198

P51825 O15198

P61968 O15198

Q01082 O15198

Q03181 O15198

Q14669 O15198

Q16537 O15198

Q4LDE5 O15198

Q5XPI4 O15198

Q6PD62 O15198

Q6PGP7 O15198

Q86UK5 O15198

Q8IZ52 O15198

Q8N3P4 O15198

Q8N5G2 O15198

Q8N988 O15198

Q8NAM6 O15198

Q8NEV8 O15198

Q8NFD5 O15198

Q92967 O15198

Q969K4 O15198

Q96AD5 O15198

Q96FW1 O15198

Q96LA8 O15198

Q96MK7 O15198

Q99613 O15198

Q9GZM7 O15198

Q9H078 O15198

Q9H3U1 O15198

Q9H6V3 O15198

Q9NR34 O15198

Q9NRL2 O15198

Q9NSV4 O15198

Q9UBL3 O15198

Q9UJ70 O15198

Q9Y2U8 O15198

Q9Y5J3 O15198

Q96SB4 O15205

O00189 O15209

Q9NRR5 O15209

O95251 O15211

P01112 O15211

P24385 O15211

P26641 O15211

Q13432 O15211

Q14194 O15211

Q92993 O15211

Q9GZT6 O15211

O43172 O15212

P62714 O15212

Q00987 O15212

Q13164 O15212

Q13547 O15212

Q6PEY2 O15212

Q9UM82 O15212

O15217 O15217

O75569 O15226

O95166 O15226

P30519 O15226

P41182 O15226

Q92731 O15226

Q96C10 O15226

O75381 O15228

P46089 O15228

Q8IY92 O15228

P46109 O15230

Q6VMQ6 O15230

P33993 O15231

P49747 O15232

O95793 O15234

P38919 O15234

P61326 O15234

P62993 O15234

Q00534 O15234

Q9BZI7 O15234

Q9H1J1 O15234

Q9Y2W1 O15234

Q9Y5S9 O15234

P58062 O15235

Q13432 O15235

Q14194 O15235

Q5UIP0 O15235

Q9UKR5 O15235

Q9HAU4 O15239

P41161 O15244

Q9H4G1 O15244

Q9H9G7 O15244

Q9Y4Z0 O15244

Q96G23 O15245

P02751 O15247

Q9HAU4 O15254

P00519 O15255

Q9UMX0 O15255

O95758 O15258

P54257 O15258

Q16891 O15258

Q99963 O15258

P98161 O15259

Q13444 O15259

Q9NRR5 O15259

P49257 O15260

P49755 O15260

Q13530 O15260

Q15363 O15260

Q86WV6 O15260

P15336 O15264

P21462 O15264

P36969 O15264

Q15139 O15264

Q86XL3 O15264

O00468 O15265

O43707 O15265

O75095 O15265

P02751 O15265

P12814 O15265

P14373 O15265

P36406 O15265

P37198 O15265

P54725 O15265

P55268 O15265

Q08379 O15265

Q12805 O15265

Q13625 O15265

Q15276 O15265

Q5JY77 O15265

Q6PKC3 O15265

Q6UWE0 O15265

Q6WCQ1 O15265

Q7Z7M0 O15265

Q8N2S1 O15265

Q8NHQ1 O15265

Q96EP0 O15265

Q9BX66 O15265

Q9BZL4 O15265

Q9NRD5 O15265

Q9P209 O15265

Q9UHD9 O15265

Q9Y219 O15265

Q9Y2V7 O15265

Q9Y3T9 O15265

Q9Y4A5 O15265

O15266 O15266

P35712 O15266

P01106 O15269

P13569 O15269

Q96DT6 O15269

Q9P2Y5 O15269

A0AUL9 O15270

P41182 O15270

O43504 O15273

O60662 O15273

P06241 O15273

P06733 O15273

P11142 O15273

P23759 O15273

P54253 O15273

P98160 O15273

Q04206 O15273

Q12988 O15273

Q8WUM0 O15273

Q8WZ42 O15273

Q9NPC6 O15273

Q9UHD1 O15273

O15287 O15287

O15360 O15287

O96006 O15287

P08727 O15287

Q06547 O15287

Q13813 O15287

Q16658 O15287

Q8N6K7 O15287

Q8N6Y0 O15287

Q8NB91 O15287

Q9BXW9 O15287

Q9HB96 O15287

Q9NPI8 O15287

O95758 O15294

P37198 O15294

P51610 O15294

P63165 O15294

P98177 O15294

Q04206 O15294

Q8IZD2 O15294

Q92560 O15294

Q9H1M0 O15294

Q9UKB1 O15294

Q9Y297 O15294

Q00005 O15294

P16104 O15297

Q00987 O15297

Q99459 O15297

P26842 O15304

Q07817 O15304

Q9NR77 O15304

P62805 O15315

Q6NVH7 O15315

P00519 O15320

P02768 O15320

P62993 O15320

O60930 O15321

P08473 O15321

P38606 O15321

B3KSH4 O15344

O15344 O15344

P51668 O15344

P51965 O15344

P61077 O15344

P62837 O15344

P68036 O15344

Q02556 O15344

Q13404 O15344

Q969T4 O15344

Q96B02 O15344

Q96LR5 O15344

Q9Y2X8 O15344

P17096 O15347

O14965 O15350

P00519 O15350

P38646 O15350

Q03701 O15350

Q12834 O15350

Q96EB6 O15350

Q9NZC7 O15350

P46937 O15350

Q96J02 O15350

Q9UKL3 O15350

P43356 O15350

P0CG48 O15350

P46934 O15350

P04637 O15355

P49286 O15355

Q13568 O15355

Q15418 O15355

Q6PIJ6 O15355

Q70CQ1 O15355

Q8IY92 O15355

Q92560 O15355

Q93009 O15355

Q9P275 O15355

Q9Y6K5 O15355

A9UF07 O15357

O00459 O15357

O60504 O15357

P01730 O15357

P19320 O15357

P22681 O15357

P46108 O15357

P62993 O15357

Q15811 O15357

Q8IV36 O15357

Q8IZP0 O15357

Q8TF42 O15357

Q9BX66 O15357

O15287 O15360

O15360 O15360

P06241 O15360

P12931 O15360

P16333 O15360

P19174 O15360

P38398 O15360

P51532 O15360

Q00597 O15360

Q0VG06 O15360

Q13472 O15360

Q13813 O15360

Q8NB91 O15360

Q9HB96 O15360

Q9NPI8 O15360

P19623 O15370

P49005 O15370

O00232 O15371

O00571 O15371

O15372 O15371

P12931 O15371

P14921 O15371

P19320 O15371

P30480 O15371

P55884 O15371

P60228 O15371

P60709 O15371

P62993 O15371

Q14240 O15371

Q15796 O15371

Q8WV24 O15371

Q9Y6I4 O15371

O00571 O15372

O15372 O15372

P08238 O15372

P23508 O15372

P55884 O15372

Q13573 O15372

Q14240 O15372

Q15834 O15372

Q8TAF3 O15372

Q9NRI5 O15372

Q9P2A4 O15372

Q9Y6I4 O15372

O60341 O15379

O60566 O15379

O75376 O15379

P01106 O15379

P43356 O15379

P48552 O15379

P56524 O15379

P60510 O15379

Q06330 O15379

Q13526 O15379

Q15022 O15379

Q15156 O15379

Q15910 O15379

Q16576 O15379

Q6MZP7 O15379

Q7L2E3 O15379

Q8WUI4 O15379

Q92766 O15379

Q92786 O15379

Q969R5 O15379

Q96EK4 O15379

Q9BZK7 O15379

Q9UIS9 O15379

Q9UKG1 O15379

Q9UKV0 O15379

Q9UQL6 O15379

Q9UQR1 O15379

Q9Y5X4 O15379

Q9Y618 O15379

P63279 O15381

Q14194 O15381

Q6T424 O15381

P04075 O15382

P41222 O15382

Q15645 O15389

O14980 O15392

O15392 O15392

P06493 O15392

P55211 O15392

Q14457 O15392

Q53HL2 O15392

Q96GD4 O15392

Q9NQS7 O15392

Q9UQB9 O15392

Q9P0W5 O15394

P13984 O15397

P45973 O15397

Q9H9G7 O15397

Q9HCK5 O15397

Q9UKV8 O15397

Q9UL18 O15397

O43889 O15400

O75379 O15400

P46459 O15400

P51809 O15400

Q15836 O15400

Q16659 O15400

Q9BV40 O15400

P08238 O15409

Q13547 O15417

O00716 O15427

P40692 O15438

P03372 O15440

P20073 O15442

Q9NRI5 O15442

O95602 O15446

P23025 O15446

P49407 O15446

Q08945 O15446

Q9UKV8 O15451

P12931 O15455

Q7L0X0 O15455

O43196 O15457

O43889 O15460

P16104 O15460

Q09019 O15460

O14744 O15479

P10276 O15479

P35268 O15479

P35637 O15479

P37108 O15479

P39019 O15479

P39023 O15479

P42766 O15479

P46779 O15479

P46781 O15479

P47914 O15479

P49207 O15479

P52272 O15479

P54253 O15479

P61247 O15479

P61254 O15479

P62249 O15479

P62263 O15479

P62280 O15479

P62750 O15479

P62807 O15479

P62854 O15479

P62899 O15479

P62910 O15479

P67870 O15479

P83731 O15479

Q02543 O15479

Q13257 O15479

Q13263 O15479

Q93009 O15479

Q9BS40 O15479

Q9H0J4 O15479

Q9H7X7 O15479

Q9UJX6 O15479

Q9UQ35 O15479

Q9Y3U8 O15479

Q8IY81 O15481

Q12929 O15482

P40692 O15484

Q70SY1 O15492

Q9Y5Y6 O15492

Q9HD67 O15496

O95758 O15498

P24386 O15504

P25054 O15504

Q9H0R8 O15504

O15372 O15511

P49407 O15511

P62993 O15511

A0JLT2 O15514

O95402 O15514

Q9BTT4 O15514

Q9NWA0 O15514

Q9NX70 O15514

O00327 O15516

O00220 O15519

O00303 O15519

O60496 O15519

O75533 O15519

P04049 O15519

P14174 O15519

P14866 O15519

P22087 O15519

P25445 O15519

P49411 O15519

P50591 O15519

P58107 O15519

P62273 O15519

Q14790 O15519

Q15393 O15519

Q16543 O15519

Q16658 O15519

Q92851 O15519

Q96JH8 O15519

Q9UH99 O15519

Q9Y4E8 O15519

O14733 O15519

P45985 O15519

Q02750 O15519

P21802 O15520

P03372 O15523

P04626 O15524

Q04206 O15524

Q13617 O15524

Q13748 O15524

Q15369 O15524

Q8N668 O15524

Q99683 O15524

P08238 O15525

P08670 O15525

Q16236 O15525

Q5RL73 O15525

Q9Y3C7 O15525

P05060 O15527

P62306 O15527

O00141 O15530

O14543 O15530

O15273 O15530

O15530 O15530

O95248 O15530

P01857 O15530

P08575 O15530

P23467 O15530

P27348 O15530

P31749 O15530

P42345 O15530

P52736 O15530

Q00005 O15530

Q12824 O15530

Q13867 O15530

Q15262 O15530

Q16827 O15530

Q86WB0 O15530

Q8IWY9 O15530

Q8TBF2 O15530

Q96SB4 O15530

Q9BXL7 O15530

Q9H6R7 O15530

Q9H814 O15530

Q03518 O15533

Q03519 O15533

P49674 O15534

Q9Y4F9 O15534

O43463 O15535

Q96AF5 O15535

Q14498 O15541

Q5VVX9 O15541

Q96SB4 O15547

O14686 O15550

Q15291 O15550

Q6ZW49 O15550

Q9UBL3 O15550

Q9UIU6 O15550

Q6BCY4 O15552

P62158 O15554

O75923 O43143

O95166 O43143

P01106 O43143

P02751 O43143

P03372 O43143

P19320 O43143

P32121 O43143

P38919 O43143

P51858 O43143

P52756 O43143

P60520 O43143

P61326 O43143

P78362 O43143

Q13573 O43143

Q13595 O43143

Q86XP3 O43143

Q8IWZ8 O43143

Q8N5A5 O43143

Q8NCN4 O43143

Q96BK5 O43143

Q96I25 O43143

Q9H0R8 O43143

Q9H492 O43143

Q9UKV8 O43143

Q9UL18 O43143

Q9UPE1 O43143

O43570 O43147

O75525 O43147

P02771 O43147

P21246 O43147

P62851 O43147

Q00013 O43147

Q12873 O43147

Q12905 O43147

Q5T0N5 O43147

Q96B86 O43147

Q9HCC8 O43147

Q9NRX1 O43147

Q9NYQ7 O43147

O95166 O43148

P01106 O43149

P12757 O43149

P0CG48 O43150

P12931 O43150

P19174 O43150

P46108 O43150

P62993 O43150

Q13191 O43150

Q15811 O43150

Q96B97 O43150

O43155 O43155

P19388 O43156

Q13315 O43156

Q6NXR4 O43156

Q96Q15 O43156

Q9Y265 O43156

Q9Y4R8 O43156

O14807 O43157

P61587 O43157

Q53GS9 O43159

Q96EB6 O43159

O94888 O43164

P51668 O43164

P61077 O43164

P61088 O43164

P62068 O43164

Q04323 O43164

Q96B02 O43164

Q96CS3 O43164

Q9UNN5 O43164

Q9Y2X8 O43164

P31947 O43166

P33993 O43166

P51784 O43166

Q13627 O43166

Q9Y463 O43166

Q99750 O43167

P30480 O43169

O75554 O43172

P02751 O43172

Q01081 O43172

Q13107 O43172

Q13523 O43172

Q15020 O43172

Q53GS9 O43172

Q92917 O43172

Q9Y4E8 O43172

O15264 O43175

P02751 O43175

P19320 O43175

P60520 O43175

P62745 O43175

P62993 O43175

Q13573 O43175

Q8TDR0 O43175

Q99459 O43175

Q9GZQ8 O43175

Q9H0R8 O43175

Q9H492 O43175

O43678 O43181

P60604 O43181

Q5HYK7 O43184

O95633 O43184

Q8TC57 O43186

Q99750 O43186

O43187 O43187

P08238 O43187

P08473 O43187

P10398 O43187

P46977 O43187

P51617 O43187

P58753 O43187

Q09028 O43187

Q13148 O43187

Q99836 O43187

Q9H0E2 O43187

Q9NWZ3 O43187

Q9Y4K3 O43187

Q9Y5A9 O43187

Q9Y616 O43187

O75923 O43189

Q13620 O43189

Q15910 O43189

Q16531 O43189

Q93062 O43189

Q9NVV9 O43189

O15457 O43196

P00519 O43196

Q13573 O43209

Q99459 O43209

Q6ZU15 O43236

Q8IUQ4 O43236

P98170 O43236

Q13573 O43237

Q99459 O43237

Q5UIP0 O43240

O00487 O43242

P01106 O43242

P19320 O43242

P51665 O43242

P54578 O43242

P62195 O43242

Q13573 O43242

Q16401 O43242

Q99459 O43242

Q9NWV8 O43242

Q9UKV8 O43242

Q9Y5K5 O43242

P42858 O43248

Q9Y5K6 O43248

O43251 O43251

O95758 O43251

P01106 O43251

P03372 O43251

P52597 O43251

P54253 O43251

P61978 O43251

Q07666 O43251

Q86U06 O43251

Q92530 O43251

Q96PU8 O43251

Q9BQY4 O43251

Q9BZE0 O43251

Q9H0E2 O43251

Q9NW64 O43251

P68104 O43252

Q13432 O43252

O75376 O43255

P10275 O43255

P29692 O43255

P35372 O43255

P41145 O43255

Q7Z6J0 O43255

Q99836 O43255

Q9H422 O43255

O96019 O43257

P0C0S5 O43257

P0CG48 O43257

Q12888 O43257

Q15759 O43257

Q15906 O43257

Q16539 O43257

Q9Y230 O43257

Q9Y265 O43257

O14678 O43261

O60383 O43261

P04637 O43261

P25786 O43261

P68104 O43261

Q04724 O43261

Q13885 O43261

Q15047 O43261

Q7L5N1 O43261

Q8IWV7 O43261

Q92993 O43261

Q9BVJ6 O43261

Q9GZT6 O43261

Q9NUQ8 O43261

Q9NVJ2 O43261

Q9P2H0 O43261

Q9Y3C7 O43261

O14777 O43264

O60566 O43264

O95229 O43264

P02751 O43264

Q04756 O43278

Q5TF85 O43278

Q9NRR5 O43278

O75865 O43281

P00519 O43281

P12931 O43281

P16333 O43281

P19174 O43281

O43283 O43283

P40763 O43283

Q04760 O43283

O43172 O43290

O75554 O43290

P24386 O43290

P49756 O43290

P55081 O43290

P55854 O43290

P63165 O43290

Q08170 O43290

Q13523 O43290

Q13573 O43290

Q15287 O43290

Q53GS9 O43290

Q8IWX8 O43290

Q8IY92 O43290

Q8TBK6 O43290

Q96EB6 O43290

Q99459 O43290

Q9BZL1 O43290

P60228 O43292

Q13526 O43292

Q16478 O43292

Q9NZ81 O43292

O43293 O43293

P08238 O43293

P62993 O43293

Q96IZ0 O43293

Q9UER7 O43293

O14796 O43294

P13612 O43294

P42772 O43294

Q05397 O43294

Q8IVE3 O43294

Q9H422 O43294

O43463 O43295

O60341 O43295

P48023 O43295

P51532 O43295

Q9UKE5 O43295

P45973 O43296

Q7L590 O43298

Q8IYF1 O43298

Q8TAP4 O43298

Q8TAU3 O43298

Q8TBB1 O43298

P14316 O43299

Q13573 O43301

Q92823 O43301

Q99459 O43301

O15078 O43303

P25189 O43303

P41002 O43303

P41208 O43303

P62158 O43303

Q5T7B8 O43303

Q8IW35 O43303

Q8TAP6 O43303

Q96JN8 O43303

Q9P0W5 O43303

Q9UBB9 O43303

Q9UBE8 O43303

Q9HAU0 O43307

P63165 O43309

O43312 O43312

Q14247 O43312

Q13315 O43313

P01106 O43314

O00743 O43318

O14733 O43318

O15111 O43318

P01106 O43318

P07900 O43318

P08238 O43318

P0CG48 O43318

P19634 O43318

P32121 O43318

P40763 O43318

P53041 O43318

P62877 O43318

P67775 O43318

Q13451 O43318

Q15750 O43318

Q16543 O43318

Q6NUN9 O43318

Q86Y07 O43318

Q8N5C8 O43318

Q8TAM1 O43318

Q9H8T0 O43318

Q9NV58 O43318

Q9NYJ8 O43318

Q9UBE8 O43318

Q9UDY8 O43318

Q9UQF2 O43318

Q9Y4K3 O43318

Q9Y6K9 O43318

O43318 O43318

O43809 O43318

P04350 O43318

P06733 O43318

P07437 O43318

P07814 O43318

P11021 O43318

P11142 O43318

P14868 O43318

P17844 O43318

P21333 O43318

P23396 O43318

P27708 O43318

P31689 O43318

P34931 O43318

P38646 O43318

P41252 O43318

P62158 O43318

P62805 O43318

P63261 O43318

Q00610 O43318

Q13748 O43318

Q8NHW5 O43318

Q9BVA1 O43318

Q9UQC1 O43318

Q9Y230 O43318

Q9Y265 O43318

P19320 O43324

Q13126 O43325

O14818 O43353

O43353 O43353

O94832 O43353

P04179 O43353

P04350 O43353

P04406 O43353

P06733 O43353

P07437 O43353

P07900 O43353

P08670 O43353

P11142 O43353

P15311 O43353

P17066 O43353

P21796 O43353

P22061 O43353

P28074 O43353

P29466 O43353

P30049 O43353

P30084 O43353

P34931 O43353

P35579 O43353

P35580 O43353

P51991 O43353

P54652 O43353

P55084 O43353

P57678 O43353

P60900 O43353

P62158 O43353

P62263 O43353

P63173 O43353

P63261 O43353

P98170 O43353

Q01469 O43353

Q13469 O43353

Q13489 O43353

Q13490 O43353

Q13748 O43353

Q15058 O43353

Q4G148 O43353

Q7Z434 O43353

Q96CX2 O43353

Q99623 O43353

Q9BVA1 O43353

Q9HC29 O43353

Q9NZ23 O43353

Q9UQC1 O43353

Q9Y239 O43353

Q9Y6K9 O43353

P21860 O43374

Q12889 O43374

Q9H910 O43374

Q9HDC9 O43374

O43379 O43379

P20226 O43379

P53779 O43379

P60520 O43379

Q5UIP0 O43379

Q92731 O43379

Q9UKM9 O43379

O15084 O43390

O95166 O43390

P00519 O43390

P03372 O43390

P11387 O43390

P16104 O43390

P16333 O43390

P19320 O43390

P27695 O43390

P32121 O43390

P46108 O43390

P60520 O43390

P61254 O43390

P67809 O43390

Q13573 O43390

Q15428 O43390

Q16637 O43390

Q5U5Q3 O43390

Q99873 O43390

Q9GZQ8 O43390

Q9H0R8 O43390

Q9H3D4 O43390

Q9H492 O43390

Q9UPE1 O43390

Q9Y285 O43390

O43172 O43395

O43395 O43395

O75554 O43395

O75940 O43395

O95391 O43395

O95758 O43395

P19320 O43395

P26368 O43395

P33993 O43395

P55081 O43395

Q13107 O43395

Q13435 O43395

Q14498 O43395

Q15020 O43395

Q15428 O43395

Q15834 O43395

Q15843 O43395

Q53GS9 O43395

Q8N5F7 O43395

Q8TBK6 O43395

Q8WYA6 O43395

Q92997 O43395

Q969Y2 O43395

Q99459 O43395

Q9C005 O43395

Q9Y4E8 O43395

P54578 O43396

Q9Y5K5 O43396

O43399 O43399

P55327 O43399

Q16890 O43399

Q96J77 O43399

O95400 O43426

P49418 O43426

P62993 O43426

Q07912 O43426

Q12965 O43426

Q15811 O43426

Q99961 O43426

Q99962 O43426

Q9Y5X1 O43426

O15294 O43427

P14174 O43427

P56537 O43427

P57678 O43427

Q00535 O43427

Q86YS7 O43427

Q9H0A6 O43427

Q9Y6B7 O43427

O15372 O43432

P03372 O43432

P60842 O43432

Q14240 O43432

Q14694 O43432

P18428 O43439

Q96HA8 O43439

Q9UPN9 O43439

O43172 O43447

O95758 O43447

P19320 O43447

Q13107 O43447

Q15020 O43447

Q9Y4E8 O43447

A5D8V7 O43463

O43159 O43463

O43167 O43463

O95243 O43463

O95260 O43463

P29590 O43463

P45973 O43463

P49639 O43463

P50749 O43463

P55055 O43463

P68431 O43463

Q01081 O43463

Q13547 O43463

Q14005 O43463

Q15156 O43463

Q15910 O43463

Q494V2 O43463

Q53FE4 O43463

Q5T0J7 O43463

Q68EA5 O43463

Q68G74 O43463

Q6NXT2 O43463

Q6P047 O43463

Q6P1K8 O43463

Q6PJQ5 O43463

Q6UXH1 O43463

Q6UXX9 O43463

Q6ZNL6 O43463

Q86W54 O43463

Q86X59 O43463

Q8N4C7 O43463

Q8NFT6 O43463

Q8TBB5 O43463

Q92769 O43463

Q96BD6 O43463

Q96EZ8 O43463

Q96GN5 O43463

Q96H12 O43463

Q96PV6 O43463

Q96SW2 O43463

Q96T60 O43463

Q9BS31 O43463

Q9BS34 O43463

Q9BYZ2 O43463

Q9C0F3 O43463

Q9C0K0 O43463

Q9H0H0 O43463

Q9H6K4 O43463

Q9UIS9 O43463

Q9UNL4 O43463

Q9Y2L8 O43463

Q9Y2M5 O43463

Q9Y4E5 O43463

P98170 O43464

Q12933 O43464

Q13489 O43464

Q9P0J0 O43464

P68104 O43464

Q04637 O43464

Q86XE2 O43464

Q99714 O43464

P42684 O43482

Q92851 O43482

Q92949 O43482

Q9NYP9 O43482

O95154 O43488

P19320 O43488

P60709 O43490

P04150 O43491

P61981 O43491

Q92905 O43491

P00519 O43493

P06241 O43493

P11717 O43493

P16333 O43493

P46108 O43493

P50281 O43493

P62993 O43493

P63010 O43493

Q7Z3C6 O43493

Q8N9I0 O43493

Q99640 O43493

Q9H3P7 O43493

Q9NZ52 O43493

Q9UIL1 O43493

P01106 O43497

O15273 O43504

O60343 O43504

P04406 O43504

P05556 O43504

P11055 O43504

P15173 O43504

P23246 O43504

P68104 O43504

Q05516 O43504

Q0VGL1 O43504

Q14161 O43504

Q15464 O43504

Q5UIP0 O43504

Q5VZM2 O43504

Q6IAA8 O43504

Q86TG7 O43504

Q92610 O43504

Q96AW1 O43504

Q96MU7 O43504

Q99081 O43504

Q9BVJ6 O43504

Q9H063 O43504

Q9HB90 O43504

Q9P2H0 O43504

Q9Y2Q5 O43504

Q9Y2X7 O43504

Q9NP84 O43508

A0JLT2 O43513

O95402 O43513

P49336 O43513

P51116 O43513

Q9BSL1 O43513

Q9BTT4 O43513

Q9BWU1 O43513

Q9BZE3 O43513

Q9NWA0 O43513

Q9NX70 O43513

O00401 O43516

O75554 O43516

P01106 O43516

P08631 O43516

P14317 O43516

P16333 O43516

P42768 O43516

P62993 O43516

Q14247 O43516

O43521 O43521

O75348 O43521

P10415 O43521

P10644 O43521

P17612 O43521

P31946 O43521

Q07812 O43521

Q07817 O43521

Q07820 O43521

Q08830 O43521

Q92843 O43521

Q92934 O43521

Q96GD4 O43521

P31749 O43521

P63167 O43521

P14174 O43521

O43524 O43524

O75323 O43524

P51610 O43524

P63104 O43524

Q04917 O43524

Q13485 O43524

Q16659 O43524

Q96EB6 O43524

Q9BSB4 O43524

Q9Y5Z7 O43524

Q00613 O43529

O75582 O43541

P51817 O43541

Q13950 O43541

Q16659 O43541

Q9C0C9 O43541

O95630 O43541

Q6NVH7 O43542

Q9P013 O43556

O95407 O43557

Q06643 O43557

Q92956 O43557

Q06124 O43559

O43889 O43561

P02768 O43561

P06241 O43561

P19174 O43561

P27986 O43561

P62993 O43561

P78314 O43561

Q9Y6X8 O43561

P08754 O43566

P19838 O43566

P42768 O43566

P63096 O43566

P51668 O43567

P61077 O43567

P62837 O43567

Q9Y2X8 O43567

P47929 O43570

P10644 O43572

P49748 O43583

Q8WX92 O43583

Q9NX47 O43583

Q9ULC4 O43583

P05408 O43586

P06729 O43586

P15924 O43586

P18031 O43586

P18077 O43586

P31939 O43586

P37268 O43586

P39023 O43586

P42768 O43586

P46779 O43586

P47914 O43586

P48023 O43586

P49207 O43586

P61254 O43586

P61513 O43586

P62750 O43586

P62899 O43586

P62910 O43586

P83881 O43586

Q02543 O43586

Q05209 O43586

Q15058 O43586

Q53ZZ1 O43586

Q75MH1 O43586

Q7L1Q6 O43586

Q8IZX4 O43586

Q96LW2 O43586

Q99952 O43586

Q99996 O43586

Q9BVP2 O43586

Q9H204 O43586

Q9NR30 O43586

Q9UBS9 O43586

Q9UJX6 O43586

Q9ULH1 O43586

Q9UMN6 O43586

Q9Y265 O43586

Q9Y3U8 O43586

Q9Y6E2 O43586

P00533 O43592

P01106 O43592

P19438 O43592

P62826 O43592

Q13286 O43592

Q15051 O43592

Q96RR4 O43592

P11473 O43593

Q92731 O43593

O60341 O43597

P04080 O43597

P21912 O43597

P22681 O43597

P32242 O43597

P35555 O43597

P50570 O43597

P52943 O43597

P53384 O43597

P55055 O43597

P57075 O43597

P62993 O43597

Q05516 O43597

Q14194 O43597

Q14584 O43597

Q6UY14 O43597

Q8IW50 O43597

Q9BQ66 O43597

Q9BSJ1 O43597

Q9BY42 O43597

Q9P283 O43597

O43598 O43598

Q9UKJ1 O43598

O15265 O43609

P04196 O43609

P22681 O43609

P40763 O43609

P52292 O43609

P01909 O43612

O95758 O43615

Q8IY92 O43615

O75419 O43617

O75865 O43617

P19320 O43617

Q86SZ2 O43617

Q96QF0 O43617

Q9UL33 O43617

P10600 O43633

P19320 O43633

P40818 O43633

P61457 O43633

Q93062 O43638

O15117 O43639

P04626 O43639

P05141 O43639

P09619 O43639

P0CG48 O43639

P15882 O43639

P21860 O43639

P26641 O43639

P51116 O43639

P52799 O43639

P54259 O43639

Q08345 O43639

Q13153 O43639

Q14185 O43639

Q15427 O43639

Q16630 O43639

Q5T5P2 O43639

Q6PJG9 O43639

Q7L0Q8 O43639

Q8N684 O43639

Q96L33 O43639

Q96SB4 O43639

Q9NQC3 O43639

Q9NZQ3 O43639

Q9NYB9 O43639

Q9Y2W2 O43639

Q12873 O43657

Q5UIP0 O43657

Q9Y328 O43657

O75934 O43660

O95166 O43660

P19784 O43660

P38919 O43660

P52272 O43660

P60520 O43660

P61326 O43660

Q13573 O43660

Q15428 O43660

Q99459 O43660

Q9H9J4 O43660

Q9UMS4 O43660

O94972 O43663

O95758 O43663

P35900 O43663

Q15834 O43663

Q8N6Y0 O43663

O43663 O43663

O00422 O43665

O43684 O43670

O75554 O43670

P36507 O43670

Q93009 O43670

Q9GZQ8 O43670

Q9Y371 O43670

O43678 O43674

Q17RG0 O43674

O43678 O43676

P02751 O43678

P19320 O43678

Q9Y328 O43678

P61968 O43679

Q9NVW2 O43679

P51693 O43680

Q9UIH9 O43680

O00555 O43681

O43681 O43681

P11441 O43681

Q7L5D6 O43681

O43264 O43683

O43683 O43683

O43684 O43683

O95229 O43683

O95376 O43683

P01106 O43683

P46108 O43683

Q13573 O43683

Q96NR8 O43683

Q99459 O43683

Q9H081 O43683

O43670 O43684

O60566 O43684

O75554 O43684

P10827 O43684

P19320 O43684

P51784 O43684

P51808 O43684

Q12834 O43684

Q13042 O43684

Q13573 O43684

Q15428 O43684

Q16186 O43684

Q8N5F7 O43684

Q8NHZ8 O43684

Q93009 O43684

Q99459 O43684

Q9GZQ8 O43684

Q9H0R8 O43684

Q9H1A4 O43684

Q9H492 O43684

Q9UJX2 O43684

Q9UJX4 O43684

Q9UJX5 O43684

Q9UKE5 O43684

P84022 O43684

Q15796 O43684

P40692 O43688

P46379 O43688

Q13432 O43688

O00151 O43707

P02751 O43707

P12830 O43707

P19320 O43707

P29474 O43707

P35222 O43707

P42677 O43707

P42771 O43707

P46940 O43707

P62993 O43707

P63104 O43707

Q07157 O43707

Q13164 O43707

Q8TDR0 O43707

Q9NP98 O43707

Q9NPC6 O43707

Q9NUX5 O43707

O43708 O43708

P00519 O43708

P06241 O43708

P36873 O43708

P00973 O43709

P01100 O43709

Q16512 O43709

P14921 O43711

P25791 O43711

P32121 O43719

Q08170 O43719

Q13573 O43719

Q92731 O43719

Q99689 O43719

O00463 O43734

Q08379 O43734

P05067 O43736

Q969H4 O43739

Q9P1Z2 O43739

O43741 O43741

P14920 O43741

P54619 O43741

P54646 O43741

Q12933 O43741

Q13131 O43741

Q8IYT8 O43741

Q9UGJ0 O43741

Q9UKT9 O43741

Q9UPY3 O43741

Q9Y478 O43741

P61106 O43747

Q10567 O43747

Q15276 O43747

Q6VY07 O43747

O75379 O43752

P46459 O43752

P51809 O43752

Q15836 O43752

Q86Y82 O43752

Q8TAC9 O43752

Q9BV40 O43752

O43889 O43759

Q969F0 O43760

O60844 O43765

O75925 O43765

O95967 O43765

P08238 O43765

P10145 O43765

P10451 O43765

P11142 O43765

P11441 O43765

P19438 O43765

P35080 O43765

P60520 O43765

Q13568 O43765

Q14653 O43765

Q8IW45 O43765

Q8TCT8 O43765

Q9BXJ1 O43765

Q9H2B2 O43765

Q9HB09 O43765

Q9UBU9 O43765

Q9UQK1 O43765

Q9Y624 O43765

P10114 O43768

P25205 O43768

P49768 O43768

Q9H910 O43772

P02751 O43776

Q92769 O43776

P04156 O43781

Q92673 O43781

O14986 O43791

O60496 O43791

O60684 O43791

O75925 O43791

O95197 O43791

P28702 O43791

P31150 O43791

Q7Z7J5 O43791

Q8IXJ6 O43791

Q8IYB3 O43791

Q99836 O43791

Q9UER7 O43791

Q9Y6Q9 O43791

O00716 O43795

P03372 O43795

P38606 O43795

P62993 O43795

Q14240 O43795

Q9H492 O43795

O43805 O43805

Q9UM82 O43805

P40855 O43808

O43809 O43809

O75533 O43809

O95166 O43809

P01023 O43809

P05386 O43809

P09651 O43809

P10074 O43809

P18754 O43809

P19320 O43809

P19387 O43809

P21246 O43809

P28702 O43809

P29353 O43809

P32322 O43809

P54253 O43809

P60520 O43809

P60896 O43809

P78362 O43809

Q13422 O43809

Q13523 O43809

Q13595 O43809

Q15652 O43809

Q16630 O43809

Q16643 O43809

Q5RL73 O43809

Q75N90 O43809

Q86X55 O43809

Q8N5V2 O43809

Q8N684 O43809

Q8NFF5 O43809

Q8WWI5 O43809

Q99459 O43809

Q9BWH2 O43809

Q9GZQ8 O43809

Q9GZT8 O43809

Q9GZV5 O43809

Q9H0R8 O43809

Q9NV58 O43809

Q9P283 O43809

Q9P2H0 O43809

Q9UJW3 O43809

Q9ULV5 O43809

Q9UQB3 O43809

P60520 O43813

P63104 O43813

O00506 O43815

P30153 O43815

P30154 O43815

P62714 O43815

P67775 O43815

Q13033 O43815

Q5VSL9 O43815

Q8WZ74 O43815

Q9BRV8 O43815

Q9BUL8 O43815

Q9H2K2 O43815

Q9NRL3 O43815

Q9P0W5 O43815

Q9P289 O43815

Q9P2B4 O43815

Q9UGI0 O43815

Q9Y228 O43815

Q9Y3A3 O43815

Q9Y6E0 O43815

P10114 O43818

P01106 O43819

P15735 O43819

Q13418 O43819

O15244 O43820

O00716 O43823

P01106 O43823

P03372 O43823

P09651 O43823

P68400 O43823

Q5S007 O43823

Q8NE63 O43823

Q92731 O43823

Q96DB2 O43823

Q99459 O43823

Q9P0W5 O43823

P01106 O43837

P50213 O43837

Q16659 O43837

Q99700 O43837

P01106 O43847

P04637 O43847

Q8IY92 O43847

Q96RL1 O43847

Q9UKB1 O43847

Q9Y297 O43847

O14965 O43852

P11021 O43852

P13569 O43852

P15336 O43852

Q15051 O43852

Q96DB2 O43852

Q9H492 O43852

P68400 O43852

Q00532 O43854

P49674 O43865

P78362 O43865

Q7Z7N5 O43865

Q8IY92 O43865

Q8TAF3 O43865

Q9H9J4 O43865

O14817 O43889

O15382 O43889

O43889 O43889

O60830 O43889

O75023 O43889

P05090 O43889

P05412 O43889

P09093 O43889

P11836 O43889

P22307 O43889

P23141 O43889

P34897 O43889

P41732 O43889

P43378 O43889

P48230 O43889

P48651 O43889

P49447 O43889

P51610 O43889

P51798 O43889

P54315 O43889

P56202 O43889

P62070 O43889

Q03518 O43889

Q07817 O43889

Q12893 O43889

Q13323 O43889

Q14534 O43889

Q14542 O43889

Q14773 O43889

Q15382 O43889

Q53HI1 O43889

Q6N075 O43889

Q6NSH3 O43889

Q6UX01 O43889

Q8N6L1 O43889

Q8TAC9 O43889

Q8WVV5 O43889

Q8WWG1 O43889

Q8WY98 O43889

Q92520 O43889

Q92624 O43889

Q96A57 O43889

Q96BI3 O43889

Q96DZ9 O43889

Q96GQ5 O43889

Q96GZ6 O43889

Q96I15 O43889

Q96IZ2 O43889

Q96LL9 O43889

Q9BQ16 O43889

Q9BQB6 O43889

Q9BRI3 O43889

Q9BRV3 O43889

Q9NRX5 O43889

Q9NRZ7 O43889

Q9NS37 O43889

Q9NTJ5 O43889

Q9NUH8 O43889

Q9NV12 O43889

Q9NV29 O43889

Q9UHQ4 O43889

Q9UJD0 O43889

Q9UNK0 O43889

Q9UPQ8 O43889

Q9Y287 O43889

Q9Y3D6 O43889

Q9Y6G1 O43889

Q8IUR6 O43889

Q9H295 O43889

P27348 O43896

P61981 O43896

P62258 O43896

Q04917 O43896

P00519 O43900

P06241 O43900

P12931 O43900

P16333 O43900

P46108 O43900

P19174 O43909

P27986 O43909

P46108 O43909

P62993 O43909

Q9HAU0 O43909

O43913 O43913

O43929 O43913

O75419 O43913

P33992 O43913

Q13137 O43913

Q13415 O43913

Q13416 O43913

Q15233 O43913

Q86VP1 O43913

Q9NRR5 O43913

Q9UBD5 O43913

O00241 O43914

O43914 O43914

P26717 O43914

P43405 O43914

Q14952 O43914

P55769 O43916

O43918 O43918

O60216 O43918

P00519 O43918

P11388 O43918

P12931 O43918

P13010 O43918

P16104 O43918

P19174 O43918

P46108 O43918

P62993 O43918

P68431 O43918

P78527 O43918

Q01130 O43918

Q8N1F7 O43918

Q9UER7 O43918

O43678 O43920

P43354 O43920

Q9Y375 O43920

P36404 O43924

P36405 O43924

P51153 O43924

P54725 O43924

Q15382 O43924

Q92834 O43924

P26641 O43927

O43913 O43929

P24385 O43929

P33993 O43929

Q13415 O43929

Q13416 O43929

Q96CD0 O43929

Q9NRD1 O43929

Q9UBD5 O43929

Q9UKA1 O43929

O75381 O43933

Q13608 O43933

Q7Z412 O43933

O95347 O60216

P49711 O60216

P51587 O60216

Q03188 O60216

Q13573 O60216

Q14683 O60216

Q29RF7 O60216

Q7Z5K2 O60216

Q8N3U4 O60216

Q8WVM7 O60216

Q9NP77 O60216

Q9NRI5 O60216

Q9NTI5 O60216

Q9UQE7 O60216

Q13085 O60218

Q15040 O60220

Q70CQ3 O60220

O15265 O60229

P01106 O60229

P13929 O60229

P38432 O60229

P48023 O60229

Q9GZM8 O60229

Q9NRI5 O60229

P19320 O60231

P38432 O60231

P68400 O60231

Q13435 O60231

Q92917 O60231

Q9HCS7 O60231

O60232 O60232

O60496 O60232

O75865 O60232

P04181 O60232

P14866 O60232

P15924 O60232

P48643 O60232

P81605 O60232

Q02413 O60232

Q53TS8 O60232

Q8N5R6 O60232

Q96NW7 O60232

Q99873 O60232

Q9BVI4 O60232

Q9BVV2 O60232

Q9H0J4 O60232

Q9NP79 O60232

Q9Y230 O60232

Q9Y262 O60232

Q9Y371 O60232

A5PKW4 O60234

Q8TAA9 O60234

O60238 O60238

P02545 O60238

P10415 O60238

P17152 O60238

Q01844 O60238

Q12983 O60238

Q16610 O60238

Q8NBE8 O60238

Q92934 O60238

Q9BXN2 O60238

Q9H492 O60238

Q9NQ11 O60238

Q9NRQ5 O60238

Q9P104 O60238

P02768 O60239

Q06187 O60239

Q9NRI5 O60239

Q9NV70 O60239

Q06546 O60241

Q06547 O60241

Q9Y3M8 O60241

Q9Y566 O60241

P17028 O60242

A0JLT2 O60244

O95402 O60244

P00519 O60244

P06241 O60244

P16333 O60244

P49336 O60244

P62993 O60244

Q92731 O60244

Q92830 O60244

Q9BTT4 O60244

Q9BWU1 O60244

Q9NWA0 O60244

Q9NX70 O60244

P41220 O60259

O14818 O60260

O60260 O60260

O60671 O60260

P49792 O60260

P57075 O60260

P63000 O60260

Q15645 O60260

Q16342 O60260

Q5S007 O60260

Q6NUN9 O60260

Q8IXI2 O60260

Q9BXM7 O60260

A4PIW0 O60264

O95758 O60264

P01106 O60264

P04179 O60264

P19320 O60264

P62993 O60264

Q13573 O60264

Q92731 O60264

Q96T23 O60264

Q99459 O60264

Q9H9Y6 O60264

Q9UIG0 O60264

Q9UPN9 O60264

O43597 O60269

Q96FJ2 O60269

Q9BQ66 O60269

O60271 O60271

P53350 O60271

P62330 O60271

Q14240 O60271

Q9GZQ8 O60271

Q9HC52 O60271

Q9NPJ4 O60271

P02768 O60281

P19784 O60282

P67870 O60282

P68400 O60282

Q8IXI2 O60282

Q9HCC6 O60282

P01877 O60285

P05109 O60285

P06702 O60285

P10644 O60285

Q14558 O60285

Q93008 O60285

Q15750 O60287

Q16659 O60287

Q96AY4 O60290

Q9ULK2 O60290

P51965 O60291

P62837 O60291

P63279 O60291

Q8WVN8 O60291

Q96B02 O60291

Q99816 O60291

Q9NPD8 O60291

Q9Y2X8 O60291

P01106 O60292

Q9Y463 O60292

P10242 O60293

Q15834 O60293

Q9Y463 O60299

P09651 O60306

P38919 O60306

P61326 O60306

Q13573 O60306

Q96NB3 O60306

Q96RU2 O60306

Q9BRX9 O60306

O43741 O60307

P54646 O60307

P60484 O60307

Q04917 O60307

P10588 O60308

P01106 O60313

Q12983 O60313

P84022 O60313

Q15796 O60313

P08473 O60313

O15198 O60315

P84022 O60315

Q7L5N1 O60315

Q9P0W5 O60315

O15198 O60318

P30304 O60318

P60953 O60318

P84022 O60318

Q09472 O60318

Q14194 O60318

Q9BPY3 O60318

Q9H5J8 O60318

O43504 O60333

P61981 O60333

P68104 O60333

Q04917 O60333

Q12800 O60333

Q7Z460 O60333

Q96G23 O60333

Q99497 O60333

Q9Y244 O60333

Q12959 O60333

Q96QZ7 O60333

P37231 O60337

A5D8V7 O60341

A8MW92 O60341

O00716 O60341

O14544 O60341

O14737 O60341

O15198 O60341

O15379 O60341

O43167 O60341

O43463 O60341

O43482 O60341

O60341 O60341

O60383 O60341

O75528 O60341

O94776 O60341

O95243 O60341

O95863 O60341

P01106 O60341

P04259 O60341

P04637 O60341

P08727 O60341

P08729 O60341

P12645 O60341

P15313 O60341

P22692 O60341

P35240 O60341

P45379 O60341

P45973 O60341

P49639 O60341

P49643 O60341

P50749 O60341

P54274 O60341

P54687 O60341

P57682 O60341

P59817 O60341

P62191 O60341

Q03181 O60341

Q04695 O60341

Q12873 O60341

Q13123 O60341

Q13133 O60341

Q13287 O60341

Q13432 O60341

Q13435 O60341

Q13547 O60341

Q13573 O60341

Q14141 O60341

Q14525 O60341

Q15047 O60341

Q15049 O60341

Q16695 O60341

Q16891 O60341

Q3KNS6 O60341

Q494V2 O60341

Q49A26 O60341

Q49A88 O60341

Q52LW3 O60341

Q53QZ3 O60341

Q5T0J7 O60341

Q5T6S3 O60341

Q5VU43 O60341

Q68CZ6 O60341

Q6P047 O60341

Q6P1K2 O60341

Q6PIW4 O60341

Q6ZU52 O60341

Q6ZUS5 O60341

Q70UQ0 O60341

Q7L2K0 O60341

Q7L2Z9 O60341

Q7Z4H7 O60341

Q86X59 O60341

Q86Y37 O60341

Q8IUI8 O60341

Q8IVI9 O60341

Q8IZL8 O60341

Q8IZU1 O60341

Q8IZS5 O60341

Q8N1A0 O60341

Q8N4C7 O60341

Q8N5U6 O60341

Q8N6F8 O60341

Q8N6V9 O60341

Q8NEC7 O60341

Q8NEZ2 O60341

Q8NHQ1 O60341

Q8NHQ8 O60341

Q8NHS9 O60341

Q8NHY6 O60341

Q8TAK5 O60341

Q8TBB5 O60341

Q8TBC4 O60341

Q8WV37 O60341

Q8WXI3 O60341

Q8WXU2 O60341

Q8WY64 O60341

Q92574 O60341

Q92766 O60341

Q92769 O60341

Q96AQ1 O60341

Q96CS2 O60341

Q96DB2 O60341

Q96FF9 O60341

Q96FW1 O60341

Q96H20 O60341

Q96JB6 O60341

Q96JL9 O60341

Q96LL4 O60341

Q96LY2 O60341

Q96PV6 O60341

Q96QG7 O60341

Q96T60 O60341

Q99459 O60341

Q99996 O60341

Q9BQS8 O60341

Q9BRX9 O60341

Q9BUZ4 O60341

Q9BX10 O60341

Q9BXG8 O60341

Q9BZK7 O60341

Q9C0F3 O60341

Q9GZT6 O60341

Q9H0H0 O60341

Q9H147 O60341

Q9H270 O60341

Q9H2G9 O60341

Q9H6K4 O60341

Q9H9P5 O60341

Q9HCM9 O60341

Q9NQZ8 O60341

Q9NR46 O60341

Q9NS23 O60341

Q9NU02 O60341

Q9NWQ4 O60341

Q9P0T4 O60341

Q9UBN7 O60341

Q9UBX0 O60341

Q9UGK8 O60341

Q9UJX2 O60341

Q9UQB8 O60341

Q9Y4E5 O60341

Q9Y575 O60341

O15042 O60343

O60303 O60343

O94916 O60343

P51970 O60343

P63104 O60343

Q01973 O60343

Q04917 O60343

Q6N021 O60343

Q8IY92 O60343

Q8NEY1 O60343

Q92547 O60343

Q96L91 O60343

Q96SN8 O60343

Q9HCM1 O60343

Q9NY93 O60343

Q9UK61 O60343

Q9ULJ6 O60343

O75317 O60346

O94782 O60346

P12830 O60346

P62068 O60346

Q09019 O60346

Q14160 O60346

Q7KZI7 O60346

Q8TAF3 O60346

Q8N474 O60353

P00352 O60356

Q15761 O60356

P15336 O60361

P06400 O60381

Q15797 O60381

Q8WXS4 O60381

Q96EK5 O60381

Q9Y5K6 O60381

O95257 O60383

P38936 O60383

P50570 O60383

Q99447 O60383

Q9BRP4 O60427

Q9UBM7 O60427

O76027 O60437

P05787 O60437

P31749 O60437

O14713 O60443

P21246 O60443

Q13573 O60443

Q99459 O60443

Q9UKT4 O60447

O15198 O60476

P08047 O60476

P13984 O60476

P09651 O60477

P24385 O60477

P63000 O60477

Q5BKZ1 O60477

Q96EB6 O60477

O95551 O60479

P18848 O60487

Q6UWV2 O60487

P63104 O60488

Q8N0X7 O60488

Q9P2Y5 O60488

Q9UL01 O60488

O95758 O60493

P00519 O60493

P06241 O60493

P16333 O60493

P19320 O60493

P46108 O60493

O60494 O60494

O60496 O60496

O95757 O60496

P01588 O60496

P16333 O60496

P61026 O60496

Q96EK5 O60496

Q9UBS4 O60496

O43639 O60500

P02768 O60500

P46940 O60500

P54257 O60502

Q00987 O60502

Q16543 O60502

Q96R06 O60502

Q9HC52 O60502

Q9NQC7 O60502

O14512 O60504

O15357 O60504

O43281 O60504

P02768 O60504

P38159 O60504

Q13177 O60504

Q5T5P2 O60504

Q6UY14 O60504

Q8N684 O60504

Q8TDM6 O60504

Q92841 O60504

Q9BST9 O60504

P00519 O60504

Q14151 O60504

O00401 O60504

P13631 O60504

P17612 O60504

Q9Y6W5 O60504

O75569 O60506

O95166 O60506

P02751 O60506

P03372 O60506

P16104 O60506

P17096 O60506

P19320 O60506

P27695 O60506

P60520 O60506

P67809 O60506

Q00839 O60506

Q13573 O60506

Q14103 O60506

Q5JVS0 O60506

Q96EB6 O60506

Q96Q15 O60506

Q99873 O60506

Q9GZQ8 O60506

Q9H0R8 O60506

Q9H3D4 O60506

Q9H492 O60506

Q9UL18 O60506

P38919 O60508

P61326 O60508

Q9UMS4 O60508

Q15527 O60512

O60573 O60516

P06730 O60516

P15927 O60516

O95166 O60518

P60520 O60518

Q15796 O60518

Q9H0R8 O60518

Q13573 O60535

Q05516 O60543

O60547 O60547

Q9H492 O60547

P04183 O60551

P04637 O60551

P09525 O60551

P10415 O60551

P20073 O60551

P42574 O60551

P67870 O60551

Q05513 O60551

Q16637 O60551

Q9BVK6 O60551

O00472 O60563

O15350 O60563

O94992 O60563

O95402 O60563

O95758 O60563

P42568 O60563

P50750 O60563

P51825 O60563

Q03111 O60563

Q03164 O60563

Q09472 O60563

Q13573 O60563

Q4G0J3 O60563

Q9UHB7 O60563

Q04917 O60565

O43683 O60566

O60566 O60566

O95229 O60566

P30260 O60566

Q12834 O60566

Q13042 O60566

Q13257 O60566

Q13573 O60566

Q8NHZ8 O60566

Q96DE5 O60566

Q99459 O60566

Q9H081 O60566

Q9H1A4 O60566

Q9UJX2 O60566

Q9UJX4 O60566

Q9UJX5 O60566

P01106 O60568

P04637 O60568

P60520 O60568

Q15834 O60568

Q7Z6L1 O60568

Q7Z7J5 O60568

Q96KQ7 O60568

Q9BQY4 O60568

Q9H0R8 O60568

Q9H492 O60568

O15372 O60573

P35900 O60573

Q04917 O60573

Q13107 O60573

Q8TF46 O60573

Q99750 O60573

Q9BQ66 O60573

Q9NRA8 O60573

O00472 O60583

O95402 O60583

P42568 O60583

P50750 O60583

Q9UHB7 O60583

Q99836 O60602

P61073 O60603

Q99836 O60603

O60610 O60610

O60861 O60610

O75400 O60610

O75554 O60610

P02751 O60610

P08631 O60610

P19320 O60610

P30622 O60610

P49593 O60610

P61586 O60610

P62745 O60610

P62993 O60610

Q13563 O60610

Q13573 O60610

Q99459 O60610

Q9HBH0 O60610

Q13145 O60613

Q8TDR0 O60641

O43684 O60645

P40692 O60645

Q13573 O60645

P20929 O60662

Q86VF7 O60662

O60664 O60664

O94955 O60664

P62380 O60664

Q9BZB8 O60669

O60921 O60671

Q99638 O60671

Q9GZQ8 O60671

O14492 O60674

O14744 O60674

O15524 O60674

P10276 O60674

P12931 O60674

P16333 O60674

P18031 O60674

P21860 O60674

P27986 O60674

P32927 O60674

P38159 O60674

P46527 O60674

P48357 O60674

P62993 O60674

Q12913 O60674

Q13283 O60674

Q9Y4K3 O60674

P50749 O60678

Q03181 O60678

O60683 O60683

O75381 O60683

P63279 O60683

Q13064 O60683

Q9BYE7 O60683

P63000 O60684

Q13309 O60684

Q13547 O60684

P22309 O60701

P62993 O60701

Q06609 O60701

Q9NTG7 O60701

Q9UKP3 O60701

Q9Y6K8 O60701

P13612 O60711

P18206 O60711

Q05397 O60711

Q16659 O60711

Q96BD5 O60711

Q96HA1 O60711

Q9BUY5 O60711

Q9NRQ2 O60711

Q9NV31 O60711

Q9UBN7 O60711

Q9Y473 O60711

P00533 O60716

P01106 O60716

P15336 O60716

P19022 O60716

P32121 O60716

P33993 O60716

P46937 O60716

Q12913 O60716

Q13501 O60716

Q6IQ23 O60716

Q7Z6J6 O60716

Q86T24 O60716

Q8TAF3 O60716

Q96CW1 O60716

Q99459 O60716

P00519 O60721

P06241 O60721

P16333 O60721

Q8IXJ6 O60729

O75964 O60732

P0C0S5 O60732

P35269 O60732

P78358 O60732

O00151 O60739

O00264 O60739

O00303 O60739

O14641 O60739

O14737 O60739

O14818 O60739

O15371 O60739

O15372 O60739

O43324 O60739

O43809 O60739

O75821 O60739

P00451 O60739

P00492 O60739

P00505 O60739

P04179 O60739

P04181 O60739

P04406 O60739

P05198 O60739

P06753 O60739

P07355 O60739

P09936 O60739

P10599 O60739

P12004 O60739

P12532 O60739

P13693 O60739

P13797 O60739

P13804 O60739

P14174 O60739

P16152 O60739

P19105 O60739

P22061 O60739

P22234 O60739

P24534 O60739

P24666 O60739

P24752 O60739

P25787 O60739

P26583 O60739

P27695 O60739

P27797 O60739

P29401 O60739

P29692 O60739

P30046 O60739

P30049 O60739

P30084 O60739

P30085 O60739

P30153 O60739

P35244 O60739

P35268 O60739

P35544 O60739

P35637 O60739

P37108 O60739

P37802 O60739

P37837 O60739

P39019 O60739

P39023 O60739

P41091 O60739

P42766 O60739

P42771 O60739

P43487 O60739

P46781 O60739

P46783 O60739

P47813 O60739

P47914 O60739

P48047 O60739

P49720 O60739

P49903 O60739

P50395 O60739

P51149 O60739

P51571 O60739

P51668 O60739

P52434 O60739

P52907 O60739

P54577 O60739

P54819 O60739

P59998 O60739

P60228 O60739

P60900 O60739

P60981 O60739

P61158 O60739

P61160 O60739

P61247 O60739

P61254 O60739

P61289 O60739

P61923 O60739

P62158 O60739

P62249 O60739

P62263 O60739

P62273 O60739

P62280 O60739

P62854 O60739

P62857 O60739

P62899 O60739

P62910 O60739

P63279 O60739

P67812 O60739

P68036 O60739

P84090 O60739

Q01105 O60739

Q01469 O60739

Q01844 O60739

Q02543 O60739

Q09028 O60739

Q12888 O60739

Q13155 O60739

Q13347 O60739

Q13526 O60739

Q14152 O60739

Q14D04 O60739

Q15008 O60739

Q15056 O60739

Q15365 O60739

Q15369 O60739

Q15382 O60739

Q15631 O60739

Q15691 O60739

Q15819 O60739

Q16543 O60739

Q16658 O60739

Q2NL82 O60739

Q5SQT9 O60739

Q6FGD7 O60739

Q6FGG2 O60739

Q6PKG0 O60739

Q7L2H7 O60739

Q86UE4 O60739

Q8N983 O60739

Q8TEW0 O60739

Q8WVJ2 O60739

Q96CX2 O60739

Q96FW1 O60739

Q99436 O60739

Q99873 O60739

Q9BSL1 O60739

Q9BWD1 O60739

Q9GZZ1 O60739

Q9HB71 O60739

Q9NQP4 O60739

Q9NR99 O60739

Q9NRN7 O60739

Q9NX55 O60739

Q9NZ23 O60739

Q9UBQ5 O60739

Q9UHV9 O60739

Q9UN86 O60739

Q9UNM6 O60739

Q9Y262 O60739

Q9Y265 O60739

Q9Y266 O60739

Q9Y3F4 O60739

Q9Y3U8 O60739

Q9Y5K5 O60739

Q9UL18 O60747

O95125 O60749

P11047 O60749

P67870 O60749

Q6ZSJ8 O60749

Q96RU3 O60749

Q99459 O60749

Q9UNH7 O60749

Q9Y5X3 O60749

Q9Y6W6 O60749

Q15438 O60759

P01106 O60762

Q05516 O60762

Q13418 O60762

P62820 O60763

Q13114 O60763

Q6P1W5 O60763

Q99459 O60763

Q9BS26 O60763

P21860 O60783

P67809 O60783

Q14197 O60783

Q9H492 O60783

Q9H9J4 O60783

Q9NY93 O60783

A1L0T0 O60784

O14964 O60784

O75674 O60784

P42677 O60784

Q15075 O60784

Q7Z3T8 O60784

Q9H0E2 O60784

Q9UKA9 O60784

P19525 O60812

P67809 O60812

P78362 O60812

Q92731 O60812

Q9GZQ8 O60812

Q9H3D4 O60812

Q9H492 O60812

P51858 O60814

O60825 O60825

Q04917 O60825

O60306 O60828

P09497 O60828

P35243 O60828

P61006 O60828

P68104 O60828

Q15428 O60828

Q5UIP0 O60828

Q9UKR5 O60828

Q9Y2W2 O60828

Q9Y3C7 O60828

Q9BQ83 O60830

O60664 O60831

O60831 O60831

O75915 O60831

P51681 O60831

O95166 O60832

P03372 O60832

P26641 O60832

P32121 O60832

P33993 O60832

P49407 O60832

Q8TAF3 O60832

Q96DB2 O60832

Q96HR8 O60832

Q9BUR4 O60832

Q9Y230 O60832

Q9Y265 O60832

P06241 O60840

O15264 O60841

P02751 O60841

P04150 O60841

P14921 O60841

P51858 O60841

P68400 O60841

P83916 O60841

Q13131 O60841

Q13573 O60841

Q14457 O60841

Q99459 O60841

Q9NRR5 O60844

Q9UMX0 O60844

P23246 O60861

P55160 O60861

Q00839 O60861

Q14457 O60861

Q14820 O60861

Q15459 O60861

Q8IZP0 O60861

Q96F07 O60861

Q9BY11 O60861

Q9NWJ6 O60861

Q9Y2A7 O60861

Q9Y6W5 O60861

O00482 O60869

P03372 O60869

P19793 O60869

P37231 O60869

Q13133 O60869

Q13285 O60869

P01100 O60869

P05412 O60869

P20226 O60869

P42785 O60883

O95758 O60884

P00533 O60884

P01106 O60884

P04626 O60884

P07900 O60884

P09913 O60884

P13569 O60884

P29353 O60884

P62805 O60884

P62993 O60884

P84022 O60884

Q15796 O60884

Q96DB2 O60884

Q9GZQ8 O60884

Q9GZX7 O60884

Q9HCK5 O60884

Q9UL18 O60884

O75400 O60885

O95832 O60885

P10276 O60885

P16333 O60885

P27986 O60885

P62993 O60885

Q14696 O60885

Q6NYC1 O60885

Q96EP1 O60885

Q99459 O60885

Q9BPX7 O60885

Q9H7E9 O60885

P22736 O60888

P40692 O60888

P01106 O60890

P11802 O60902

O15379 O60907

O60479 O60907

Q07666 O60907

Q8WUI4 O60907

Q9BZK7 O60907

Q9UQL6 O60907

O15212 O60909

A5PKW4 O60921

O60671 O60921

P00492 O60921

P05062 O60921

P25787 O60921

P41217 O60921

Q99638 O60921

Q9Y3F4 O60921

O00291 O60925

O14777 O60925

O43172 O60925

O43681 O60925

P05386 O60925

P10645 O60925

P13378 O60925

P54257 O60925

P59665 O60925

P62857 O60925

Q06323 O60925

Q12766 O60925

Q13164 O60925

Q14203 O60925

Q16891 O60925

Q3B7T1 O60925

Q5R3I4 O60925

Q5UIP0 O60925

Q5VU43 O60925

Q8IXL6 O60925

Q8WUW1 O60925

Q93063 O60925

Q96AW1 O60925

Q96DF8 O60925

Q96PX6 O60925

Q9BT40 O60925

Q9H254 O60925

Q9H7V2 O60925

Q9H9A7 O60925

Q9P2H0 O60925

Q9Y2X7 O60925

Q9Y3C7 O60925

Q9Y5X3 O60925

Q5VYV7 O60930

P09874 O60934

P16104 O60934

Q13573 O60934

Q14676 O60934

Q92878 O60934

Q96EB6 O60934

Q99459 O60934

Q9BXW9 O60934

Q9HC52 O60934

O60936 O60936

P78324 O60936

Q14790 O60936

Q9GZT8 O60936

Q9NVF9 O60936

P37198 O60941

Q12933 O60941

Q15834 O60941

Q8ND30 O60941

Q96A04 O60941

Q9BUH8 O60941

Q9NP66 O60941

Q9NRG1 O60941

O95477 O60941

P01106 O60942

P01106 O75027

Q14197 O75027

P52790 O75030

P63104 O75030

O14744 O75044

O75044 O75044

O95466 O75044

P0DJJ0 O75044

P48023 O75044

P61981 O75044

Q04917 O75044

Q13153 O75044

Q9NRI5 O75044

Q14157 O75051

Q8IY37 O75051

Q8IYU2 O75051

Q8N3X1 O75051

Q8WUJ3 O75051

Q8WUM4 O75051

P32121 O75056

P49407 O75056

P02649 O75069

P19784 O75071

O76061 O75078

P51805 O75081

Q9UFB7 O75081

Q9UKD1 O75081

Q9UPN9 O75081

Q9Y2D9 O75081

P62993 O75083

P67870 O75083

Q15477 O75083

O00560 O75084

P56703 O75084

Q9NRR5 O75084

Q9UMX0 O75084

O00555 O75095

O15265 O75095

P00533 O75095

P49407 O75096

Q9P202 O75096

P03372 O75110

P02768 O75112

O75716 O75116

O95793 O75116

P25963 O75116

Q13573 O75116

Q14254 O75116

Q96C90 O75116

Q99459 O75116

O95758 O75122

P11217 O75122

P49841 O75122

Q04917 O75122

Q92731 O75122

Q99996 O75122

Q14197 O75127

Q9BY11 O75128

Q13573 O75131

Q9BY41 O75131

Q9H9J4 O75131

O00716 O75132

P08238 O75132

P83916 O75132

O75143 O75143

O75385 O75143

O95166 O75143

P60520 O75143

Q8IYT8 O75143

Q8TDY2 O75143

Q9BSB4 O75143

Q9GZQ8 O75143

Q9H0R8 O75143

P27986 O75145

P67775 O75145

P19320 O75146

Q13573 O75146

Q53HG7 O75146

Q99459 O75146

A7MCY6 O75147

P02768 O75147

Q15759 O75147

Q16539 O75147

Q8IYT8 O75147

Q8WVZ9 O75147

Q8WZ42 O75147

Q96RR4 O75147

P04637 O75150

P08238 O75150

P49459 O75150

P63146 O75150

Q5VTR2 O75150

Q9BSJ1 O75150

Q9BU64 O75150

Q9Y4E5 O75150

Q9Y4E8 O75150

O00716 O75152

P38919 O75152

P40692 O75152

P61326 O75152

Q8NCN4 O75152

Q9UKV0 O75152

O75190 O75155

Q969X1 O75159

P62805 O75164

P68431 O75164

Q16695 O75164

Q8TAF3 O75164

P00533 O75165

P38606 O75165

Q13573 O75165

Q9UBS4 O75165

P12931 O75167

O00743 O75170

O15084 O75170

P53350 O75170

P54253 O75170

Q70CQ1 O75170

Q05516 O75173

A5YKK6 O75175

P50616 O75175

Q02535 O75175

Q96LI5 O75175

Q99459 O75175

Q9NZN8 O75175

Q9UBE8 O75175

Q9UFF9 O75175

Q9ULM6 O75175

Q15427 O75177

Q70CQ3 O75177

Q8HWS3 O75177

Q92567 O75177

P19320 O75179

P25054 O75179

Q00987 O75179

Q8IY92 O75179

Q92560 O75179

Q9HC29 O75179

P36873 O75182

P48382 O75182

Q13547 O75182

Q92769 O75182

Q96D31 O75185

O75563 O75190

P01106 O75190

P04626 O75190

P05783 O75190

P11142 O75190

P36871 O75190

P42336 O75190

P52179 O75190

P58340 O75190

Q13326 O75190

Q15327 O75190

Q15773 O75190

Q7KZ85 O75190

Q8IY92 O75190

Q8WZ42 O75190

Q96CW5 O75190

Q96DB2 O75190

Q9Y2S0 O75190

Q00604 O75197

Q6IMN6 O75197

O94805 O75208

Q9HCE9 O75208

P00533 O75223

O43678 O75251

Q9Y375 O75251

Q7KZI7 O75271

Q9UK80 O75271

O43678 O75306

O75489 O75306

P01106 O75306

P11161 O75306

P30519 O75306

Q09019 O75306

Q13501 O75306

Q14197 O75306

Q16795 O75306

Q9BU61 O75306

P35575 O75309

Q13368 O75312

O60346 O75317

P62068 O75317

Q09019 O75317

Q6ZVD8 O75317

Q8TAF3 O75317

Q8TBZ3 O75317

P47897 O75319

O75323 O75323

O95166 O75323

P11217 O75323

P12830 O75323

P41743 O75323

P60520 O75323

Q13501 O75323

Q14145 O75323

Q9BXB5 O75323

Q9BXW4 O75323

Q9GZQ8 O75323

Q9H0R8 O75323

Q9H492 O75323

Q9HD90 O75325

O60486 O75326

O75326 O75326

P00519 O75326

P06241 O75326

P62993 O75326

O95758 O75330

P48729 O75330

P49674 O75330

Q9UHD2 O75330

P30153 O75334

P67775 O75334

Q99459 O75334

P19174 O75335

Q9UPW5 O75335

P01137 O75339

O75340 O75340

O94979 O75340

O95758 O75340

P13569 O75340

P16104 O75340

P19320 O75340

P35968 O75340

P42771 O75340

P53355 O75340

P60520 O75340

P62993 O75340

Q8NDC0 O75340

Q8WUM4 O75340

Q9GZQ8 O75340

Q9H3S7 O75340

Q9H492 O75340

Q9NRY6 O75340

Q9UBV8 O75340

Q9Y5Z4 O75340

Q9Y6Y8 O75340

P22735 O75342

Q9BYJ1 O75342

O75909 O75344

Q9Y5B8 O75344

P38606 O75348

Q96A05 O75348

Q9NWV8 O75351

O95714 O75352

P61247 O75352

Q15014 O75352

Q15126 O75352

Q15758 O75352

Q9H0J4 O75352

O75356 O75356

Q13547 O75362

Q92769 O75362

P63167 O75363

Q9Y230 O75363

A0N4V7 O75365

O00159 O75365

O14787 O75365

O14925 O75365

O15260 O75365

O43156 O75365

O43242 O75365

O43264 O75365

O43795 O75365

O60313 O75365

O60762 O75365

O60763 O75365

O75365 O75365

O76031 O75365

O94822 O75365

O94874 O75365

O95347 O75365

O95816 O75365

O95831 O75365

P04181 O75365

P08195 O75365

P12004 O75365

P16615 O75365

P24941 O75365

P25205 O75365

P28288 O75365

P28340 O75365

P31689 O75365

P33993 O75365

P39656 O75365

P42224 O75365

P42704 O75365

P43246 O75365

P49354 O75365

P49356 O75365

P51571 O75365

P52272 O75365

P54709 O75365

P57088 O75365

P57678 O75365

P58107 O75365

P61619 O75365

P78330 O75365

P78344 O75365

P78347 O75365

Q13257 O75365

Q13617 O75365

Q13733 O75365

Q14139 O75365

Q14558 O75365

Q15029 O75365

Q15042 O75365

Q15386 O75365

Q29RF7 O75365

Q5SRE5 O75365

Q5VYK3 O75365

Q6P3X3 O75365

Q6P996 O75365

Q7Z4Q2 O75365

Q86Y56 O75365

Q8N163 O75365

Q8NBQ5 O75365

Q8NE01 O75365

Q8TC07 O75365

Q8TEX9 O75365

Q8WUM4 O75365

Q92538 O75365

Q96T76 O75365

Q99653 O75365

Q9BPX3 O75365

Q9C0B7 O75365

Q9H2M9 O75365

Q9H3U1 O75365

Q9H583 O75365

Q9HAV4 O75365

Q9NRH3 O75365

Q9NS69 O75365

Q9NU22 O75365

Q9NVH1 O75365

Q9NVI1 O75365

Q9NVI7 O75365

Q9P035 O75365

Q9UBB4 O75365

Q9UBB6 O75365

Q9UBS4 O75365

Q9UGP8 O75365

Q9UHI6 O75365

Q9UI12 O75365

Q9UI26 O75365

Q9UIA9 O75365

Q9UJ14 O75365

Q9UNL2 O75365

Q9Y5L0 O75365

Q9Y5M8 O75365

Q9Y5Y2 O75365

Q9Y6D6 O75365

P15336 O75367

P19320 O75367

P04626 O75367

O75369 O75369

P02751 O75369

P03372 O75369

P05161 O75369

P19174 O75369

P19320 O75369

P21333 O75369

P27986 O75369

P40692 O75369

P46108 O75369

P49810 O75369

P62993 O75369

Q13233 O75369

Q13283 O75369

Q14324 O75369

Q53GL0 O75369

Q5VV17 O75369

Q6VMQ6 O75369

Q92731 O75369

Q9GZQ8 O75369

Q9H0R8 O75369

Q9HAU4 O75369

O15379 O75376

O75386 O75376

O95758 O75376

P01106 O75376

P03372 O75376

P06733 O75376

P10275 O75376

P10276 O75376

P12755 O75376

P37231 O75376

P56524 O75376

P62258 O75376

Q13263 O75376

Q13573 O75376

Q6MZP7 O75376

Q7L2E3 O75376

Q8WUI4 O75376

Q96EB6 O75376

Q99459 O75376

Q9BZK7 O75376

Q9UI36 O75376

Q9UKV0 O75376

Q9UQL6 O75376

Q9Y5X4 O75376

Q86Y82 O75379

Q8TAC9 O75379

O43318 O75380

O43678 O75380

O75381 O75381

P40855 O75381

P50542 O75381

Q86WA8 O75381

Q8TAQ2 O75381

Q92968 O75381

Q96G25 O75381

Q9UKY1 O75381

O75143 O75385

O75323 O75385

O75385 O75385

O95166 O75385

P54646 O75385

Q16625 O75385

Q676U5 O75385

Q7Z3C6 O75385

Q8TDY2 O75385

Q96F24 O75385

Q99689 O75385

Q9BSB4 O75385

Q9BXW4 O75385

Q9GZQ8 O75385

Q9H0R8 O75385

Q9P2M4 O75385

O14647 O75386

O14983 O75386

P10644 O75386

P11055 O75386

P14635 O75386

P62508 O75386

Q03001 O75386

Q13118 O75386

Q14324 O75386

Q14687 O75386

Q5PSV4 O75386

Q5QJE6 O75386

Q6ZN11 O75386

Q8IUG5 O75386

Q8IVI9 O75386

Q8IWI9 O75386

Q8N2I2 O75386

Q8N5U6 O75386

Q96JM2 O75386

Q99683 O75386

Q9H4A3 O75386

Q9HCG1 O75386

Q9NRR4 O75386

Q9NWQ4 O75386

Q9P243 O75386

Q9UBU8 O75386

Q9Y496 O75386

P15336 O75390

P24386 O75390

P51784 O75390

O43889 O75396

O94766 O75396

O95249 O75396

P02751 O75396

Q13190 O75396

P01116 O75398

P04183 O75398

P38936 O75398

P42771 O75398

P49841 O75398

P52758 O75398

P54727 O75398

P62136 O75398

Q13155 O75398

Q16543 O75398

Q9HAT8 O75398

O15042 O75400

O75400 O75400

P08621 O75400

P10586 O75400

P10644 O75400

P17858 O75400

P18206 O75400

P18583 O75400

P40123 O75400

P42858 O75400

P63279 O75400

Q01081 O75400

Q13129 O75400

Q13435 O75400

Q13485 O75400

Q13573 O75400

Q14004 O75400

Q14562 O75400

Q15459 O75400

Q70Z53 O75400

Q7Z5N4 O75400

Q8IVF7 O75400

Q8N5F7 O75400

Q8TBK6 O75400

Q9BUJ2 O75400

Q9H492 O75400

Q9NPH3 O75400

Q9NRR5 O75400

Q9NYV4 O75400

Q9P246 O75400

Q9UMX0 O75400

Q9UQ88 O75400

Q9Y388 O75400

Q9Y4E5 O75400

Q9Y6D5 O75400

Q9Y6X2 O75400

Q00005 O75400

O14965 O75410

O60341 O75410

O95619 O75410

Q14008 O75410

Q8NHU6 O75410

P62308 O75410

Q9UK45 O75410

O60232 O75414

P02768 O75419

P15927 O75419

P51587 O75419

Q13501 O75419

Q92547 O75419

Q96NX5 O75419

Q9HAW4 O75419

Q9NTI5 O75419

P54259 O75420

Q92731 O75420

Q99750 O75427

Q9NQC3 O75427

Q13505 O75431

Q16891 O75431

P02751 O75436

Q9BVS4 O75436

Q14197 O75439

P35869 O75444

Q13547 O75446

Q86X95 O75446

Q92769 O75446

Q96ST3 O75446

Q9UPN9 O75446

Q9Y618 O75446

A0JLT2 O75448

O14788 O75448

O95402 O75448

P49336 O75448

P49841 O75448

Q15648 O75448

Q15796 O75448

Q92731 O75448

Q92830 O75448

Q9BTT4 O75448

Q9BWU1 O75448

Q9NWA0 O75448

Q9NX70 O75448

O75460 O75460

Q07812 O75460

Q12933 O75460

Q86TM6 O75460

Q9H2K8 O75460

P10398 O75461

P35226 O75461

Q14186 O75461

Q14188 O75461

Q8N488 O75461

P61964 O75461

P45973 O75467

P08238 O75469

P09769 O75469

Q13164 O75469

P49841 O75474

O00255 O75475

P03372 O75475

P17096 O75475

P51858 O75475

P63165 O75475

Q16659 O75475

P19320 O75477

O14964 O75478

O75528 O75478

O95990 O75478

Q8IYI6 O75478

Q92831 O75478

P38398 O75486

Q92830 O75486

Q92831 O75486

Q9UPT9 O75486

O95166 O75487

P19784 O75487

P24863 O75487

Q13501 O75487

Q15051 O75487

Q96GS6 O75487

O43678 O75489

O75306 O75489

P01106 O75489

P02751 O75489

P12544 O75489

P19320 O75489

P51970 O75489

Q13418 O75489

Q14197 O75489

Q8NC60 O75489

Q9BU61 O75489

Q9Y375 O75489

O95166 O75494

P31947 O75494

P38919 O75494

P60520 O75494

P61326 O75494

P61981 O75494

P62993 O75494

P78362 O75494

Q07955 O75494

Q08170 O75494

Q96SB4 O75494

Q9H492 O75494

Q9UL18 O75494

D6RGH6 O75496

P24941 O75496

P27540 O75496

Q9H211 O75496

Q6MZP7 O75506

Q8TDY2 O75506

Q9Y3C0 O75506

P05067 O75509

Q6VEQ5 O75509

O60341 O75521

Q2T9J0 O75521

Q9Y2K6 O75521

O75525 O75525

P07910 O75525

P35637 O75525

P38159 O75525

P61978 O75525

Q07666 O75525

Q5VWX1 O75525

Q6ZVK8 O75525

Q7Z6E9 O75525

Q8N5R6 O75525

Q8TBB1 O75525

Q8TBE0 O75525

Q8WX92 O75525

Q92530 O75525

Q9H9D4 O75525

Q9Y580 O75525

O95166 O75526

P60520 O75526

Q92731 O75526

Q9GZQ8 O75526

Q9H0R8 O75526

Q9H492 O75526

O75822 O75528

P11142 O75528

P22670 O75528

P38398 O75528

P45974 O75528

Q8N2W9 O75528

Q8WYH8 O75528

Q92830 O75528

Q92831 O75528

Q96ES7 O75528

Q9BRR6 O75528

Q9H213 O75528

Q9UPT9 O75528

P10276 O75529

P16104 O75529

P38936 O75529

Q92830 O75529

Q92831 O75529

Q99459 O75529

Q9UPT9 O75529

A4PIW0 O75530

O43809 O75530

O75530 O75530

O75956 O75530

P15336 O75530

P26358 O75530

P35226 O75530

P46783 O75530

Q04724 O75530

Q13547 O75530

Q13619 O75530

Q13620 O75530

Q13642 O75530

Q14774 O75530

Q15022 O75530

Q15156 O75530

Q15714 O75530

Q15910 O75530

Q16531 O75530

Q8IXJ9 O75530

Q8IZ13 O75530

Q96EB6 O75530

Q99496 O75530

Q9UBC3 O75530

O95758 O75531

P38606 O75531

O00193 O75533

O15198 O75533

O15379 O75533

O43660 O75533

O75554 O75533

O95166 O75533

O95758 O75533

P01106 O75533

P02751 O75533

P03372 O75533

P11387 O75533

P32121 O75533

P38919 O75533

P61326 O75533

P62136 O75533

P68400 O75533

Q13573 O75533

Q15797 O75533

Q96DI7 O75533

Q99459 O75533

Q99717 O75533

Q9UKV8 O75533

Q9UL18 O75533

Q9Y3B4 O75533

P46977 O75534

P63279 O75534

Q96LR2 O75541

P09012 O75554

Q5H919 O75554

Q5SZ64 O75554

Q8WXF1 O75554

Q9BUQ8 O75554

O00161 O75558

O43463 O75558

O60341 O75558

O60493 O75558

O75509 O75558

O75558 O75558

O75909 O75558

P17980 O75558

P25788 O75558

P56545 O75558

Q96QD8 O75558

Q9Y6J9 O75558

Q9Y6K9 O75558

P48023 O75563

P62993 O75563

O60383 O75569

O75569 O75569

P19525 O75569

P29353 O75569

P62487 O75569

Q14558 O75569

Q15047 O75569

Q15633 O75569

Q6NUN9 O75569

Q7L5N1 O75569

Q8NHY6 O75569

Q92731 O75569

Q9NZI8 O75569

Q9UKV8 O75569

Q9UPE1 O75569

Q9UPY3 O75569

Q9Y3U8 O75569

Q9Y5X3 O75569

P01106 O75570

O15169 O75581

P49841 O75581

P58335 O75581

P98082 O75581

Q03135 O75581

Q5S007 O75581

Q6IMN6 O75581

Q9H6X2 O75581

Q9NQ11 O75581

Q9Y4X0 O75581

O00151 O75582

O95865 O75582

P04150 O75582

P08238 O75582

P10636 O75582

P27694 O75582

P67870 O75582

P84090 O75582

Q12824 O75582

Q14194 O75582

Q15811 O75582

Q16539 O75582

Q6PKC3 O75582

Q96B36 O75582

Q96BV0 O75582

Q96D09 O75582

Q9BVK2 O75582

Q9HCK4 O75582

Q9NRM6 O75582

Q9NV58 O75582

Q9NVH1 O75582

Q9NYL2 O75582

A0JLT2 O75586

O95402 O75586

P36956 O75586

P49336 O75586

Q12772 O75586

Q9BTT4 O75586

Q9BWU1 O75586

Q9NWA0 O75586

Q9NX70 O75586

P24386 O75592

P31946 O75592

P51784 O75592

Q14781 O75592

Q5U5Q3 O75592

Q8TAF3 O75592

Q96DB2 O75592

Q9H492 O75592

P16333 O75593

P27986 O75593

P19012 O75604

Q00987 O75604

Q15834 O75604

Q7L4P6 O75604

Q96ED9 O75604

Q9BUH8 O75604

Q9P2H0 O75607

P19320 O75608

Q14197 O75616

Q99459 O75616

P29374 O75618

P36543 O75618

P50053 O75618

P84022 O75618

Q06481 O75618

Q5H9L2 O75618

Q7L4I2 O75618

Q93009 O75618

Q96GA3 O75618

Q9H6Z4 O75618

Q9P031 O75618

Q9UPT9 O75618

P08238 O75626

P63165 O75626

O15541 O75643

O43187 O75643

O75643 O75643

O95166 O75643

P01106 O75643

P07900 O75643

P11387 O75643

P19320 O75643

P38919 O75643

P58753 O75643

P60520 O75643

P61326 O75643

Q13523 O75643

Q13573 O75643

Q15796 O75643

Q53GS9 O75643

Q5U5Q3 O75643

Q6P2Q9 O75643

Q96DI7 O75643

Q99459 O75643

Q9H0R8 O75643

Q9H492 O75643

Q9UL18 O75643

O00743 O75663

P62714 O75663

P67775 O75663

P78318 O75663

Q8IY92 O75663

Q9NY27 O75663

P53350 O75665

Q13352 O75665

Q96AW1 O75665

Q9H7U1 O75665

Q9P2H0 O75665

Q9Y383 O75665

Q9Y5B8 O75665

O60784 O75674

P00533 O75674

P52735 O75674

P62993 O75674

P68363 O75674

Q00610 O75674

Q15075 O75674

Q99759 O75674

Q9H0E2 O75674

Q13573 O75676

Q16539 O75676

Q16543 O75676

Q99459 O75676

Q92731 O75683

A0N4V7 O75688

O95267 O75688

P01100 O75688

P04083 O75688

P05109 O75688

P05161 O75688

P05783 O75688

P06396 O75688

P07355 O75688

P09327 O75688

P27448 O75688

P30084 O75688

P32121 O75688

P49407 O75688

P50750 O75688

P52735 O75688

P55072 O75688

P62993 O75688

Q13310 O75688

P49841 O75689

Q5T8A7 O75691

O95758 O75694

P02751 O75694

P19320 O75694

Q09019 O75694

Q13573 O75694

Q6GQQ9 O75694

Q8IY92 O75694

Q8WVZ9 O75694

Q99459 O75694

P36405 O75695

P49247 O75716

P67870 O75716

Q6P2I7 O75716

Q6UY14 O75716

Q8N5Z5 O75716

Q9HB66 O75716

Q9NRH2 O75716

O00257 O75717

Q8TAF3 O75718

A0AUL9 O75746

P01106 O75746

P15336 O75746

P17535 O75746

P84022 O75746

Q14197 O75746

Q9Y2R0 O75746

P00519 O75751

P16333 O75751

Q99741 O75752

O43543 O75771

P81605 O75771

Q13422 O75771

Q6NVH7 O75771

Q96C19 O75771

Q96JH8 O75771

Q9Y2J4 O75771

Q9Y2K6 O75787

O15117 O75791

O15156 O75791

O95630 O75791

P02768 O75791

P04626 O75791

Q13094 O75791

Q13480 O75791

Q7L4P6 O75791

Q93062 O75791

Q9BQ66 O75791

Q9H3S7 O75791

Q9ULH1 O75791

Q96KM6 O75794

Q15466 O75800

Q9Y3Q8 O75800

P62136 O75807

Q13522 O75807

O15067 O75815

O95197 O75815

P00505 O75815

P00533 O75815

P04626 O75815

P06753 O75815

P07355 O75815

P11940 O75815

P12268 O75815

P15170 O75815

P17812 O75815

P20700 O75815

P21860 O75815

P23526 O75815

P25787 O75815

P26639 O75815

P29692 O75815

P30153 O75815

P33176 O75815

P35637 O75815

P39023 O75815

P49321 O75815

P49755 O75815

P53396 O75815

P55072 O75815

P61160 O75815

P62273 O75815

P62750 O75815

Q01105 O75815

Q01813 O75815

Q13200 O75815

Q13263 O75815

Q15365 O75815

Q15631 O75815

Q8NC51 O75815

Q92945 O75815

Q96AG4 O75815

Q99460 O75815

Q9Y265 O75815

P63162 O75817

Q9BUL9 O75817

Q9H633 O75818

O00571 O75821

O15372 O75821

O75822 O75821

O95257 O75821

O95831 O75821

P54619 O75821

P55884 O75821

Q13347 O75821

Q13526 O75821

Q13573 O75821

Q16637 O75821

Q99459 O75821

Q9P2Y5 O75821

Q9Y6I4 O75821

O15372 O75822

P19784 O75822

P55884 O75822

Q04637 O75822

O95166 O75828

Q9H0R8 O75828

Q9Y6I4 O75828

P01106 O75829

P02647 O75829

Q96EQ0 O75830

Q9NRR5 O75830

Q9UMX0 O75830

O00231 O75832

O00232 O75832

O00487 O75832

O43242 O75832

O60496 O75832

P06400 O75832

P17980 O75832

P35998 O75832

P43358 O75832

P43686 O75832

P51665 O75832

P54578 O75832

P55036 O75832

P62191 O75832

P62195 O75832

P62333 O75832

P62807 O75832

Q13200 O75832

Q15008 O75832

Q99460 O75832

Q9BRP4 O75832

Q9NWV8 O75832

Q9Y5K5 O75832

Q9Y617 O75832

O60341 O75843

Q15276 O75843

Q96LA8 O75843

Q9H832 O75843

P08473 O75844

P62136 O75864

O43617 O75865

P49639 O75865

Q96QF0 O75865

O75874 O75874

Q8IYX8 O75874

Q9UKY1 O75874

Q9UJS0 O75880

O95630 O75886

P00533 O75886

P23458 O75886

P54253 O75886

Q13094 O75886

Q13671 O75886

Q15884 O75886

Q969R5 O75886

Q9NRR5 O75886

Q9Y3C5 O75886

Q8IU85 O75888

O95757 O75896

P50851 O75896

Q562F6 O75896

Q6P2I7 O75896

P46459 O75899

Q99963 O75899

P35754 O75900

P48169 O75907

O95967 O75909

P54253 O75909

Q15834 O75909

Q8HWS3 O75909

Q8N5I3 O75909

Q8N6Y0 O75909

Q8TAP6 O75909

Q93062 O75909

Q9BQ66 O75909

Q9BQY4 O75909

Q9NP66 O75909

Q9Y3Q8 O75909

P40337 O75912

P53350 O75914

P60953 O75914

O60831 O75915

P78362 O75920

O60333 O75923

O75190 O75923

O95295 O75923

P11055 O75923

P12956 O75923

P17027 O75923

P17661 O75923

P20929 O75923

P21675 O75923

P26641 O75923

P35609 O75923

P52179 O75923

P54296 O75923

Q00872 O75923

Q13326 O75923

Q14315 O75923

Q15046 O75923

Q7Z494 O75923

Q8N3K9 O75923

Q8TCU4 O75923

Q8WZ42 O75923

Q99715 O75923

Q9BVP2 O75923

Q9NZB2 O75923

Q9UHW9 O75923

Q9UKG1 O75923

Q9Y3Z3 O75923

O75400 O75925

O75626 O75925

P04637 O75925

P10242 O75925

P16220 O75925

P18031 O75925

P54253 O75925

P62136 O75925

P63279 O75925

Q12772 O75925

Q13485 O75925

Q15797 O75925

Q15843 O75925

Q96RG2 O75925

Q99856 O75925

Q9UIS9 O75925

Q9UKL3 O75925

P01106 O75928

P04637 O75928

P63244 O75928

P63279 O75928

Q12772 O75928

Q15843 O75928

Q8IVL1 O75928

Q8N2W9 O75928

Q92547 O75928

Q96T51 O75928

Q99814 O75928

O95166 O75934

O95758 O75934

P19784 O75934

P38919 O75934

P61326 O75934

Q09472 O75934

Q13573 O75934

Q15427 O75934

Q6P2Q9 O75934

Q99459 O75934

Q9UMS4 O75934

P49841 O75935

P17096 O75937

P78362 O75937

Q15428 O75937

Q96SB4 O75937

O43395 O75940

O75533 O75940

P09661 O75940

P37268 O75940

P39023 O75940

P49137 O75940

Q01844 O75940

Q12874 O75940

Q13099 O75940

Q13435 O75940

Q14684 O75940

Q15393 O75940

Q15427 O75940

Q15428 O75940

Q15811 O75940

Q2NL82 O75940

Q76L83 O75940

Q8N8D1 O75940

Q8TCU6 O75940

Q96JB5 O75940

Q9BQ39 O75940

Q9BVP2 O75940

Q9HC36 O75940

Q9NQ55 O75940

Q9NR30 O75940

Q9Y265 O75940

Q9Y2I6 O75940

Q9Y3B4 O75940

Q9Y4A5 O75940

O95503 O75943

O95931 O75943

Q9UM11 O75943

Q9Y2K6 O75943

P24539 O75947

Q14197 O75947

Q7L5D6 O75947

Q9H845 O75947

O00555 O75953

P21675 O75953

Q01484 O75953

Q5VST9 O75953

Q8WZ42 O75953

Q9Y605 O75953

P21926 O75954

P02751 O75955

P05067 O75955

P05089 O75955

P19320 O75955

P38606 O75955

Q03135 O75955

Q92905 O75955

Q96SB4 O75955

Q9BXC9 O75955

Q9HC96 O75955

P01023 O75956

P05386 O75956

P10074 O75956

P18754 O75956

P32322 O75956

Q13422 O75956

Q13547 O75956

Q14703 O75956

Q16643 O75956

Q92769 O75956

Q9BTC8 O75956

P48059 O75962

Q9NRI5 O75962

Q9NV70 O75962

P24539 O75964

Q14197 O75964

Q9H0R8 O75964

Q9H845 O75964

O95477 O75970

P04637 O75970

P32745 O75970

P34982 O75970

P41595 O75970

P47881 O75970

Q4VCS5 O75970

Q6PJG9 O75970

Q92905 O75970

Q9H205 O75970

Q9HB21 O75970

P46736 O75971

Q13287 O75971

Q16520 O75971

O95544 O76003

O95967 O76003

P02751 O76003

P17027 O76003

P19320 O76003

P28799 O76003

Q04759 O76003

Q6FI81 O76003

Q6UY14 O76003

Q8IYT8 O76003

Q96KM6 O76003

Q9BQ66 O76003

Q9BZL6 O76003

P42566 O76009

P62993 O76009

Q9Y4E8 O76011

A0AUL9 O76013

P40818 O76013

P42566 O76013

P62993 O76013

Q01804 O76013

Q969Q5 O76013

A0JLT2 O76021

O95166 O76021

P03372 O76021

P19320 O76021

P51858 O76021

P60520 O76021

P62633 O76021

P68400 O76021

Q9H0R8 O76021

Q9NV56 O76021

Q9UL18 O76021

Q9UPE1 O76021

P13569 O76024

O60437 O76027

P62993 O76031

Q14197 O76031

Q9NWV8 O76031

Q9UKB1 O76031

Q9Y297 O76031

P00519 O76039

P06241 O76039

P62993 O76039

Q92731 O76041

Q9H204 O76041

Q9HAU4 O76041

P62256 O76050

P21246 O76061

O00401 O76064

O60934 O76064

O76064 O76064

P16104 O76064

P38398 O76064

P62837 O76064

Q00987 O76064

Q12888 O76064

Q13315 O76064

Q13416 O76064

Q14676 O76064

Q14839 O76064

Q15834 O76064

Q9C0F1 O76064

Q9UL42 O76064

A6NEW6 O76070

P17844 O76070

P25205 O76070

P26038 O76070

P63167 O76070

Q96AE4 O76070

O00159 O76071

O15160 O76071

O43795 O76071

O60762 O76071

O75419 O76071

O75449 O76071

O75643 O76071

O76031 O76071

O94832 O76071

P04792 O76071

P10155 O76071

P20618 O76071

P22087 O76071

P25205 O76071

P33992 O76071

P35606 O76071

P46379 O76071

P51784 O76071

P78344 O76071

Q13085 O76071

Q13263 O76071

Q14203 O76071

Q14527 O76071

Q16795 O76071

Q75MH1 O76071

Q7Z7L1 O76071

Q86Y56 O76071

Q8N163 O76071

Q8N1F7 O76071

Q8TEX9 O76071

Q92769 O76071

Q96A35 O76071

Q96M83 O76071

Q96SB4 O76071

Q96T76 O76071

Q9BVP2 O76071

Q9BWH6 O76071

Q9H5X1 O76071

Q9NRH3 O76071

Q9NRZ9 O76071

Q9UHI6 O76071

Q9ULX6 O76071

Q9Y3D0 O76071

Q9Y5Q8 O76071

Q9Y5Q9 O76071

Q9Y5Y2 O76071

O00273 O76075

P35226 O76075

O15269 O76081

O76003 O76081

P00519 O76081

P40222 O76081

P46459 O76081

P48039 O76081

P49286 O76081

Q01105 O76081

Q15126 O76081

Q92504 O76081

Q96CS3 O76081

Q96GS6 O76081

Q9UI30 O76081

Q9Y230 O76081

Q9Y3E2 O76081

O76083 O76083

Q13049 O76083

Q96FC7 O76083

Q9NX58 O76093

O75386 O76094

O94868 O76094

Q13573 O76094

Q14457 O76094

Q99459 O76094

O14965 O76095

O15392 O76095

Q15369 O76095

Q16763 O76095

Q2QGD7 O76095

P53634 O76096

O77932 O77932

Q01780 O77932

Q14254 O77932

Q96LA8 O77932

Q15051 O94760

Q00005 O94761

O43312 O94762

O95402 O94762

O95630 O94762

P16220 O94762

P26641 O94762

P49419 O94762

P53350 O94762

Q13352 O94762

Q7Z465 O94762

Q86XP3 O94762

Q9BTT4 O94762

Q9UQF2 O94762

O43156 O94763

P07900 O94763

P13984 O94763

P17544 O94763

P24928 O94763

P35269 O94763

P36873 O94763

Q6P1J9 O94763

Q8N7H5 O94763

Q9Y265 O94763

P62847 O94768

O60341 O94776

O75323 O94776

P03372 O94776

P49674 O94776

P62805 O94776

Q13547 O94776

Q13573 O94776

Q14839 O94776

Q8IYT8 O94776

Q8N5A5 O94776

Q8NEU8 O94776

Q92731 O94776

Q92769 O94776

Q99459 O94776

Q99497 O94776

Q99576 O94776

Q9BTC8 O94776

Q9UKG1 O94776

P01106 O94779

O60346 O94782

Q6ZVD8 O94782

Q8TAF3 O94782

Q9Y2X8 O94788

O94804 O94804

P04183 O94805

P20073 O94805

P38936 O94805

P49841 O94805

P51531 O94805

P53350 O94805

P67870 O94805

Q16637 O94805

O95989 O94806

P11055 O94806

P21675 O94806

P24386 O94806

P63027 O94806

Q8WVK7 O94806

Q01959 O94811

O94813 O94813

P01106 O94813

O75533 O94817

O75817 O94817

P07814 O94817

P08579 O94817

P11234 O94817

P20700 O94817

P26196 O94817

P42858 O94817

P51991 O94817

P60228 O94817

P62273 O94817

P62807 O94817

Q01804 O94817

Q08188 O94817

Q13151 O94817

Q14152 O94817

Q14457 O94817

Q15323 O94817

Q15459 O94817

Q16540 O94817

Q16543 O94817

Q676U5 O94817

Q6P2I7 O94817

Q7Z6L1 O94817

Q8NC51 O94817

Q8TDY2 O94817

Q96A65 O94817

Q96F87 O94817

Q99729 O94817

Q9BQ66 O94817

Q9BUJ2 O94817

Q9H0Y0 O94817

Q9H1Y0 O94817

Q9H2U1 O94817

Q9HC36 O94817

Q9HCE1 O94817

Q9HCM4 O94817

Q9NSB4 O94817

Q9NT62 O94817

Q9P031 O94817

Q9UH99 O94817

Q9Y3D9 O94817

Q9Y3U8 O94817

Q9Y3Y2 O94817

Q9Y679 O94817

O75971 O94818

Q16659 O94818

Q99873 O94818

P58753 O94822

Q15051 O94822

Q86WV6 O94822

Q92985 O94822

Q9HCC6 O94822

P01106 O94829

P62826 O94829

P63279 O94829

P01106 O94832

P29353 O94832

P38606 O94832

P62136 O94832

Q8TF42 O94832

Q92731 O94832

Q9UGN5 O94832

P16220 O94842

Q13573 O94842

Q96B97 O94850

P02647 O94854

Q02742 O94855

Q15363 O94855

Q15436 O94855

Q15437 O94855

P00519 O94856

P06241 O94856

P21246 O94864

Q9UPT9 O94864

P35790 O94868

P55072 O94868

P78371 O94868

Q15834 O94868

Q5M9N0 O94868

Q8N5S3 O94868

Q96G28 O94868

Q96QZ7 O94868

P49662 O94874

P51965 O94874

Q969T4 O94874

Q96B02 O94874

O43426 O94875

P31749 O94875

P55055 O94875

Q13153 O94875

Q13177 O94875

Q13444 O94875

Q8WX93 O94875

Q92558 O94875

P14921 O94880

P12757 O94885

P31947 O94885

P49674 O94887

P60604 O94887

P55072 O94888

Q16665 O94888

Q8TAT6 O94888

P26641 O94889

Q92905 O94889

O94901 O94901

Q8NF91 O94901

Q8WXH0 O94901

P02751 O94905

Q14197 O94905

Q96DB2 O94905

O43172 O94906

O95166 O94906

O95400 O94906

P01106 O94906

P51531 O94906

P60520 O94906

Q13523 O94906

Q13573 O94906

Q53GS9 O94906

Q8NCN4 O94906

Q96DB2 O94906

Q96DI7 O94906

Q99459 O94906

Q9H492 O94906

P31146 O94907

Q99750 O94907

Q13573 O94913

P31946 O94915

Q9HAT8 O94915

Q9NRI5 O94915

P17844 O94916

Q92841 O94916

P01877 O94921

P27348 O94921

P30281 O94921

P31946 O94921

P38936 O94921

P62258 O94921

Q04917 O94921

Q8ND76 O94921

O94925 O94925

P15336 O94925

P41220 O94927

Q13418 O94927

Q7Z4H7 O94927

Q96CS2 O94927

Q9H6D7 O94927

Q9NVX0 O94927

P33993 O94929

P62993 O94929

P36406 O94941

P51965 O94941

P61077 O94941

P62837 O94941

Q13404 O94941

Q96LR5 O94941

Q9BWF2 O94941

Q9Y4K3 O94941

Q9NP90 O94955

P17948 O94964

Q96L34 O94964

Q9HAU0 O94964

P07900 O94966

P13569 O94966

P27824 O94966

Q6ISU1 O94966

Q93009 O94966

Q6IAM1 O94967

O43663 O94972

O95990 O94972

P51116 O94972

P62256 O94972

Q15398 O94972

Q5VVX9 O94972

Q6ZU52 O94972

Q6ZVK8 O94972

Q7L590 O94972

Q8IYF1 O94972

Q8N5R6 O94972

Q8N720 O94972

Q8TAU3 O94972

Q8TCX5 O94972

Q96EZ8 O94972

Q96KB5 O94972

Q96M61 O94972

Q96T60 O94972

Q9H4K1 O94972

Q9H5Z6 O94972

Q9UBZ4 O94972

P00533 O94973

P02751 O94973

P29353 O94973

P32121 O94973

P42566 O94973

Q15797 O94973

Q96CW1 O94973

O60925 O94979

O75340 O94979

P13805 O94979

P19320 O94979

P54296 O94979

P55735 O94979

Q15436 O94979

O00213 O94985

O60318 O94985

P01106 O94985

Q00987 O94985

Q06710 O94985

Q14669 O94985

Q15047 O94985

Q92547 O94985

Q9H2G2 O94985

Q9P281 O94985

Q13568 O94986

Q9H6J7 O94989

Q9Y3A3 O94989

O60563 O94992

P50750 O94992

Q4G0J3 O94992

O95989 O94993

P08754 O94993

P55259 O94993

Q13145 O94993

Q6PIJ6 O94993

Q8WV16 O94993

Q93034 O94993

Q9NY93 O94993

P19320 O95036

P13569 O95049

Q8NI35 O95049

O75818 O95059

O95059 O95059

O95785 O95059

P01023 O95059

P43246 O95059

P47756 O95059

P51513 O95059

P78346 O95059

Q969H6 O95059

Q96ID5 O95059

Q9UMN6 O95059

O15372 O95065

Q9Y6I4 O95065

P06493 O95067

P14635 O95067

P21333 O95067

P24941 O95067

O75956 O95070

Q9NQ11 O95070

A0JLT2 O95071

O43683 O95071

O94888 O95071

O95402 O95071

P32121 O95071

P51965 O95071

Q04323 O95071

Q70CQ1 O95071

Q8IY92 O95071

Q8IYW5 O95071

Q8TAF3 O95071

Q969T4 O95071

Q96CS3 O95071

Q96DB2 O95071

Q9BPZ3 O95071

O95073 O95073

O15530 O95084

O94761 O95084

P12236 O95084

P34896 O95084

P34932 O95084

P36578 O95084

P42695 O95084

P43250 O95084

P51114 O95084

P52797 O95084

P61769 O95084

P62937 O95084

P63261 O95084

Q6KC79 O95084

Q8NB37 O95084

Q99081 O95084

Q99653 O95084

Q9BUL5 O95084

Q9BVI4 O95084

Q9H0H5 O95084

Q9H0R3 O95084

Q9ULJ1 O95084

Q9Y296 O95084

O75554 O95104

O60749 O95125

Q8WTR7 O95125

O75489 O95139

Q9Y375 O95139

Q16611 O95140

Q9NX47 O95140

Q15796 O95140

Q9NPH2 O95147

O95395 O95149

P09104 O95149

P18621 O95149

P45378 O95149

P50542 O95149

Q13573 O95149

Q6ZN04 O95149

Q9BV68 O95149

Q9UPP5 O95149

O00555 O95153

O95154 O95154

P00533 O95155

P42771 O95155

P54252 O95155

Q04323 O95155

P00519 O95157

P12931 O95157

P16333 O95157

O43318 O95163

O95295 O95163

P01106 O95163

P29353 O95163

P42858 O95163

Q15306 O95163

Q8TE02 O95163

Q96EB1 O95163

Q99459 O95163

Q9H9T3 O95163

O94817 O95166

O95166 O95166

O95210 O95166

O95352 O95166

P10644 O95166

P40692 O95166

P41145 O95166

P60520 O95166

P78362 O95166

Q13188 O95166

Q13501 O95166

Q14151 O95166

Q14596 O95166

Q14677 O95166

Q15459 O95166

Q2TAZ0 O95166

Q32NB8 O95166

Q5VYV7 O95166

Q676U5 O95166

Q86WH2 O95166

Q8IYT8 O95166

Q8NI08 O95166

Q8TC07 O95166

Q8TD19 O95166

Q8WUM4 O95166

Q8WVZ9 O95166

Q8WXU2 O95166

Q96RU3 O95166

Q9BQ83 O95166

Q9BQS8 O95166

Q9BXW4 O95166

Q9GZQ8 O95166

Q9H0R8 O95166

Q9H0S4 O95166

Q9H0Y0 O95166

Q9H492 O95166

Q9NT62 O95166

Q9UPU7 O95166

Q9Y4P1 O95166

P08473 O95168

O43678 O95169

P09669 O95169

P35080 O95169

Q9BVV7 O95169

Q9GZY4 O95169

Q9NR50 O95169

Q9P1W9 O95169

P08237 O95178

Q8TD08 O95180

O43678 O95182

Q99750 O95182

Q9BQ52 O95182

Q9P2P6 O95182

Q9Y478 O95182

O14977 O95190

O95197 O95197

Q8N612 O95197

Q96CV9 O95197

Q9H8W4 O95197

Q9NQC3 O95197

Q53G59 O95198

O95199 O95199

P08238 O95199

Q9BT78 O95199

Q13137 O95201

Q13418 O95201

Q15834 O95201

Q16543 O95201

Q9H7E9 O95201

Q9NRR5 O95201

Q14197 O95202

Q9UHD2 O95202

Q9Y276 O95202

Q9Y297 O95206

O95166 O95210

P60520 O95210

Q9H0R8 O95210

P32121 O95218

P49407 O95218

P78362 O95218

Q53GS9 O95218

Q5S007 O95218

Q96SB4 O95218

P68400 O95218

O00499 O95219

P13569 O95219

P15822 O95219

P54829 O95219

Q93050 O95219

O14777 O95229

O43264 O95229

O43683 O95229

O95229 O95229

Q14457 O95229

Q9H081 O95229

A0JLT2 O95232

O95232 O95232

P03372 O95232

P61981 O95232

P78362 O95232

Q9NX70 O95232

O95218 O95235

Q13573 O95239

Q99459 O95239

P14373 O95243

Q13158 O95243

Q69YH5 O95243

O14788 O95248

Q9NTG7 O95248

O95249 O95249

P08670 O95251

P10275 O95251

P21127 O95251

Q5T3J3 O95251

Q8NHQ1 O95251

Q8WZ19 O95251

Q96BR9 O95251

Q96ED9 O95251

Q9HC52 O95251

Q9P2H0 O95251

Q9Y2I6 O95251

P22736 O95255

P54819 O95255

O00141 O95257

O95865 O95257

P00738 O95257

P13667 O95257

P29622 O95257

P52943 O95257

P68104 O95257

Q12824 O95257

Q15262 O95257

Q15910 O95257

Q504T8 O95257

Q5RL73 O95257

Q5T3J3 O95257

Q5VU43 O95257

Q8IZ69 O95257

Q8N2W9 O95257

Q8NAF0 O95257

Q8NBN7 O95257

Q8TAE8 O95257

Q92993 O95257

Q9BPX5 O95257

Q9BVJ6 O95257

Q9NQ29 O95257

Q9P2H0 O95257

Q9Y383 O95257

Q9Y3C7 O95257

Q9Y6K9 O95257

P62158 O95259

Q15276 O95259

O43463 O95260

P24386 O95260

Q13185 O95260

O15084 O95271

O43303 O95271

O60239 O95271

O95271 O95271

P54274 O95271

Q96RU3 O95271

Q9HC77 O95271

Q9NRI5 O95271

Q9NWT6 O95271

Q9NWV8 O95271

Q9Y2T1 O95271

O75928 O95273

P15923 O95273

P46779 O95273

P52737 O95273

P55042 O95273

Q02363 O95273

Q8TAK6 O95273

Q8TAU3 O95273

Q92905 O95273

Q9HCZ1 O95273

Q9P015 O95273

O95278 O95278

P13807 O95278

Q9UQK1 O95278

Q92837 O95279

P04626 O95292

P17612 O95292

P22694 O95292

P48039 O95292

Q5T4F4 O95292

Q96SU4 O95292

Q9Y2K6 O95292

O00499 O95295

O60282 O95295

O75923 O95295

O94826 O95295

O95163 O95295

O95251 O95295

P07951 O95295

P11047 O95295

P11277 O95295

P12883 O95295

P13929 O95295

P17661 O95295

P24043 O95295

P26641 O95295

P50570 O95295

P54257 O95295

P63104 O95295

P78537 O95295

Q14511 O95295

Q14974 O95295

Q15149 O95295

Q15276 O95295

Q15796 O95295

Q15849 O95295

Q16539 O95295

Q16891 O95295

Q6QNY0 O95295

Q6QNY1 O95295

Q7Z465 O95295

Q8N3K9 O95295

Q92993 O95295

Q969Q1 O95295

Q96AW1 O95295

Q96EV8 O95295

Q96L14 O95295

Q96R06 O95295

Q9BZ29 O95295

Q9H6L5 O95295

Q9NV70 O95295

Q9UJ41 O95295

Q9UL45 O95295

Q9UPN3 O95295

Q9Y561 O95295

O43678 O95298

Q9Y2R0 O95298

O43678 O95299

P08473 O95299

P46736 O95299

Q96DB2 O95299

Q9Y4P8 O95299

O95758 O95319

P02751 O95319

P62993 O95319

Q14210 O95342

O15440 O95347

P01106 O95347

Q13573 O95347

Q15021 O95347

Q5U5Q3 O95347

Q6IBW4 O95347

Q99459 O95347

Q9NRI5 O95347

Q9NTJ3 O95347

O00443 O95352

O94817 O95352

O95166 O95352

O95352 O95352

P14316 O95352

P29692 O95352

P60520 O95352

Q13286 O95352

Q8IY92 O95352

Q9BXW4 O95352

Q9GZQ8 O95352

Q9H0R8 O95352

Q9H0Y0 O95352

Q9H492 O95352

Q9NT62 O95352

P33981 O95359

P08670 O95361

Q01094 O95361

Q15047 O95361

Q14197 O95363

Q70EL2 O95363

Q9UN86 O95363

Q9ULU4 O95365

O60832 O95372

P19320 O95372

Q9Y3C7 O95372

P01106 O95373

P13569 O95373

P19320 O95373

P48059 O95373

P62826 O95373

P62993 O95373

Q70CQ1 O95373

Q8IY92 O95373

Q99459 O95373

A4D1P6 O95376

O15127 O95376

O43526 O95376

O43556 O95376

O43768 O95376

O75828 O95376

P04637 O95376

P14373 O95376

P21246 O95376

P24394 O95376

P26641 O95376

P36957 O95376

P62837 O95376

P62917 O95376

P68036 O95376

Q00987 O95376

Q13404 O95376

Q15382 O95376

Q92993 O95376

Q969T4 O95376

Q96P48 O95376

Q9BVJ6 O95376

Q9BWX1 O95376

Q9HC35 O95376

Q9NPD8 O95376

Q9NRI5 O95376

Q9P2H0 O95376

Q9Y2X8 O95376

P10916 O95377

P35227 O95377

P49841 O95377

Q9C040 O95377

Q12974 O95379

Q14315 O95379

Q93096 O95379

Q9NPJ6 O95379

O43741 O95382

P08238 O95382

P54646 O95382

Q99683 O95382

Q9NYU2 O95388

Q13705 O95390

Q8TEU8 O95390

O60341 O95391

O95816 O95391

P01375 O95391

Q13427 O95391

Q13435 O95391

Q15366 O95391

Q15834 O95391

Q6NYC1 O95391

Q6P2Q9 O95391

Q96LA8 O95391

Q9BZ95 O95391

Q9H2H8 O95391

O14936 O95395

Q96H35 O95395

P61224 O95398

Q13370 O95398

Q5UE93 O95398

O43426 O95400

O43809 O95400

O75643 O95400

O94906 O95400

O95073 O95400

P06729 O95400

P07237 O95400

P09012 O95400

P14678 O95400

P23246 O95400

P28749 O95400

P50053 O95400

P62136 O95400

P62306 O95400

P62308 O95400

P63162 O95400

P81605 O95400

P82279 O95400

Q01844 O95400

Q14240 O95400

Q15029 O95400

Q15182 O95400

Q15427 O95400

Q15428 O95400

Q15637 O95400

Q53GS9 O95400

Q6P2Q9 O95400

Q7Z591 O95400

Q8IUR6 O95400

Q96DG6 O95400

Q96DI7 O95400

Q9BPU5 O95400

Q9BUQ8 O95400

Q9NTZ6 O95400

Q9P2D0 O95400

Q9UHX1 O95400

Q9Y5V3 O95400

A0JLT2 O95402

O95402 O95402

Q96JC9 O95402

Q9BTT4 O95402

Q9BWU1 O95402

Q9NWA0 O95402

Q9NX70 O95402

O00267 O95405

O75694 O95405

P01100 O95405

P08100 O95405

P14923 O95405

P15036 O95405

P36873 O95405

P62136 O95405

P84022 O95405

Q13277 O95405

Q15038 O95405

Q15075 O95405

Q4LDE5 O95405

Q5T124 O95405

Q5TF85 O95405

Q6P3W7 O95405

Q86XL3 O95405

Q8IXI1 O95405

Q9ULU4 O95405

Q9Y561 O95405

Q15796 O95405

P62993 O95411

Q9BTW9 O95424

P13569 O95425

P20929 O95425

P62993 O95425

Q9BY41 O95425

Q9Y383 O95425

O95166 O95429

Q9GZQ8 O95429

Q9H492 O95429

O75365 O95433

P07900 O95433

P13569 O95433

P30793 O95433

P60520 O95433

Q9GZQ8 O95433

Q9GZX7 O95433

Q9P0W5 O95433

Q8WYR1 O95450

P33240 O95453

Q09161 O95453

Q99728 O95453

Q9H0A9 O95453

P16333 O95455

P27986 O95455

P02751 O95456

P01106 O95460

O60861 O95466

O75044 O95466

O75400 O95466

P12956 O95466

P00533 O95470

P04626 O95470

Q15051 O95470

Q86WV6 O95470

Q93099 O95471

Q9BQY4 O95471

Q8N9A8 O95476

Q13424 O95477

Q13425 O95477

Q13884 O95477

Q92796 O95477

Q9NZN5 O95477

Q9Y616 O95478

O75081 O95486

O95166 O95486

Q9H0R8 O95486

O95166 O95487

O95758 O95487

Q15363 O95487

Q15436 O95487

Q9BQS8 O95487

Q9H0R8 O95487

Q9H492 O95487

P16333 O95490

P35226 O95503

P62993 O95544

P63104 O95544

Q04917 O95544

Q6ZVK8 O95544

Q8TBB1 O95544

P12757 O95551

P54253 O95551

P62491 O95551

P78362 O95551

Q03518 O95551

Q06055 O95551

Q99996 O95551

Q9BSF0 O95551

P05386 O95563

P41220 O95568

Q5UIP0 O95571

Q96ID5 O95571

Q9UI12 O95571

P00533 O95573

P04626 O95573

Q86WV6 O95573

Q8N0X7 O95573

Q8NE63 O95573

Q92597 O95573

O95166 O95602

P04626 O95602

P32121 O95602

P49407 O95602

P51784 O95602

Q05048 O95602

Q5UIP0 O95602

Q99728 O95602

Q9H5J8 O95602

Q9H9Y6 O95602

Q9NYV6 O95602

Q9UL18 O95602

P55036 O95613

P67809 O95613

Q99459 O95613

Q9NRI5 O95613

O43257 O95619

O75410 O95619

O96019 O95619

Q15906 O95619

Q99459 O95619

Q9NV56 O95619

Q9UPN9 O95619

Q9Y230 O95619

Q9Y265 O95619

P01106 O95628

P61088 O95628

Q09472 O95628

Q969T4 O95628

A5YKK6 O95628

O43541 O95630

O75886 O95630

P0CG47 O95630

P11712 O95630

P62993 O95630

Q7LBR1 O95630

Q92783 O95630

Q96FW1 O95630

Q9BSD7 O95630

Q9BY43 O95630

Q9HAU4 O95630

Q9HD42 O95630

Q9NZZ3 O95630

Q9UQ90 O95630

Q9Y3C5 O95630

Q9Y3E7 O95630

P43146 O95631

O43184 O95633

P78325 O95633

P06748 O95639

P49760 O95639

Q13573 O95639

Q6UN15 O95639

Q9H6E5 O95639

O15294 O95644

P63104 O95644

P40763 O95661

P60520 O95670

Q15051 O95670

O95671 O95671

O95166 O95678

P60520 O95678

Q9GZQ8 O95678

Q9H0R8 O95678

Q9H492 O95678

P30154 O95684

P62714 O95684

P67775 O95684

O95684 O95684

P06241 O95685

P31947 O95685

P62136 O95685

P08670 O95696

Q9P2H0 O95696

P00533 O95704

P05067 O95704

O95059 O95707

O95707 O95707

P01106 O95707

P78345 O95707

Q969H6 O95707

Q9BUL9 O95707

O94966 O95714

P04637 O95714

P49336 O95714

Q16659 O95714

Q9GZQ8 O95714

Q9H9J4 O95714

Q9NRI5 O95714

Q9NTG7 O95714

Q9UGI0 O95714

Q9Y2K6 O95714

Q9Y5T5 O95714

P61073 O95715

Q04864 O95715

Q53EZ4 O95716

Q9Y2H9 O95716

O95229 O95721

O95758 O95721

P18848 O95721

P19320 O95721

P46459 O95721

Q02833 O95721

Q96LR2 O95721

Q9BVG8 O95721

P04183 O95741

P20073 O95741

Q14451 O95741

Q16637 O95741

Q9UHA4 O95741

O95747 O95747

P55011 O95747

Q13573 O95747

Q96J92 O95747

Q99459 O95747

Q9Y376 O95747

O14639 O95751

O75346 O95751

P01106 O95751

P15622 O95751

P51116 O95751

Q5VWN6 O95751

Q6ZN18 O95751

Q7L190 O95751

Q8TBB1 O95751

Q96PV7 O95751

Q9BTN8 O95751

Q9BXS5 O95751

Q9H9D4 O95751

Q9NVN8 O95751

Q9NY61 O95751

Q9Y247 O95751

P05787 O95757

P60520 O95757

Q9GZQ8 O95757

Q9H492 O95757

Q99459 O95758

P0C0S8 O95760

Q01638 O95760

Q14194 O95760

O75618 O95766

Q99459 O95766

Q9BQS8 O95766

P19419 O95772

O14874 O95777

O43172 O95777

O75643 O95777

P14678 O95777

P19320 O95777

P37837 O95777

P38935 O95777

P42898 O95777

P52209 O95777

P52756 O95777

P62310 O95777

P62312 O95777

P78347 O95777

P83731 O95777

Q01780 O95777

Q13107 O95777

Q15020 O95777

Q16342 O95777

Q2TAY7 O95777

Q53GS9 O95777

Q6WCQ1 O95777

Q86UX7 O95777

Q86Y22 O95777

Q86YB7 O95777

Q8IU57 O95777

Q8IWI9 O95777

Q8N5J2 O95777

Q92900 O95777

Q969T4 O95777

Q96AB3 O95777

Q99719 O95777

Q9BV44 O95777

Q9NPI6 O95777

Q9NZM3 O95777

Q9UBU7 O95777

Q9UK45 O95777

Q9Y333 O95777

Q9Y4E8 O95777

Q9Y4Z0 O95777

Q9Y597 O95777

A9UF07 O95782

O00291 O95782

P00533 O95782

P02751 O95782

P19320 O95782

P29353 O95782

P32121 O95782

P42566 O95782

P46108 O95782

P49757 O95782

P62993 O95782

Q0JRZ9 O95782

Q92731 O95782

Q96CW1 O95782

Q9H492 O95782

O43463 O95785

P18754 O95785

P38936 O95785

P45973 O95785

P46781 O95785

P46783 O95785

Q13526 O95785

Q13573 O95785

Q15714 O95785

Q16637 O95785

Q92769 O95785

Q96KQ7 O95785

Q9H5I1 O95785

O95786 O95786

Q7Z434 O95786

P01100 O95789

Q9P2N7 O95789

P03372 O95793

P07437 O95793

P19525 O95793

P46940 O95793

P51858 O95793

P62136 O95793

P62424 O95793

P62753 O95793

P63000 O95793

P67870 O95793

Q08211 O95793

Q92731 O95793

Q92900 O95793

Q96C10 O95793

Q9HC52 O95793

Q9UPE1 O95793

A0AUL9 O95801

P07900 O95801

Q13428 O95801

P08567 O95810

Q03135 O95810

Q6ZVK8 O95810

Q9H8W4 O95810

A0JLT2 O95816

O43447 O95816

O75061 O95816

O95816 O95816

P02751 O95816

P04637 O95816

P07900 O95816

Q00005 O95816

Q96DB2 O95816

Q9BRX9 O95816

Q9GZX7 O95816

Q9NWA0 O95816

Q9NX70 O95816

Q9Y2T4 O95816

O43187 O95817

P00441 O95817

P10276 O95817

Q13501 O95817

Q15038 O95817

Q4VCS5 O95817

Q8TCX1 O95817

Q9UJY1 O95817

Q9Y4P8 O95817

P08238 O95819

Q00987 O95819

Q16778 O95819

Q9H0R5 O95819

Q9UGI0 O95819

Q9Y5B8 O95819

O75821 O95831

O76061 O95831

O95166 O95831

O95747 O95831

O95831 O95831

P03372 O95831

P13569 O95831

P16104 O95831

P21860 O95831

P40692 O95831

P62937 O95831

Q13418 O95831

Q14197 O95831

Q15047 O95831

Q63ZY3 O95831

Q8IX03 O95831

Q8N0X7 O95831

Q8NC60 O95831

Q96DB2 O95831

Q96HA7 O95831

Q9GZQ8 O95831

Q9H0R8 O95831

Q9H492 O95831

Q9NX70 O95831

Q9Y3Q8 O95831

Q9Y295 O95832

O60285 O95835

P01106 O95835

P06493 O95835

P46937 O95835

P53667 O95835

P63000 O95835

Q15942 O95835

Q7L9L4 O95835

Q9GZV5 O95835

Q9H8S9 O95835

Q13573 O95837

P10644 O95847

Q00604 O95859

Q9ULV1 O95859

Q15051 O95861

O60341 O95863

P04637 O95863

P09874 O95863

P0CG48 O95863

P18146 O95863

P49841 O95863

P61201 O95863

P68431 O95863

Q00987 O95863

Q6FI27 O95863

Q96NG3 O95863

Q9H0J4 O95863

Q9NRM7 O95863

Q9Y297 O95863

O14788 O95865

P05026 O95865

P11802 O95865

P18754 O95865

P20073 O95865

P38936 O95865

P61224 O95865

Q15418 O95865

Q15714 O95865

Q6P1J9 O95865

Q9UBS4 O95865

P42684 O95866

O43889 O95870

O75391 O95870

P03901 O95870

P09651 O95870

P13164 O95870

P40692 O95870

P48201 O95870

Q15424 O95870

Q8TCT9 O95870

Q96KC8 O95870

Q99942 O95870

Q9BVK8 O95870

Q9H0R3 O95870

Q9UKJ0 O95870

Q15834 O95872

Q9BRK4 O95872

P16104 O95881

P00519 O95886

P06241 O95886

P16333 O95886

P46108 O95886

P62993 O95886

A2RRP1 O95897

O14775 O95897

P14618 O95897

P41567 O95897

P50502 O95897

P62753 O95897

Q13243 O95897

Q9HCK4 O95897

Q14197 O95900

P08238 O95905

P63208 O95905

P35226 O95931

P35227 O95931

P51784 O95931

Q06587 O95931

Q3KNV8 O95931

Q86SE9 O95931

Q93009 O95931

O60925 O95936

O00555 O95967

O15162 O95967

O15265 O95967

O95967 O95967

P15502 O95967

P28300 O95967

P29474 O95967

P54253 O95967

Q8NEC5 O95967

Q8TCX5 O95967

Q96EQ0 O95967

Q96FE5 O95967

Q9BQY4 O95967

Q9BUY5 O95967

Q9H5Z6 O95967

Q9HB75 O95967

Q9NRQ2 O95967

Q9UBX5 O95967

P01024 O95973

O60341 O95983

O94776 O95983

P17844 O95983

Q13547 O95983

Q13573 O95983

Q8N5A5 O95983

Q92769 O95983

Q99497 O95983

Q9BTC8 O95983

Q9UKG1 O95983

Q9UPN9 O95983

Q9BQ66 O95985

Q9UKJ5 O95985

O14737 O95989

P05386 O95989

P13056 O95989

P13196 O95989

P27695 O95989

P45378 O95989

P50542 O95989

P51671 O95989

Q14469 O95989

Q14802 O95989

Q15041 O95989

Q15051 O95989

Q16643 O95989

Q8IWV7 O95989

Q8TB96 O95989

Q8WXK3 O95989

Q9BV68 O95989

Q9H063 O95989

Q9NQW7 O95989

Q9NX63 O95989

Q9UGJ1 O95989

O95751 O95990

O95967 O95990

P10599 O95990

P17812 O95990

P63151 O95990

P81605 O95990

Q8N6Y0 O95990

Q96ED9 O95990

Q9BQ66 O95990

Q9BUH8 O95990

Q9Y2I6 O95990

Q9Y4E8 O95990

P40692 O95994

Q01995 O95994

Q14194 O95994

Q9GZT6 O95994

Q9NPI1 O95994

Q9NRR5 O95994

Q9Y3C7 O95994

O76071 O95995

Q15051 O95995

P67809 O95996

O95997 O95997

O14745 O95999

P20749 O95999

P31749 O95999

P50750 O95999

P62807 O95999

Q04759 O95999

Q07955 O95999

Q14525 O95999

Q14790 O95999

Q96LW7 O95999

Q9BXL7 O95999

Q9H257 O95999

Q9UDY8 O95999

Q9ULZ3 O95999

O43678 O96000

Q8IWF7 O96000

O14582 O96006

O15287 O96006

O60341 O96006

O60504 O96006

P10644 O96006

Q6ZVK8 O96006

Q9GZT8 O96006

Q9HCN4 O96006

Q9NX04 O96006

Q9Y221 O96006

Q9Y4P9 O96006

Q9NS69 O96008

O75192 O96011

P40855 O96011

P42858 O96011

P31947 O96013

P36957 O96013

P60953 O96013

P61981 O96013

P63104 O96013

Q04917 O96013

Q13573 O96013

Q86YP4 O96013

Q92974 O96013

Q96SB4 O96013

Q99459 O96013

Q96FJ2 O96015

O60934 O96017

O96017 O96017

P04637 O96017

P06400 O96017

P18887 O96017

P30304 O96017

P52292 O96017

P53350 O96017

P55072 O96017

P56645 O96017

P62875 O96017

Q00987 O96017

Q13362 O96017

Q15172 O96017

Q15173 O96017

Q16537 O96017

Q96SB4 O96017

Q9NY61 O96017

Q9Y248 O96017

A7MCY6 O96018

P11274 O96018

Q92844 O96018

O43257 O96019

P01106 O96019

P25490 O96019

P45973 O96019

P51532 O96019

Q01844 O96019

Q13573 O96019

Q15906 O96019

Q53TQ3 O96019

Q8NBZ0 O96019

Q92769 O96019

Q92993 O96019

Q96EZ8 O96019

Q99459 O96019

Q9C086 O96019

Q9H981 O96019

Q9NV56 O96019

Q9UPN9 O96019

Q9Y230 O96019

Q9Y265 O96019

Q9Y5K5 O96019

P24941 O96020

P46527 O96020

P49918 O96020

Q00526 O96020

Q08999 O96020

O95568 O96024

P63167 O96024

Q9UMR2 O96024

P10275 O96028

Q00987 O96028

P08574 P00156

P22310 P00167

Q8TAV3 P00167

P01011 P00325

O15264 P00338

P00338 P00338

P02751 P00338

P07195 P00338

P08238 P00338

P11142 P00338

P15336 P00338

P19320 P00338

Q96LR5 P00338

Q9H0R8 P00338

P00352 P00352

P05091 P00352

P02751 P00367

P02763 P00367

P04004 P00367

P42229 P00367

Q00535 P00367

Q13418 P00367

Q14197 P00367

Q99442 P00367

Q8NG27 P00390

P05141 P00395

P09669 P00395

P10809 P00395

Q02221 P00395

Q6UXB4 P00395

Q7Z7K0 P00395

Q96I36 P00395

Q9BVV7 P00395

Q9GZY4 P00395

Q9Y2R0 P00395

P02751 P00403

Q14197 P00403

Q9UJS0 P00403

Q9Y2R0 P00403

Q9UJS0 P00414

Q9Y2R0 P00414

P62847 P00439

O95817 P00441

P00441 P00441

Q13501 P00441

Q13561 P00441

Q8TCX1 P00441

P01857 P00450

P02647 P00450

Q9H492 P00451

Q9UMX0 P00451

O43809 P00488

P04183 P00488

P08567 P00488

P20073 P00488

P38936 P00488

Q05397 P00488

Q99962 P00488

P04637 P00491

P19320 P00491

P51693 P00491

Q5RL73 P00491

Q5UIP0 P00491

Q9GZT6 P00491

Q9UKY1 P00491

O95166 P00492

Q14164 P00492

Q96HA8 P00492

Q9H0R8 P00492

Q9NRG1 P00492

O00585 P00505

P02743 P00505

Q6I9Y2 P00505

Q9H6R6 P00505

O15457 P00519

O43196 P00519

O95758 P00519

P43699 P00519

Q13315 P00519

Q38SD2 P00519

Q7Z434 P00519

O00206 P00533

O00213 P00533

O00459 P00533

O00750 P00533

O14818 P00533

O15511 P00533

O43147 P00533

O60603 P00533

O60674 P00533

O60880 P00533

O75165 P00533

O75368 P00533

O75674 P00533

O75886 P00533

O94875 P00533

O94992 P00533

O95433 P00533

O96018 P00533

P00519 P00533

P00533 P00533

P01133 P00533

P02751 P00533

P03372 P00533

P04075 P00533

P04406 P00533

P04626 P00533

P04792 P00533

P06132 P00533

P06396 P00533

P07332 P00533

P08107 P00533

P08238 P00533

P08575 P00533

P08581 P00533

P09936 P00533

P10599 P00533

P11279 P00533

P12931 P00533

P13693 P00533

P15941 P00533

P16152 P00533

P16333 P00533

P16885 P00533

P16930 P00533

P17931 P00533

P19174 P00533

P20936 P00533

P21291 P00533

P21860 P00533

P22681 P00533

P25098 P00533

P27348 P00533

P27986 P00533

P29353 P00533

P34932 P00533

P35568 P00533

P40763 P00533

P40925 P00533

P41240 P00533

P42224 P00533

P42684 P00533

P43405 P00533

P46108 P00533

P46109 P00533

P49757 P00533

P51451 P00533

P51692 P00533

P51813 P00533

P52306 P00533

P54368 P00533

P60174 P00533

P60520 P00533

P62158 P00533

P62993 P00533

P63104 P00533

P98077 P00533

Q01973 P00533

Q05209 P00533

Q06124 P00533

Q06830 P00533

Q09666 P00533

Q13322 P00533

Q13387 P00533

Q13491 P00533

Q13671 P00533

Q14050 P00533

Q14204 P00533

Q14318 P00533

Q15120 P00533

Q15303 P00533

Q15819 P00533

Q16186 P00533

Q16539 P00533

Q16543 P00533

Q53GZ6 P00533

Q59EJ3 P00533

Q59GR8 P00533

Q6PKX4 P00533

Q7L273 P00533

Q7Z6G8 P00533

Q7Z7G1 P00533

Q8IZV2 P00533

Q8IZW8 P00533

Q8N2Y8 P00533

Q8N4S1 P00533

Q8TDB4 P00533

Q8TF42 P00533

Q92529 P00533

Q92569 P00533

Q92733 P00533

Q92870 P00533

Q96AD5 P00533

Q96B97 P00533

Q96CW1 P00533

Q96D37 P00533

Q99418 P00533

Q99608 P00533

Q99962 P00533

Q9NSE2 P00533

Q9P0L0 P00533

Q9UBN7 P00533

Q9UJ41 P00533

Q9UJM3 P00533

Q9UKG1 P00533

Q9UQC2 P00533

Q9UQF2 P00533

Q9UQQ2 P00533

P08238 P00540

Q9NSC5 P00540

O00443 P00558

O15264 P00558

O43524 P00558

O75323 P00558

P02751 P00558

P04406 P00558

P08238 P00558

P15336 P00558

P16104 P00558

P54619 P00558

P63104 P00558

Q15051 P00558

Q8TDR0 P00558

Q96F24 P00558

Q9BSB4 P00558

Q9BW61 P00558

Q9GZZ9 P00558

Q9H0R8 P00558

Q9NR46 P00558

Q9NRI5 P00558

Q9UGJ0 P00558

P01008 P00734

P02768 P00734

P07204 P00734

P07359 P00734

Q07021 P00734

P00736 P00736

P09871 P00736

O60907 P00738

P02649 P00738

P02768 P00738

P62993 P00738

Q14764 P00738

Q8IY92 P00738

Q9H081 P00738

Q9H3D4 P00738

Q9Y6N6 P00738

O14880 P00742

P16403 P00742

Q9Y478 P00742

P02768 P00746

P01876 P00747

P10114 P00747

P84022 P00747

Q16539 P00747

Q7Z6E9 P00747

P01042 P00748

Q07021 P00748

P01106 P00749

P13196 P00749

P02768 P00751

P15927 P00751

O75928 P00813

Q9H845 P00846

P56937 P00915

Q9H9J4 P00918

O60674 P00966

P00966 P00966

P01106 P00966

P02795 P00966

P40692 P00966

P63104 P00966

Q01850 P00966

Q07021 P01008

P01009 P01009

P08246 P01009

P10114 P01009

P27824 P01009

P34741 P01009

P43307 P01009

Q8IY92 P01009

Q8TAX9 P01009

Q9H081 P01009

O15440 P01011

P20700 P01011

P38606 P01011

Q96KC8 P01011

Q9P1U0 P01011

Q9Y6N6 P01011

Q01844 P01019

P30556 P01019

P42785 P01019

P50052 P01019

O00193 P01023

O14879 P01023

O60502 P01023

P02766 P01023

P10632 P01023

P11172 P01023

P16104 P01023

P29474 P01023

P32119 P01023

P50613 P01023

P55040 P01023

P60953 P01023

P61224 P01023

P62993 P01023

Q13325 P01023

Q16637 P01023

Q16659 P01023

Q6ZW49 P01023

Q86Z02 P01023

Q8IUR0 P01023

Q92599 P01023

Q92947 P01023

Q96QH2 P01023

Q9H3D4 P01023

Q9UQ88 P01023

P01871 P01024

P15529 P01024

Q9Y279 P01024

P08603 P01024

O94762 P01033

P08962 P01033

P24534 P01033

P61956 P01033

Q13011 P01033

P02768 P01034

P04179 P01036

P04179 P01037

P00533 P01040

P21246 P01040

P60520 P01040

Q70EK8 P01040

Q9GZQ8 P01040

P00748 P01042

P46663 P01042

Q07021 P01042

P30411 P01042

O14974 P01100

O60476 P01100

O60636 P01100

P05067 P01100

P05412 P01100

P08708 P01100

P15336 P01100

P19338 P01100

P31946 P01100

P43243 P01100

P46821 P01100

P49802 P01100

P51693 P01100

P52736 P01100

P56385 P01100

P62280 P01100

P63313 P01100

P78332 P01100

Q12874 P01100

Q13427 P01100

Q13823 P01100

Q14320 P01100

Q14353 P01100

Q5JTH9 P01100

Q6U841 P01100

Q8NC51 P01100

Q96AG4 P01100

Q96KN3 P01100

Q96L91 P01100

Q96SB4 P01100

Q99959 P01100

Q9H307 P01100

Q9H501 P01100

Q9NZU7 P01100

Q9UND3 P01100

Q9Y3E7 P01100

Q9Y4G6 P01100

Q9Y5T5 P01100

O004990 P01106

O004991 P01106

O00567 P01106

O00767 P01106

O14617 P01106

O14981 P01106

O15075 P01106

O15111 P01106

O15169 P01106

O15379 P01106

O43795 P01106

O60313 P01106

O60645 P01106

O75643 P01106

O75807 P01106

O75928 P01106

O94906 P01106

O95347 P01106

O95782 P01106

P07910 P01106

P08047 P01106

P08621 P01106

P0CG48 P01106

P11387 P01106

P11802 P01106

P17480 P01106

P17812 P01106

P18283 P01106

P24928 P01106

P25205 P01106

P30876 P01106

P33993 P01106

P35249 P01106

P35251 P01106

P35606 P01106

P42704 P01106

P43243 P01106

P46940 P01106

P48444 P01106

P51532 P01106

P52292 P01106

P52701 P01106

P56192 P01106

P57088 P01106

P57678 P01106

P61244 P01106

P61964 P01106

P62805 P01106

P62913 P01106

P63208 P01106

P98172 P01106

Q02818 P01106

Q03701 P01106

Q06830 P01106

Q07864 P01106

Q12888 P01106

Q13085 P01106

Q13105 P01106

Q13263 P01106

Q13287 P01106

Q13576 P01106

Q14203 P01106

Q14683 P01106

Q14690 P01106

Q14739 P01106

Q14839 P01106

Q15022 P01106

Q15029 P01106

Q15059 P01106

Q15477 P01106

Q15910 P01106

Q29RF7 P01106

Q3KQU3 P01106

Q5SRE5 P01106

Q5SY16 P01106

Q6NXE6 P01106

Q6P2Q9 P01106

Q6UB35 P01106

Q6Y7W6 P01106

Q75T13 P01106

Q7Z4Q2 P01106

Q86XI2 P01106

Q86XR8 P01106

Q8IY37 P01106

Q8N163 P01106

Q8N1F7 P01106

Q8N3U4 P01106

Q8N3Y1 P01106

Q8N6R0 P01106

Q8N6T7 P01106

Q8NG66 P01106

Q8TAD8 P01106

Q8TAQ2 P01106

Q8TCG1 P01106

Q8TDI0 P01106

Q8TED0 P01106

Q8TEX9 P01106

Q8WUM0 P01106

Q92621 P01106

Q92922 P01106

Q92974 P01106

Q93008 P01106

Q96A65 P01106

Q96EB6 P01106

Q96HA7 P01106

Q96P70 P01106

Q96PQ7 P01106

Q96T76 P01106

Q99471 P01106

Q9BQG0 P01106

Q9BX66 P01106

Q9GZR7 P01106

Q9H3U1 P01106

Q9H4L7 P01106

Q9H583 P01106

Q9H8V3 P01106

Q9HAV4 P01106

Q9NSV4 P01106

Q9NTI5 P01106

Q9NTJ3 P01106

Q9NU22 P01106

Q9NV70 P01106

Q9NVI1 P01106

Q9NXA8 P01106

Q9NYY8 P01106

Q9UGJ1 P01106

Q9UHI6 P01106

Q9UI12 P01106

Q9UI26 P01106

Q9UIA9 P01106

Q9UL63 P01106

Q9UNF1 P01106

Q9Y2C2 P01106

Q9Y4A5 P01106

Q9Y4I1 P01106

Q9Y4W2 P01106

Q9Y5L0 P01106

Q9Y5Q9 P01106

Q9Y678 P01106

Q9Y6K9 P01106

O15211 P01111

P25685 P01111

P68104 P01111

O00329 P01112

P04049 P01112

P05783 P01112

P20936 P01112

P42684 P01112

Q07889 P01112

Q12967 P01112

Q13671 P01112

Q7Z569 P01112

Q9UQ13 P01112

O15211 P01116

P50749 P01116

Q12967 P01116

Q5VU43 P01116

Q92633 P01116

Q9GZT9 P01116

Q9H0S4 P01116

P01127 P01127

P04085 P01127

P09619 P01127

P27816 P01130

P30101 P01130

Q8NBP7 P01130

P00533 P01133

P26447 P01133

Q9UBN7 P01133

P00533 P01135

Q5TCQ9 P01135

Q969F2 P01135

P05067 P01137

P07942 P01137

P07996 P01137

P36897 P01137

P37173 P01137

Q03167 P01137

Q12841 P01137

Q14689 P01137

Q8TEU8 P01137

Q96NZ8 P01137

Q9UPU3 P01138

Q9NRR5 P01160

P37288 P01185

P35372 P01210

P01225 P01215

P01233 P01215

P16471 P01236

O00232 P01241

O43242 P01241

O75190 P01241

O75534 P01241

O75937 P01241

P00505 P01241

P01241 P01241

P06748 P01241

P07355 P01241

P07741 P01241

P07910 P01241

P08134 P01241

P09960 P01241

P10912 P01241

P11586 P01241

P14923 P01241

P15924 P01241

P16471 P01241

P17980 P01241

P21266 P01241

P24534 P01241

P24752 P01241

P25788 P01241

P26368 P01241

P27361 P01241

P29401 P01241

P29692 P01241

P30084 P01241

P33316 P01241

P35998 P01241

P39019 P01241

P41091 P01241

P43487 P01241

P47756 P01241

P49591 P01241

P49720 P01241

P50395 P01241

P54819 P01241

P55786 P01241

P56537 P01241

P60981 P01241

P61077 P01241

P61326 P01241

P62158 P01241

P62280 P01241

P62714 P01241

P62807 P01241

P67870 P01241

P99999 P01241

Q00796 P01241

Q01105 P01241

Q01469 P01241

Q02413 P01241

Q13347 P01241

Q13617 P01241

Q15019 P01241

Q15185 P01241

Q15365 P01241

Q15691 P01241

Q16186 P01241

Q16658 P01241

Q16740 P01241

Q75MH1 P01241

Q8N5K1 P01241

Q8WUH6 P01241

Q96DZ1 P01241

Q96FW1 P01241

Q99471 P01241

Q9HB71 P01241

Q9NR30 P01241

Q9UKK9 P01241

Q9UM00 P01241

Q9Y262 P01241

Q9Y3D7 P01241

Q9Y3F4 P01241

P01258 P01258

P23634 P01258

P46379 P01270

Q99689 P01270

P01275 P01275

P27487 P01282

Q9NRR5 P01282

P32241 P01282

P37288 P01282

P27487 P01303

P46379 P01303

Q12884 P01303

Q9UMX0 P01303

P25929 P01303

P49146 P01303

P01308 P01308

P02787 P01350

P01374 P01374

P05162 P01374

A1L162 P01375

P01375 P01375

P13521 P01375

P19438 P01375

P23471 P01375

P62158 P01375

Q08629 P01375

P20333 P01375

P29466 P01583

Q9H0F6 P01583

P01106 P01584

P14778 P01584

Q9NPH3 P01584

Q09472 P01588

Q8IY92 P01602

Q9H0R8 P01602

Q9H1Y0 P01602

Q15051 P01604

P01871 P01614

P01876 P01614

P02647 P01614

P02768 P01614

P02787 P01614

P01876 P01620

P01857 P01700

P01871 P01700

P01876 P01700

P04150 P01730

P06239 P01730

P08183 P01730

P51681 P01730

P60709 P01730

Q96S82 P01730

P06493 P01732

P13760 P01737

P01857 P01764

P01857 P01781

Q00534 P01833

Q9H081 P01833

P01857 P01834

P01861 P01834

P01871 P01834

P02647 P01834

P02768 P01834

P02787 P01834

P40692 P01834

P01848 P01850

P01709 P01857

P01834 P01857

P02768 P01857

P02787 P01857

P19320 P01857

P31994 P01857

Q15051 P01857

P02768 P01859

Q9H0R8 P01859

P49841 P01871

Q06187 P01871

Q16637 P01871

Q16644 P01871

Q8IY92 P01871

Q99856 P01871

Q9H0R8 P01871

P12830 P01876

P25054 P01876

Q8IY92 P01876

Q96JB5 P01876

Q9H1Y0 P01876

O95166 P01889

P42224 P01889

Q9NR80 P01889

P61769 P01891

P00533 P01892

P08473 P01892

P21926 P01892

P30101 P01892

P61769 P01892

Q13501 P01893

P04229 P01903

P13760 P01903

P28067 P01903

P01920 P01909

P04233 P01909

P50613 P01909

P51693 P01909

Q04724 P01909

Q13432 P01909

Q5UIP0 P01909

Q99962 P01909

Q9Y6K9 P01909

P28067 P01911

P01903 P01912

P01909 P01920

P19320 P02008

P19320 P02100

O43405 P02452

O95758 P02452

P17020 P02452

Q9NQ11 P02452

O43405 P02458

P02458 P02458

Q04206 P02458

P02461 P02461

O43405 P02462

P02462 P02462

P08572 P02462

P02489 P02489

P02511 P02489

P04792 P02489

P07315 P02489

P43320 P02489

P02489 P02511

P02511 P02511

P04792 P02511

P05067 P02511

P05813 P02511

P07315 P02511

P20807 P02511

P35222 P02511

P43320 P02511

Q16082 P02511

O00716 P02533

O95166 P02533

P00533 P02533

P01857 P02533

P01876 P02533

P02768 P02533

P11021 P02533

P38919 P02533

P60520 P02533

Q00005 P02533

Q00987 P02533

Q9GZQ8 P02533

Q9H0R8 P02533

Q9H492 P02533

O60341 P02538

P00533 P02538

P01857 P02538

P02768 P02538

P19012 P02538

P19320 P02538

P60520 P02538

Q00005 P02538

Q04695 P02538

Q15834 P02538

Q9H0R8 P02538

Q9Y3C0 P02538

A0JLT2 P02545

O00716 P02545

P01106 P02545

P08473 P02545

P12830 P02545

P13569 P02545

P16104 P02545

P18054 P02545

P19320 P02545

P33993 P02545

P38398 P02545

P62993 P02545

P63104 P02545

Q13155 P02545

Q13573 P02545

Q5S007 P02545

Q5VWP3 P02545

Q71DI3 P02545

Q92993 P02545

Q96Q15 P02545

Q96RG2 P02545

Q99459 P02545

Q99469 P02545

Q9BQS8 P02545

Q9H0R8 P02545

Q9NRM7 P02545

Q9UH99 P02545

P11277 P02549

P22681 P02549

P29353 P02549

P50552 P02549

Q01082 P02549

Q06187 P02549

Q8IZP0 P02549

Q8TF42 P02549

Q9UI08 P02549

P00738 P02647

P01857 P02647

P01871 P02647

P01876 P02647

P02647 P02647

P02768 P02647

P02787 P02647

P05067 P02647

P52333 P02647

Q16659 P02647

Q9H081 P02647

O75069 P02649

P00738 P02649

P01023 P02649

P02768 P02649

P10632 P02649

P19320 P02649

P49768 P02649

P50502 P02649

Q16543 P02649

Q53EL6 P02649

Q8N6T3 P02649

Q96JB6 P02649

Q9BQ95 P02649

Q9H0A6 P02649

O43889 P02652

P02768 P02652

P13569 P02652

P01857 P02654

P01876 P02654

P02647 P02654

P02768 P02654

Q9Y328 P02654

P02768 P02655

P02647 P02656

P02768 P02656

Q8N6T3 P02656

P01857 P02671

P01876 P02671

P02647 P02671

P02768 P02671

Q92876 P02671

Q96RG2 P02671

Q9H081 P02671

P02671 P02675

P04183 P02675

P20073 P02675

P67870 P02675

Q16637 P02675

Q92876 P02675

Q9H081 P02675

P02671 P02679

P04229 P02686

P05067 P02686

P13760 P02686

P28067 P02686

P02724 P02724

G3XAG3 P02730

P00367 P02741

P27361 P02741

P28482 P02741

P31995 P02741

Q15485 P02741

Q9Y6N6 P02741

P62993 P02743

Q92905 P02743

O43765 P02745

O75928 P02745

P02647 P02745

P02746 P02745

Q6Q0C1 P02745

Q9H422 P02745

Q9UHD9 P02745

Q9UMX0 P02745

P02745 P02746

Q9HAV5 P02746

P02647 P02747

P02745 P02747

P02747 P02747

P02749 P02749

P04637 P02749

P60953 P02749

P62993 P02749

Q6UXB4 P02749

Q7Z6E9 P02749

O75582 P02751

P01106 P02751

P01857 P02751

P01871 P02751

P01876 P02751

P02647 P02751

P02751 P02751

P02768 P02751

P02787 P02751

P05106 P02751

P06756 P02751

P08648 P02751

P11684 P02751

P11912 P02751

P21980 P02751

P23142 P02751

P29279 P02751

P40337 P02751

P42229 P02751

P49023 P02751

Q13485 P02751

Q15750 P02751

Q15942 P02751

Q16586 P02751

Q8WZ42 P02751

Q9Y371 P02751

Q9Y6W6 P02751

P07858 P02760

P40763 P02760

P42336 P02760

P62993 P02760

Q92569 P02760

Q9Y4D1 P02760

P05121 P02763

Q9H3D4 P02763

P00519 P02765

P02768 P02765

P15336 P02765

P31749 P02765

P60953 P02765

P62993 P02765

O43681 P02766

O75529 P02766

O95163 P02766

O95741 P02766

O96006 P02766

P01857 P02766

P01876 P02766

P02647 P02766

P02753 P02766

P02766 P02766

P02768 P02766

P02787 P02766

P08670 P02766

P25713 P02766

P55854 P02766

P60709 P02766

P68104 P02766

Q12873 P02766

Q15047 P02766

Q15109 P02766

Q15572 P02766

Q6UXH1 P02766

Q7Z5J4 P02766

Q96FW1 P02766

Q99689 P02766

Q9BRK5 P02766

Q9BT40 P02766

Q9HB58 P02766

Q9UKR5 P02766

Q9UQB3 P02766

O00716 P02768

O60925 P02768

O95166 P02768

P01857 P02768

P01876 P02768

P02768 P02768

P02786 P02768

P02787 P02768

P49748 P02768

P60520 P02768

Q09019 P02768

Q15051 P02768

Q86T82 P02768

Q8IY92 P02768

Q96RL1 P02768

Q9GZQ8 P02768

Q9H0R8 P02768

Q9H492 P02768

Q9Y383 P02768

Q00005 P02768

O95630 P02771

P62873 P02771

Q6P2C8 P02771

Q99623 P02771

Q9H223 P02771

Q9Y587 P02771

P68133 P02774

P84101 P02774

Q5UIP0 P02774

Q6P1J9 P02774

P02768 P02775

P22736 P02775

P42677 P02775

Q16659 P02775

Q86TA1 P02775

Q9NW61 P02775

Q9NX14 P02775

P08567 P02776

B0I1T2 P02786

O00716 P02786

P02751 P02786

P02786 P02786

P02787 P02786

P03372 P02786

P04626 P02786

P15336 P02786

P19320 P02786

P21926 P02786

P38606 P02786

P61769 P02786

Q30201 P02786

Q7Z3C6 P02786

Q8TCT7 P02786

Q9P0V3 P02786

O95758 P02787

P01106 P02787

P01350 P02787

P01876 P02787

P03372 P02787

P13984 P02787

Q30201 P02787

Q8IY92 P02787

Q9H081 P02787

Q9H0R8 P02787

Q9Y6N6 P02787

P19338 P02788

Q8TD08 P02788

P00367 P02790

P02768 P02790

Q9H3D4 P02790

O15198 P02792

P02792 P02792

P02794 P02792

P10114 P02792

P21246 P02792

P24522 P02792

P43490 P02792

P62993 P02792

Q12852 P02792

Q12962 P02792

Q8IWF7 P02792

Q99816 P02792

P02792 P02794

P0CG48 P02794

P18428 P02794

P30304 P02794

P38606 P02794

P45984 P02794

P51116 P02794

P52292 P02794

P61244 P02794

Q14194 P02794

Q8IWV7 P02794

Q96CV9 P02794

Q9NPI1 P02794

Q9P0W5 P02794

Q9UER7 P02794

Q8TAX7 P02808

O43741 P02810

O95166 P02810

Q8TAX7 P02810

O00165 P03372

O00429 P03372

O14686 P03372

O15054 P03372

O43251 P03372

O94776 P03372

P00533 P03372

P03372 P03372

P12931 P03372

P17844 P03372

P20290 P03372

P27986 P03372

P28065 P03372

P38398 P03372

P42229 P03372

P51532 P03372

P53041 P03372

P55345 P03372

P60660 P03372

P60763 P03372

Q02447 P03372

Q04206 P03372

Q12778 P03372

Q12873 P03372

Q13330 P03372

Q13642 P03372

Q14151 P03372

Q14686 P03372

Q15596 P03372

Q15788 P03372

Q3L8U1 P03372

Q86Y37 P03372

Q8N895 P03372

Q92731 P03372

Q96AQ6 P03372

Q96GM5 P03372

Q99623 P03372

Q99966 P03372

Q9BQ69 P03372

Q9BTC8 P03372

Q9BTK6 P03372

Q9H467 P03372

Q9NPJ4 P03372

Q9Y294 P03372

Q9Y3R0 P03372

Q9Y6C7 P03372

Q9Y6Q9 P03372

P29353 P03372

O43678 P03886

P43490 P03886

Q02878 P03886

Q9Y375 P03886

O43678 P03905

O75489 P03923

Q9BY11 P03928

P13489 P03950

P19883 P03950

P35609 P03950

P57073 P03956

P41161 P03971

Q9NVJ2 P03971

P78347 P04003

Q14161 P04003

P04004 P04004

P06756 P04004

P18084 P04004

P20963 P04004

P33552 P04004

P40429 P04004

P55040 P04004

P78560 P04004

P83916 P04004

Q07021 P04004

Q13162 P04004

Q9H0R8 P04004

Q9Y4P8 P04004

Q9Y603 P04004

Q9Y3M8 P04035

Q9Y5Z9 P04035

P04040 P04040

P05067 P04040

P20340 P04040

Q06124 P04040

Q2T9J0 P04040

Q70EK8 P04040

Q9H0R8 P04040

O43482 P04049

O95848 P04049

P01106 P04049

P01112 P04049

P01116 P04049

P04049 P04049

P05783 P04049

P06400 P04049

P08238 P04049

P08727 P04049

P09619 P04049

P10809 P04049

P11021 P04049

P14618 P04049

P15056 P04049

P25490 P04049

P27348 P04049

P30086 P04049

P30304 P04049

P31327 P04049

P31946 P04049

P36507 P04049

P45983 P04049

P46109 P04049

P49368 P04049

P53805 P04049

P61981 P04049

P62070 P04049

P62714 P04049

P62834 P04049

P63104 P04049

Q02750 P04049

Q04917 P04049

Q12805 P04049

Q13153 P04049

Q13177 P04049

Q13362 P04049

Q13418 P04049

Q16543 P04049

Q3ZCQ8 P04049

Q92934 P04049

Q96KB5 P04049

Q96S96 P04049

Q99708 P04049

Q9NVR2 P04049

Q9UNS2 P04049

P02647 P04053

P01023 P04062

P38606 P04062

P52735 P04066

Q7KZI7 P04066

Q9UK80 P04066

P04183 P04070

P51511 P04070

O00555 P04075

O15264 P04075

P02751 P04075

P04075 P04075

P08238 P04075

P12004 P04075

P15336 P04075

P16104 P04075

P19320 P04075

P40692 P04075

P43351 P04075

P63104 P04075

Q15051 P04075

Q8N2W9 P04075

Q8NCL4 P04075

Q9BXB5 P04075

Q9Y4E8 P04075

O95166 P04080

P13569 P04080

Q6MZP7 P04080

Q9H0R8 P04080

Q9H492 P04080

O00213 P04083

P00533 P04083

P02751 P04083

P04406 P04083

P12956 P04083

P15336 P04083

P17096 P04083

P19320 P04083

P27694 P04083

P60709 P04083

P63104 P04083

Q13501 P04083

Q13546 P04083

Q5W0Q7 P04083

Q7L5N1 P04083

Q99832 P04083

Q9BQ83 P04083

Q9H0R8 P04083

Q9NWV4 P04083

Q9UHP3 P04083

Q9UKR5 P04083

Q9UMN6 P04083

Q9UNN5 P04083

Q9Y600 P04083

Q9Y6K9 P04083

P04085 P04085

P00533 P04114

P31751 P04114

P84022 P04114

Q8N6T3 P04114

P32927 P04141

O00481 P04155

Q6UX41 P04155

Q6UXZ3 P04155

Q96PJ5 P04155

Q9NRR5 P04155

Q9UMX0 P04155

P04156 P04156

P05067 P04156

P07498 P04156

P25788 P04156

P29372 P04156

P46379 P04156

P46695 P04156

P49639 P04156

P61964 P04156

P62380 P04156

P62945 P04156

Q14011 P04156

Q15527 P04156

Q15776 P04156

Q53QZ3 P04156

Q6ZUS6 P04156

Q8NDX6 P04156

Q92913 P04156

Q969X6 P04156

Q96A00 P04156

Q96L34 P04156

Q9BRJ6 P04156

Q9BRP8 P04156

Q9BSJ6 P04156

Q9BWG6 P04156

Q9H4B4 P04156

Q9HCC9 P04156

Q9NPF8 P04156

Q9P013 P04156

Q9UIF8 P04156

Q9UL18 P04156

Q9ULW3 P04156

Q9ULX3 P04156

P04179 P04179

P07237 P04179

P16104 P04179

P51531 P04179

Q13029 P04179

Q14789 P04179

Q9GZU8 P04179

Q9UHQ7 P04179

O95989 P04181

P01106 P04181

P15336 P04181

Q01804 P04181

Q09019 P04181

Q14197 P04181

Q8N357 P04181

Q9H492 P04181

B4DNA4 P04183

O15054 P04183

O15442 P04183

O43681 P04183

O60383 P04183

O75037 P04183

O75821 P04183

O95785 P04183

O95865 P04183

P01023 P04183

P01871 P04183

P02751 P04183

P02768 P04183

P04183 P04183

P04217 P04183

P04406 P04183

P04637 P04183

P05060 P04183

P05452 P04183

P06576 P04183

P08138 P04183

P08572 P04183

P11802 P04183

P11926 P04183

P13196 P04183

P13942 P04183

P14618 P04183

P19793 P04183

P19883 P04183

P26373 P04183

P27694 P04183

P29400 P04183

P29597 P04183

P35611 P04183

P38606 P04183

P42025 P04183

P43246 P04183

P47897 P04183

P49815 P04183

P50224 P04183

P51693 P04183

P51805 P04183

P55854 P04183

P60510 P04183

P68104 P04183

Q00013 P04183

Q04724 P04183

Q05516 P04183

Q09013 P04183

Q09028 P04183

Q12766 P04183

Q13107 P04183

Q13432 P04183

Q13596 P04183

Q13885 P04183

Q14194 P04183

Q14240 P04183

Q14919 P04183

Q15047 P04183

Q15262 P04183

Q15572 P04183

Q15784 P04183

Q15811 P04183

Q15910 P04183

Q2QGD7 P04183

Q5D0E6 P04183

Q5R3I4 P04183

Q5RL73 P04183

Q5TA45 P04183

Q5UIP0 P04183

Q5VU43 P04183

Q6IE81 P04183

Q6P0Q8 P04183

Q6P4I2 P04183

Q6UXD5 P04183

Q75N90 P04183

Q7L5N1 P04183

Q7Z4K8 P04183

Q86XA0 P04183

Q8IV08 P04183

Q8IVF5 P04183

Q8IXL6 P04183

Q8IY92 P04183

Q8N6T3 P04183

Q8NBE8 P04183

Q8NFF5 P04183

Q8TB40 P04183

Q8TF32 P04183

Q8WW35 P04183

Q93063 P04183

Q96EN9 P04183

Q96J01 P04183

Q96N67 P04183

Q96NT0 P04183

Q96PQ7 P04183

Q9BZE1 P04183

Q9GZT6 P04183

Q9H324 P04183

Q9NPB8 P04183

Q9NV06 P04183

Q9NYF0 P04183

Q9P283 P04183

Q9UKR5 P04183

Q9UPQ3 P04183

Q9Y265 P04183

Q9Y2V2 P04183

Q9Y3C7 P04183

Q9Y600 P04183

Q9Y605 P04183

O15162 P04196

P02751 P04196

Q96PM5 P04196

Q9H2X0 P04196

O14965 P04198

P61244 P04198

Q13287 P04198

Q8N726 P04198

Q92769 P04198

Q969H0 P04198

Q96EB6 P04198

O95255 P04217

P20073 P04217

P37840 P04217

Q8WTS6 P04217

P08473 P04229

P21926 P04229

P28067 P04229

P01903 P04233

P28067 P04233

P61073 P04233

P04150 P04234

P02768 P04259

P08727 P04259

P19012 P04259

P60520 P04259

Q00005 P04259

Q9H0R8 P04259

O00716 P04264

O95166 P04264

P00533 P04264

P01857 P04264

P01871 P04264

P01876 P04264

P02647 P04264

P02751 P04264

P02768 P04264

P12830 P04264

P19320 P04264

P25054 P04264

P27348 P04264

P38919 P04264

P60520 P04264

Q00005 P04264

Q15759 P04264

Q9GZQ8 P04264

Q9H0R8 P04264

Q9H492 P04264

P04271 P04271

P04637 P04271

P06703 P04271

P23297 P04271

P31949 P04271

Q00987 P04271

Q15109 P04271

Q6NSH3 P04271

Q969U6 P04271

O00555 P04275

O15265 P04275

P04275 P04275

P06756 P04275

P07359 P04275

P63000 P04275

Q76LX8 P04275

Q8WXH5 P04275

O00459 P04279

Q9NRR5 P04279

O75381 P04350

O95166 P04350

P02787 P04350

P18858 P04350

P25963 P04350

P26641 P04350

P32121 P04350

Q01101 P04350

Q07157 P04350

Q07666 P04350

Q13084 P04350

Q13838 P04350

Q14155 P04350

Q15012 P04350

Q68CR1 P04350

Q8WVB3 P04350

Q8WWQ0 P04350

Q969W0 P04350

Q969X0 P04350

Q9H9G7 P04350

Q9P1Y5 P04350

Q9Y6N7 P04350

O15264 P04406

O60861 P04406

O95166 P04406

P00505 P04406

P00533 P04406

P00558 P04406

P01023 P04406

P03372 P04406

P04406 P04406

P08238 P04406

P12004 P04406

P15336 P04406

P15927 P04406

P19320 P04406

P24522 P04406

P28482 P04406

P32121 P04406

P37840 P04406

P42771 P04406

P43351 P04406

P48637 P04406

P49841 P04406

P52756 P04406

P60520 P04406

P60709 P04406

P62258 P04406

P63104 P04406

P68032 P04406

Q00537 P04406

Q06187 P04406

Q07820 P04406

Q13164 P04406

Q13268 P04406

Q14469 P04406

Q15051 P04406

Q16637 P04406

Q92993 P04406

Q96Q15 P04406

Q99459 P04406

Q99650 P04406

Q9GZQ8 P04406

Q9H0J4 P04406

Q9H0R8 P04406

Q9H492 P04406

Q9Y6H6 P04406

P04424 P04424

P09467 P04424

P19784 P04424

Q12889 P04424

Q96AW5 P04424

P40763 P04439

Q9NQC3 P04440

O00213 P04626

O00459 P04626

O00750 P04626

O14492 P04626

O14980 P04626

O43157 P04626

O75367 P04626

O75553 P04626

O75815 P04626

O95602 P04626

O95704 P04626

O95980 P04626

P00519 P04626

P00533 P04626

P04626 P04626

P06401 P04626

P08238 P04626

P08575 P04626

P09769 P04626

P12931 P04626

P15882 P04626

P16070 P04626

P19174 P04626

P20936 P04626

P21860 P04626

P23458 P04626

P23467 P04626

P23471 P04626

P27986 P04626

P29353 P04626

P35568 P04626

P40763 P04626

P42224 P04626

P42566 P04626

P42679 P04626

P42680 P04626

P42681 P04626

P42684 P04626

P43405 P04626

P46109 P04626

P46940 P04626

P49792 P04626

P51451 P04626

P51813 P04626

P60709 P04626

P62993 P04626

P78314 P04626

P98077 P04626

P98172 P04626

Q05209 P04626

Q05397 P04626

Q08881 P04626

Q12913 P04626

Q13239 P04626

Q13387 P04626

Q13671 P04626

Q14451 P04626

Q14974 P04626

Q15075 P04626

Q15262 P04626

Q15303 P04626

Q16543 P04626

Q16827 P04626

Q63HR2 P04626

Q6ZV89 P04626

Q7Z6G8 P04626

Q7Z7G1 P04626

Q8TEW6 P04626

Q8WYP3 P04626

Q92569 P04626

Q92625 P04626

Q96D37 P04626

Q99704 P04626

Q9H299 P04626

Q9H6Q3 P04626

Q9NP31 P04626

Q9NSE2 P04626

Q9UBN7 P04626

Q9UJM3 P04626

Q9UKW4 P04626

Q9UQF2 P04626

Q9UQQ2 P04626

Q9Y316 P04626

Q9Y490 P04626

P01138 P04629

P04629 P04629

P08238 P04629

P18031 P04629

P19174 P04629

P22681 P04629

P27986 P04629

P29353 P04629

Q05209 P04629

Q12913 P04629

Q16827 P04629

P07384 P04632

P13569 P04632

P17655 P04632

P19320 P04632

P20936 P04632

P40763 P04632

O14503 P04637

O14576 P04637

O14757 P04637

O14920 P04637

O15151 P04637

O15169 P04637

O15379 P04637

O43524 P04637

O43847 P04637

O60285 P04637

O75925 P04637

O75970 P04637

O95758 P04637

O95863 P04637

O96017 P04637

P01106 P04637

P04183 P04637

P04271 P04637

P04637 P04637

P04731 P04637

P06400 P04637

P06703 P04637

P06748 P04637

P08047 P04637

P08238 P04637

P09874 P04637

P0CG48 P04637

P10275 P04637

P10415 P04637

P11473 P04637

P12956 P04637

P13497 P04637

P13693 P04637

P17676 P04637

P17844 P04637

P18283 P04637

P18754 P04637

P18887 P04637

P20226 P04637

P22736 P04637

P23297 P04637

P23511 P04637

P24522 P04637

P25208 P04637

P26447 P04637

P27694 P04637

P29034 P04637

P29590 P04637

P30405 P04637

P30419 P04637

P31947 P04637

P32780 P04637

P35232 P04637

P36956 P04637

P38398 P04637

P38646 P04637

P38936 P04637

P42345 P04637

P42858 P04637

P43356 P04637

P45983 P04637

P46821 P04637

P49761 P04637

P49841 P04637

P51784 P04637

P51813 P04637

P53350 P04637

P55060 P04637

P61224 P04637

P61289 P04637

P61978 P04637

P61981 P04637

P62081 P04637

P63151 P04637

P63279 P04637

P68400 P04637

Q00987 P04637

Q01484 P04637

Q05086 P04637

Q05397 P04637

Q06609 P04637

Q07817 P04637

Q09472 P04637

Q12772 P04637

Q12888 P04637

Q13263 P04637

Q13315 P04637

Q13362 P04637

Q13526 P04637

Q13547 P04637

Q13625 P04637

Q13972 P04637

Q14151 P04637

Q14191 P04637

Q14695 P04637

Q14999 P04637

Q15424 P04637

Q15466 P04637

Q15486 P04637

Q15648 P04637

Q15672 P04637

Q15759 P04637

Q15796 P04637

Q15831 P04637

Q16637 P04637

Q16666 P04637

Q53GS9 P04637

Q7L7W2 P04637

Q7Z2E3 P04637

Q7Z6Z7 P04637

Q86TM6 P04637

Q86XK2 P04637

Q86Z02 P04637

Q8IWT3 P04637

Q8IZD2 P04637

Q8N2W9 P04637

Q8N488 P04637

Q8N726 P04637

Q8N9B5 P04637

Q8N9N5 P04637

Q8NHY2 P04637

Q8TAF3 P04637

Q8TAQ5 P04637

Q8TDN4 P04637

Q8TDY2 P04637

Q8TEW0 P04637

Q8WTS6 P04637

Q8WUF5 P04637

Q92841 P04637

Q92890 P04637

Q93009 P04637

Q96EB6 P04637

Q96FW1 P04637

Q96G74 P04637

Q96GM8 P04637

Q96KB5 P04637

Q96KQ4 P04637

Q96M61 P04637

Q96PM5 P04637

Q96PM9 P04637

Q96PU4 P04637

Q96SB4 P04637

Q96ST3 P04637

Q99966 P04637

Q99986 P04637

Q9BQA5 P04637

Q9BUJ2 P04637

Q9BVP2 P04637

Q9BWC9 P04637

Q9BX70 P04637

Q9BY41 P04637

Q9H3D4 P04637

Q9H422 P04637

Q9H7Z6 P04637

Q9H9J4 P04637

Q9HBJ7 P04637

Q9NPI1 P04637

Q9NVN8 P04637

Q9NZC7 P04637

Q9UBE0 P04637

Q9UBF1 P04637

Q9UER7 P04637

Q9UHC7 P04637

Q9UN74 P04637

Q9UPZ9 P04637

Q9Y3T9 P04637

P55209 P04637

Q9H160 P04637

P63096 P04731

P04179 P04745

O43715 P04746

Q00534 P04746

O94782 P04792

O95758 P04792

P00488 P04792

P00533 P04792

P01106 P04792

P02511 P04792

P04637 P04792

P04792 P04792

P08473 P04792

P11413 P04792

P13569 P04792

P15336 P04792

P18428 P04792

P19320 P04792

P31749 P04792

P35813 P04792

P43320 P04792

P60520 P04792

P62993 P04792

P63104 P04792

Q04637 P04792

Q09019 P04792

Q13573 P04792

Q15029 P04792

Q15051 P04792

Q15181 P04792

Q16644 P04792

Q5RL73 P04792

Q5UIP0 P04792

Q8IY92 P04792

Q8NB14 P04792

Q96ID5 P04792

Q96RG2 P04792

Q99459 P04792

Q9BRX9 P04792

Q9GZQ8 P04792

Q9H0R8 P04792

Q9H492 P04792

Q9UER7 P04792

Q9UHV2 P04792

Q9UKG1 P04792

P19320 P04818

Q99962 P04818

P01106 P04843

P02751 P04843

P03372 P04843

P15336 P04843

P19320 P04843

Q09028 P04843

Q14695 P04843

Q5VYV7 P04843

Q70CQ1 P04843

Q8TCJ2 P04843

Q92782 P04843

Q9H0R8 P04843

Q9NRR5 P04843

Q9NYS7 P04843

P01106 P04844

P03372 P04844

P16104 P04844

P27824 P04844

P32121 P04844

P61586 P04844

Q13286 P04844

Q75N90 P04844

Q96PM5 P04844

Q9NXR7 P04844

O15162 P04899

P06748 P04899

P08238 P04899

P13569 P04899

P14598 P04899

P19878 P04899

P32302 P04899

P48039 P04899

P49286 P04899

P49795 P04899

P60709 P04899

P62873 P04899

P68366 P04899

Q99750 P04899

O95166 P04908

O95503 P04908

P01106 P04908

P60520 P04908

P83916 P04908

Q14781 P04908

Q16637 P04908

Q96HA7 P04908

Q9H0R8 P04908

Q9HC52 P04908

Q9HCK5 P04908

Q9UKV8 P04908

Q6MZP7 P04920

O00716 P05023

O95295 P05023

P00533 P05023

P01106 P05023

P04626 P05023

P13693 P05023

P13984 P05023

P15336 P05023

P31152 P05023

Q15051 P05023

Q15628 P05023

Q15796 P05023

Q8IZL9 P05023

Q8NE63 P05023

Q9H492 P05023

Q9NR20 P05023

Q9UL18 P05023

P00533 P05026

P04150 P05026

Q5UIP0 P05026

Q8IZ69 P05026

Q9UMN6 P05026

P18754 P05060

P20073 P05060

P38936 P05060

Q15796 P05060

P04075 P05062

Q8IWZ6 P05062

Q8N307 P05062

Q8NFJ9 P05062

Q96RK4 P05062

Q99757 P05062

Q9BXC9 P05062

Q9BYV1 P05062

O00213 P05067

O14879 P05067

O43399 P05067

O75509 P05067

O75955 P05067

P00519 P05067

P01023 P05067

P01100 P05067

P02647 P05067

P05067 P05067

P05412 P05067

P07339 P05067

P08138 P05067

P10636 P05067

P29353 P05067

P30101 P05067

P49768 P05067

Q00987 P05067

Q02410 P05067

Q13526 P05067

Q13951 P05067

Q8TAG9 P05067

Q93074 P05067

Q96QH2 P05067

Q99714 P05067

Q9H0A6 P05067

Q9NP59 P05067

Q9NPJ6 P05067

Q9Y6Q5 P05067

O43736 P05067

Q06481 P05067

Q07954 P05067

Q13867 P05067

Q8IWV2 P05067

Q9HCB6 P05067

Q9NSC5 P05067

O75069 P05067

P03897 P05067

P04040 P05067

P07996 P05067

P39060 P05067

Q5JRX3 P05067

O95166 P05089

P01106 P05089

P03372 P05089

P30038 P05089

P60520 P05089

Q70EK8 P05089

Q9BSB4 P05089

P01023 P05090

P03372 P05090

Q6EER8 P05090

Q9BWU1 P05090

P00352 P05091

P49789 P05091

Q14197 P05091

Q8N357 P05091

Q96ID5 P05091

Q9GZZ9 P05091

Q9H0R8 P05091

Q9NTG7 P05091

Q9UKR5 P05091

P08238 P05093

P56381 P05093

Q86UA6 P05093

Q9HBU1 P05093

O14924 P05106

P05106 P05106

P06756 P05106

P07948 P05106

P08514 P05106

P18031 P05106

P23471 P05106

Q12929 P05106

Q7Z6G8 P05106

Q9UBP9 P05106

Q9UQF2 P05106

Q9Y490 P05106

P00519 P05107

P15498 P05107

P20701 P05107

P35241 P05107

Q13349 P05107

Q15438 P05107

Q96S59 P05107

Q9UNS2 P05107

Q9Y4G6 P05107

P01023 P05108

O60383 P05109

P03372 P05109

P04637 P05109

P05060 P05109

P06702 P05109

P17020 P05109

P19320 P05109

P29353 P05109

P38936 P05109

P62937 P05109

P62993 P05109

P68366 P05109

Q00005 P05109

Q09019 P05109

Q14690 P05109

Q5UIP0 P05109

Q8IY92 P05109

Q8TDR0 P05109

Q8TF42 P05109

Q96ID5 P05109

Q99873 P05109

Q9BQ83 P05109

Q9H0B6 P05109

Q9H0R8 P05109

Q9H0T7 P05109

Q9UKR5 P05109

P24394 P05112

P31785 P05112

P78552 P05112

P32927 P05113

Q01344 P05113

Q9H0R8 P05120

O95758 P05121

P00749 P05121

P00750 P05121

P02763 P05121

P04004 P05121

Q9NRR5 P05121

P08238 P05129

Q86UR1 P05129

Q8TD31 P05129

Q9NRD5 P05129

O00257 P05141

O00716 P05141

O95166 P05141

P00533 P05141

P01023 P05141

P03372 P05141

P13489 P05141

P19320 P05141

P51858 P05141

P60520 P05141

P61326 P05141

P62993 P05141

Q13131 P05141

Q14197 P05141

Q15796 P05141

Q5S007 P05141

Q5U5Q3 P05141

Q8N357 P05141

Q96HA7 P05141

Q9GZQ8 P05141

Q9H0R8 P05141

Q9H492 P05141

Q9HCK5 P05141

Q9UKV8 P05141

Q9UL18 P05141

Q9Y6E7 P05141

Q09472 P05154

Q7L5N1 P05154

Q8N2W9 P05154

P02768 P05155

P22736 P05155

O75369 P05161

O75688 P05161

Q9UMW8 P05161

P01023 P05164

P09914 P05165

Q00005 P05165

Q9UGN5 P05165

O15357 P05166

P09914 P05166

Q00005 P05166

Q14197 P05166

O15525 P05181

P10909 P05181

P30084 P05181

Q13287 P05181

Q8WWZ7 P05181

Q9BYV1 P05181

Q9Y6N6 P05181

Q14240 P05186

O43292 P05187

P01023 P05187

P55854 P05187

P63165 P05187

Q92643 P05187

B2Y833 P05198

O00571 P05198

P19320 P05198

P19525 P05198

P20042 P05198

P36873 P05198

P41091 P05198

P43146 P05198

P51858 P05198

Q13573 P05198

Q9UGN5 P05204

P05230 P05230

P11362 P05230

P21802 P05230

P22607 P05230

Q96SB4 P05230

Q05516 P05231

Q99962 P05231

P24530 P05305

P25101 P05305

P46379 P05305

Q7L5N1 P05305

Q9NRR5 P05305

P00533 P05362

P0CG48 P05362

P20701 P05362

O00193 P05386

O95166 P05386

O95989 P05386

P09913 P05386

P09914 P05386

P12956 P05386

P15927 P05386

P21246 P05386

P32121 P05386

P60520 P05386

P60709 P05386

P61077 P05386

P62714 P05386

P68104 P05386

Q00005 P05386

Q13885 P05386

Q5UIP0 P05386

Q96GY3 P05386

Q96PM5 P05386

Q9GZQ8 P05386

Q9H0R8 P05386

Q9H492 P05386

Q9H840 P05386

Q9UK32 P05386

O15264 P05387

O95166 P05387

P02751 P05387

P16104 P05387

P32121 P05387

P60520 P05387

P62993 P05387

P63104 P05387

Q00005 P05387

Q06710 P05387

Q6MZP7 P05387

Q9GZQ8 P05387

Q9H0R8 P05387

Q9H492 P05387

Q9UQ80 P05387

B2Y833 P05388

O60861 P05388

O95166 P05388

O95793 P05388

P03372 P05388

P08238 P05388

P19320 P05388

P32121 P05388

P35813 P05388

P60520 P05388

P60709 P05388

P63104 P05388

Q00005 P05388

Q13573 P05388

Q8WYK2 P05388

Q92731 P05388

Q99459 P05388

Q9GZQ8 P05388

Q9H0R8 P05388

Q9H3D4 P05388

Q9H492 P05388

Q9H4L4 P05388

Q9UKV8 P05388

Q9UL18 P05388

Q9UQ80 P05388

Q07666 P05408

Q9NRR5 P05408

O00533 P05412

O43889 P05412

P01100 P05412

P01106 P05412

P05067 P05412

P07437 P05412

P07900 P05412

P0CG48 P05412

P14921 P05412

P15336 P05412

P18847 P05412

P18848 P05412

P28562 P05412

P45983 P05412

P45984 P05412

P48634 P05412

P51858 P05412

P52292 P05412

P53779 P05412

P67775 P05412

P98082 P05412

Q00005 P05412

Q00987 P05412

Q06481 P05412

Q13951 P05412

Q15751 P05412

Q71U36 P05412

Q8WUY8 P05412

Q8WYK2 P05412

Q96ED9 P05412

Q99966 P05412

Q99986 P05412

Q9NR30 P05412

Q9NRL3 P05412

Q9UPY8 P05412

P05556 P05413

P08648 P05413

P16144 P05413

P22736 P05413

P53350 P05413

P56199 P05413

Q59H18 P05413

Q15759 P05423

Q15796 P05423

Q8NCN4 P05423

Q99459 P05423

P05783 P05452

P20073 P05452

P23443 P05452

P38936 P05452

Q92882 P05452

Q99962 P05452

Q9UK32 P05452

Q9Y2W1 P05452

O14879 P05455

O95166 P05455

O95503 P05455

P02751 P05455

P04150 P05455

P17096 P05455

P19525 P05455

P60520 P05455

P63104 P05455

Q01892 P05455

Q9GZQ8 P05455

Q9H0R8 P05455

Q9P0W5 P05455

P01857 P05546

P01876 P05546

P02787 P05546

P06748 P05549

P62993 P05549

P63279 P05549

Q09472 P05549

O00716 P05556

O14713 P05556

O75578 P05556

P02751 P05556

P05413 P05556

P05556 P05556

P07948 P05556

P08648 P05556

P08962 P05556

P13612 P05556

P15336 P05556

P17301 P05556

P17813 P05556

P18031 P05556

P19320 P05556

P20339 P05556

P21333 P05556

P21926 P05556

P23229 P05556

P30101 P05556

P32927 P05556

P39060 P05556

P49023 P05556

P56199 P05556

Q13683 P05556

Q14192 P05556

Q9BY76 P05556

Q9H0F6 P05556

Q9UL25 P05556

Q9Y490 P05556

Q12929 P05556

Q7Z6G8 P05556

P02751 P05771

Q05655 P05771

O14964 P05783

O43913 P05783

O75190 P05783

O95751 P05783

P02671 P05783

P02751 P05783

P04049 P05783

P05787 P05783

P13569 P05783

P15336 P05783

P19320 P05783

P27348 P05783

P52292 P05783

P62993 P05783

P63104 P05783

Q12815 P05783

Q13895 P05783

Q14161 P05783

Q15628 P05783

Q15834 P05783

Q6PKC3 P05783

Q8IWZ6 P05783

Q8IYI6 P05783

Q8NFA0 P05783

Q8NFJ9 P05783

Q92837 P05783

Q96MU7 P05783

Q99757 P05783

Q9BVR6 P05783

Q9BXC9 P05783

Q9NUX5 P05783

Q9Y5B8 P05783

Q9Y6K9 P05783

O75190 P05787

O75503 P05787

P04049 P05787

P05783 P05787

P11532 P05787

P13569 P05787

P15336 P05787

P19320 P05787

P62993 P05787

P63104 P05787

Q01804 P05787

Q07157 P05787

Q12815 P05787

Q13895 P05787

Q8NFA0 P05787

Q9H0R6 P05787

Q9Y6K9 P05787

P05813 P05813

P53674 P05813

P32121 P05814

P49407 P05814

P60953 P05814

P62993 P05814

P63000 P05814

Q13952 P05814

P42574 P05937

Q15051 P05937

Q9H1D0 P05937

Q9NQA5 P05937

Q9Y6K9 P05937

Q15327 P05976

P12107 P05997

P20908 P05997

O75907 P06126

P21854 P06127

Q12873 P06132

Q9UGU5 P06132

Q9UNN5 P06132

P42229 P06133

P17706 P06213

P18031 P06213

P19174 P06213

P20936 P06213

P23470 P06213

P27986 P06213

P28347 P06213

P29353 P06213

P35568 P06213

P46108 P06213

P46109 P06213

P51692 P06213

P62745 P06213

Q06124 P06213

Q13257 P06213

Q13322 P06213

Q14449 P06213

Q15262 P06213

Q99490 P06213

Q9BX66 P06213

Q9NRF2 P06213

Q9Y4H2 P06213

O43561 P06239

O60880 P06239

P01730 P06239

P04150 P06239

P06239 P06239

P06729 P06239

P07766 P06239

P07900 P06239

P08238 P06239

P08575 P06239

P0CG48 P06239

P15498 P06239

P20749 P06239

P20963 P06239

P22681 P06239

P23467 P06239

P27986 P06239

P28827 P06239

P29350 P06239

P42768 P06239

P43403 P06239

P43405 P06239

P60709 P06239

P60953 P06239

P67870 P06239

P78314 P06239

Q04759 P06239

Q07666 P06239

Q12913 P06239

Q13094 P06239

Q13283 P06239

Q13444 P06239

Q15262 P06239

Q16827 P06239

Q71SY5 P06239

Q8IZP0 P06239

Q9H204 P06239

Q9NP31 P06239

Q9Y2R2 P06239

A7KAX9 P06241

O14559 P06241

O14974 P06241

O15085 P06241

O15117 P06241

O43150 P06241

O43390 P06241

O43463 P06241

O43516 P06241

O43909 P06241

O43918 P06241

O60341 P06241

O60880 P06241

P00519 P06241

P00540 P06241

P02751 P06241

P06241 P06241

P06729 P06241

P08047 P06241

P08238 P06241

P09619 P06241

P10301 P06241

P10636 P06241

P11137 P06241

P12931 P06241

P15586 P06241

P18887 P06241

P20810 P06241

P21333 P06241

P22681 P06241

P23469 P06241

P23759 P06241

P23760 P06241

P26992 P06241

P29074 P06241

P29353 P06241

P30260 P06241

P31994 P06241

P31995 P06241

P32239 P06241

P35968 P06241

P42167 P06241

P42566 P06241

P42684 P06241

P42768 P06241

P43699 P06241

P46013 P06241

P46092 P06241

P50406 P06241

P50851 P06241

P56945 P06241

P78345 P06241

P78357 P06241

P98161 P06241

Q04759 P06241

Q05655 P06241

Q07666 P06241

Q07889 P06241

Q07890 P06241

Q13087 P06241

Q13094 P06241

Q13111 P06241

Q13177 P06241

Q13285 P06241

Q13415 P06241

Q13444 P06241

Q13796 P06241

Q14118 P06241

Q14185 P06241

Q14289 P06241

Q15427 P06241

Q7Z7K6 P06241

Q86SG6 P06241

Q86WV1 P06241

Q8IZD9 P06241

Q8IZP0 P06241

Q8NFP9 P06241

Q8WUM4 P06241

Q8WX92 P06241

Q92874 P06241

Q92988 P06241

Q96NS5 P06241

Q96PD2 P06241

Q96PU4 P06241

Q96RL7 P06241

Q9BQ89 P06241

Q9BWW9 P06241

Q9BYB0 P06241

Q9BZJ6 P06241

Q9H1R2 P06241

Q9H204 P06241

Q9H9L3 P06241

Q9HCM9 P06241

Q9NWQ8 P06241

Q9NYA1 P06241

Q9NYQ7 P06241

Q9P1A6 P06241

Q9UBN7 P06241

Q9UIF9 P06241

Q9UJT2 P06241

Q9UJV9 P06241

Q9UK85 P06241

Q9ULD4 P06241

Q9ULH1 P06241

Q9UMN6 P06241

Q9UQ16 P06241

Q9UQ26 P06241

Q9Y2H0 P06241

Q9Y2J2 P06241

Q9Y3Q4 P06241

Q9Y5K6 P06241

Q9Y5X2 P06241

P06276 P06276

O95166 P06280

Q01804 P06280

P32239 P06307

Q70EL2 P06312

O75674 P06396

P02768 P06396

P03372 P06396

P21796 P06396

P32121 P06396

P49407 P06396

P62993 P06396

P68133 P06396

Q92731 P06396

Q9H0R8 P06396

O00716 P06400

O14757 P06400

O15151 P06400

O43236 P06400

O60381 P06400

O75150 P06400

O96017 P06400

P00390 P06400

P04049 P06400

P06400 P06400

P07197 P06400

P19338 P06400

P21675 P06400

P24385 P06400

P24941 P06400

P33993 P06400

P35869 P06400

P39880 P06400

P55345 P06400

P62136 P06400

P62805 P06400

Q00534 P06400

Q00577 P06400

Q00987 P06400

Q01094 P06400

Q09472 P06400

Q13547 P06400

Q13574 P06400

Q13627 P06400

Q14209 P06400

Q14653 P06400

Q14686 P06400

Q16254 P06400

Q16539 P06400

Q16666 P06400

Q63HQ0 P06400

Q8WTS6 P06400

Q96DB2 P06400

Q99708 P06400

Q9UQ80 P06400

Q9Y463 P06400

Q9Y468 P06400

P04626 P06401

P08047 P06401

P40763 P06401

P55345 P06401

Q9H467 P06401

Q00537 P06454

Q01105 P06454

O00418 P06493

O15392 P06493

O94966 P06493

O95835 P06493

P00533 P06493

P01106 P06493

P01732 P06493

P0CG48 P06493

P14635 P06493

P19320 P06493

P19525 P06493

P20248 P06493

P30153 P06493

P30154 P06493

P30307 P06493

P31350 P06493

P49748 P06493

P51858 P06493

P52333 P06493

P62136 P06493

P63316 P06493

P78396 P06493

Q01538 P06493

Q12778 P06493

Q13573 P06493

Q70EL2 P06493

Q8NFA0 P06493

Q8WUF5 P06493

Q96EB6 P06493

Q96GD4 P06493

Q96KB5 P06493

Q96RL1 P06493

Q99640 P06493

Q9H0H5 P06493

Q9H211 P06493

Q9NXR1 P06493

O95059 P06576

O95166 P06576

P00533 P06576

P01023 P06576

P02751 P06576

P03372 P06576

P11802 P06576

P15336 P06576

P19320 P06576

P24539 P06576

P30519 P06576

P36873 P06576

P49407 P06576

P60520 P06576

P61981 P06576

P63104 P06576

Q06187 P06576

Q14197 P06576

Q14451 P06576

Q16637 P06576

Q63ZY3 P06576

Q969Q1 P06576

Q9GZQ8 P06576

Q9H0R8 P06576

Q9H492 P06576

Q9H845 P06576

Q9HAU4 P06576

Q9NTG7 P06576

P00533 P06702

P05109 P06702

P13569 P06702

P17948 P06702

P29353 P06702

P30153 P06702

P32121 P06702

P49407 P06702

P60520 P06702

P60953 P06702

P62993 P06702

Q00005 P06702

Q9H0R8 P06702

Q9H492 P06702

P04271 P06703

P06703 P06703

P52292 P06703

P62993 P06703

Q02790 P06703

P02768 P06727

O43586 P06729

O95400 P06729

P06241 P06729

P19256 P06729

Q96B97 P06729

Q9Y5K6 P06729

O00571 P06730

O15372 P06730

O60516 P06730

P19320 P06730

P35568 P06730

P42704 P06730

P55884 P06730

P63165 P06730

P67775 P06730

Q04637 P06730

Q04743 P06730

Q13541 P06730

Q13542 P06730

Q14240 P06730

Q9BUB5 P06730

Q9NRA8 P06730

Q9UKN5 P06730

Q9UKV8 P06730

Q9UL03 P06730

P06731 P06731

P13688 P06731

Q15051 P06732

O14503 P06733

O15264 P06733

O95166 P06733

P02751 P06733

P04075 P06733

P05556 P06733

P07237 P06733

P0DI81 P06733

P12004 P06733

P15336 P06733

P16104 P06733

P17661 P06733

P19320 P06733

P22303 P06733

P41182 P06733

P43351 P06733

P45973 P06733

P52789 P06733

P60520 P06733

P60709 P06733

P63104 P06733

Q13326 P06733

Q14315 P06733

Q496Y0 P06733

Q8N7H5 P06733

Q8NCP5 P06733

Q8NFD5 P06733

Q8WZ42 P06733

Q92966 P06733

Q99816 P06733

Q9GZQ8 P06733

Q9H0R8 P06733

Q9Y6Y0 P06733

O14920 P06737

O94966 P06737

P48735 P06737

P60520 P06737

Q99933 P06737

Q9H2F3 P06737

P60520 P06744

Q9GZQ8 P06744

O95251 P06746

P78362 P06746

Q04724 P06746

Q9H5J8 P06746

A0JLT2 P06748

O95166 P06748

P02751 P06748

P03372 P06748

P04637 P06748

P05549 P06748

P07919 P06748

P0CG48 P06748

P11387 P06748

P19320 P06748

P26599 P06748

P31268 P06748

P32121 P06748

P33527 P06748

P43686 P06748

P49407 P06748

P49450 P06748

P52292 P06748

P55345 P06748

P60520 P06748

P60709 P06748

P62805 P06748

P62993 P06748

P63104 P06748

Q00987 P06748

Q07955 P06748

Q13111 P06748

Q14137 P06748

Q14686 P06748

Q14781 P06748

Q5JVS0 P06748

Q5S007 P06748

Q5U5Q3 P06748

Q6MZP7 P06748

Q8IZL8 P06748

Q8N726 P06748

Q92769 P06748

Q99459 P06748

Q9BQG0 P06748

Q9BTT4 P06748

Q9BXL5 P06748

Q9BZQ8 P06748

Q9GZQ8 P06748

Q9H0R8 P06748

Q9H492 P06748

Q9H4L4 P06748

Q9HC52 P06748

Q9NWA0 P06748

Q9NX70 P06748

Q9UKV8 P06748

Q9UL18 P06748

Q9UQ80 P06748

P68400 P06748

O14641 P06753

P02751 P06753

P08238 P06753

P13569 P06753

P15336 P06753

Q12931 P06753

Q14240 P06753

Q6P0Q8 P06753

Q9BQD3 P06753

Q9BY41 P06753

Q9Y6D9 P06753

P02751 P06756

P05106 P06756

P05556 P06756

P18084 P06756

P23471 P06756

P26012 P06756

Q9Y5C1 P06756

P34998 P06850

P53350 P06858

Q7L5N1 P06858

Q9H832 P06858

Q9P2H0 P06858

Q9Y383 P06858

P00519 P06865

P06241 P06865

P16104 P06865

P46108 P06865

Q16602 P06881

Q96RK4 P06881

P02751 P06899

P19320 P06899

Q6NYC1 P06899

P49810 P07098

P54727 P07108

P68543 P07108

Q96CD2 P07108

Q9BPW8 P07108

P62993 P07148

O15264 P07195

O95758 P07195

P02751 P07195

P15336 P07195

P16104 P07195

P19320 P07195

P43351 P07195

P60763 P07195

O60346 P07196

P01375 P07196

P05067 P07196

P08670 P07196

Q12873 P07196

Q8N2W9 P07196

Q96LA8 P07196

Q9NPI1 P07196

Q9P0W5 P07196

O95758 P07197

P01100 P07197

P05412 P07197

P06400 P07197

P14921 P07197

P60763 P07197

Q13501 P07197

Q15051 P07197

Q15796 P07197

Q9UKE5 P07197

O14777 P07199

P04150 P07199

P04183 P07199

P14921 P07199

P20366 P07199

P24522 P07199

P49841 P07199

P67870 P07199

Q16637 P07199

Q9BZD4 P07199

Q9H5J8 P07199

P00734 P07204

Q9NZ42 P07204

O95402 P07237

P15336 P07237

P16104 P07237

P16615 P07237

P21246 P07237

P30101 P07237

P32121 P07237

P49407 P07237

P60520 P07237

P63104 P07237

Q03518 P07237

Q14240 P07237

Q8TCT9 P07237

Q96HE7 P07237

Q99689 P07237

P01011 P07288

P01857 P07288

P02768 P07288

P10275 P07288

O15315 P07305

O76075 P07305

O95166 P07305

P62993 P07305

P63104 P07305

Q8NC60 P07305

Q9Y6K9 P07305

Q6UXB4 P07306

Q7Z6E9 P07306

Q96G23 P07306

P00367 P07307

Q14469 P07307

P02489 P07315

P02511 P07315

P07315 P07315

P43320 P07315

P19784 P07327

P07332 P07332

P15311 P07332

P46459 P07332

Q6NUN9 P07332

Q96BV0 P07332

P06241 P07333

P08238 P07333

P05067 P07339

P16104 P07339

P19320 P07339

P28482 P07339

P38606 P07339

Q86YJ5 P07339

Q9H0R8 P07339

O95758 P07355

P01106 P07355

P02751 P07355

P03372 P07355

P04626 P07355

P12004 P07355

P16104 P07355

P17096 P07355

P17948 P07355

P19320 P07355

P21860 P07355

P32121 P07355

P40692 P07355

P49407 P07355

P60709 P07355

P62993 P07355

P63104 P07355

Q00005 P07355

Q15051 P07355

Q9H0R8 P07355

Q9UJM3 P07355

Q9Y6R4 P07355

P04275 P07359

P31946 P07359

P61981 P07359

P62258 P07359

P63104 P07359

Q04917 P07359

P15311 P07384

P19320 P07384

P32121 P07384

P40763 P07384

P53805 P07384

B2Y833 P07437

O75381 P07437

O95166 P07437

O95758 P07437

O95793 P07437

P00533 P07437

P04637 P07437

P05067 P07437

P07900 P07437

P15336 P07437

P19320 P07437

P36873 P07437

P42771 P07437

P45973 P07437

P46781 P07437

P49407 P07437

P60520 P07437

P60709 P07437

P61981 P07437

P62993 P07437

P63104 P07437

Q00005 P07437

Q00987 P07437

Q07157 P07437

Q13546 P07437

Q13573 P07437

Q15051 P07437

Q6IBW4 P07437

Q8IXJ6 P07437

Q96L34 P07437

Q99459 P07437

Q9GZQ8 P07437

Q9H0R8 P07437

Q9H492 P07437

Q9H9G7 P07437

Q9HCK5 P07437

Q9NRI5 P07437

P02768 P07477

Q00005 P07477

O14745 P07550

P07550 P07550

P12931 P07550

P62633 P07550

Q13639 P07550

Q14232 P07550

Q5TCQ9 P07550

Q7Z362 P07550

Q96B67 P07550

O15198 P07602

O96006 P07602

P13569 P07602

Q05086 P07602

Q13326 P07602

Q15796 P07602

Q7L5N1 P07602

O75530 P07711

P01876 P07711

P02647 P07711

P02768 P07711

P02787 P07711

P36873 P07711

O15264 P07737

O95166 P07737

P03372 P07737

P15336 P07737

P17020 P07737

P19320 P07737

P43351 P07737

P60520 P07737

P60709 P07737

Q04724 P07737

Q13432 P07737

Q14194 P07737

Q5UIP0 P07737

Q8TDM6 P07737

Q96SB4 P07737

Q9GZQ8 P07737

Q9H0R8 P07737

Q9UKR5 P07737

P18850 P07738

P19835 P07738

P51164 P07738

P51956 P07738

P62993 P07738

Q9NZN4 P07738

P19320 P07741

P40692 P07741

O43639 P07766

P04234 P07766

P09693 P07766

P16333 P07766

P32248 P07766

P43405 P07766

Q12929 P07766

Q8TE67 P07766

Q8TE68 P07766

Q9Y2R2 P07766

O15264 P07814

O60506 P07814

P07900 P07814

P19320 P07814

P23508 P07814

P41252 P07814

P54136 P07814

P60520 P07814

Q14164 P07814

Q15750 P07814

Q5S007 P07814

Q9H492 P07814

Q9UKV8 P07814

Q9UL18 P07814

O43597 P07858

P01040 P07858

P02760 P07858

P20073 P07858

P38936 P07858

P40692 P07858

P46379 P07858

Q09019 P07858

Q6UY14 P07858

Q8NE63 P07858

Q9HBJ7 P07858

Q9NYA1 P07858

O43318 P07900

O95166 P07900

O95758 P07900

P00533 P07900

P04150 P07900

P04626 P07900

P05067 P07900

P07900 P07900

P08253 P07900

P09914 P07900

P11802 P07900

P12544 P07900

P12830 P07900

P15336 P07900

P16104 P07900

P17612 P07900

P21860 P07900

P22694 P07900

P24941 P07900

P25098 P07900

P33993 P07900

P41743 P07900

P42771 P07900

P47897 P07900

P49407 P07900

P50750 P07900

P53041 P07900

P56192 P07900

P60520 P07900

P60709 P07900

P61247 P07900

P63104 P07900

Q00005 P07900

Q00535 P07900

Q02790 P07900

Q08752 P07900

Q13131 P07900

Q13573 P07900

Q14318 P07900

Q14342 P07900

Q15051 P07900

Q15185 P07900

Q15418 P07900

Q15831 P07900

Q16543 P07900

Q5S007 P07900

Q8TDR0 P07900

Q96G23 P07900

Q99459 P07900

Q9GZX7 P07900

Q9H093 P07900

Q9H0R8 P07900

Q9H492 P07900

Q9H9G7 P07900

Q9HCK5 P07900

Q9HCU9 P07900

Q9UHD1 P07900

Q9UKP3 P07900

Q9UKV8 P07900

Q9UNE7 P07900

Q9Y239 P07900

P19320 P07900

Q15645 P07902

O95166 P07910

P01106 P07910

P01116 P07910

P07910 P07910

P09651 P07910

P09914 P07910

P11387 P07910

P16104 P07910

P19320 P07910

P19525 P07910

P32121 P07910

P38919 P07910

P42771 P07910

P49761 P07910

P51116 P07910

P60520 P07910

P61326 P07910

P63104 P07910

P63279 P07910

P67809 P07910

P78362 P07910

Q09161 P07910

Q13435 P07910

Q13526 P07910

Q13573 P07910

Q14164 P07910

Q86SE5 P07910

Q8IYQ9 P07910

Q8TAP4 P07910

Q92879 P07910

Q93100 P07910

Q96IZ5 P07910

Q96SB4 P07910

Q99459 P07910

Q9BXS5 P07910

Q9GZQ8 P07910

Q9H0R8 P07910

Q9H492 P07910

Q9HCK5 P07910

Q9NR30 P07910

Q9P0T4 P07910

Q9UKV8 P07910

Q9UL18 P07910

Q9Y5S9 P07910

P08574 P07919

Q9NTG7 P07919

O15230 P07942

O15265 P07942

P24043 P07942

P25391 P07942

Q16363 P07942

Q16787 P07942

Q92731 P07942

O14641 P07947

P00533 P07947

P02751 P07947

P19320 P07947

P25445 P07947

P35222 P07947

P46527 P07947

P48023 P07947

P49407 P07947

Q13177 P07947

Q8IZP0 P07947

Q8WUM4 P07947

Q96KM6 P07947

Q9H204 P07947

Q9H5V8 P07947

Q9HCN6 P07947

P00533 P07948

P05107 P07948

P05556 P07948

P07948 P07948

P08238 P07948

P11049 P07948

P20273 P07948

P21145 P07948

P25063 P07948

P29350 P07948

P29353 P07948

P31994 P07948

P33993 P07948

P48023 P07948

P67870 P07948

Q07666 P07948

Q15642 P07948

Q7Z7K6 P07948

Q9HCN6 P07948

O60716 P07949

P22681 P07949

P40763 P07949

P62993 P07949

P42574 P07949

O95295 P07951

P12757 P07951

P31749 P07951

P62993 P07951

P15336 P07954

Q15051 P07954

P23025 P07992

Q8IY92 P07992

Q96S82 P07992

Q99459 P07992

Q9BQ83 P07992

P01137 P07996

P02751 P07996

P07996 P07996

P08567 P07996

P21980 P07996

Q15051 P07996

P13489 P07998

O00716 P08047

P00519 P08047

P00846 P08047

P01106 P08047

P03372 P08047

P03905 P08047

P03915 P08047

P04198 P08047

P04637 P08047

P06401 P08047

P08047 P08047

P12931 P08047

P14859 P08047

P17096 P08047

P21453 P08047

P32519 P08047

P40763 P08047

P42858 P08047

P46108 P08047

P51532 P08047

P51610 P08047

P60174 P08047

Q01094 P08047

Q01196 P08047

Q01780 P08047

Q04206 P08047

Q06455 P08047

Q06546 P08047

Q12756 P08047

Q12772 P08047

Q13118 P08047

Q13427 P08047

Q13485 P08047

Q13952 P08047

Q15459 P08047

Q15759 P08047

Q16665 P08047

Q5T5U3 P08047

Q7Z3K3 P08047

Q92769 P08047

Q92988 P08047

Q9NRR4 P08047

Q9NV58 P08047

Q9NY61 P08047

Q9UBL3 P08047

O15552 P08069

P04746 P08069

P08069 P08069

P08238 P08069

P18031 P08069

P27986 P08069

P29353 P08069

P31946 P08069

P46108 P08069

P49913 P08069

P62258 P08069

Q00987 P08069

Q01995 P08069

Q06124 P08069

Q9NZN5 P08069

Q9UJU2 P08069

Q9Y4H2 P08069

O95405 P08100

Q15075 P08100

O00635 P08107

O14727 P08107

O43156 P08107

O94826 P08107

O95166 P08107

O95758 P08107

O95816 P08107

O95817 P08107

O95831 P08107

P00533 P08107

P01100 P08107

P01106 P08107

P02751 P08107

P03372 P08107

P07900 P08107

P08238 P08107

P08473 P08107

P10636 P08107

P12544 P08107

P12830 P08107

P13569 P08107

P15056 P08107

P15976 P08107

P16104 P08107

P17096 P08107

P19320 P08107

P27348 P08107

P27695 P08107

P31946 P08107

P32121 P08107

P32745 P08107

P37840 P08107

P43351 P08107

P46781 P08107

P49407 P08107

P49841 P08107

P53041 P08107

P53350 P08107

P54725 P08107

P60520 P08107

P61326 P08107

P61981 P08107

P63104 P08107

P84022 P08107

P85299 P08107

Q00005 P08107

Q02750 P08107

Q06710 P08107

Q12905 P08107

Q12982 P08107

Q13464 P08107

Q14197 P08107

Q14324 P08107

Q15796 P08107

Q15831 P08107

Q53FT3 P08107

Q6NXR4 P08107

Q6PJG9 P08107

Q6R327 P08107

Q6T424 P08107

Q8IXJ6 P08107

Q8TDR0 P08107

Q8TF46 P08107

Q8WZ42 P08107

Q92731 P08107

Q92905 P08107

Q96QF0 P08107

Q99933 P08107

Q9BXA6 P08107

Q9BXL5 P08107

Q9GZQ8 P08107

Q9H0R8 P08107

Q9H492 P08107

Q9HB09 P08107

Q9HCK5 P08107

Q9NYB0 P08107

Q9UN19 P08107

Q9UNE7 P08107

Q9Y4R8 P08107

P02452 P08123

P17301 P08123

P08473 P08133

P21926 P08133

P40692 P08133

P60709 P08133

P62993 P08133

Q07157 P08133

Q6P1J9 P08133

O60610 P08134

P15336 P08134

Q07960 P08134

P02766 P08138

P04844 P08138

P08138 P08138

P16104 P08138

P18754 P08138

P20073 P08138

P38936 P08138

Q16637 P08138

Q92890 P08138

Q9Y467 P08138

P23443 P08151

P40337 P08151

P62258 P08151

Q96J02 P08151

Q9UMX1 P08151

Q5JY77 P08172

Q96D09 P08172

P01106 P08183

Q99496 P08183

Q9BPZ7 P08183

Q9P287 P08183

P00533 P08195

P02751 P08195

P04626 P08195

P15336 P08195

P16070 P08195

P21926 P08195

Q8NCN4 P08195

P19793 P08235

P33121 P08235

P01023 P08236

Q14161 P08236

P08237 P08237

P17858 P08237

P27361 P08237

Q13573 P08237

Q99459 P08237

Q9UK45 P08237

A9UEZ6 P08238

C9JYL6 P08238

H0YE48 P08238

O00141 P08238

O14544 P08238

O14682 P08238

O14757 P08238

O15111 P08238

O15146 P08238

O15197 P08238

O43930 P08238

O60260 P08238

O75426 P08238

O75469 P08238

O75478 P08238

O75916 P08238

O94810 P08238

O94844 P08238

O94868 P08238

O94921 P08238

O94972 P08238

O95166 P08238

O95433 P08238

P00519 P08238

P00533 P08238

P03372 P08238

P04049 P08238

P04626 P08238

P05771 P08238

P06241 P08238

P06733 P08238

P07332 P08238

P07900 P08238

P07947 P08238

P08235 P08238

P08238 P08238

P09769 P08238

P09914 P08238

P0CI25 P08238

P10398 P08238

P11362 P08238

P11801 P08238

P11802 P08238

P13569 P08238

P13805 P08238

P14616 P08238

P15056 P08238

P15336 P08238

P15918 P08238

P16591 P08238

P17612 P08238

P17658 P08238

P18847 P08238

P19320 P08238

P20594 P08238

P21860 P08238

P22460 P08238

P22694 P08238

P24723 P08238

P24941 P08238

P25098 P08238

P29597 P08238

P31152 P08238

P31751 P08238

P32121 P08238

P32298 P08238

P33993 P08238

P36894 P08238

P36956 P08238

P37231 P08238

P41279 P08238

P41743 P08238

P42224 P08238

P42679 P08238

P42685 P08238

P42771 P08238

P43250 P08238

P49116 P08238

P49674 P08238

P49754 P08238

P49758 P08238

P49840 P08238

P50613 P08238

P50750 P08238

P51451 P08238

P51817 P08238

P53041 P08238

P53667 P08238

P53671 P08238

P54646 P08238

P54764 P08238

P56179 P08238

P60520 P08238

P60709 P08238

P61244 P08238

P62993 P08238

P63104 P08238

P63208 P08238

P68400 P08238

P80192 P08238

Q00526 P08238

Q00534 P08238

Q00535 P08238

Q01974 P08238

Q03181 P08238

Q04759 P08238

Q05397 P08238

Q06187 P08238

Q07002 P08238

Q07912 P08238

Q08380 P08238

Q08752 P08238

Q09013 P08238

Q13049 P08238

Q13105 P08238

Q13131 P08238

Q13133 P08238

Q13163 P08238

Q13164 P08238

Q13464 P08238

Q13470 P08238

Q13541 P08238

Q13546 P08238

Q13557 P08238

Q13573 P08238

Q13616 P08238

Q13617 P08238

Q13618 P08238

Q13705 P08238

Q13882 P08238

Q14190 P08238

Q14289 P08238

Q14994 P08238

Q15047 P08238

Q15131 P08238

Q15139 P08238

Q15185 P08238

Q15208 P08238

Q15303 P08238

Q15393 P08238

Q15418 P08238

Q15562 P08238

Q15831 P08238

Q16512 P08238

Q16543 P08238

Q16566 P08238

Q16587 P08238

Q16620 P08238

Q16659 P08238

Q16671 P08238

Q2M1V0 P08238

Q2WGJ6 P08238

Q32MK0 P08238

Q3SYB3 P08238

Q53GT1 P08238

Q53HC5 P08238

Q5GLZ8 P08238

Q5H9I0 P08238

Q5S007 P08238

Q5SSJ5 P08238

Q5TEA3 P08238

Q5VZB9 P08238

Q6J9G0 P08238

Q6JEL2 P08238

Q6NXR4 P08238

Q6P0Q8 P08238

Q6PJ21 P08238

Q6T424 P08238

Q6VAB6 P08238

Q6VVB1 P08238

Q6ZMZ0 P08238

Q6ZNA4 P08238

Q6ZWB6 P08238

Q6ZSB9 P08238

Q7L3B6 P08238

Q7L5Y6 P08238

Q7L622 P08238

Q7Z6M2 P08238

Q86SG6 P08238

Q86UP8 P08238

Q86UV6 P08238

Q86UV7 P08238

Q86V86 P08238

Q86YJ5 P08238

Q86YV5 P08238

Q86YV6 P08238

Q8IVT5 P08238

Q8IVU3 P08238

Q8IYA7 P08238

Q8N165 P08238

Q8N1E6 P08238

Q8N239 P08238

Q8N4C8 P08238

Q8N4N3 P08238

Q8N5S9 P08238

Q8N5U6 P08238

Q8NBE8 P08238

Q8NCB2 P08238

Q8NDN9 P08238

Q8NE63 P08238

Q8NEE6 P08238

Q8NER5 P08238

Q8NG66 P08238

Q8NI29 P08238

Q8TCJ0 P08238

Q8TD08 P08238

Q8TDR0 P08238

Q8TEC5 P08238

Q8TF76 P08238

Q8WTQ7 P08238

Q8WV44 P08238

Q8WVZ9 P08238

Q8WXH6 P08238

Q8WXJ9 P08238

Q8WXR4 P08238

Q8WY36 P08238

Q8WY91 P08238

Q8WZ60 P08238

Q92615 P08238

Q92905 P08238

Q92918 P08238

Q969H0 P08238

Q96A26 P08238

Q96BR1 P08238

Q96C12 P08238

Q96CT2 P08238

Q96GD4 P08238

Q96M94 P08238

Q96ME1 P08238

Q96NJ5 P08238

Q96NX5 P08238

Q96Q27 P08238

Q96QP1 P08238

Q96QS6 P08238

Q96RG2 P08238

Q96RR4 P08238

Q96S44 P08238

Q96S53 P08238

Q96SB4 P08238

Q99459 P08238

Q9BQ31 P08238

Q9BQI3 P08238

Q9BUB5 P08238

Q9BXA6 P08238

Q9BXA7 P08238

Q9BYE7 P08238

Q9BZL6 P08238

Q9GZQ8 P08238

Q9GZX7 P08238

Q9H0H3 P08238

Q9H0M0 P08238

Q9H0R8 P08238

Q9H3D4 P08238

Q9H469 P08238

Q9H492 P08238

Q9H9G7 P08238

Q9HC78 P08238

Q9HCC6 P08238

Q9HCK5 P08238

Q9HCU5 P08238

Q9NR20 P08238

Q9NR64 P08238

Q9NRD1 P08238

Q9NVF7 P08238

Q9NVX7 P08238

Q9NWN3 P08238

Q9NWX5 P08238

Q9NY57 P08238

Q9NYS7 P08238

Q9P0U4 P08238

Q9P1W9 P08238

Q9P253 P08238

Q9P2G3 P08238

Q9P2N7 P08238

Q9UDY6 P08238

Q9UGR2 P08238

Q9UH90 P08238

Q9UHD2 P08238

Q9UIX4 P08238

Q9UJX6 P08238

Q9UK32 P08238

Q9UK96 P08238

Q9UKT7 P08238

Q9UKV8 P08238

Q9UL18 P08238

Q9UL58 P08238

Q9UM73 P08238

Q9UNE7 P08238

Q9UQ88 P08238

Q9UQB9 P08238

Q9Y2E6 P08238

Q9Y2H1 P08238

Q9Y3M8 P08238

Q9Y458 P08238

Q9Y463 P08238

Q9Y574 P08238

Q9Y575 P08238

Q9Y616 P08238

Q9Y696 P08238

Q9Y6S9 P08238

P62906 P08240

P19320 P08243

Q9BSB4 P08243

Q9BW61 P08243

P10144 P08246

O75317 P08253

P39060 P08253

Q8IX30 P08253

Q9BXB5 P08253

Q9GZQ8 P08253

Q11201 P08263

Q86UA6 P08263

P01023 P08311

P42766 P08319

P08107 P08473

P21926 P08473

P60709 P08473

Q9BY41 P08473

P05106 P08514

P08514 P08514

O94966 P08559

P19320 P08559

Q15118 P08559

Q8TBZ3 P08559

Q9NWV8 P08559

Q14642 P08567

Q12841 P08571

Q96LZ3 P08571

P02462 P08572

P12757 P08572

P20073 P08572

Q15038 P08572

Q16637 P08572

Q5TF85 P08572

Q92876 P08572

P01106 P08574

P15336 P08574

Q13286 P08574

P04629 P08575

P06213 P08575

P06239 P08575

P08575 P08575

P09382 P08575

P09619 P08575

P10912 P08575

P19022 P08575

P20273 P08575

P20701 P08575

P22681 P08575

P41240 P08575

P49023 P08575

Q02763 P08575

Q13224 P08575

Q13480 P08575

Q92854 P08575

O14879 P08579

P09661 P08579

P09913 P08579

P09914 P08579

Q13573 P08579

Q15428 P08579

Q96DI7 P08579

Q9UKE5 P08579

O15031 P08581

O43157 P08581

P00533 P08581

P08575 P08581

P14210 P08581

P16070 P08581

P18031 P08581

P22681 P08581

P23467 P08581

P35968 P08581

Q06124 P08581

Q12913 P08581

Q13480 P08581

Q96EY1 P08581

Q9ULL4 P08581

Q5JY77 P08588

Q5TCQ9 P08588

Q86UL8 P08588

Q96D09 P08588

Q9HD26 P08588

P02794 P08590

P63104 P08590

P01024 P08603

P02768 P08603

P62993 P08603

P84022 P08603

O14879 P08621

O15198 P08621

O75554 P08621

O95166 P08621

P02751 P08621

P09913 P08621

P09914 P08621

P12757 P08621

P19320 P08621

P38919 P08621

P49760 P08621

P60520 P08621

P61326 P08621

P78362 P08621

Q13573 P08621

Q15428 P08621

Q16637 P08621

Q8IVW4 P08621

Q92769 P08621

Q96MT8 P08621

Q99459 P08621

Q99717 P08621

Q9BZL6 P08621

Q9H0R8 P08621

Q9UPE1 P08621

P68400 P08621

P00519 P08631

P00533 P08631

P08238 P08631

P17948 P08631

P19174 P08631

P35326 P08631

P42684 P08631

P42768 P08631

P46109 P08631

P48023 P08631

P61978 P08631

Q07666 P08631

Q07889 P08631

Q13094 P08631

Q13177 P08631

Q14192 P08631

Q8IZP0 P08631

Q96T51 P08631

Q9H204 P08631

Q9Y4K4 P08631

Q9Y5K6 P08631

P24278 P08637

O95630 P08648

P02751 P08648

P05556 P08648

P17813 P08648

P19320 P08648

P29279 P08648

P36897 P08648

P37023 P08648

P39060 P08648

Q9H0F6 P08648

P08238 P08651

Q13573 P08651

Q96J77 P08651

Q9BRT6 P08651

Q9H0I9 P08651

Q9P0L1 P08651

O00151 P08670

O15160 P08670

O43464 P08670

O95166 P08670

O95251 P08670

O95361 P08670

O95990 P08670

P06132 P08670

P08670 P08670

P13569 P08670

P15090 P08670

P15336 P08670

P17861 P08670

P19320 P08670

P20073 P08670

P24522 P08670

P27694 P08670

P31749 P08670

P31751 P08670

P32121 P08670

P35900 P08670

P37840 P08670

P49407 P08670

P51693 P08670

P54727 P08670

P55055 P08670

P60520 P08670

P60709 P08670

P61326 P08670

P61981 P08670

P62993 P08670

P63104 P08670

Q00005 P08670

Q07157 P08670

Q13501 P08670

Q13573 P08670

Q15046 P08670

Q15047 P08670

Q53TS8 P08670

Q5RL73 P08670

Q5S007 P08670

Q5UIP0 P08670

Q6ZU52 P08670

Q7L311 P08670

Q7L5N1 P08670

Q8N0X7 P08670

Q8N283 P08670

Q8N2W9 P08670

Q8NEY8 P08670

Q8WYH8 P08670

Q92731 P08670

Q96DF8 P08670

Q96GX9 P08670

Q99459 P08670

Q99961 P08670

Q9BT92 P08670

Q9BVI4 P08670

Q9BVJ6 P08670

Q9BVR6 P08670

Q9GZQ8 P08670

Q9GZT8 P08670

Q9H0R8 P08670

Q9H492 P08670

Q9H4K1 P08670

Q9H6U6 P08670

Q9NRH1 P08670

Q9P2H0 P08670

Q9UI14 P08670

Q9UKY1 P08670

O00264 P08684

O95166 P08708

P10644 P08708

P32121 P08708

P49407 P08708

P51668 P08708

P60520 P08708

P62280 P08708

P62837 P08708

Q92731 P08708

Q9H0R8 P08708

Q9H492 P08708

P02768 P08709

P08709 P08709

Q09019 P08709

Q14457 P08709

Q70CQ1 P08709

Q8IY92 P08709

Q96PM5 P08709

Q96RL1 P08709

Q9H0R8 P08709

O14964 P08727

O95990 P08727

P04049 P08727

P11532 P08727

P19320 P08727

P25054 P08727

P63104 P08727

Q14966 P08727

Q15154 P08727

Q8IYI6 P08727

Q8N6Y0 P08727

Q8NFA0 P08727

Q9BY27 P08727

Q9HAU0 P08727

Q9NTK1 P08727

Q9NYB9 P08727

P01857 P08729

P15336 P08729

P62993 P08729

Q14152 P08729

A0AUL9 P08754

P02751 P08754

P19320 P08754

P46060 P08754

P48039 P08754

P49286 P08754

P54707 P08754

P80303 P08754

Q02818 P08754

P15336 P08758

P16104 P08758

P30154 P08758

P37268 P08758

Q9Y6N6 P08758

O95166 P08779

P01857 P08779

P02647 P08779

P02768 P08779

P19320 P08779

P38919 P08779

P60520 P08779

Q00005 P08779

Q9GZQ8 P08779

Q9H0R8 P08779

P01023 P08842

P03372 P08865

P07225 P08865

P08238 P08865

P12004 P08865

P19320 P08865

P22732 P08865

P33897 P08865

P60709 P08865

P62158 P08865

Q6UXB4 P08865

Q9H3D4 P08865

Q9HCJ1 P08865

P05231 P08887

Q9NZ08 P08887

P08908 P08908

P11362 P08908

P01033 P08962

P05556 P08962

P12931 P08962

P21926 P08962

P38606 P08962

Q92574 P08962

P67809 P09001

Q14197 P09001

Q9BYC9 P09001

O14879 P09012

O95166 P09012

O95758 P09012

P02751 P09012

P09012 P09012

P09913 P09012

P09914 P09012

P42771 P09012

P60520 P09012

P78362 P09012

Q99459 P09012

Q9H3D4 P09012

Q9UPE1 P09012

O43463 P09017

Q96LA8 P09017

Q9H0A6 P09017

P11362 P09038

P113624 P09038

P21802 P09038

P23352 P09038

P39019 P09038

P01106 P09067

O00762 P09104

O43768 P09104

O75251 P09104

O95166 P09104

O95873 P09104

P19367 P09104

P27816 P09104

P50995 P09104

P60520 P09104

P68366 P09104

Q00613 P09104

Q5JVS0 P09104

Q6IA17 P09104

Q9BTE0 P09104

Q9H0R8 P09104

Q9H492 P09104

Q9UHF5 P09104

Q6PKC3 P09110

O95758 P09132

Q13573 P09132

O95166 P09211

O95758 P09211

P15336 P09211

P19320 P09211

P21246 P09211

P40692 P09211

P60520 P09211

P62993 P09211

Q00987 P09211

Q9GZQ8 P09211

Q9H0R8 P09211

Q9H492 P09211

Q9NRX1 P09211

Q9Y371 P09211

O14879 P09234

O43251 P09234

O75525 P09234

O95166 P09234

P09913 P09234

P09914 P09234

P52597 P09234

P60520 P09234

P78362 P09234

P98175 P09234

Q12906 P09234

Q15428 P09234

Q9BQA1 P09234

Q9BRX9 P09234

Q9BUJ2 P09234

Q9GZQ8 P09234

Q9H0R8 P09234

Q9H492 P09234

Q9UPE1 P09234

P15502 P09237

P29692 P09326

Q9BZW8 P09326

P09327 P09327

P19174 P09327

P01106 P09382

P02751 P09382

P19320 P09382

P60953 P09382

P63104 P09382

P02647 P09417

Q15051 P09417

O15350 P09429

O95758 P09429

P02751 P09429

P04637 P09429

P10599 P09429

P17096 P09429

P19320 P09429

P28482 P09429

P48729 P09429

P50570 P09429

Q13432 P09429

Q14194 P09429

Q5UIP0 P09429

Q9UKR5 P09429

Q9Y6K9 P09455

P12956 P09466

P68104 P09466

P84022 P09466

Q5UIP0 P09466

Q7L5N1 P09466

Q96ID5 P09466

O00499 P09467

P09467 P09467

P26717 P09467

P51116 P09467

Q08043 P09467

Q63HM2 P09467

Q8TBB1 P09467

Q96D59 P09467

P12956 P09486

Q8NAF0 P09486

P62993 P09488

Q15051 P09488

O95295 P09493

P15336 P09493

P39060 P09493

P62993 P09493

P63104 P09493

Q6P0Q8 P09493

Q8N3C7 P09493

Q9BQD3 P09493

O95630 P09496

P00533 P09496

P10242 P09496

P13569 P09496

P19320 P09496

P32121 P09496

P35222 P09496

P49407 P09496

Q14008 P09496

Q15796 P09496

Q9GZQ8 P09496

Q9H492 P09496

P02751 P09497

P32121 P09497

P17405 P09525

Q9H0R8 P09525

O75381 P09543

P04156 P09543

P48039 P09543

O14957 P09544

P08631 P09544

P55064 P09544

Q8N474 P09544

Q9HB90 P09544

P25963 P09564

O43889 P09601

P04424 P09601

P01106 P09603

O14492 P09619

O14745 P09619

P04049 P09619

P06241 P09619

P07947 P09619

P08238 P09619

P12931 P09619

P18031 P09619

P19174 P09619

P20936 P09619

P27449 P09619

P27986 P09619

P60484 P09619

P62993 P09619

Q05209 P09619

Q06124 P09619

Q12913 P09619

Q13239 P09619

Q13322 P09619

Q14451 P09619

Q15262 P09619

Q16827 P09619

Q9BQ66 P09619

P02751 P09622

P09622 P09622

P15336 P09622

P62993 P09622

Q14197 P09622

Q9H0R8 P09622

Q9H492 P09622

P12956 P09629

P13010 P09629

P78527 P09629

Q9Y616 P09629

O14879 P09651

O14948 P09651

O15264 P09651

O60477 P09651

O95166 P09651

O95758 P09651

P03372 P09651

P06730 P09651

P07910 P09651

P09913 P09651

P11021 P09651

P11387 P09651

P16104 P09651

P17096 P09651

P19320 P09651

P23246 P09651

P31942 P09651

P32121 P09651

P38919 P09651

P49407 P09651

P60520 P09651

P60709 P09651

P62993 P09651

P63104 P09651

Q07666 P09651

Q09161 P09651

Q13268 P09651

Q13573 P09651

Q15428 P09651

Q5BKZ1 P09651

Q5U5Q3 P09651

Q9BZI7 P09651

Q9GZQ8 P09651

Q9H0R8 P09651

Q9H1J1 P09651

Q9H492 P09651

Q9UKV8 P09651

Q9UL18 P09651

Q9Y5S9 P09651

O14879 P09661

P08579 P09661

P09012 P09661

P09913 P09661

P09914 P09661

P11387 P09661

P19320 P09661

Q15428 P09661

Q16637 P09661

Q96FJ0 P09661

Q99459 P09661

P19320 P09668

P00403 P09669

P13984 P09669

P21246 P09669

P35232 P09669

Q96PM5 P09669

Q9UJS0 P09669

Q9Y2R0 P09669

P15336 P09758

P38606 P09758

P04626 P09769

P08238 P09769

P40763 P09769

P42768 P09769

P00736 P09871

P09871 P09871

B2Y833 P09874

O00716 P09874

O15264 P09874

O75323 P09874

O75928 P09874

O95863 P09874

P03372 P09874

P04637 P09874

P09874 P09874

P10276 P09874

P11308 P09874

P11387 P09874

P11388 P09874

P12956 P09874

P13010 P09874

P14921 P09874

P16104 P09874

P17947 P09874

P18887 P09874

P19320 P09874

P22415 P09874

P42574 P09874

P49715 P09874

P50549 P09874

P51858 P09874

P63165 P09874

P78527 P09874

Q01094 P09874

Q13131 P09874

Q14191 P09874

Q14781 P09874

Q2M1K9 P09874

Q5U5Q3 P09874

Q7Z2E3 P09874

Q86W56 P09874

Q8IW19 P09874

Q8IY92 P09874

Q8N2W9 P09874

Q99459 P09874

Q9BVS4 P09874

Q9NQB0 P09874

Q9P275 P09874

Q9Y530 P09874

P24386 P09884

P27694 P09884

Q14181 P09884

O14879 P09913

P09914 P09913

O14879 P09914

P09913 P09914

Q14019 P09917

O00716 P09923

Q14240 P09923

P05067 P09936

P46527 P09936

P63279 P09936

Q15051 P09936

Q9UK80 P09936

O14793 P09958

O43493 P09958

O95255 P09958

P50281 P09958

Q04912 P09958

Q9NY93 P09958

Q0P6H9 P09960

Q9BW61 P09960

Q9GZZ9 P09960

P46734 P09972

Q14534 P09972

Q9H0R8 P09972

P01106 P0C0L4

P02768 P0C0L4

Q16594 P0C0L4

O43257 P0C0S5

P01106 P0C0S5

O95760 P0C0S8

P08473 P0C0S8

P49407 P0C0S8

P63104 P0C0S8

Q8N488 P0C0S8

O96019 P0C1Z6

P55199 P0C1Z6

Q53TQ3 P0C1Z6

Q8NBZ0 P0C1Z6

Q96CJ1 P0C1Z6

Q96EZ8 P0C1Z6

Q96JC9 P0C1Z6

Q9C086 P0C1Z6

Q9H981 P0C1Z6

Q9Y5K5 P0C1Z6

P51784 P0C2W1

Q0WX57 P0C7H9

Q6R6M4 P0C7H9

Q9BXU7 P0C7H9

Q06330 P0C7T5

P68363 P0C7T8

P01106 P0C7U0

P62136 P0C7U0

O60341 P0C7W6

P45973 P0C7X2

P23109 P0C862

P46976 P0C862

Q00872 P0C862

Q03001 P0C862

Q8WZ42 P0C862

Q92615 P0C862

P01709 P0CG04

P01772 P0CG04

P01857 P0CG04

P01876 P0CG04

Q8IY92 P0CG04

O00189 P0CG13

O00308 P0CG47

O94888 P0CG47

P00533 P0CG47

P03372 P0CG47

P04637 P0CG47

P09936 P0CG47

P13569 P0CG47

P16104 P0CG47

P23246 P0CG47

P28562 P0CG47

P46934 P0CG47

P54252 P0CG47

P54727 P0CG47

P55036 P0CG47

P60709 P0CG47

P61586 P0CG47

P62993 P0CG47

Q13501 P0CG47

Q14790 P0CG47

Q15038 P0CG47

Q15653 P0CG47

Q16401 P0CG47

Q16658 P0CG47

Q5VTR2 P0CG47

Q68DV7 P0CG47

Q6GQQ9 P0CG47

Q8IVM0 P0CG47

Q8N0X7 P0CG47

Q96CV9 P0CG47

Q96CS3 P0CG47

Q96PM9 P0CG47

Q99741 P0CG47

Q9BTT4 P0CG47

Q9HAU4 P0CG47

Q9NWA0 P0CG47

Q9NX70 P0CG47

Q9NZ52 P0CG47

Q9UJ41 P0CG47

Q9UJY5 P0CG47

Q9UM54 P0CG47

Q9Y2H0 P0CG47

O00308 P0CG48

O14964 P0CG48

O15151 P0CG48

O15350 P0CG48

O43150 P0CG48

O43318 P0CG48

O43583 P0CG48

O43639 P0CG48

O60566 P0CG48

O75460 P0CG48

O75604 P0CG48

O75886 P0CG48

O76080 P0CG48

O95630 P0CG48

O95786 P0CG48

O95816 P0CG48

P01106 P0CG48

P04637 P0CG48

P05412 P0CG48

P06239 P0CG48

P06493 P0CG48

P07550 P0CG48

P09874 P0CG48

P0CG48 P0CG48

P17252 P0CG48

P25098 P0CG48

P25963 P0CG48

P35226 P0CG48

P35227 P0CG48

P38936 P0CG48

P46736 P0CG48

P48357 P0CG48

P51617 P0CG48

P53355 P0CG48

P54252 P0CG48

P54727 P0CG48

P55036 P0CG48

P57075 P0CG48

P61224 P0CG48

P62993 P0CG48

P84022 P0CG48

P98170 P0CG48

Q00987 P0CG48

Q04206 P0CG48

Q06587 P0CG48

Q13233 P0CG48

Q13351 P0CG48

Q13485 P0CG48

Q13490 P0CG48

Q13501 P0CG48

Q13887 P0CG48

Q14901 P0CG48

Q14934 P0CG48

Q15034 P0CG48

Q15717 P0CG48

Q15843 P0CG48

Q16186 P0CG48

Q5MJ70 P0CG48

Q6FI35 P0CG48

Q7L2E3 P0CG48

Q86VP1 P0CG48

Q8N488 P0CG48

Q8N668 P0CG48

Q8TAT6 P0CG48

Q8TEJ3 P0CG48

Q92547 P0CG48

Q92783 P0CG48

Q969V5 P0CG48

Q96B97 P0CG48

Q96NR8 P0CG48

Q96PU5 P0CG48

Q96RN5 P0CG48

Q99062 P0CG48

Q99816 P0CG48

Q9H013 P0CG48

Q9H0M0 P0CG48

Q9H160 P0CG48

Q9HB09 P0CG48

Q9NX09 P0CG48

Q9NYJ8 P0CG48

Q9UBN7 P0CG48

Q9UDY8 P0CG48

Q9UER7 P0CG48

Q9UGI0 P0CG48

Q9UGP5 P0CG48

Q9UKV8 P0CG48

Q9UL18 P0CG48

Q9ULH1 P0CG48

Q9UNA4 P0CG48

Q9UNQ0 P0CG48

Q9Y253 P0CG48

Q9Y2U5 P0CG48

Q9Y3D6 P0CG48

Q9Y4K3 P0CG48

Q9Y5X1 P0CG48

Q9Y6K9 P0CG48

Q9Y6R4 P0CG48

Q5U5Q3 P0CW22

P11217 P0DI81

P13747 P0DI81

P13929 P0DI81

Q8N0Z2 P0DI81

Q96QF0 P0DI81

Q99613 P0DI81

P0DJJ0 P0DJJ0

P62258 P10071

Q9NRP7 P10071

Q9UMX1 P10071

Q9UQ88 P10072

P41220 P10074

Q8WV44 P10074

Q92956 P10074

Q96BR9 P10074

Q96L11 P10074

P25929 P10082

P27487 P10082

P49146 P10082

Q12884 P10082

P43354 P10109

O76062 P10114

P26885 P10114

P49354 P10114

O43765 P10124

P10144 P10124

P13611 P10124

P14222 P10124

P14780 P10124

Q6PGN9 P10124

Q6UXH1 P10124

Q8NHQ1 P10124

Q9NRR5 P10124

P10124 P10144

P14222 P10144

Q15075 P10144

P25025 P10145

P46379 P10145

P10147 P10147

O95166 P10155

P60520 P10155

Q5U5Q3 P10155

Q9H0R8 P10155

P02760 P10176

O14917 P10242

O15394 P10242

O75376 P10242

P20962 P10242

P24311 P10242

P29590 P10242

P61956 P10242

P63165 P10242

Q00994 P10242

Q15759 P10242

Q5TI25 P10242

Q5TKA1 P10242

Q8N3J5 P10242

Q96RN5 P10242

Q9NPC6 P10242

Q9UBE8 P10242

Q5TKA1 P10243

Q6MZP7 P10243

Q96GY3 P10243

O00716 P10244

Q52LA3 P10244

Q5TKA1 P10244

Q6MZP7 P10244

Q96GY3 P10244

P15822 P10253

P52630 P10253

Q09472 P10253

P02647 P10266

O14595 P10275

O14672 P10275

O14976 P10275

O95251 P10275

O96028 P10275

P10275 P10275

P11308 P10275

P14921 P10275

P20711 P10275

P20794 P10275

P35222 P10275

P35269 P10275

P55317 P10275

P56524 P10275

P60709 P10275

P63165 P10275

P78543 P10275

Q00987 P10275

Q06830 P10275

Q12778 P10275

Q13772 P10275

Q15596 P10275

Q15788 P10275

Q15797 P10275

Q16665 P10275

Q16666 P10275

Q92793 P10275

Q92993 P10275

Q99497 P10275

Q9NSA3 P10275

Q9UBS8 P10275

Q9UER7 P10275

Q9UQ80 P10275

Q9Y252 P10275

Q9Y6Q9 P10275

O75376 P10276

P02751 P10276

P14866 P10276

P28702 P10276

P48443 P10276

P48552 P10276

P50148 P10276

Q05086 P10276

Q12796 P10276

Q13573 P10276

Q14254 P10276

Q15648 P10276

Q15788 P10276

Q15910 P10276

Q2M1K9 P10276

Q71SY5 P10276

Q8IXJ9 P10276

Q8IZD2 P10276

Q96EB6 P10276

Q9UIS9 P10276

Q9UPP1 P10276

Q9Y6Q9 P10276

P38606 P10301

O43187 P10398

O43615 P10398

O94906 P10398

O95848 P10398

P00966 P10398

P01112 P10398

P08238 P10398

P14618 P10398

P15056 P10398

P27986 P10398

P31327 P10398

P36507 P10398

P40692 P10398

P53611 P10398

P58753 P10398

P62070 P10398

P63104 P10398

P67870 P10398

Q02750 P10398

Q04917 P10398

Q12805 P10398

Q16543 P10398

Q3ZCQ8 P10398

Q53G59 P10398

Q8IXH7 P10398

Q8NE63 P10398

Q92985 P10398

Q96KB5 P10398

Q9BTC0 P10398

Q9UNS2 P10398

O15264 P10412

O95503 P10412

O95758 P10412

P00533 P10412

P19320 P10412

P51858 P10412

P62805 P10412

Q00005 P10412

Q9UGN5 P10412

Q9Y468 P10412

O15151 P10415

O15304 P10415

O43521 P10415

P04637 P10415

P10415 P10415

P22736 P10415

P28482 P10415

P30405 P10415

P36873 P10415

P38398 P10415

P51572 P10415

P55957 P10415

P62140 P10415

P63098 P10415

P67775 P10415

Q07812 P10415

Q07817 P10415

Q13323 P10415

Q13625 P10415

Q14318 P10415

Q14457 P10415

Q92934 P10415

Q9BXH1 P10415

Q9C000 P10415

Q9C0C7 P10415

Q9H2V7 P10415

Q13794 P10415

O43765 P10451

P02647 P10451

P12955 P10451

P46379 P10451

Q8WYA6 P10451

Q9NR12 P10451

Q9NRR5 P10451

O00330 P10515

O94806 P10515

P11177 P10515

Q9GZQ8 P10515

Q9H0R8 P10515

Q9H492 P10515

P32121 P10523

P49407 P10523

P00533 P10586

P10586 P10586

P46778 P10588

P55209 P10588

P83916 P10588

Q13573 P10588

Q9UIF8 P10588

Q9Y272 P10588

O60925 P10589

Q00403 P10589

O14879 P10599

O43707 P10599

O95166 P10599

O95758 P10599

P00533 P10599

P01106 P10599

P04406 P10599

P19320 P10599

P32119 P10599

P60520 P10599

Q06830 P10599

Q16621 P10599

Q92905 P10599

Q96D09 P10599

Q99683 P10599

Q99836 P10599

Q9GZQ8 P10599

Q9H0R8 P10599

Q9H3M7 P10599

Q9H492 P10599

P10600 P10600

P31431 P10600

P37173 P10600

Q03167 P10600

O95758 P10606

P00403 P10606

P36406 P10606

Q5VWN6 P10606

Q8IYA8 P10606

O95166 P10619

P60520 P10619

Q9H0R8 P10619

P04626 P10620

P36776 P10632

Q9H0R8 P10632

Q9H492 P10632

Q9Y2H9 P10632

O15428 P10636

O95793 P10636

P05067 P10636

P06241 P10636

P10636 P10636

P43004 P10636

P49840 P10636

P49841 P10636

P63104 P10636

Q96RG2 P10636

Q9NY61 P10636

O00141 P10636

O00499 P10636

P23443 P10636

P51812 P10636

Q14203 P10636

Q5S007 P10636

P17612 P10644

P22694 P10644

P35250 P10644

P49841 P10644

Q13627 P10644

Q9H0R8 P10644

Q9Y463 P10644

P02768 P10720

P19174 P10721

P21583 P10721

P29350 P10721

P46108 P10721

Q05209 P10721

Q12913 P10721

Q14451 P10721

Q92729 P10721

O75791 P10747

P27986 P10747

P33681 P10747

Q04759 P10747

P19320 P10768

Q13547 P10768

Q9BW61 P10768

A0JLT2 P10809

O95166 P10809

P00533 P10809

P01023 P10809

P01106 P10809

P03372 P10809

P04637 P10809

P12830 P10809

P13569 P10809

P15336 P10809

P17152 P10809

P19320 P10809

P30153 P10809

P30154 P10809

P38398 P10809

P43351 P10809

P49789 P10809

P60520 P10809

P60709 P10809

P62993 P10809

P63104 P10809

Q00005 P10809

Q13573 P10809

Q14197 P10809

Q16659 P10809

Q8N0X7 P10809

Q96EB6 P10809

Q99459 P10809

Q9BXK5 P10809

Q9BXN2 P10809

Q9GZQ8 P10809

Q9H0R8 P10809

Q9NTG7 P10809

Q9NWA0 P10809

Q9UKV8 P10809

Q9UL18 P10809

Q15796 P10826

Q8IXJ9 P10826

Q9BZL6 P10826

Q14686 P10827

Q15648 P10827

Q4VCS5 P10827

Q8TAP6 P10827

Q92949 P10827

P55345 P10828

Q14686 P10828

Q96EY9 P10828

O75376 P10828

P15336 P10828

Q9Y618 P10828

Q9Y6Q9 P10828

O14901 P10909

P01100 P10909

P01857 P10909

P01876 P10909

P02647 P10909

P04150 P10909

P16104 P10909

P30101 P10909

P37231 P10909

Q9BWU1 P10909

Q9NRI5 P10909

Q9UKE5 P10909

Q9Y3Q8 P10909

Q07817 P10909

P16333 P10912

P17706 P10912

P18031 P10912

P23467 P10912

P26045 P10912

P43378 P10912

Q12913 P10912

Q14686 P10912

Q9HD43 P10912

P47897 P10914

Q8IYT8 P10914

P35125 P10916

P10997 P10997

O00716 P11021

O15264 P11021

O94868 P11021

O95166 P11021

P00533 P11021

P01023 P11021

P02751 P11021

P03372 P11021

P04049 P11021

P04626 P11021

P04637 P11021

P10809 P11021

P13569 P11021

P15056 P11021

P19320 P11021

P21860 P11021

P25054 P11021

P32121 P11021

P48039 P11021

P49286 P11021

P49407 P11021

P60520 P11021

P62993 P11021

Q00005 P11021

Q03135 P11021

Q0P6H9 P11021

Q12772 P11021

Q13131 P11021

Q13573 P11021

Q14790 P11021

Q15051 P11021

Q16543 P11021

Q30201 P11021

Q6E0U4 P11021

Q6T424 P11021

Q8N0X7 P11021

Q92905 P11021

Q96EB6 P11021

Q96IZ0 P11021

Q99459 P11021

Q9GZQ8 P11021

Q9H0R8 P11021

Q9H3D4 P11021

Q9H492 P11021

Q9HCK5 P11021

Q9NTG7 P11021

Q9UBS4 P11021

O15230 P11047

O60749 P11047

O95295 P11047

P24043 P11047

P25391 P11047

Q16363 P11047

Q16787 P11047

Q6VMQ6 P11047

Q96SB4 P11047

P29350 P11049

P43405 P11049

P48736 P11049

O95295 P11055

P63104 P11055

Q15435 P11055

P83916 P11117

O95758 P11137

P62993 P11137

Q13951 P11137

Q9P0W5 P11137

O75190 P11142

O95166 P11142

O95816 P11142

P00338 P11142

P00533 P11142

P01106 P11142

P03372 P11142

P05067 P11142

P05412 P11142

P06733 P11142

P07900 P11142

P08473 P11142

P09467 P11142

P0CG47 P11142

P15056 P11142

P19320 P11142

P27348 P11142

P30408 P11142

P32121 P11142

P34932 P11142

P36873 P11142

P41220 P11142

P42771 P11142

P43351 P11142

P49407 P11142

P53041 P11142

P60520 P11142

P60709 P11142

P62993 P11142

P78364 P11142

P84090 P11142

Q02750 P11142

Q04917 P11142

Q08752 P11142

Q13573 P11142

Q13620 P11142

Q14527 P11142

Q15051 P11142

Q15628 P11142

Q53FT3 P11142

Q5S007 P11142

Q6PJG9 P11142

Q8IZP0 P11142

Q8TDR0 P11142

Q92731 P11142

Q99459 P11142

Q99933 P11142

Q9BSM1 P11142

Q9BXA6 P11142

Q9GZQ8 P11142

Q9GZX7 P11142

Q9H0R8 P11142

Q9H492 P11142

Q9HCK5 P11142

Q9HCU9 P11142

Q9NQ11 P11142

Q9NY33 P11142

Q9UNE7 P11142

Q00005 P11142

P07738 P11161

P13686 P11161

P51610 P11161

Q6DKI1 P11161

Q9H910 P11161

Q9Y3C7 P11161

O00716 P11166

P11166 P11166

P15336 P11166

P19320 P11166

Q7L5N1 P11166

O43889 P11169

O00299 P11171

O14818 P11171

O15067 P11171

O60493 P11171

O75643 P11171

O75694 P11171

O75964 P11171

P00491 P11171

P00492 P11171

P00505 P11171

P02751 P11171

P04080 P11171

P04921 P11171

P07951 P11171

P10599 P11171

P10606 P11171

P12004 P11171

P12882 P11171

P13693 P11171

P14174 P11171

P16152 P11171

P20618 P11171

P22061 P11171

P22234 P11171

P22307 P11171

P24534 P11171

P24666 P11171

P25786 P11171

P26640 P11171

P27797 P11171

P28074 P11171

P30084 P11171

P30086 P11171

P31948 P11171

P31949 P11171

P35080 P11171

P35579 P11171

P37802 P11171

P40939 P11171

P41567 P11171

P42704 P11171

P42771 P11171

P43487 P11171

P43686 P11171

P48047 P11171

P49411 P11171

P49458 P11171

P49720 P11171

P51148 P11171

P54819 P11171

P55769 P11171

P57088 P11171

P58546 P11171

P60900 P11171

P61006 P11171

P61019 P11171

P61247 P11171

P61289 P11171

P61457 P11171

P61604 P11171

P61803 P11171

P61923 P11171

P62942 P11171

P63167 P11171

P68036 P11171

P68402 P11171

P99999 P11171

Q04637 P11171

Q08211 P11171

Q13404 P11171

Q13526 P11171

Q13642 P11171

Q15369 P11171

Q15691 P11171

Q15907 P11171

Q53Y06 P11171

Q5SQT9 P11171

Q6FGG2 P11171

Q6FGH5 P11171

Q96AG4 P11171

Q99436 P11171

Q99623 P11171

Q9BQC6 P11171

Q9BRA2 P11171

Q9GZT3 P11171

Q9HB71 P11171

Q9NP97 P11171

Q9UI30 P11171

Q9Y237 P11171

Q9Y241 P11171

Q9Y5L4 P11171

O43852 P11172

P29474 P11172

P36957 P11172

P62273 P11172

Q70EL3 P11172

Q86WZ6 P11172

Q99459 P11172

Q9UKI2 P11172

O60925 P11177

O95166 P11177

P01241 P11177

P02751 P11177

P05556 P11177

P08559 P11177

P10515 P11177

P12319 P11177

P18754 P11177

P20073 P11177

P61224 P11177

P62879 P11177

Q15714 P11177

Q9GZQ8 P11177

P09913 P11182

P67809 P11182

Q14197 P11182

Q9BV57 P11182

Q9H9G7 P11182

Q9HCK5 P11182

Q9Y5T5 P11182

P05107 P11215

Q9H2X3 P11215

O76094 P11217

P02545 P11217

P50570 P11217

Q13326 P11217

Q5VU43 P11217

Q8NFD5 P11217

Q96HW7 P11217

Q9BQ89 P11217

Q9BYV2 P11217

Q9BYV6 P11217

P67809 P11226

Q07954 P11226

Q7L5N1 P11230

P01112 P11233

P24385 P11233

P30154 P11233

P62158 P11233

Q14457 P11234

Q15311 P11234

O14908 P11274

O14924 P11274

O96018 P11274

P01106 P11274

P04637 P11274

P13569 P11274

P19784 P11274

P36402 P11274

P53618 P11274

P62993 P11274

Q13432 P11274

Q13588 P11274

Q38SD2 P11274

Q5T2W1 P11274

Q8TAF3 P11274

Q96CW1 P11274

Q96ID5 P11274

Q9H2K2 P11274

Q9UDY2 P11274

O75386 P11277

Q13813 P11277

P04062 P11279

P38606 P11279

Q5VZM2 P11279

Q92542 P11279

Q9ULZ3 P11279

B2Y833 P11308

P09874 P11308

P10275 P11308

P13010 P11308

P25791 P11308

P78527 P11308

Q15047 P11308

P08238 P11309

P39019 P11309

P51813 P11309

Q9UNQ0 P11309

P11310 P11310

Q13547 P11310

Q70EL3 P11310

Q9H0R8 P11310

Q9Y2K6 P11310

P01857 P11362

P05230 P11362

P08908 P11362

P09038 P11362

P11362 P11362

P12830 P11362

P19174 P11362

P21860 P11362

P23352 P11362

P27986 P11362

P35222 P11362

P46108 P11362

P46934 P11362

P50750 P11362

Q86Z14 P11362

Q8IVI9 P11362

P09038 P113624

O94921 P11387

O95166 P11387

P00519 P11387

P01106 P11387

P03372 P11387

P13010 P11387

P19320 P11387

P19338 P11387

P19525 P11387

P51858 P11387

P60520 P11387

Q14197 P11387

Q15796 P11387

Q8IW19 P11387

Q92547 P11387

Q99801 P11387

Q9H0R8 P11387

Q9UKV0 P11387

Q9UQ80 P11387

Q9Y6K5 P11387

P01106 P11388

P03372 P11388

P09874 P11388

P11387 P11388

P13010 P11388

P15884 P11388

P16104 P11388

P19320 P11388

P35222 P11388

P78527 P11388

Q9NR30 P11388

P68400 P11388

P02751 P11413

P04792 P11413

Q07002 P11441

Q13573 P11441

Q92995 P11441

Q99459 P11441

Q9BVJ6 P11464

P04637 P11473

P08603 P11473

P14314 P11473

P18146 P11473

P19793 P11473

P26045 P11473

P28702 P11473

P42785 P11473

P84022 P11473

Q09472 P11473

Q13573 P11473

Q14686 P11473

Q15648 P11473

Q7LFL8 P11473

Q969F2 P11473

Q96SB4 P11473

P11766 P11474

P54253 P11474

A6NIH7 P11488

Q13432 P11488

P09914 P11498

Q00005 P11498

P01023 P11511

O60941 P11532

P02768 P11532

P08727 P11532

P49407 P11532

Q13884 P11532

Q14118 P11532

Q9NRI5 P11532

Q9Y4J8 P11532

Q01484 P11532

O95166 P11586

P08238 P11586

P08473 P11586

P19320 P11586

Q8NCN4 P11586

Q9H0R8 P11586

Q9H492 P11586

P22612 P11597

Q01844 P11597

P19174 P11678

P27986 P11678

O95716 P11712

Q16543 P11712

Q9Y2H9 P11712

P01344 P11717

P02751 P11717

P11717 P11717

P19320 P11717

P54253 P11717

Q15075 P11717

Q6VY07 P11717

Q70EL3 P11717

Q9NZ52 P11717

Q9UJY4 P11717

Q9UJY5 P11717

P04004 P11766

P11766 P11766

P27540 P11766

P67870 P11766

Q12873 P11766

Q13547 P11766

Q8N2U9 P11766

Q8NBN7 P11801

O75694 P11802

P01106 P11802

P04632 P11802

P07948 P11802

P08151 P11802

P14618 P11802

P20073 P11802

P24385 P11802

P28698 P11802

P28749 P11802

P30153 P11802

P30154 P11802

P30279 P11802

P30281 P11802

P32121 P11802

P35606 P11802

P37840 P11802

P38936 P11802

P42771 P11802

P42772 P11802

P42773 P11802

P46379 P11802

P47897 P11802

P49736 P11802

P49918 P11802

P51693 P11802

P52209 P11802

P55273 P11802

P57678 P11802

P62136 P11802

P63208 P11802

Q02556 P11802

Q04323 P11802

Q05516 P11802

Q08999 P11802

Q12766 P11802

Q12996 P11802

Q14493 P11802

Q14683 P11802

Q15047 P11802

Q15555 P11802

Q16543 P11802

Q6UB98 P11802

Q8IZC7 P11802

Q8IZT6 P11802

Q8N720 P11802

Q96PU4 P11802

Q96T76 P11802

Q99828 P11802

Q99956 P11802

Q9BQA1 P11802

Q9C0J8 P11802

Q9H0R8 P11802

Q9H4L4 P11802

Q9H4Z2 P11802

Q9NP79 P11802

Q9NS64 P11802

Q9P2Y4 P11802

Q9UDX5 P11802

Q9UJU6 P11802

Q9UQE7 P11802

P15172 P11831

P15173 P11831

Q14011 P11831

Q8IZQ8 P11831

Q969V6 P11831

Q9ULH7 P11831

P02751 P11908

O95190 P11926

P43307 P11926

P67870 P11926

O00571 P11940

O14879 P11940

O15264 P11940

O43432 P11940

O75554 P11940

O95166 P11940

O95503 P11940

O95793 P11940

P03372 P11940

P09913 P11940

P09914 P11940

P16104 P11940

P19320 P11940

P32121 P11940

P35568 P11940

P38919 P11940

P49760 P11940

P49761 P11940

P60520 P11940

P61981 P11940

P62993 P11940

P67809 P11940

P78362 P11940

Q01081 P11940

Q04637 P11940

Q13573 P11940

Q14103 P11940

Q14106 P11940

Q14240 P11940

Q15366 P11940

Q58A45 P11940

Q8IYD1 P11940

Q8NDV7 P11940

Q8WWM7 P11940

Q92597 P11940

Q92900 P11940

Q96Q15 P11940

Q96SB4 P11940

Q99700 P11940

Q9BPZ3 P11940

Q9BWF3 P11940

Q9H074 P11940

Q9H0R8 P11940

Q9H492 P11940

Q9HCJ0 P11940

Q9HCK5 P11940

Q9UKV8 P11940

Q9UL18 P11940

Q9UPQ9 P11940

Q9Y5S9 P11940

P84022 P11940

Q00005 P11940

O15304 P12004

P00558 P12004

P00918 P12004

P02751 P12004

P04406 P12004

P08473 P12004

P08729 P12004

P12004 P12004

P15336 P12004

P18669 P12004

P28340 P12004

P36873 P12004

P38936 P12004

P39748 P12004

P42771 P12004

P49005 P12004

P49918 P12004

P51587 P12004

P58753 P12004

Q05048 P12004

Q13111 P12004

Q15054 P12004

Q15843 P12004

Q16658 P12004

Q99728 P12004

Q9GZQ8 P12004

Q9H160 P12004

Q9H211 P12004

Q9H492 P12004

Q9HCU8 P12004

Q9NTI5 P12004

P00519 P12018

P60520 P12035

Q9BQ83 P12035

Q9H0R8 P12035

P62993 P12036

Q92731 P12036

P62993 P12074

Q15843 P12081

Q9UKE5 P12081

P02458 P12107

P20908 P12107

P12109 P12110

Q02221 P12110

Q9NRI5 P12110

O75923 P12111

P12109 P12111

O00716 P12235

P62993 P12235

Q14197 P12235

Q5S007 P12235

O00716 P12236

O14798 P12236

O95166 P12236

P01023 P12236

P05060 P12236

P05141 P12236

P08670 P12236

P16104 P12236

P62993 P12236

P63244 P12236

Q04917 P12236

Q0P6H9 P12236

Q12873 P12236

Q15796 P12236

Q9BQ66 P12236

Q9GZQ8 P12236

Q9H492 P12236

Q9NTM9 P12236

Q9UKV8 P12236

Q9UL18 P12236

Q9Y6E7 P12236

O75381 P12268

P02751 P12268

P08473 P12268

P24941 P12268

P53779 P12268

P60520 P12268

P62993 P12268

Q8N2W9 P12268

Q9H0R8 P12268

O95931 P12270

P01100 P12270

P02751 P12270

P19320 P12270

P25054 P12270

Q5S007 P12270

Q99459 P12270

Q9H492 P12270

Q9UKV0 P12270

Q9Y6R4 P12270

P53350 P12272

Q5W0Q7 P12273

Q92731 P12273

Q9BWU1 P12273

Q9H0R8 P12273

Q9UHP3 P12273

O15264 P12277

O95166 P12277

P40692 P12277

P43351 P12277

P60520 P12277

Q13418 P12277

Q15051 P12277

Q5W0Q7 P12277

Q8N6R1 P12277

Q8TDR0 P12277

Q96DX5 P12277

Q9H492 P12277

Q9Y4E8 P12277

Q13094 P12314

P02741 P12318

P04637 P12429

P15336 P12429

Q13432 P12429

Q8IWV7 P12429

Q96ID5 P12429

P01023 P12532

P56537 P12532

Q15051 P12532

P12544 P12544

O00238 P12643

P12643 P12643

P36894 P12643

Q13253 P12643

O95405 P12644

P36894 P12644

Q8TEU8 P12644

O95166 P12694

P21953 P12694

Q9H0R8 P12694

P84022 P12755

Q13485 P12755

Q15796 P12755

Q9UBE8 P12755

Q9Y463 P12755

O00257 P12757

O60437 P12757

O75376 P12757

O75925 P12757

O76094 P12757

P10586 P12757

P12956 P12757

P14543 P12757

P15104 P12757

P23142 P12757

P48307 P12757

P63279 P12757

P78317 P12757

P78344 P12757

Q12873 P12757

Q13569 P12757

Q14112 P12757

Q14789 P12757

Q15036 P12757

Q15796 P12757

Q15916 P12757

Q16557 P12757

Q4LDE5 P12757

Q7L2E3 P12757

Q8N2W9 P12757

Q8N3C0 P12757

Q8N4S1 P12757

Q8WUA8 P12757

Q96C36 P12757

Q9H5J0 P12757

Q9NQ87 P12757

Q9UBW7 P12757

Q9UHP3 P12757

Q9UJ78 P12757

Q9UK41 P12757

Q9UNN5 P12757

Q9UPN9 P12757

Q9Y5J3 P12757

Q9Y6X2 P12757

O00330 P12814

O00555 P12814

P03372 P12814

P08238 P12814

P08567 P12814

P12814 P12814

P12931 P12814

P15336 P12814

P25786 P12814

P43364 P12814

P49768 P12814

P52292 P12814

P62993 P12814

Q06187 P12814

Q13426 P12814

Q15942 P12814

Q8N2W9 P12814

Q8TDR0 P12814

Q8WZ42 P12814

Q92731 P12814

Q9H0R8 P12814

Q9NP98 P12814

Q9Y6R0 P12814

P02751 P12829

P02768 P12829

P19320 P12829

O607169 P12830

O60716 P12830

P00533 P12830

P02511 P12830

P11362 P12830

P12931 P12830

P13569 P12830

P15151 P12830

P30260 P12830

P35222 P12830

P46937 P12830

P62136 P12830

P63244 P12830

Q15139 P12830

Q68CZ2 P12830

Q6IQ23 P12830

Q75N03 P12830

Q7Z6J6 P12830

Q92597 P12830

Q96CW1 P12830

Q9BVG8 P12830

O95793 P12882

P49407 P12882

Q9UIG0 P12882

O75386 P12883

O95295 P12883

P63104 P12883

Q92731 P12883

Q9NRI5 P12883

O14490 P12931

O15455 P12931

O43909 P12931

O60493 P12931

O95886 P12931

P00519 P12931

P00533 P12931

P01112 P12931

P03372 P12931

P04626 P12931

P05023 P12931

P07550 P12931

P07948 P12931

P08581 P12931

P09619 P12931

P09848 P12931

P0CG48 P12931

P10301 P12931

P10636 P12931

P12814 P12931

P12830 P12931

P12931 P12931

P15586 P12931

P15941 P12931

P16284 P12931

P17302 P12931

P18031 P12931

P18433 P12931

P20774 P12931

P20810 P12931

P21145 P12931

P22681 P12931

P23743 P12931

P23759 P12931

P26373 P12931

P27635 P12931

P27986 P12931

P28340 P12931

P33151 P12931

P35968 P12931

P40763 P12931

P41240 P12931

P43699 P12931

P46013 P12931

P46527 P12931

P48023 P12931

P49023 P12931

P49916 P12931

P50570 P12931

P55196 P12931

P61978 P12931

P68400 P12931

P78329 P12931

P78345 P12931

Q01973 P12931

Q03135 P12931

Q05193 P12931

Q05397 P12931

Q05655 P12931

Q05996 P12931

Q06124 P12931

Q07666 P12931

Q07889 P12931

Q07890 P12931

Q13087 P12931

Q13177 P12931

Q13444 P12931

Q13905 P12931

Q14118 P12931

Q14185 P12931

Q14289 P12931

Q15027 P12931

Q15036 P12931

Q16832 P12931

Q4V348 P12931

Q68CZ2 P12931

Q7Z7K6 P12931

Q8IWT3 P12931

Q8IZD9 P12931

Q8NF50 P12931

Q8TB24 P12931

Q8TBB1 P12931

Q8WUM4 P12931

Q92918 P12931

Q92988 P12931

Q9BWW9 P12931

Q9BYB0 P12931

Q9C0H9 P12931

Q9H1R2 P12931

Q9H5V8 P12931

Q9H9L3 P12931

Q9HCU4 P12931

Q9NRJ4 P12931

Q9NZM4 P12931

Q9NZV5 P12931

Q9P1A6 P12931

Q9UL51 P12931

Q9ULH1 P12931

Q9UPX8 P12931

Q9Y2D5 P12931

Q9Y2J2 P12931

Q9Y6K9 P12931

B2Y833 P12956

O00716 P12956

O15264 P12956

O15530 P12956

O60613 P12956

O75528 P12956

O75554 P12956

O95166 P12956

O95758 P12956

P01106 P12956

P02751 P12956

P04637 P12956

P09629 P12956

P09874 P12956

P10644 P12956

P11308 P12956

P13010 P12956

P13569 P12956

P14921 P12956

P17096 P12956

P17947 P12956

P19320 P12956

P20366 P12956

P20827 P12956

P20936 P12956

P22415 P12956

P32121 P12956

P38432 P12956

P42858 P12956

P46783 P12956

P49368 P12956

P49715 P12956

P50549 P12956

P51828 P12956

P51858 P12956

P53618 P12956

P60520 P12956

P62070 P12956

P62324 P12956

P62993 P12956

P68400 P12956

P78527 P12956

P83916 P12956

P84022 P12956

Q07812 P12956

Q08752 P12956

Q12851 P12956

Q12929 P12956

Q13185 P12956

Q13573 P12956

Q14191 P12956

Q15118 P12956

Q8IW19 P12956

Q96EB6 P12956

Q96HA7 P12956

Q96KM6 P12956

Q96P48 P12956

Q99728 P12956

Q9GZQ8 P12956

Q9H0R6 P12956

Q9H0R8 P12956

Q9NPJ4 P12956

Q9NQB0 P12956

Q9NYB0 P12956

Q9UKV8 P12956

Q9UL18 P12956

Q9UMQ6 P12956

Q9Y6B2 P12956

Q9UPN9 P12980

B2Y833 P13010

O00716 P13010

O95166 P13010

O95758 P13010

P01106 P13010

P02751 P13010

P08473 P13010

P09629 P13010

P09874 P13010

P11308 P13010

P11388 P13010

P12830 P13010

P12956 P13010

P14921 P13010

P16104 P13010

P17947 P13010

P19320 P13010

P22415 P13010

P32121 P13010

P38432 P13010

P49407 P13010

P49715 P13010

P50549 P13010

P51858 P13010

P60520 P13010

P78527 P13010

Q12800 P13010

Q14191 P13010

Q8IW19 P13010

Q96HA7 P13010

Q9GZQ8 P13010

Q9H9Q4 P13010

Q9NQB0 P13010

Q9NYB0 P13010

P03372 P13056

P00395 P13073

P00403 P13073

P10809 P13073

Q02221 P13073

Q07820 P13073

Q14197 P13073

Q9BVV7 P13073

Q9UJS0 P13073

Q9Y2R0 P13073

P61224 P13196

Q8WTR2 P13196

Q96L91 P13196

Q9H7E9 P13196

Q9Y2S7 P13196

P07359 P13224

P19320 P13224

P16871 P13232

P13236 P13236

P14735 P13236

P51681 P13236

P18754 P13378

O60383 P13385

P03950 P13385

P23142 P13385

P35052 P13385

P46379 P13385

P53680 P13385

Q5UIP0 P13385

Q7L5N1 P13385

Q9Y285 P13385

P00441 P13473

P07550 P13473

P11142 P13473

P38606 P13473

P51798 P13473

Q5S007 P13473

Q5VZM2 P13473

P01106 P13489

P02751 P13489

P03950 P13489

P07998 P13489

P09874 P13489

Q15051 P13489

Q6URH7 P13489

Q8IY92 P13489

Q96KP6 P13489

Q9UN86 P13489

O14793 P13497

P35348 P13497

Q9H2X0 P13497

P01106 P13498

P14598 P13498

P50416 P13498

Q15080 P13498

Q8NFA2 P13498

P13501 P13501

P22732 P13501

P27487 P13501

P51681 P13501

Q15654 P13501

Q6P2E9 P13501

P27986 P13521

Q9P0W5 P13521

Q9UMX0 P13521

P04179 P13535

Q14324 P13535

O14745 P13569

P05787 P13569

P11274 P13569

P13569 P13569

P30153 P13569

P51572 P13569

Q15599 P13569

Q5T2W1 P13569

Q96RN1 P13569

Q99942 P13569

Q9BUN8 P13569

Q9H8Y8 P13569

P09936 P13591

P54253 P13591

Q15382 P13591

P13591 P13591

Q9NQ11 P13598

P02751 P13612

P05556 P13612

P07948 P13612

P13612 P13612

P19320 P13612

P26010 P13612

P49023 P13612

O43482 P13631

O60504 P13631

Q2M1K9 P13631

Q86X29 P13631

Q9H609 P13631

Q9NYA1 P13631

Q9Y217 P13631

Q9UKV8 P13637

O15264 P13639

O95166 P13639

P02751 P13639

P04637 P13639

P08238 P13639

P15336 P13639

P19320 P13639

P27695 P13639

P30153 P13639

P30154 P13639

P32121 P13639

P40692 P13639

P42771 P13639

P54725 P13639

P60520 P13639

P61244 P13639

P62993 P13639

Q07002 P13639

Q09472 P13639

Q15843 P13639

Q16539 P13639

Q8IZL9 P13639

Q8NE63 P13639

Q92597 P13639

Q92900 P13639

Q96Q15 P13639

Q9GZQ8 P13639

Q9H0R8 P13639

Q9H492 P13639

Q9NRI5 P13639

P58062 P13640

O95166 P13645

P01857 P13645

P01876 P13645

P02751 P13645

P02768 P13645

P08473 P13645

P12931 P13645

P13569 P13645

P19320 P13645

P46781 P13645

P60520 P13645

P84022 P13645

Q00005 P13645

Q07157 P13645

Q8TDR0 P13645

Q9GZQ8 P13645

Q9H0R8 P13645

Q9H492 P13645

O95166 P13646

P02768 P13646

P25054 P13646

P60520 P13646

Q9GZQ8 P13646

Q9H0R8 P13646

Q9H492 P13646

O95166 P13647

P00533 P13647

P01857 P13647

P02751 P13647

P02768 P13647

P18054 P13647

P60520 P13647

Q00005 P13647

Q06187 P13647

Q9GZQ8 P13647

Q9H0R8 P13647

Q9H3D4 P13647

Q9H492 P13647

P15336 P13667

P16104 P13667

Q86YB8 P13667

P00519 P13671

P06241 P13671

P16333 P13671

P62993 P13671

Q15020 P13671

P01106 P13674

Q92985 P13674

Q969S8 P13674

O14737 P13686

P15927 P13686

P62714 P13686

Q05516 P13686

Q8N357 P13686

Q96DI7 P13686

P13688 P13688

P29353 P13688

P40199 P13688

Q14002 P13688

Q16568 P13688

P04637 P13693

P05023 P13693

P05386 P13693

Q15714 P13693

Q15796 P13693

Q7Z6E9 P13693

Q96EP1 P13693

P40189 P13725

P42702 P13725

P08709 P13726

Q12873 P13727

Q5RL73 P13727

P28067 P13760

O95671 P13762

P02751 P13796

Q99700 P13796

O15264 P13797

O95758 P13797

P62993 P13797

Q8IY92 P13797

Q99700 P13797

Q9H0R8 P13797

Q9H3D4 P13797

P13798 P13798

P53990 P13798

Q0P6H9 P13798

Q9GZZ9 P13798

P01106 P13804

P60953 P13804

Q14197 P13804

Q9BSB4 P13804

Q9BW61 P13804

Q9GZZ9 P13804

O15069 P13805

O75386 P13805

P06241 P13805

P08670 P13805

P09493 P13805

P13725 P13805

P13805 P13805

P15622 P13805

P26641 P13805

P29622 P13805

P51116 P13805

P54296 P13805

Q13136 P13805

Q15834 P13805

Q16891 P13805

Q8IUR7 P13805

Q92993 P13805

Q969R2 P13805

Q96E35 P13805

Q96S99 P13805

Q99963 P13805

Q9H5H4 P13805

Q9UGU5 P13805

Q9UNN5 P13805

Q9Y2I6 P13805

Q9Y2L8 P13805

Q9Y2Y6 P13805

Q9Y328 P13805

O15488 P13807

O43741 P13807

O95166 P13807

P46976 P13807

P54646 P13807

P60520 P13807

Q14457 P13807

Q8IY92 P13807

Q96G74 P13807

Q9GZQ8 P13807

Q9H0R8 P13807

Q9P2Y5 P13807

Q9Y371 P13807

P17612 P13861

P22694 P13861

Q9H492 P13861

P00533 P13866

O15273 P13929

P04075 P13929

P11277 P13929

P13929 P13929

P14618 P13929

P15924 P13929

P36871 P13929

Q13326 P13929

Q14203 P13929

Q15051 P13929

Q8NCE2 P13929

Q8WZ42 P13929

P02458 P13942

P20073 P13942

Q13526 P13942

Q16832 P13942

Q8TBZ3 P13942

O00124 P13984

O94763 P13984

O94985 P13984

O95573 P13984

P05067 P13984

P17096 P13984

P46776 P13984

P55209 P13984

Q00403 P13984

Q14966 P13984

Q8WW35 P13984

Q9NT62 P13984

Q9UHV9 P13984

Q9UKW4 P13984

P00533 P13987

P13569 P13987

P62993 P13987

Q03135 P13987

Q13485 P13987

P14923 P13994

P49910 P13994

P68104 P13994

Q05516 P13994

Q9UJ70 P13995

P46379 P14091

O00151 P14136

O75791 P14136

O95166 P14136

O95758 P14136

P02768 P14136

P05067 P14136

P08670 P14136

P60520 P14136

P62993 P14136

Q15796 P14136

Q5T2W1 P14136

Q6ZU52 P14136

Q8TAP6 P14136

Q92731 P14136

Q96HA1 P14136

Q96HL8 P14136

Q9BVR6 P14136

Q9GZQ8 P14136

Q9H0R8 P14136

Q9H492 P14136

Q9H4K1 P14136

Q9NR12 P14136

Q9Y6K9 P14136

O43521 P14174

O95166 P14174

P19320 P14174

P60520 P14174

Q00994 P14174

Q7L5N1 P14174

Q92905 P14174

Q9GZQ8 P14174

Q9H0R8 P14174

Q9H492 P14174

Q9H8Y8 P14174

O43765 P14209

Q9NRR5 P14209

Q9UMX0 P14209

P08581 P14210

P16070 P14210

P14210 P14210

P10144 P14222

P14222 P14222

P11021 P14314

P16104 P14314

P08238 P14316

P16298 P14316

P85037 P14316

O43516 P14317

P07948 P14317

P13569 P14317

P42768 P14317

Q96PU8 P14317

Q9H788 P14317

Q99816 P14324

B3KSH4 P14373

O14933 P14373

P35226 P14373

P36406 P14373

P49459 P14373

P51668 P14373

P60228 P14373

P61077 P14373

P62837 P14373

P62877 P14373

P63279 P14373

Q05516 P14373

Q14511 P14373

Q15040 P14373

Q8TAU3 P14373

Q96BQ3 P14373

Q96JG8 P14373

Q9BUH8 P14373

Q9BYV6 P14373

Q9BZY9 P14373

Q9H832 P14373

Q9NNX1 P14373

Q9NUQ8 P14373

Q9NX04 P14373

Q9UBN7 P14373

Q9UGJ1 P14373

Q9UJ55 P14373

P00403 P14406

Q9UJS0 P14406

P14416 P14416

P29274 P14416

P62158 P14416

Q01959 P14416

Q96SB3 P14416

Q14194 P14555

P04899 P14598

P05771 P14598

P13498 P14598

P17252 P14598

P19878 P14598

P60709 P14598

Q04206 P14598

Q15080 P14598

Q8IZP0 P14598

Q9C026 P14598

O15264 P14618

O95059 P14618

P01106 P14618

P02751 P14618

P08238 P14618

P15336 P14618

P16104 P14618

P18754 P14618

P19320 P14618

P32121 P14618

P49407 P14618

P53355 P14618

P60520 P14618

P62993 P14618

Q00987 P14618

Q06710 P14618

Q8TDR0 P14618

Q8WWI1 P14618

Q92597 P14618

Q9GZQ8 P14618

Q9H0R8 P14618

Q9H492 P14618

Q9UBE8 P14618

Q09472 P14618

Q16665 P14618

O15264 P14625

O95166 P14625

P00533 P14625

P01100 P14625

P04179 P14625

P15336 P14625

P16104 P14625

P19388 P14625

P22736 P14625

P24386 P14625

P29692 P14625

P60520 P14625

P68400 P14625

Q13573 P14625

Q99459 P14625

Q9GZZ9 P14625

Q9H0R8 P14625

Q9NTG7 P14625

Q9NZJ5 P14625

P06493 P14635

P24941 P14635

Q00535 P14635

Q13573 P14635

Q96EB6 P14635

Q99459 P14635

Q99640 P14635

P03372 P14649

P62068 P14649

P40424 P14653

Q9UER7 P14672

O14744 P14678

O14879 P14678

O43719 P14678

O75643 P14678

O94906 P14678

O95166 P14678

O95400 P14678

P02751 P14678

P09913 P14678

P09914 P14678

P38432 P14678

P38919 P14678

P42771 P14678

P50053 P14678

P54105 P14678

P57678 P14678

P60520 P14678

P61326 P14678

P78362 P14678

Q13523 P14678

Q14204 P14678

Q15020 P14678

Q15029 P14678

Q16637 P14678

Q8HWS3 P14678

Q8TEQ6 P14678

Q9GZQ8 P14678

Q9H0R8 P14678

Q9UKR5 P14678

Q9UPE1 P14678

Q9Y241 P14678

Q9Y3F4 P14678

O75940 P14678

P10147 P14735

Q9BYX4 P14735

Q9Y6E7 P14735

P07359 P14770

P46108 P14770

Q92624 P14770

Q86XR7 P14778

Q9NPH3 P14778

Q9Y4K3 P14778

P08648 P14780

P13611 P14780

P14780 P14780

P15502 P14780

Q16819 P14780

Q16820 P14780

Q8IX30 P14780

P00403 P14854

Q9UJS0 P14854

P08047 P14859

O14879 P14866

O95166 P14866

P09913 P14866

P11387 P14866

P17096 P14866

P19320 P14866

P22607 P14866

P60520 P14866

P67809 P14866

P68400 P14866

Q00005 P14866

Q15366 P14866

Q9GZQ8 P14866

Q9H0R8 P14866

Q9H3D4 P14866

Q9H492 P14866

Q9H4D4 P14866

Q9UL18 P14866

P07900 P14868

P19320 P14868

P27986 P14868

P62993 P14868

Q13155 P14868

Q9H492 P14868

Q09472 P14920

O00470 P14921

O43711 P14921

O94813 P14921

O95551 P14921

P05412 P14921

P07814 P14921

P09429 P14921

P10275 P14921

P17535 P14921

P17542 P14921

P19338 P14921

P23497 P14921

P28069 P14921

P28482 P14921

P31314 P14921

P49588 P14921

P51531 P14921

P53804 P14921

P54727 P14921

P84996 P14921

Q01105 P14921

Q06481 P14921

Q14152 P14921

Q4LE39 P14921

Q5VUB5 P14921

Q66PJ3 P14921

Q6IA86 P14921

Q8IZU2 P14921

Q8N6N3 P14921

Q969E4 P14921

Q96KR1 P14921

Q96NC0 P14921

Q96RV3 P14921

Q99471 P14921

Q9BQA9 P14921

Q9UER7 P14921

Q9Y4W2 P14921

O94782 P14923

P03372 P14923

P15924 P14923

P16284 P14923

P25054 P14923

P33993 P14923

P38398 P14923

P60520 P14923

Q00005 P14923

Q13164 P14923

Q14934 P14923

Q96F31 P14923

Q96HA8 P14923

Q96JB5 P14923

Q96Q40 P14923

Q9GZQ8 P14923

Q9H0R8 P14923

Q9H4K1 P14923

Q9NQB0 P14923

Q9NSA3 P14923

P08574 P14927

P31930 P14927

P40189 P15018

P42702 P15018

Q15131 P15036

Q15672 P15036

O43482 P15056

P04049 P15056

P15056 P15056

P31946 P15056

P31947 P15056

P54274 P15056

P63104 P15056

Q02750 P15056

Q04917 P15056

Q13177 P15056

Q15349 P15056

Q3V6T2 P15056

Q3ZCQ8 P15056

Q6VAB6 P15056

Q8IVT5 P15056

P49407 P15085

O76070 P15090

O96006 P15090

P08670 P15090

P17020 P15090

P41743 P15090

Q92882 P15090

Q93063 P15090

Q9Y4E8 P15090

O75925 P15104

O75928 P15104

P03915 P15104

P15104 P15104

Q6ZVK8 P15104

Q7Z6E9 P15104

Q9H8W4 P15104

Q9UKL3 P15104

Q9Y6X2 P15104

O75346 P15121

P15924 P15121

P23508 P15121

P23759 P15121

Q03001 P15121

Q15797 P15121

Q96PZ7 P15121

Q495A1 P15151

Q9H4A3 P15151

Q9NZ20 P15151

P04839 P15153

P05060 P15153

P15153 P15153

P19320 P15153

P42768 P15153

P52566 P15153

P98170 P15153

Q969S8 P15153

P60953 P15169

Q6UXB4 P15169

P11940 P15170

P62495 P15170

Q13573 P15170

Q14457 P15170

Q92731 P15170

Q92900 P15170

O00505 P15172

O15105 P15172

P11831 P15172

Q13485 P15172

Q8IXJ6 P15172

Q9BSF0 P15172

Q9UL46 P15172

P11831 P15173

Q13634 P15173

Q5I0X7 P15173

Q9H0R8 P15259

Q9Y371 P15259

Q9Y4E8 P15259

P01579 P15260

P19838 P15260

P42224 P15260

P41208 P15289

Q15645 P15289

Q6UXB4 P15289

P01871 P15309

P04626 P15309

P15309 P15309

O94782 P15311

P07332 P15311

P15311 P15311

P16070 P15311

P23508 P15311

P35241 P15311

P43146 P15311

P52565 P15311

P53779 P15311

Q00987 P15311

Q01970 P15311

Q6ZVD8 P15311

Q8IZE3 P15311

Q9BXI6 P15311

Q9H0R8 P15311

Q9UFF9 P15311

Q9Y6Q1 P15311

O43463 P15313

Q13618 P15328

Q9Y616 P15328

A4PIV8 P15336

A4PIW0 P15336

O75530 P15336

P01100 P15336

P05412 P15336

P15336 P15336

P17275 P15336

P19367 P15336

P36542 P15336

P40692 P15336

Q04724 P15336

Q15022 P15336

Q15532 P15336

Q15759 P15336

Q15910 P15336

Q8IU81 P15336

Q8WYK2 P15336

Q99966 P15336

Q99986 P15336

P0CG47 P15374

Q7L5N1 P15374

Q92905 P15374

Q9H078 P15374

Q9P215 P15374

Q9Y2K6 P15374

Q9Y328 P15374

P62158 P15382

P01871 P15391

P21926 P15391

P27986 P15391

Q06187 P15391

Q92569 P15391

Q99856 P15391

O95751 P15407

P01106 P15407

P54253 P15407

Q9HBI0 P15407

Q9P1Z2 P15407

P01106 P15408

P00519 P15498

P06239 P15498

P22681 P15498

P27986 P15498

P61586 P15498

P62993 P15498

P63000 P15498

P78314 P15498

Q07666 P15498

Q13094 P15498

Q8IZP0 P15498

Q8N720 P15498

Q9Y2R2 P15498

O95967 P15502

P01857 P15502

P17540 P15502

Q9H213 P15502

Q9UBX5 P15502

P39900 P15502

P04141 P15509

P33763 P15514

Q9NRR5 P15514

Q8TAX7 P15515

Q8TAX7 P15516

P78504 P15529

Q9UEW8 P15529

O00746 P15531

O15160 P15531

O94993 P15531

P10911 P15531

P15531 P15531

P21980 P15531

P22392 P15531

P30281 P15531

P51116 P15531

P54274 P15531

Q13232 P15531

Q86TP1 P15531

Q96GM5 P15531

Q96HA8 P15531

Q9GZT8 P15531

Q9H0R8 P15531

Q9H492 P15531

Q9H8Y8 P15531

Q9UMW8 P15531

Q9Y3F4 P15531

P19320 P15559

Q9UK53 P15559

P00519 P15586

P16333 P15586

Q7Z7J5 P15622

Q96BR9 P15622

Q9BQ66 P15622

Q9C0F1 P15622

O14786 P15692

P15692 P15692

P17948 P15692

P35968 P15692

Q96QK1 P15692

Q9BZD6 P15692

P01857 P15814

P08473 P15814

P21926 P15814

O00560 P15822

O14503 P15822

P05062 P15822

P07306 P15822

P08107 P15822

P10644 P15822

P32754 P15822

P38398 P15822

Q02575 P15822

Q12983 P15822

Q13568 P15822

Q16891 P15822

Q969X6 P15822

Q96BI3 P15822

Q9UHV2 P15822

Q9UKB1 P15822

Q9Y297 P15822

P01106 P15848

P03372 P15848

O95166 P15880

O95793 P15880

P01106 P15880

P03372 P15880

P04183 P15880

P04626 P15880

P19320 P15880

P20073 P15880

P35711 P15880

P40692 P15880

P49407 P15880

P60520 P15880

Q14451 P15880

Q15843 P15880

Q16543 P15880

Q9H0R8 P15880

Q9H492 P15880

Q9H4L4 P15880

Q9UKV8 P15880

Q9UL18 P15880

P61247 P15882

Q12789 P15882

Q13387 P15882

O00512 P15884

P11388 P15884

P15884 P15884

P17542 P15884

P35222 P15884

P41134 P15884

P50553 P15884

Q96AC1 P15884

Q9UJU2 P15884

Q9UPN9 P15884

P01106 P15918

P19174 P15918

P52294 P15918

P17542 P15923

P50553 P15923

P61927 P15923

Q9Y230 P15923

Q9Y265 P15923

P01009 P15923

P15172 P15923

P15923 P15923

Q02363 P15923

Q02535 P15923

O14879 P15924

O95166 P15924

P01106 P15924

P16284 P15924

P60520 P15924

Q02156 P15924

Q13573 P15924

Q15051 P15924

Q99459 P15924

Q99569 P15924

Q9GZQ8 P15924

Q9H0R8 P15924

Q9H492 P15924

Q00005 P15924

Q9H7D0 P15924

O75419 P15927

P02751 P15927

P23025 P15927

P27694 P15927

P35244 P15927

P51587 P15927

P54132 P15927

Q8TDY2 P15927

Q9BQS8 P15927

Q9H9A7 P15927

Q9UQ84 P15927

P00519 P15941

P00533 P15941

P05412 P15941

P12931 P15941

P15941 P15941

P62993 P15941

P00403 P15954

P08107 P15976

P0CG48 P15976

P42574 P15976

O00264 P16035

O14818 P16035

O76070 P16035

O95757 P16035

O95996 P16035

P08253 P16035

P20336 P16035

P22061 P16035

P22234 P16035

P30084 P16035

P43487 P16035

P47914 P16035

P55786 P16035

P60900 P16035

P61289 P16035

Q14697 P16035

Q9Y266 P16035

Q9Y617 P16035

P01106 P16066

P15336 P16070

Q9UPY5 P16070

P00533 P16083

Q9H8Y8 P16083

O60934 P16104

P03372 P16104

P26358 P16104

P32121 P16104

P38398 P16104

P49407 P16104

P63104 P16104

Q13315 P16104

Q14676 P16104

Q15843 P16104

Q9ULG1 P16104

Q9UQ84 P16104

Q14242 P16109

P16118 P16118

P27361 P16118

P35557 P16118

P00533 P16144

P15336 P16144

P18054 P16144

Q12772 P16144

Q15149 P16144

Q9BY67 P16144

P84022 P16144

Q15796 P16144

O15264 P16152

P03372 P16152

P62993 P16152

Q13216 P16152

Q9BSB4 P16152

Q9BW61 P16152

Q9GZZ9 P16152

Q9Y478 P16152

Q5VST9 P161577

P30450 P16188

P21860 P16189

O00716 P16219

O00141 P16220

O00470 P16220

P08670 P16220

Q12772 P16220

Q12873 P16220

Q6SA08 P16220

Q6UUV9 P16220

Q6ZNA4 P16220

Q7Z3K3 P16220

Q8IYT8 P16220

Q92793 P16220

Q9UKR5 P16220

Q9UKY1 P16220

P62258 P16233

P37173 P16234

P46108 P16234

P01023 P16278

P01100 P16278

P04183 P16278

P16104 P16278

P67870 P16278

Q99519 P16278

O14796 P16284

O75400 P16284

P08670 P16284

P12931 P16284

P12956 P16284

P14923 P16284

P29350 P16284

P42684 P16284

Q02156 P16284

Q06124 P16284

O95180 P16298

P14316 P16298

P60520 P16298

P62993 P16298

O00294 P16333

O00750 P16333

O14513 P16333

O15056 P16333

O15117 P16333

O43150 P16333

O43426 P16333

O43516 P16333

O43708 P16333

O43918 P16333

O60287 P16333

O60500 P16333

O75167 P16333

O75326 P16333

O75369 P16333

O75534 P16333

O94856 P16333

O94885 P16333

P00519 P16333

P00533 P16333

P01178 P16333

P06241 P16333

P07766 P16333

P08047 P16333

P09619 P16333

P10301 P16333

P10412 P16333

P11678 P16333

P15918 P16333

P20231 P16333

P20936 P16333

P21817 P16333

P21860 P16333

P22681 P16333

P23759 P16333

P23760 P16333

P26951 P16333

P26992 P16333

P29074 P16333

P30260 P16333

P35968 P16333

P41970 P16333

P42167 P16333

P42768 P16333

P43699 P16333

P46092 P16333

P51816 P16333

P54762 P16333

P62987 P16333

P98082 P16333

Q01484 P16333

Q05193 P16333

Q07889 P16333

Q07890 P16333

Q07912 P16333

Q13094 P16333

Q13111 P16333

Q13177 P16333

Q14118 P16333

Q14185 P16333

Q14315 P16333

Q15027 P16333

Q15109 P16333

Q15661 P16333

Q6PJG9 P16333

Q7Z408 P16333

Q86UR5 P16333

Q8IWN7 P16333

Q8IXK0 P16333

Q8WX92 P16333

Q96NS5 P16333

Q99259 P16333

Q9BWF2 P16333

Q9BYB0 P16333

Q9H222 P16333

Q9H9L3 P16333

Q9H9Y6 P16333

Q9HCM9 P16333

Q9HCQ7 P16333

Q9HCU4 P16333

Q9NQ76 P16333

Q9NRR4 P16333

Q9NYQ7 P16333

Q9UHI7 P16333

Q9UL42 P16333

Q9ULH1 P16333

Q9ULW0 P16333

Q9UM47 P16333

Q9UNH6 P16333

Q9UPX8 P16333

Q9UQ26 P16333

Q9Y2D5 P16333

Q9Y2H0 P16333

P06241 P16383

P54253 P16383

Q96DB2 P16383

O76075 P16401

P02751 P16401

P19320 P16401

Q96HA7 P16401

O76075 P16402

O95758 P16402

P51858 P16402

P63104 P16402

Q9H3D4 P16402

O15264 P16403

O95166 P16403

O95503 P16403

P11387 P16403

P19525 P16403

P32121 P16403

P38919 P16403

P49407 P16403

P51858 P16403

P60520 P16403

P61326 P16403

P63104 P16403

Q04206 P16403

Q92731 P16403

Q96HA7 P16403

Q9GZQ8 P16403

Q9H0R8 P16403

Q9Y6K5 P16403

P27986 P16410

P33681 P16410

Q13153 P16415

P13569 P16422

O00264 P16435

P12956 P16435

P42224 P16435

Q96EK5 P16444

P17020 P16452

P58062 P16452

Q16539 P16452

P01236 P16471

P16519 P16519

P04626 P16520

Q92731 P16520

Q99459 P16520

O00257 P16591

O15117 P16591

P04626 P16591

P21860 P16591

P46459 P16591

Q8IZP0 P16591

O00716 P16615

P00533 P16615

P01106 P16615

P01375 P16615

P04626 P16615

P13569 P16615

P15336 P16615

P41143 P16615

Q13418 P16615

P34903 P16662

Q12884 P16860

P27694 P16870

Q12789 P16870

Q12873 P16870

Q13131 P16870

Q14181 P16870

Q5UIP0 P16870

Q9BVJ6 P16870

Q9HCK4 P16870

Q9Y2S7 P16870

Q9Y3C7 P16870

P13232 P16871

P04626 P16885

P19235 P16885

P27986 P16885

P78314 P16885

Q8WV28 P16885

Q96KM6 P16885

Q9H204 P16885

P19320 P16949

O14879 P16989

O95166 P16989

P03372 P16989

P09913 P16989

P28482 P16989

P32121 P16989

P38919 P16989

P49841 P16989

P60520 P16989

P61326 P16989

Q13573 P16989

Q96DB2 P16989

Q9GZQ8 P16989

Q9HC52 P16989

Q9UPE1 P16989

O95425 P17020

P02795 P17020

P03905 P17020

P06733 P17020

P08123 P17020

P11142 P17020

P17097 P17020

P25685 P17020

P31997 P17020

P49915 P17020

P56385 P17020

P59665 P17020

P62987 P17020

P68104 P17020

P68133 P17020

Q13185 P17020

Q2QGD7 P17020

Q86WV6 P17020

Q9UKU0 P17020

Q9GZM8 P17021

P54257 P17024

Q9BXL5 P17024

P04637 P17028

P09429 P17028

P10909 P17028

P13994 P17028

P26641 P17028

P46781 P17028

P51693 P17028

P51784 P17028

P54257 P17028

P57086 P17028

P61927 P17028

P68104 P17028

Q05516 P17028

Q13129 P17028

Q13432 P17028

Q14194 P17028

Q15032 P17028

Q15047 P17028

Q5RL73 P17028

Q5UIP0 P17028

Q7L5N1 P17028

Q96AF5 P17028

Q99442 P17028

Q9BVJ6 P17028

Q9NX65 P17028

Q9P2H0 P17028

Q9UJV3 P17028

Q9UKR5 P17028

Q9BXL5 P17030

P17036 P17036

Q02535 P17036

Q9UDV6 P17036

P49760 P17039

P57086 P17040

Q8IYT8 P17041

O00716 P17066

O95166 P17066

P32121 P17066

P60520 P17066

Q15051 P17066

Q3MIX3 P17066

Q8TDR0 P17066

Q92731 P17066

Q92905 P17066

Q99459 P17066

Q9GZQ8 P17066

Q9H0R8 P17066

Q9H492 P17066

Q9UDY4 P17066

Q9UNE7 P17066

Q13153 P17081

P02751 P17096

P08047 P17096

P08238 P17096

P17676 P17096

P19320 P17096

P42858 P17096

Q01844 P17096

P06493 P17096

Q96LA8 P17096

P68400 P17096

O75528 P17097

P45973 P17098

Q13185 P17098

O43463 P17152

P10809 P17152

Q12983 P17152

Q96BA8 P17152

Q96GD3 P17152

P00533 P17174

Q7Z7G0 P17174

P05000 P17181

P52630 P17181

Q09472 P17181

P05198 P17252

P08238 P17252

P0CG48 P17252

P15056 P17252

P28070 P17252

P29597 P17252

P35222 P17252

P63000 P17252

Q07021 P17252

Q15311 P17252

Q6R327 P17252

Q8WV44 P17252

O14776 P17275

P01100 P17275

P0CG48 P17275

P11137 P17275

P15407 P17275

P17275 P17275

P21673 P17275

P23560 P17275

P31268 P17275

Q13485 P17275

Q13569 P17275

Q16520 P17275

Q7Z3I0 P17275

Q8N1L9 P17275

Q8WYK2 P17275

Q9Y2I6 P17275

O00401 P17301

P02768 P17301

P05413 P17301

P05556 P17301

P15336 P17301

P17301 P17301

Q99828 P17301

Q9H0F6 P17301

P41240 P17302

Q02487 P17302

Q07157 P17302

A0JLT2 P17480

P01106 P17480

P03372 P17480

P05060 P17480

P49715 P17480

Q01105 P17480

Q9BQG0 P17480

Q9BXL5 P17480

O95758 P17482

P21673 P17482

Q8IY92 P17482

Q93062 P17482

Q99750 P17482

Q9BQ66 P17482

Q9BRK4 P17482

P19012 P17509

P21673 P17509

P01100 P17535

P61158 P17535

Q00987 P17535

Q8NHY2 P17535

Q8WYK2 P17535

Q13432 P17540

Q5UIP0 P17540

Q9BVK6 P17540

Q9NRN5 P17540

P15923 P17542

P17482 P17542

P50750 P17542

Q01196 P17542

Q9UPN9 P17542

Q9Y295 P17542

Q9Y5B9 P17542

O95983 P17544

P20936 P17544

P41182 P17544

Q8WYK2 P17544

Q9NX40 P17544

O43678 P17568

P20794 P17568

Q8NB16 P17568

P00533 P17600

P08670 P17600

P17600 P17600

P37840 P17600

Q92993 P17600

P22694 P17612

P49841 P17612

P83916 P17612

Q08499 P17612

Q13185 P17612

Q70CQ1 P17612

Q9HBX9 P17612

Q9Y297 P17612

Q9Y463 P17612

P30419 P17655

O95758 P17661

P15336 P17661

P17661 P17661

P40692 P17661

P63104 P17661

Q13496 P17661

Q8N3C7 P17661

Q969Q1 P17661

Q9HAU4 P17661

P03372 P17676

P04637 P17676

P17096 P17676

Q13485 P17676

Q05655 P17677

Q8NHL6 P17693

O00401 P17706

P10912 P17706

P31947 P17706

P60953 P17706

Q9Y5K5 P17706

P49755 P17706

Q14789 P17706

Q9BVK6 P17706

P01106 P17752

O95166 P17812

O95758 P17812

P01106 P17812

P32121 P17812

P60520 P17812

P63104 P17812

Q14164 P17812

Q8NCN4 P17812

Q9GZQ8 P17812

Q9H0R8 P17812

Q9H492 P17812

P01137 P17813

P08648 P17813

P50281 P17813

B2Y833 P17844

O43251 P17844

O43918 P17844

O94916 P17844

O94921 P17844

O95166 P17844

O95229 P17844

O95983 P17844

P03372 P17844

P04637 P17844

P08047 P17844

P11387 P17844

P13805 P17844

P19320 P17844

P22087 P17844

P24928 P17844

P31749 P17844

P31942 P17844

P32121 P17844

P42336 P17844

P49760 P17844

P50616 P17844

P51858 P17844

P55795 P17844

P60520 P17844

P60709 P17844

P62993 P17844

P67809 P17844

Q07666 P17844

Q08211 P17844

Q09472 P17844

Q12873 P17844

Q13573 P17844

Q15428 P17844

Q15596 P17844

Q16543 P17844

Q5U5Q3 P17844

Q7L0Y3 P17844

Q92597 P17844

Q92841 P17844

Q99459 P17844

Q9GZQ8 P17844

Q9H0R8 P17844

Q9H492 P17844

Q9NRR4 P17844

Q9UKV8 P17844

Q9UL18 P17844

Q9Y2W2 P17844

Q9Y6Q9 P17844

P02751 P17858

P08237 P17858

P17858 P17858

P19320 P17858

P19438 P17858

Q13573 P17858

Q14164 P17858

Q7L5N1 P17858

Q99836 P17858

Q9BQ66 P17858

P17861 P17861

P60520 P17900

P13569 P17931

Q29983 P17931

Q12789 P17936

Q14181 P17936

P31260 P17947

P78527 P17947

Q15156 P17947

Q99684 P17947

P01130 P17948

P15692 P17948

P19174 P17948

P22681 P17948

P35222 P17948

P46108 P17948

P46109 P17948

P49763 P17948

Q05397 P17948

Q06124 P17948

Q12913 P17948

Q96SB4 P17948

O00233 P17980

O75832 P17980

P01106 P17980

P20807 P17980

P24522 P17980

P35998 P17980

P51665 P17980

P54578 P17980

P62195 P17980

P62333 P17980

Q13573 P17980

Q16401 P17980

Q99459 P17980

Q9H0R8 P17980

Q9NWV8 P17980

Q9Y5K5 P17980

O00506 P17987

O15379 P17987

O75381 P17987

O95166 P17987

P01106 P17987

P04637 P17987

P08238 P17987

P13569 P17987

P15336 P17987

P19320 P17987

P60510 P17987

P60520 P17987

P62714 P17987

P67775 P17987

P78318 P17987

P84022 P17987

Q00005 P17987

Q00535 P17987

Q04206 P17987

Q13033 P17987

Q13418 P17987

Q13573 P17987

Q15257 P17987

Q66LE6 P17987

Q8IWZ6 P17987

Q8TAM1 P17987

Q8WZ74 P17987

Q99459 P17987

Q9GZQ8 P17987

Q9GZX7 P17987

Q9H0R8 P17987

Q9H492 P17987

Q9H7D0 P17987

Q9NPJ1 P17987

Q9NRL3 P17987

Q9UL18 P17987

Q9Y228 P17987

Q9Y2T4 P17987

Q9Y3A3 P17987

Q9Y6E0 P17987

O43561 P18031

O60674 P18031

P00519 P18031

P00533 P18031

P00966 P18031

P02730 P18031

P03372 P18031

P04629 P18031

P05129 P18031

P06213 P18031

P07384 P18031

P08069 P18031

P08922 P18031

P09619 P18031

P10912 P18031

P11274 P18031

P12814 P18031

P12931 P18031

P15336 P18031

P17655 P18031

P18031 P18031

P19235 P18031

P29376 P18031

P40763 P18031

P42229 P18031

P49841 P18031

P56945 P18031

P62993 P18031

Q03135 P18031

Q14247 P18031

Q16288 P18031

Q9H1D0 P18031

P02545 P18054

P13647 P18054

P14555 P18054

Q96RU8 P18054

Q9C086 P18065

P50613 P18074

Q13253 P18075

Q5JR59 P18075

O95758 P18077

P32121 P18077

P51858 P18077

P60520 P18077

P62993 P18077

Q15843 P18077

Q99963 P18077

Q9UKV8 P18077

Q9UL18 P18077

P02768 P18084

P04406 P18084

P30101 P18084

P37268 P18084

Q01844 P18084

Q12929 P18084

Q14324 P18084

Q63HR2 P18084

Q8WZ42 P18084

Q9BY76 P18084

P01106 P18085

P15336 P18085

Q13573 P18085

Q9HCK5 P18085

Q9NWV8 P18085

O00410 P18124

P02751 P18124

P03372 P18124

P08238 P18124

P19320 P18124

P22736 P18124

P32121 P18124

P38919 P18124

P42345 P18124

P60520 P18124

P60709 P18124

P61254 P18124

P61326 P18124

P62826 P18124

P62993 P18124

P63244 P18124

Q15843 P18124

Q8NB14 P18124

Q8TDR0 P18124

Q92731 P18124

Q9UKV8 P18124

Q9UL18 P18124

P00451 P18135

Q9H0R8 P18135

P04637 P18146

P08047 P18146

P11473 P18146

P15884 P18146

P35222 P18146

Q09472 P18146

Q8N726 P18146

Q9UJU2 P18146

O15144 P18206

P02751 P18206

P19320 P18206

P49023 P18206

Q0VDD7 P18206

Q9NYB9 P18206

Q9Y244 P18206

O15047 P18428

P04003 P18428

P84022 P18428

Q10588 P18428

Q13162 P18428

Q13595 P18428

Q53H96 P18428

P12931 P18433

P18433 P18433

P60520 P18433

P62993 P18433

Q9P2Y5 P18433

Q9HD90 P18505

P14778 P18510

P06756 P18564

P31946 P18583

P38919 P18583

P49760 P18583

P61326 P18583

P78362 P18583

Q8N3C7 P18583

Q8TAD8 P18583

Q96DB2 P18583

Q96SB4 P18583

Q9UPE1 P18583

O75376 P18615

P08238 P18615

Q15836 P18615

Q8IXH7 P18615

Q8TAF3 P18615

Q8WX92 P18615

Q9H3P2 P18615

O95989 P18621

P00533 P18621

P03372 P18621

P19320 P18621

P38919 P18621

P51858 P18621

P56545 P18621

P61254 P18621

P61326 P18621

Q00005 P18621

Q92731 P18621

Q9UKV8 P18621

Q9UL18 P18621

O15264 P18669

P05067 P18669

P12004 P18669

P12956 P18669

P15336 P18669

Q15051 P18669

P02751 P18754

P17096 P18754

P19320 P18754

P27694 P18754

P62826 P18754

Q99986 P18754

Q9H6Z4 P18754

Q9UKV0 P18754

O60869 P18846

P03372 P18846

O43463 P18847

P04637 P18847

P18847 P18847

Q02535 P18847

Q04206 P18847

Q14192 P18847

Q5UIP0 P18847

Q96ID5 P18847

Q9UDV6 P18847

O14733 P18848

P17275 P18848

P18848 P18848

P19387 P18848

P21980 P18848

P25963 P18848

P62753 P18848

Q00987 P18848

Q96BI3 P18848

Q96RU7 P18848

Q99871 P18848

Q9NS37 P18848

P16220 P18850

P18850 P18850

P20711 P18850

Q68CJ9 P18850

P41220 P18858

P24539 P18859

P51812 P18859

Q16659 P18859

O96017 P18887

P06746 P18887

P09874 P18887

P16104 P18887

P22674 P18887

P49916 P18887

Q7Z2E3 P18887

Q8IW19 P18887

Q96T60 P18887

Q9UGP5 P18887

Q9UNA4 P18887

O14639 P19012

O75604 P19012

O95751 P19012

O95990 P19012

P05783 P19012

P08727 P19012

P25786 P19012

P35900 P19012

P41219 P19012

P62195 P19012

Q14533 P19012

Q14966 P19012

Q15013 P19012

Q15154 P19012

Q53FD0 P19012

Q5T5P2 P19012

Q6PKC3 P19012

Q6ZU52 P19012

Q6ZVK8 P19012

Q7L590 P19012

Q7LC44 P19012

Q7Z3B3 P19012

Q8IYI6 P19012

Q8N6Y0 P19012

Q8N715 P19012

Q8TAB5 P19012

Q8TBB1 P19012

Q8TCX5 P19012

Q8WW24 P19012

Q96ES7 P19012

Q96GM5 P19012

Q96HB5 P19012

Q96IX9 P19012

Q99816 P19012

Q9BT92 P19012

Q9BTA9 P19012

Q9BVI4 P19012

Q9BVR6 P19012

Q9BY27 P19012

Q9GZT8 P19012

Q9H4K1 P19012

Q9NTK1 P19012

Q9NYB9 P19012

Q9P1Z2 P19012

Q9P2A4 P19012

Q9P2K3 P19012

Q9Y2J4 P19012

O94782 P19013

P09936 P19013

Q13501 P19013

Q5VYV7 P19013

Q5W0Q7 P19013

Q8IY92 P19013

Q92636 P19013

Q9BQ83 P19013

Q9H0R8 P19013

Q9UHP3 P19013

Q9Y4P1 P19013

O75618 P19021

P10644 P19021

P10644 P19022

P23470 P19022

P35222 P19022

Q05209 P19022

Q12913 P19022

Q15256 P19022

O14578 P19105

O95758 P19105

P42771 P19105

P60709 P19105

P62993 P19105

P63104 P19105

Q15746 P19105

Q5TKA1 P19105

Q96GY3 P19105

Q9H3D4 P19105

Q9H5I1 P19105

A2RU30 P19174

O15085 P19174

O15117 P19174

O43150 P19174

O43493 P19174

O43561 P19174

O60674 P19174

O75167 P19174

O95487 P19174

O95886 P19174

P00519 P19174

P00533 P19174

P01008 P19174

P04626 P19174

P04629 P19174

P06213 P19174

P07949 P19174

P09327 P19174

P09619 P19174

P10301 P19174

P10912 P19174

P11362 P19174

P16234 P19174

P19174 P19174

P20273 P19174

P20810 P19174

P20936 P19174

P21333 P19174

P21860 P19174

P27816 P19174

P30530 P19174

P31749 P19174

P31994 P19174

P35968 P19174

P42684 P19174

P43405 P19174

P62993 P19174

P63000 P19174

P78314 P19174

Q05397 P19174

Q07666 P19174

Q07889 P19174

Q13094 P19174

Q13393 P19174

Q14108 P19174

Q15036 P19174

Q7L0Q8 P19174

Q7Z408 P19174

Q8IZP0 P19174

Q8TB24 P19174

Q8WX92 P19174

Q92832 P19174

Q92918 P19174

Q92988 P19174

Q96EV8 P19174

Q96T58 P19174

Q9BWF2 P19174

Q9BYB0 P19174

Q9H1R2 P19174

Q9H2P0 P19174

Q9H5I1 P19174

Q9H9L3 P19174

Q9NQ76 P19174

Q9UHI7 P19174

Q9ULH1 P19174

Q9UPX8 P19174

O14508 P19235

P01588 P19235

P08575 P19235

P16885 P19235

P18031 P19235

P19235 P19235

P23470 P19235

P29350 P19235

Q12913 P19235

Q8WWM7 P19235

Q92835 P19235

Q9NSE2 P19235

P63316 P19237

Q15834 P19237

P31689 P19256

P19320 P19320

O00716 P19338

O14879 P19338

O15264 P19338

O75116 P19338

O95166 P19338

O95503 P19338

O95793 P19338

P01106 P19338

P02751 P19338

P03372 P19338

P04637 P19338

P08631 P19338

P09914 P19338

P10644 P19338

P11387 P19338

P17096 P19338

P19320 P19338

P19338 P19338

P23246 P19338

P32121 P19338

P35579 P19338

P39060 P19338

P46781 P19338

P49407 P19338

P51858 P19338

P60520 P19338

P60709 P19338

P60763 P19338

P61586 P19338

P62993 P19338

P63104 P19338

Q00987 P19338

Q01130 P19338

Q06710 P19338

Q13464 P19338

Q13573 P19338

Q14790 P19338

Q5S007 P19338

Q7Z2E3 P19338

Q8N0X7 P19338

Q92597 P19338

Q99608 P19338

Q99697 P19338

Q9GZQ8 P19338

Q9H0R8 P19338

Q9H492 P19338

Q9HC52 P19338

Q9UBU9 P19338

Q9UL18 P19338

Q9UPE1 P19338

Q9UQ80 P19338

O75323 P19367

P01106 P19367

P15336 P19367

P21796 P19367

P61244 P19367

Q14197 P19367

Q96GD3 P19367

Q9BSB4 P19367

A0JLT2 P19387

O00193 P19387

O76061 P19387

O95402 P19387

P02751 P19387

P18848 P19387

P25963 P19387

P68104 P19387

Q5UIP0 P19387

Q8TAQ2 P19387

Q96B02 P19387

Q9BTT4 P19387

Q9NWA0 P19387

Q9NX70 P19387

Q9UKR5 P19387

A0JLT2 P19388

O00445 P19388

O43156 P19388

O94763 P19388

O95402 P19388

O95602 P19388

P01100 P19388

P05060 P19388

P07900 P19388

P12830 P19388

P32121 P19388

P49407 P19388

P51784 P19388

P60520 P19388

Q13573 P19388

Q96Q15 P19388

Q9BTT4 P19388

Q9NWA0 P19388

Q9NWM8 P19388

Q9NX70 P19388

Q9Y265 P19388

O43678 P19404

O75306 P19404

O75489 P19404

P02751 P19404

Q14194 P19404

Q16795 P19404

Q92993 P19404

Q9HB58 P19404

P27361 P19419

P28482 P19419

P63279 P19419

Q00534 P19419

P19474 P19429

Q969Q1 P19429

O00165 P19438

O14735 P19438

O15397 P19438

O95816 P19438

P01375 P19438

P04196 P19438

P04350 P19438

P04908 P19438

P05023 P19438

P05141 P19438

P07437 P19438

P0CG47 P19438

P11021 P19438

P11142 P19438

P12236 P19438

P16615 P19438

P17066 P19438

P17405 P19438

P18850 P19438

P19105 P19438

P19438 P19438

P23396 P19438

P25705 P19438

P27708 P19438

P27824 P19438

P28799 P19438

P29350 P19438

P30048 P19438

P31689 P19438

P33778 P19438

P34931 P19438

P35579 P19438

P35580 P19438

P39656 P19438

P40939 P19438

P42677 P19438

P48729 P19438

P51571 P19438

P53675 P19438

P54136 P19438

P55209 P19438

P55210 P19438

P58107 P19438

P60660 P19438

P61619 P19438

P62158 P19438

P62249 P19438

P62805 P19438

P62829 P19438

P62979 P19438

P62987 P19438

P63261 P19438

P78527 P19438

Q00325 P19438

Q00610 P19438

Q07021 P19438

Q13546 P19438

Q13748 P19438

Q14257 P19438

Q15628 P19438

Q15758 P19438

Q16695 P19438

Q3ZCQ8 P19438

Q7Z3U7 P19438

Q92616 P19438

Q92636 P19438

Q92835 P19438

Q96AG4 P19438

Q96DZ1 P19438

Q96EY1 P19438

Q9BUF5 P19438

Q9BVA1 P19438

Q9BXW9 P19438

Q9NVI1 P19438

Q9NVI7 P19438

Q9NXW2 P19438

Q9UBV2 P19438

Q9UM54 P19438

Q9UMX0 P19438

Q9UQC1 P19438

Q9Y265 P19438

Q9Y4W6 P19438

Q9Y6D5 P19438

P43490 P19440

P01106 P19447

P10275 P19447

P50613 P19447

P51946 P19447

P54727 P19447

P62195 P19447

Q01831 P19447

Q15796 P19447

O94966 P19474

O95758 P19474

P01106 P19474

P14373 P19474

P19474 P19474

P22681 P19474

P29692 P19474

P60520 P19474

P61981 P19474

Q01973 P19474

Q13107 P19474

Q15051 P19474

Q6ZNA4 P19474

Q92793 P19474

Q99459 P19474

Q9BYM8 P19474

Q9GZQ8 P19474

Q9GZT8 P19474

Q9H0R8 P19474

Q9H492 P19474

Q9H9G7 P19474

Q9HCK5 P19474

B2Y833 P19474

P05198 P19525

P06493 P19525

P06748 P19525

P08238 P19525

P60520 P19525

P60953 P19525

Q08211 P19525

Q15633 P19525

Q16543 P19525

Q6P2E9 P19525

Q7L2E3 P19525

Q8IY81 P19525

Q96C10 P19525

Q9NUL3 P19525

O15067 P19532

O75694 P19532

O95347 P19532

P07814 P19532

P15121 P19532

P21266 P19532

P25786 P19532

P25788 P19532

P26640 P19532

P35244 P19532

P42704 P19532

P49321 P19532

P52788 P19532

P53396 P19532

P55072 P19532

P55209 P19532

P56192 P19532

P60900 P19532

P62714 P19532

P63173 P19532

Q00610 P19532

Q01105 P19532

Q08211 P19532

Q13263 P19532

Q13347 P19532

Q13617 P19532

Q14152 P19532

Q14697 P19532

Q15843 P19532

Q16658 P19532

Q8WUM4 P19532

Q99460 P19532

Q99623 P19532

Q9HB71 P19532

O75607 P19544

O95201 P19544

Q00987 P19544

Q7L7X3 P19544

Q92997 P19544

P19320 P19623

P19623 P19623

Q09019 P19623

Q9BW61 P19623

O43745 P19634

O95819 P19634

P31946 P19634

P63104 P19634

P63279 P19634

O00257 P19784

O15372 P19784

O60282 P19784

P04637 P19784

P07237 P19784

P16403 P19784

P21980 P19784

P67870 P19784

P68400 P19784

P78527 P19784

Q00536 P19784

Q14166 P19784

Q14781 P19784

Q14995 P19784

Q15649 P19784

Q8TAF3 P19784

Q96C86 P19784

Q96EB6 P19784

Q9BS34 P19784

Q9H492 P19784

Q9H5J8 P19784

Q9UQ88 P19784

Q9Y6N6 P19784

P10276 P19793

P11473 P19793

P24522 P19793

P27986 P19793

P36896 P19793

P42224 P19793

P53350 P19793

P55345 P19793

P67870 P19793

Q13133 P19793

Q14686 P19793

Q15648 P19793

Q15788 P19793

Q15796 P19793

Q16637 P19793

Q71SY5 P19793

Q9BZL6 P19793

P02768 P19827

P42336 P19827

O60271 P19838

P01011 P19838

P13797 P19838

P15104 P19838

P18859 P19838

P19838 P19838

P22736 P19838

P23396 P19838

P25490 P19838

P25963 P19838

P29590 P19838

P35222 P19838

P60520 P19838

Q00653 P19838

Q01082 P19838

Q01201 P19838

Q03468 P19838

Q04206 P19838

Q04864 P19838

Q06481 P19838

Q13547 P19838

Q13813 P19838

Q14690 P19838

Q15025 P19838

Q7L014 P19838

Q7Z4G1 P19838

Q86VX2 P19838

Q86X83 P19838

Q8IV08 P19838

Q8IZL8 P19838

Q8N668 P19838

Q8NFZ5 P19838

Q92887 P19838

Q92905 P19838

Q96BY9 P19838

Q9GZQ3 P19838

Q9GZQ8 P19838

Q9H0A8 P19838

Q9H492 P19838

Q9NX08 P19838

Q9NZW5 P19838

Q9P000 P19838

Q9UBI1 P19838

Q9UN37 P19838

Q9Y6G5 P19838

P41279 P19838

Q96EB6 P19838

Q12929 P19875

P04899 P19878

P14598 P19878

P15153 P19878

P60953 P19878

P62873 P19878

P63000 P19878

Q15080 P19878

Q92845 P19878

O43184 P19883

P03950 P19883

Q12841 P19883

Q14689 P19883

Q5EB52 P19883

P01100 P19961

P11274 P20020

P04233 P20036

P04637 P20042

P05198 P20042

P16104 P20042

P17096 P20042

P41091 P20042

P43146 P20042

P51693 P20042

P78344 P20042

Q04724 P20042

Q05516 P20042

Q13432 P20042

Q13573 P20042

Q14194 P20042

Q92900 P20042

Q9GZT6 P20042

Q9P2H0 P20042

Q9Y4G2 P20042

O14576 P20073

O14775 P20073

O60383 P20073

O75340 P20073

O75529 P20073

P01023 P20073

P04406 P20073

P04637 P20073

P06576 P20073

P07199 P20073

P13378 P20073

P14618 P20073

P17405 P20073

P25054 P20073

P26373 P20073

P27694 P20073

P38606 P20073

P51668 P20073

P51693 P20073

P52742 P20073

P54252 P20073

P60709 P20073

P62879 P20073

P68104 P20073

Q06710 P20073

Q09013 P20073

Q12766 P20073

Q13107 P20073

Q13432 P20073

Q13509 P20073

Q13573 P20073

Q15047 P20073

Q5SZQ8 P20073

Q6P4I2 P20073

Q6UXF1 P20073

Q75N90 P20073

Q8IV08 P20073

Q8IVF5 P20073

Q8NBE8 P20073

Q8NFF5 P20073

Q8WTW4 P20073

Q96FN4 P20073

Q99459 P20073

Q99832 P20073

Q9BT40 P20073

Q9BVA1 P20073

Q9H0R8 P20073

Q9H324 P20073

Q9P283 P20073

Q9UKR5 P20073

Q9UMN6 P20073

Q9Y3C7 P20073

Q9Y600 P20073

P29350 P20138

Q15034 P20138

P01023 P20160

O60869 P20226

O95402 P20226

P04637 P20226

P09429 P20226

P17844 P20226

P20265 P20226

P21675 P20226

P28347 P20226

P35226 P20226

P52655 P20226

P54257 P20226

P55317 P20226

Q00987 P20226

Q01105 P20226

Q12962 P20226

Q15572 P20226

Q15573 P20226

Q53T94 P20226

Q8IZX4 P20226

Q92994 P20226

Q96A83 P20226

Q9H5J8 P20226

Q9HC52 P20226

Q9P0U4 P20226

P06493 P20248

P14635 P20248

P19525 P20248

P24941 P20248

P38936 P20248

P46527 P20248

Q00526 P20248

Q00535 P20248

Q08999 P20248

Q13111 P20248

Q13415 P20248

Q13416 P20248

Q13627 P20248

Q96EV8 P20248

Q96PU4 P20248

Q96RL1 P20248

Q9H211 P20248

Q9Y463 P20248

O75807 P20264

P20265 P20265

P56693 P20265

Q00403 P20265

Q09472 P20265

P07948 P20273

P29350 P20273

P43405 P20273

P16104 P20290

Q0WX57 P20290

Q14164 P20290

P03372 P20290

Q8IZF0 P20309

Q96SB3 P20309

O00165 P20333

O14654 P20333

O14735 P20333

O14980 P20333

O14983 P20333

O15397 P20333

O43175 P20333

O43592 P20333

O95373 P20333

O95816 P20333

O95831 P20333

P01375 P20333

P04350 P20333

P04406 P20333

P05023 P20333

P05141 P20333

P07437 P20333

P07910 P20333

P08571 P20333

P08670 P20333

P0CG47 P20333

P11021 P20333

P11142 P20333

P11182 P20333

P12235 P20333

P12236 P20333

P14625 P20333

P16615 P20333

P17066 P20333

P17987 P20333

P20333 P20333

P21333 P20333

P23396 P20333

P25705 P20333

P27708 P20333

P28799 P20333

P29508 P20333

P31689 P20333

P34931 P20333

P35579 P20333

P35580 P20333

P38646 P20333

P39656 P20333

P42677 P20333

P43003 P20333

P51571 P20333

P53007 P20333

P53618 P20333

P55060 P20333

P61619 P20333

P62249 P20333

P62263 P20333

P62269 P20333

P62829 P20333

P63173 P20333

P63261 P20333

P78527 P20333

Q00325 P20333

Q00610 P20333

Q00839 P20333

Q01813 P20333

Q02978 P20333

Q05639 P20333

Q07021 P20333

Q12933 P20333

Q13257 P20333

Q13748 P20333

Q14257 P20333

Q14974 P20333

Q15006 P20333

Q15645 P20333

Q15758 P20333

Q16531 P20333

Q3ZCQ8 P20333

Q6AI08 P20333

Q71UM5 P20333

Q7Z3U7 P20333

Q8IUF1 P20333

Q92538 P20333

Q92616 P20333

Q96CX2 P20333

Q96EY1 P20333

Q9BVA1 P20333

Q9BXW9 P20333

Q9BYX7 P20333

Q9H1R3 P20333

Q9HAV4 P20333

Q9NQH7 P20333

Q9NVI1 P20333

Q9NVI7 P20333

Q9P035 P20333

Q9UM54 P20333

Q9UMX0 P20333

Q9UQC1 P20333

P00533 P20336

Q01968 P20336

O14964 P20338

O95704 P20338

P04179 P20338

P04637 P20338

P50395 P20338

Q4V328 P20338

Q9H5N1 P20338

Q9Y3P9 P20338

P22392 P20339

Q01968 P20339

Q15075 P20339

Q15276 P20339

Q92544 P20339

Q96Q42 P20339

Q9UI14 P20339

Q9UKG1 P20339

Q01968 P20340

Q8IWJ2 P20340

P27487 P20366

P46379 P20366

Q8TER5 P20366

P25103 P20366

P21452 P20366

P01106 P20585

P43246 P20585

Q8IY92 P20585

Q96PM5 P20585

P48995 P20591

Q9UBN4 P20591

Q9UL62 P20591

Q9Y210 P20591

O95166 P20618

P01106 P20618

P02751 P20618

P19320 P20618

P25786 P20618

P25787 P20618

P27824 P20618

P28074 P20618

P49720 P20618

P49721 P20618

P60900 P20618

Q15051 P20618

Q99436 P20618

Q9BQ83 P20618

Q9H0R8 P20618

Q9Y5K5 P20618

Q9UJY5 P20645

P38919 P20671

P61326 P20671

P83916 P20671

Q13185 P20671

P00403 P20674

P09669 P20674

Q02221 P20674

Q7L5N1 P20674

Q9UJS0 P20674

Q9Y2R0 P20674

O43918 P20700

O75928 P20700

O95229 P20700

P02545 P20700

P18848 P20700

P19320 P20700

P20700 P20700

P33993 P20700

P63104 P20700

Q5S007 P20700

Q8N0X7 P20700

Q9H221 P20700

P01106 P20701

P05107 P20702

Q04206 P20711

O15084 P20749

O95999 P20749

P06239 P20749

P06241 P20749

P19838 P20749

P56545 P20749

Q00653 P20749

Q01201 P20749

Q04206 P20749

Q04864 P20749

P00519 P20774

P46108 P20774

P23560 P20783

P10275 P20794

P62714 P20794

Q9NRX5 P20794

Q9UM11 P20794

Q9Y6Q9 P20794

P08253 P20807

P0C862 P20807

Q4J6C6 P20807

Q4VCS5 P20807

Q8N3K9 P20807

Q8WZ42 P20807

Q9H6Y7 P20807

P43364 P20809

P00519 P20810

P16333 P20810

P17655 P20810

P46108 P20810

P00167 P20815

Q8NBN7 P20815

Q96SQ7 P20815

P09661 P20823

P41182 P20823

P61457 P20823

P62158 P20823

Q15413 P20823

Q5H9L2 P20823

Q6FGD7 P20823

Q8IUC4 P20823

Q92484 P20823

Q9H0N5 P20823

Q9Y463 P20823

P02775 P20827

P26641 P20827

P29317 P20827

P61956 P20827

Q92993 P20827

O75496 P20839

Q92743 P20839

P05997 P20908

P20908 P20908

Q15051 P20916

O60662 P20929

O75386 P20929

O75923 P20929

O95425 P20929

P13929 P20929

P21399 P20929

P25054 P20929

P52907 P20929

P54296 P20929

Q07889 P20929

Q13188 P20929

Q13464 P20929

Q16539 P20929

Q86TC9 P20929

Q8IZP0 P20929

Q8WZ42 P20929

Q969Q1 P20929

Q96CV9 P20929

Q9BZ29 P20929

Q9H8T0 P20929

Q9HAU4 P20929

O94782 P20930

Q8IY92 P20930

Q92731 P20930

Q9GZQ8 P20930

P02768 P20933

O14543 P20936

O15117 P20936

P00533 P20936

P04626 P20936

P04632 P20936

P06127 P20936

P08069 P20936

P09619 P20936

P16333 P20936

P29622 P20936

P34741 P20936

P41567 P20936

Q07666 P20936

Q13480 P20936

Q8IZ69 P20936

Q8IZP0 P20936

Q8NAF0 P20936

Q96QB1 P20936

P17028 P20962

Q15796 P20962

Q9P0W5 P20962

Q9UGN5 P20962

P16333 P20963

P20963 P20963

P29353 P20963

P32248 P20963

P35227 P20963

P43403 P20963

Q9Y2R2 P20963

O94811 P21127

O95251 P21127

P19784 P21127

P62979 P21127

Q13153 P21127

Q16543 P21127

Q96CW1 P21127

Q9BTM9 P21127

P12931 P21145

P27986 P21145

Q06187 P21145

Q93009 P21145

Q99836 P21145

O43765 P21246

O60925 P21246

O95257 P21246

P01215 P21246

P05181 P21246

P21246 P21246

Q07817 P21246

Q12873 P21246

Q6P1K2 P21246

Q8N2W9 P21246

Q96D09 P21246

Q99689 P21246

Q9BPX7 P21246

Q9H5J8 P21246

Q9H9J2 P21246

Q9HC16 P21246

Q9UMX0 P21246

P21266 P21266

P62993 P21266

Q08999 P21266

P01023 P21281

P02751 P21281

P38606 P21281

Q6IAA8 P21281

P38606 P21283

P62330 P21283

P62993 P21291

A0AUL9 P21333

O14786 P21333

O75369 P21333

O95067 P21333

P00519 P21333

P01106 P21333

P02751 P21333

P03372 P21333

P05556 P21333

P08238 P21333

P10242 P21333

P12931 P21333

P13612 P21333

P16144 P21333

P19320 P21333

P21333 P21333

P22694 P21333

P26010 P21333

P32121 P21333

P35372 P21333

P46781 P21333

P49286 P21333

P49407 P21333

P49768 P21333

P62993 P21333

P85299 P21333

Q06187 P21333

Q07157 P21333

Q09019 P21333

Q12948 P21333

Q13283 P21333

Q15051 P21333

Q16539 P21333

Q5VV17 P21333

Q70EL2 P21333

Q86SQ0 P21333

Q8IY92 P21333

Q8IYT8 P21333

Q8N264 P21333

Q8WZ42 P21333

Q96G74 P21333

Q96RR4 P21333

Q9H0R8 P21333

Q9H9G7 P21333

Q9Y6K9 P21333

P05067 P21359

P29590 P21359

P34741 P21359

P58753 P21359

Q14204 P21359

Q99459 P21359

P01023 P21397

A6NI79 P21399

Q00610 P21399

Q13326 P21399

Q3MUY2 P21439

P20366 P21452

Q13263 P21506

Q9GZM8 P21506

P29274 P21554

P42566 P21579

P54284 P21579

Q9BT78 P21579

P01375 P21580

P08238 P21580

P0CG48 P21580

P21580 P21580

P62258 P21580

P63104 P21580

Q13601 P21580

Q86VP1 P21580

Q9H0F6 P21580

Q9Y3C5 P21580

Q9Y6K9 P21580

P10721 P21583

P21583 P21583

O15197 P21673

O60383 P21673

O60512 P21673

O95251 P21673

P04637 P21673

P05386 P21673

P21246 P21673

P21673 P21673

P22459 P21673

P26641 P21673

P32119 P21673

P32456 P21673

P49903 P21673

P50552 P21673

P51693 P21673

P61604 P21673

Q04724 P21673

Q12873 P21673

Q13432 P21673

Q13574 P21673

Q13885 P21673

Q14194 P21673

Q15047 P21673

Q5RL73 P21673

Q5UIP0 P21673

Q6PI98 P21673

Q7L5N1 P21673

Q8IY81 P21673

Q8NFR7 P21673

Q8TBB1 P21673

Q92993 P21673

Q96BY9 P21673

Q96F10 P21673

Q96I25 P21673

Q9BQ70 P21673

Q9BSH4 P21673

Q9BWH2 P21673

Q9GZT6 P21673

Q9GZT8 P21673

Q9H8M9 P21673

Q9HBL7 P21673

Q9NUT2 P21673

Q9P2H0 P21673

Q9UKR5 P21673

Q9UKY1 P21673

Q9ULA0 P21673

Q9Y2A0 P21673

Q9Y383 P21673

Q9Y3C7 P21673

Q9Y3Q8 P21673

O95402 P21675

P18084 P21675

P20226 P21675

P35269 P21675

P54296 P21675

P61964 P21675

Q16586 P21675

Q5VT25 P21675

Q8N3C7 P21675

Q9HCU9 P21675

P21246 P21695

P32189 P21695

Q9Y244 P21695

P08238 P21709

P27824 P21731

Q5JY77 P21731

P78362 P21741

Q9NRR5 P21741

Q15051 P21757

Q86VS8 P21757

P21802 P21781

Q9UMX0 P21781

O14920 P21796

P00505 P21796

P01023 P21796

P06753 P21796

P15336 P21796

P16152 P21796

P19320 P21796

P19367 P21796

P27824 P21796

P33176 P21796

P37840 P21796

P60709 P21796

P81605 P21796

Q12933 P21796

Q13268 P21796

Q14197 P21796

Q14697 P21796

Q15459 P21796

Q16611 P21796

Q16658 P21796

Q8N9P6 P21796

Q8WZ42 P21796

Q9BWD1 P21796

Q9H845 P21796

Q9Y266 P21796

P05230 P21802

P09038 P21802

P21802 P21802

P35813 P21802

P62993 P21802

P21781 P21802

O75386 P21817

P02787 P21817

P19174 P21817

P54296 P21817

P62136 P21817

Q8WZ42 P21817

O00459 P21860

O60880 P21860

O75553 P21860

P00519 P21860

P00533 P21860

P04626 P21860

P08631 P21860

P12931 P21860

P19174 P21860

P20936 P21860

P21860 P21860

P27986 P21860

P29353 P21860

P42336 P21860

P42681 P21860

P42684 P21860

P43405 P21860

P46109 P21860

P52735 P21860

P52757 P21860

P62993 P21860

Q02297 P21860

Q08881 P21860

Q13671 P21860

Q14451 P21860

Q15303 P21860

Q63HR2 P21860

Q68CZ2 P21860

Q8IZW8 P21860

Q92529 P21860

Q92569 P21860

Q96D37 P21860

Q9UN19 P21860

Q9UQ80 P21860

Q9UQQ2 P21860

O95166 P21912

P19320 P21912

P31040 P21912

Q16595 P21912

Q99643 P21912

Q9H0R8 P21912

Q9H2V7 P21912

P21917 P21917

P08473 P21926

P38606 P21926

P12694 P21953

P02751 P21964

A8K5I0 P21980

O15084 P21980

O95477 P21980

P00738 P21980

P01023 P21980

P01024 P21980

P02751 P21980

P05067 P21980

P08107 P21980

P0CG48 P21980

P11142 P21980

P12830 P21980

P21980 P21980

P25963 P21980

P28799 P21980

P39060 P21980

P40337 P21980

P47871 P21980

P82251 P21980

P84022 P21980

Q04206 P21980

Q12802 P21980

Q15796 P21980

Q96HP8 P21980

Q9BUJ2 P21980

Q9NYJ8 P21980

Q9UHV2 P21980

P61457 P22033

Q14197 P22033

P08047 P22059

P68400 P22059

P02647 P22061

P13569 P22061

P19320 P22061

P22061 P22061

P60520 P22061

P62993 P22061

Q12873 P22061

Q9GZQ8 P22061

Q9H492 P22061

Q00005 P22061

A0JLT2 P22087

O95166 P22087

P01106 P22087

P11387 P22087

P17844 P22087

P19320 P22087

P32121 P22087

P51858 P22087

P67809 P22087

Q16637 P22087

Q92905 P22087

Q96RS0 P22087

Q9BXL5 P22087

Q9HA65 P22087

Q9HC52 P22087

Q9NY93 P22087

Q9UKV8 P22087

Q92731 P22090

P02751 P22102

P19320 P22102

P52292 P22102

Q9H0R8 P22102

Q9H492 P22102

O00716 P22105

P08069 P22223

P46937 P22223

Q96CW1 P22223

O15264 P22234

P02751 P22234

P19320 P22234

P22234 P22234

P60520 P22234

Q12873 P22234

Q6ZVK8 P22234

Q8TAP6 P22234

Q8TBB1 P22234

Q96GM5 P22234

Q9GZQ8 P22234

Q9GZT8 P22234

Q9H0R8 P22234

Q13651 P22301

P06733 P22303

P17544 P22303

P22303 P22303

P63244 P22303

Q9Y215 P22303

P01100 P22304

O75381 P22307

Q03135 P22307

Q2T9J0 P22307

O15151 P22314

O15264 P22314

O43684 P22314

O75069 P22314

P01106 P22314

P08238 P22314

P30084 P22314

P35222 P22314

P43356 P22314

P46527 P22314

P63104 P22314

Q13275 P22314

Q16594 P22314

Q5VTR2 P22314

Q6DKK2 P22314

Q99717 P22314

Q9BXL5 P22314

Q9H2C0 P22314

Q9UBF1 P22314

Q9Y5U2 P22314

O00232 P22392

O00264 P22392

O00746 P22392

O14713 P22392

O14818 P22392

O60518 P22392

P01616 P22392

P04844 P22392

P05455 P22392

P15531 P22392

P16152 P22392

P22392 P22392

P25787 P22392

P28070 P22392

P28222 P22392

P31939 P22392

P31948 P22392

P33176 P22392

P39019 P22392

P50502 P22392

P52434 P22392

P53396 P22392

P54725 P22392

P54819 P22392

P58107 P22392

P61086 P22392

P61088 P22392

P61326 P22392

P61626 P22392

P62249 P22392

P62807 P22392

P63104 P22392

P63173 P22392

P68402 P22392

Q00005 P22392

Q01105 P22392

Q12972 P22392

Q13283 P22392

Q13363 P22392

Q14161 P22392

Q14204 P22392

Q15084 P22392

Q15365 P22392

Q15435 P22392

Q6FHQ0 P22392

Q7L2H7 P22392

Q8IZL9 P22392

Q9HB71 P22392

Q9NPH2 P22392

Q9NQC3 P22392

Q9NZ23 P22392

Q9Y230 P22392

Q9Y2L1 P22392

Q9Y333 P22392

Q9Y3F4 P22392

Q13501 P22413

P78527 P22415

Q92831 P22415

Q9H8W4 P22415

P50750 P22455

P35609 P22459

Q9NRX3 P22459

O43603 P22466

P47211 P22466

P63104 P22492

Q8TF42 P22492

Q92731 P22528

P62993 P22557

P10809 P22570

P49789 P22570

O75380 P22607

P02008 P22607

P05060 P22607

P05787 P22607

P08238 P22607

P12236 P22607

P18847 P22607

P22607 P22607

P43235 P22607

P62917 P22607

Q12789 P22607

Q14181 P22607

Q15486 P22607

Q6PGQ7 P22607

Q75LH2 P22607

Q86XS8 P22607

Q92540 P22607

Q96LX7 P22607

Q96P48 P22607

Q9P2H0 P22607

P17612 P22612

Q00537 P22612

B2Y833 P22626

O14879 P22626

O95166 P22626

P03372 P22626

P09913 P22626

P11021 P22626

P11387 P22626

P19320 P22626

P21860 P22626

P22626 P22626

P22736 P22626

P29353 P22626

P42771 P22626

P51858 P22626

P60520 P22626

P60709 P22626

P61244 P22626

P62993 P22626

P63104 P22626

P67809 P22626

P98179 P22626

Q12851 P22626

Q13573 P22626

Q15051 P22626

Q16637 P22626

Q17RG0 P22626

Q6MZP7 P22626

Q8IYT8 P22626

Q8TDY2 P22626

Q8TF42 P22626

Q96CW1 P22626

Q96GY3 P22626

Q99459 P22626

Q9BXW4 P22626

Q9GZQ8 P22626

Q9H0R8 P22626

Q9H492 P22626

Q9HA38 P22626

Q9NRH1 P22626

P68400 P22626

Q00005 P22626

P16104 P22626

O15198 P22670

P35611 P22670

Q13485 P22670

Q99459 P22670

P52735 P22674

A9UF07 P22681

O00459 P22681

O14492 P22681

O15357 P22681

O43597 P22681

P00519 P22681

P00533 P22681

P04629 P22681

P06239 P22681

P06241 P22681

P07948 P22681

P07949 P22681

P08581 P22681

P08842 P22681

P09619 P22681

P12314 P22681

P12931 P22681

P16333 P22681

P19320 P22681

P23467 P22681

P23470 P22681

P27348 P22681

P27986 P22681

P29353 P22681

P31946 P22681

P31947 P22681

P43405 P22681

P45983 P22681

P46108 P22681

P46109 P22681

P51451 P22681

P52735 P22681

P55085 P22681

P61981 P22681

P62258 P22681

P62993 P22681

P63104 P22681

Q07666 P22681

Q13094 P22681

Q13196 P22681

Q14155 P22681

Q15811 P22681

Q7Z698 P22681

Q8IZP0 P22681

Q96B97 P22681

Q96J02 P22681

Q9BUZ4 P22681

Q9BZR9 P22681

Q9C004 P22681

Q9Y2R2 P22681

Q9Y5K6 P22681

O43463 P22692

P08238 P22694

P17612 P22694

P10644 P22694

P68400 P22694

Q92917 P22694

O00555 P22695

P08574 P22695

P13489 P22695

P15336 P22695

P31930 P22695

Q14197 P22695

Q9BQ83 P22695

P60520 P22735

Q9H0R8 P22735

O15069 P22736

O43236 P22736

O60888 P22736

O75970 P22736

O95342 P22736

O96017 P22736

P00367 P22736

P04150 P22736

P04350 P22736

P04637 P22736

P07199 P22736

P08238 P22736

P10415 P22736

P10909 P22736

P15884 P22736

P29622 P22736

P32189 P22736

P35813 P22736

P40763 P22736

P40939 P22736

P43243 P22736

P48449 P22736

P50416 P22736

P50552 P22736

P54274 P22736

P62195 P22736

P84022 P22736

Q12873 P22736

Q13162 P22736

Q14469 P22736

Q14802 P22736

Q14839 P22736

Q16548 P22736

Q86WJ1 P22736

Q8NAF0 P22736

Q8WWQ0 P22736

Q8WWZ7 P22736

Q92598 P22736

Q9H221 P22736

Q9H501 P22736

Q9HC38 P22736

Q9P2G1 P22736

Q9UQN3 P22736

Q9Y2Z0 P22736

Q9Y3C7 P22736

Q9Y4B6 P22736

P32119 P22748

Q9NWS0 P22748

Q9Y6R1 P22748

P51449 P22794

P08473 P22830

Q9H9J4 P22830

Q9Y2K6 P22830

P15927 P23025

Q96EB6 P23025

Q9HCN4 P23025

Q9NS23 P23025

O95166 P23109

Q9GZQ8 P23109

Q9H0R8 P23109

Q9H492 P23109

P02647 P23142

P27348 P23142

Q6UY14 P23142

Q86TH1 P23142

Q92844 P23142

Q9NQB0 P23142

Q9UM47 P23142

Q9Y3D8 P23142

Q8WVC0 P23193

P16144 P23229

P21926 P23229

P30101 P23229

O15264 P23246

O75554 P23246

O95166 P23246

P00533 P23246

P08621 P23246

P09012 P23246

P09651 P23246

P0CG47 P23246

P11387 P23246

P19320 P23246

P23246 P23246

P26599 P23246

P32121 P23246

P35637 P23246

P49841 P23246

P60520 P23246

P63000 P23246

Q06187 P23246

Q13573 P23246

Q13882 P23246

Q16637 P23246

Q68DV7 P23246

Q8WXF1 P23246

Q92574 P23246

Q96ST3 P23246

Q99459 P23246

Q9GZQ8 P23246

Q9H0R8 P23246

Q9H492 P23246

Q9NZC7 P23246

Q9UBW7 P23246

Q9UK76 P23246

Q9Y2W1 P23246

B2RWN4 P23258

O15169 P23258

P01106 P23258

P63104 P23258

Q04917 P23258

Q08AG7 P23258

Q13573 P23258

Q53EZ4 P23258

Q6NZ67 P23258

Q96CW5 P23258

Q96G01 P23258

Q96L34 P23258

Q99459 P23258

Q99996 P23258

Q9BVR6 P23258

Q9NRI5 P23258

Q9NYZ3 P23258

Q71U36 P23276

P15336 P23284

Q8TAF3 P23284

Q9NRR5 P23284

Q9UK80 P23284

P04271 P23297

P04637 P23297

P23297 P23297

P25815 P23297

P26447 P23297

Q00987 P23297

Q9GZT8 P23297

P11362 P23352

P21802 P23352

P31939 P23368

P62491 P23368

Q14197 P23368

Q9H4A3 P23368

Q9Y4E8 P23368

Q14197 P23378

Q8IYT8 P23378

Q96KP6 P23378

Q9BW61 P23381

Q9NV06 P23381

Q9UBC3 P23381

O00571 P23396

O00716 P23396

O14920 P23396

O15264 P23396

O95166 P23396

P03372 P23396

P19320 P23396

P19838 P23396

P32121 P23396

P38919 P23396

P60520 P23396

P61326 P23396

P62993 P23396

P63104 P23396

P63244 P23396

Q04206 P23396

Q04609 P23396

Q06187 P23396

Q08752 P23396

Q13573 P23396

Q15843 P23396

Q92597 P23396

Q92731 P23396

Q92900 P23396

Q9GZQ8 P23396

Q9H0R8 P23396

Q9H492 P23396

Q9UBU9 P23396

Q9UKV8 P23396

Q9UL18 P23396

O43504 P23409

Q99081 P23409

P46379 P23416

Q15436 P23416

Q96I30 P23416

Q9GZR7 P23416

Q05516 P23434

Q8WYH8 P23434

O15530 P23443

P06730 P23443

P08238 P23443

P10636 P23443

P43629 P23443

P45983 P23443

P55884 P23443

P60953 P23443

Q00005 P23443

Q09161 P23443

Q13057 P23443

Q15118 P23443

Q7L190 P23443

P08151 P23443

P08238 P23458

P08887 P23458

P10276 P23458

P17252 P23458

P48551 P23458

P58753 P23458

Q9HCK5 P23458

P00533 P23467

P04626 P23467

P04629 P23467

P06213 P23467

P09619 P23467

P17948 P23467

P19022 P23467

P19235 P23467

P23467 P23467

P27361 P23467

P28482 P23467

P35222 P23467

P41240 P23467

P49023 P23467

Q02763 P23467

P00533 P23468

P23468 P23468

P09619 P23469

P23469 P23469

O15530 P23470

P04626 P23470

P04629 P23470

P06239 P23470

P08581 P23470

P09619 P23470

P23470 P23470

P27361 P23470

P35222 P23470

P35590 P23470

P42680 P23470

P42768 P23470

Q13224 P23470

Q9UM73 P23470

P06239 P23471

P06756 P23471

P23471 P23471

P41240 P23471

Q9UM73 P23471

P08670 P23490

P14921 P23497

P23497 P23497

P55854 P23497

P63165 P23497

Q03060 P23497

Q8TAD8 P23497

Q9BXL5 P23497

Q9UKL3 P23497

O00193 P23508

O00264 P23508

O00299 P23508

O00303 P23508

O00487 P23508

O14737 P23508

O14818 P23508

O15067 P23508

O15371 P23508

O43169 P23508

O43809 P23508

O43815 P23508

O60493 P23508

O60888 P23508

O75390 P23508

O75534 P23508

O75643 P23508

O75694 P23508

O75934 P23508

O95163 P23508

O95202 P23508

O95757 P23508

P00491 P23508

P00492 P23508

P00505 P23508

P02786 P23508

P04179 P23508

P04181 P23508

P05783 P23508

P06748 P23508

P06753 P23508

P07355 P23508

P07741 P23508

P07951 P23508

P08134 P23508

P08237 P23508

P08579 P23508

P09936 P23508

P09960 P23508

P10768 P23508

P11177 P23508

P11586 P23508

P11802 P23508

P12004 P23508

P13798 P23508

P13804 P23508

P13995 P23508

P15170 P23508

P15311 P23508

P16152 P23508

P19105 P23508

P21796 P23508

P21912 P23508

P21964 P23508

P22061 P23508

P22234 P23508

P23284 P23508

P23526 P23508

P23921 P23508

P24534 P23508

P24752 P23508

P25205 P23508

P25786 P23508

P25788 P23508

P26639 P23508

P26640 P23508

P27695 P23508

P29084 P23508

P29144 P23508

P29401 P23508

P29692 P23508

P30040 P23508

P30084 P23508

P30085 P23508

P30153 P23508

P33991 P23508

P33993 P23508

P35580 P23508

P35606 P23508

P36507 P23508

P37802 P23508

P37837 P23508

P39656 P23508

P41227 P23508

P42704 P23508

P42771 P23508

P43246 P23508

P43487 P23508

P43897 P23508

P45973 P23508

P46926 P23508

P47756 P23508

P48507 P23508

P48556 P23508

P48730 P23508

P49321 P23508

P49411 P23508

P49674 P23508

P49720 P23508

P50570 P23508

P51149 P23508

P51452 P23508

P51812 P23508

P52701 P23508

P52907 P23508

P54819 P23508

P55072 P23508

P55735 P23508

P56192 P23508

P56537 P23508

P57721 P23508

P60900 P23508

P61019 P23508

P61081 P23508

P61224 P23508

P61247 P23508

P61964 P23508

P62263 P23508

P62714 P23508

P62750 P23508

P62873 P23508

P62993 P23508

P68402 P23508

P83731 P23508

P99999 P23508

Q00059 P23508

Q00610 P23508

Q00688 P23508

Q01469 P23508

Q04323 P23508

Q04637 P23508

Q08257 P23508

Q08945 P23508

Q12904 P23508

Q13011 P23508

Q13033 P23508

Q13085 P23508

Q13200 P23508

Q13347 P23508

Q13439 P23508

Q13616 P23508

Q13617 P23508

Q13642 P23508

Q14152 P23508

Q14160 P23508

Q14165 P23508

Q14197 P23508

Q14232 P23508

Q14566 P23508

Q15008 P23508

Q15019 P23508

Q15185 P23508

Q15631 P23508

Q15691 P23508

Q16768 P23508

Q53Y06 P23508

Q5SQT9 P23508

Q6FGD7 P23508

Q6FHT8 P23508

Q6P1L8 P23508

Q7L5N1 P23508

Q8IYT4 P23508

Q8TCS8 P23508

Q8WUM4 P23508

Q92797 P23508

Q96AG4 P23508

Q96C36 P23508

Q96CX2 P23508

Q96EY4 P23508

Q96FW1 P23508

Q96GG9 P23508

Q96PZ0 P23508

Q96QK1 P23508

Q96S44 P23508

Q96T76 P23508

Q99623 P23508

Q9BUL8 P23508

Q9BWD1 P23508

Q9BXJ9 P23508

Q9H0U4 P23508

Q9H3N1 P23508

Q9H9B4 P23508

Q9HB71 P23508

Q9HC36 P23508

Q9HC38 P23508

Q9NPJ3 P23508

Q9NR30 P23508

Q9NRL3 P23508

Q9NTJ3 P23508

Q9NYU2 P23508

Q9UBT2 P23508

Q9UGP8 P23508

Q9UHV9 P23508

Q9UI30 P23508

Q9UJF2 P23508

Q9UJZ1 P23508

Q9UKK9 P23508

Q9UL25 P23508

Q9UNM6 P23508

Q9UQE7 P23508

Q9Y295 P23508

Q9Y3A3 P23508

Q9Y3B4 P23508

Q9Y3B8 P23508

Q9Y3D3 P23508

Q9Y4L1 P23508

Q9Y5B9 P23508

Q9Y696 P23508

Q9Y6E0 P23508

P04637 P23511

P25208 P23511

P30304 P23511

Q07955 P23511

Q12772 P23511

Q92624 P23511

O15264 P23526

O60739 P23526

O95166 P23526

P15336 P23526

Q8TDR0 P23526

Q9GZQ8 P23526

Q9H0R8 P23526

Q9H492 P23526

P32121 P23527

P49407 P23527

O95758 P23528

P00533 P23528

P01106 P23528

P02751 P23528

P15336 P23528

P16104 P23528

P17612 P23528

P19320 P23528

P32121 P23528

P49407 P23528

P49768 P23528

P60709 P23528

P62993 P23528

P63104 P23528

Q01995 P23528

Q13387 P23528

Q13393 P23528

Q15418 P23528

Q15750 P23528

Q9H0R8 P23528

Q9H492 P23528

Q9Y281 P23528

Q9BT40 P23560

Q9Y624 P23560

O15372 P23588

O43504 P23588

P23588 P23588

P32121 P23588

P49407 P23588

P51784 P23588

P53350 P23588

P68400 P23588

Q13557 P23588

Q14152 P23588

Q8WVZ9 P23588

Q93009 P23588

Q9H9G7 P23588

Q9HCK5 P23588

Q9UKV8 P23588

P00533 P23634

P01258 P23634

P50613 P23634

P62258 P23634

Q13586 P23634

Q15796 P23743

P46108 P23759

Q13642 P23759

P00519 P23760

P12931 P23760

P20265 P23760

P50222 P23760

P15923 P23769

P17542 P23769

Q13485 P23769

P17542 P23771

Q14197 P23786

P56937 P23919

Q99459 P23919

P01106 P23921

P31350 P23921

Q5UIP0 P23921

P01225 P23945

P27348 P23945

Q9NRD5 P23975

P01106 P24046

P01876 P24071

P01023 P24158

P84022 P24158

O60504 P24278

P38432 P24278

P49903 P24278

P51589 P24278

Q9BQD3 P24278

O00311 P24298

Q9UJS0 P24311

O95273 P24385

P06858 P24385

P11142 P24385

P11802 P24385

P24941 P24385

P38398 P24385

P38936 P24385

P62140 P24385

Q00534 P24385

Q15276 P24385

Q9BQA1 P24385

Q9H9Y6 P24385

Q92731 P24386

P24390 P24390

Q15436 P24390

P01106 P24394

P05112 P24394

P24394 P24394

P29350 P24394

P31785 P24394

P42226 P24394

P78552 P24394

Q13323 P24468

Q13573 P24468

Q5TF85 P24468

Q9H422 P24468

O75165 P24522

O75689 P24522

P01871 P24522

P05452 P24522

P15880 P24522

P21246 P24522

P25445 P24522

P26641 P24522

P47897 P24522

P51668 P24522

P52742 P24522

P61968 P24522

P62879 P24522

P80303 P24522

Q04724 P24522

Q06330 P24522

Q13561 P24522

Q13569 P24522

Q15262 P24522

Q16877 P24522

Q504T8 P24522

Q6UXH1 P24522

Q75N90 P24522

Q8IWV7 P24522

Q8N205 P24522

Q8TAE8 P24522

Q8TDZ2 P24522

Q92945 P24522

Q9GZX7 P24522

Q9H254 P24522

Q9UKY1 P24522

Q9UQ88 P24522

Q9Y371 P24522

O15264 P24534

O95166 P24534

P02751 P24534

P16104 P24534

P19320 P24534

P26641 P24534

P60520 P24534

P60709 P24534

P68400 P24534

Q00005 P24534

Q13268 P24534

Q14240 P24534

Q9GZQ8 P24534

Q9H0R8 P24534

Q9HCC6 P24534

Q14197 P24539

Q99816 P24539

Q9H845 P24539

P13861 P24588

P17612 P24588

P22694 P24588

P63252 P24588

Q08499 P24588

Q9HBX9 P24588

Q9NRR5 P24592

P46379 P24593

Q13642 P24593

Q9NRX1 P24593

P04150 P24666

P19320 P24666

P24666 P24666

Q5T0N5 P24666

Q9BYC9 P24666

Q9UHJ3 P24666

Q8IWT0 P24723

P19320 P24752

P21549 P24752

P60520 P24752

A0JLT2 P24863

O95402 P24863

P17022 P24863

P49336 P24863

Q9BTT4 P24863

Q9BWU1 P24863

Q9NX70 P24863

O60934 P24864

P24941 P24864

P38398 P24864

P38936 P24864

P46527 P24864

P49959 P24864

Q00526 P24864

Q08050 P24864

Q969H0 P24864

Q96PU4 P24864

A0JLT2 P24928

O60477 P24928

O95163 P24928

O95402 P24928

O95758 P24928

P01106 P24928

P17844 P24928

P23193 P24928

P62993 P24928

Q03468 P24928

Q08945 P24928

Q13573 P24928

Q5BKZ1 P24928

Q6P1J9 P24928

Q8N163 P24928

Q8N7H5 P24928

Q92541 P24928

Q96DB2 P24928

Q9BTT4 P24928

Q9H0R8 P24928

Q9H3P2 P24928

Q9HCS7 P24928

Q9NQG5 P24928

Q9NWA0 P24928

Q9NX70 P24928

Q9Y224 P24928

O15294 P24941

O95067 P24941

O95758 P24941

O96020 P24941

P00505 P24941

P05109 P24941

P06400 P24941

P11171 P24941

P11586 P24941

P11940 P24941

P13535 P24941

P20248 P24941

P22087 P24941

P24864 P24941

P24941 P24941

P26038 P24941

P29590 P24941

P30086 P24941

P31939 P24941

P37802 P24941

P38936 P24941

P46527 P24941

P51946 P24941

P61081 P24941

P62136 P24941

P62249 P24941

P78396 P24941

Q01469 P24941

Q08999 P24941

Q09472 P24941

Q13111 P24941

Q13261 P24941

Q13309 P24941

Q13415 P24941

Q14CX7 P24941

Q15714 P24941

Q15843 P24941

Q16667 P24941

Q6FGD7 P24941

Q8WWL7 P24941

Q969H0 P24941

Q96JH8 P24941

Q96PU4 P24941

Q96Q40 P24941

Q99523 P24941

Q9H1Q7 P24941

Q9H211 P24941

Q9H3J6 P24941

Q9UBI4 P24941

Q9Y5J5 P24941

P06493 P25024

Q71U36 P25024

P25025 P25025

P42261 P25025

A8CG34 P25054

O14640 P25054

O14641 P25054

O14964 P25054

O15169 P25054

O60318 P25054

O94910 P25054

O94979 P25054

O95758 P25054

P02533 P25054

P0C0L4 P25054

P12814 P25054

P13647 P25054

P19012 P25054

P19474 P25054

P21333 P25054

P21675 P25054

P31947 P25054

P35221 P25054

P35222 P25054

P35749 P25054

P42677 P25054

P49674 P25054

P49792 P25054

P49841 P25054

P52594 P25054

P52948 P25054

P54792 P25054

P63010 P25054

Q01082 P25054

Q03001 P25054

Q0VF96 P25054

Q12923 P25054

Q14160 P25054

Q15691 P25054

Q16363 P25054

Q7Z3B4 P25054

Q86T90 P25054

Q8IVI9 P25054

Q8IVL1 P25054

Q8NEY1 P25054

Q8TF01 P25054

Q96HA1 P25054

Q96KQ4 P25054

Q96RT1 P25054

Q99814 P25054

Q9H2Y7 P25054

Q9HCH0 P25054

Q9UQE7 P25054

Q9Y297 P25054

Q9Y613 P25054

P07948 P25063

P25067 P25067

P27658 P25067

Q86UT5 P25092

P00533 P25098

P0CG48 P25098

P21462 P25098

Q00987 P25098

Q9UBN7 P25098

P20366 P25103

P62158 P25105

P62993 P25106

Q03135 P25116

Q15363 P25116

Q9UNN8 P25116

Q96BK5 P25189

O00311 P25205

O43913 P25205

O43929 P25205

O95166 P25205

P02751 P25205

P19320 P25205

P24941 P25205

P27695 P25205

P30153 P25205

P30154 P25205

P32121 P25205

P33992 P25205

P33993 P25205

P49736 P25205

P53350 P25205

P62805 P25205

P63104 P25205

P68400 P25205

Q09472 P25205

Q12905 P25205

Q13416 P25205

Q14566 P25205

Q99459 P25205

Q99741 P25205

Q9BTE3 P25205

Q9BXL5 P25205

Q9GZQ8 P25205

Q9H0R8 P25205

Q9H492 P25205

P04637 P25208

P23511 P25208

P62993 P25208

Q13952 P25208

Q14919 P25208

Q9H7B4 P25208

P33527 P25311

P60520 P25311

Q70EK8 P25311

Q9BWU1 P25311

Q9BZD4 P25311

P24386 P25325

P45984 P25391

P03372 P25398

P60709 P25398

P61457 P25398

P62280 P25398

Q5U5Q3 P25398

Q8NB14 P25398

Q9HCK5 P25398

P19784 P25440

P62805 P25440

Q99459 P25440

O15273 P25445

O75340 P25445

O95243 P25445

P06241 P25445

P07910 P25445

P12931 P25445

P25445 P25445

P29590 P25445

P48023 P25445

P62158 P25445

P63165 P25445

P68104 P25445

Q03135 P25445

Q12923 P25445

Q13158 P25445

Q14194 P25445

Q14790 P25445

Q15156 P25445

Q5UIP0 P25445

Q92835 P25445

Q99683 P25445

Q9UER7 P25445

Q9UKR5 P25445

O43609 P25490

O96019 P25490

P25490 P25490

P41182 P25490

P46109 P25490

P49407 P25490

P55055 P25490

P78527 P25490

P84022 P25490

Q00987 P25490

Q13573 P25490

Q15569 P25490

Q15796 P25490

Q8IY57 P25490

Q8NBZ0 P25490

Q96PU4 P25490

Q9ULG1 P25490

Q9Y230 P25490

Q9Y265 P25490

Q9Y5K5 P25490

A0AUL9 P25685

P02751 P25685

P10632 P25685

P13569 P25685

P49810 P25685

Q01804 P25685

Q16543 P25685

Q2TAZ0 P25685

Q53GL0 P25685

Q9BQ95 P25685

Q9H0R8 P25685

Q9Y2R0 P25685

Q9Y4P8 P25685

Q9Y4R8 P25685

P04626 P25686

Q96DB2 P25686

B2Y833 P25705

O15264 P25705

O95166 P25705

P00533 P25705

P01023 P25705

P02751 P25705

P13489 P25705

P19320 P25705

P24539 P25705

P49407 P25705

P60520 P25705

P60709 P25705

P62993 P25705

P63104 P25705

P68400 P25705

P78537 P25705

Q06187 P25705

Q14197 P25705

Q15051 P25705

Q8N5M1 P25705

Q92905 P25705

Q9GZQ8 P25705

Q9H0R8 P25705

Q9H3D4 P25705

Q9H492 P25705

Q9H845 P25705

Q9NTG7 P25705

O14818 P25786

O95073 P25786

O95751 P25786

P00403 P25786

P01106 P25786

P08670 P25786

P19320 P25786

P25787 P25786

P38432 P25786

P60900 P25786

P62191 P25786

P62333 P25786

Q13137 P25786

Q13287 P25786

Q15051 P25786

Q15834 P25786

Q8N5Z5 P25786

Q96HT8 P25786

Q9BPX1 P25786

Q9BQ83 P25786

Q9UPY8 P25786

Q9Y3Q8 P25786

Q9Y5K5 P25786

Q9Y620 P25786

O14818 P25787

P01106 P25787

P02751 P25787

P25786 P25787

P25789 P25787

P60900 P25787

Q15051 P25787

Q9BQ83 P25787

Q9UKE5 P25787

Q9Y5K5 P25787

O14640 P25788

O14818 P25788

O60739 P25788

P01106 P25788

P14678 P25788

P18428 P25788

P19320 P25788

P25786 P25788

P25787 P25788

P25788 P25788

P25789 P25788

P28074 P25788

P29590 P25788

P55036 P25788

P60900 P25788

Q00987 P25788

Q15051 P25788

Q92837 P25788

Q96GD4 P25788

Q9BQ83 P25788

Q9NQB0 P25788

Q9Y244 P25788

Q9Y297 P25788

Q9Y5K5 P25788

Q9Y6R0 P25788

P68400 P25788

O14818 P25789

P01106 P25789

P19320 P25789

P25786 P25789

P25787 P25789

P25788 P25789

P25789 P25789

P60900 P25789

Q15051 P25789

Q969U7 P25789

Q99436 P25789

Q9BQ83 P25789

Q9NWV8 P25789

Q9Y244 P25789

Q9Y5K5 P25789

O15169 P25791

P42224 P25791

P55196 P25791

Q02252 P25791

Q04206 P25791

Q15038 P25791

Q15555 P25791

Q86U70 P25791

Q8IXK0 P25791

Q96KQ7 P25791

Q96RT1 P25791

Q9NVW2 P25791

Q9UPY8 P25791

P23297 P25815

P25815 P25815

Q15109 P25815

Q9UQ07 P25815

Q9NQ11 P25929

P20908 P25940

O00141 P25942

O15294 P25942

O75449 P25942

P10599 P25942

P22061 P25942

P22087 P25942

P27797 P25942

P40937 P25942

P52434 P25942

Q12933 P25942

Q13114 P25942

Q8NEW0 P25942

Q92504 P25942

Q99417 P25942

Q99460 P25942

Q9BS26 P25942

Q9NYU2 P25942

Q9Y4E8 P25942

D3DR86 P25963

O00411 P25963

O14920 P25963

O15111 P25963

O95163 P25963

P04083 P25963

P05141 P25963

P07437 P25963

P11021 P25963

P11142 P25963

P18031 P25963

P19838 P25963

P23396 P25963

P25205 P25963

P25963 P25963

P27986 P25963

P33992 P25963

P33993 P25963

P34931 P25963

P38646 P25963

P49407 P25963

P55072 P25963

P61201 P25963

P61457 P25963

P62158 P25963

P62829 P25963

P63208 P25963

P63261 P25963

Q00610 P25963

Q04206 P25963

Q04864 P25963

Q12923 P25963

Q13616 P25963

Q13748 P25963

Q15653 P25963

Q3ZCQ8 P25963

Q71U36 P25963

Q71UM5 P25963

Q8N668 P25963

Q96HA1 P25963

Q9H9T3 P25963

Q9UBC9 P25963

Q9UDV6 P25963

Q9UKB1 P25963

Q9UQC1 P25963

Q9Y297 P25963

Q9Y6K9 P25963

P05556 P26006

P30101 P26006

P13612 P26010

P21333 P26010

P23229 P26010

P26010 P26010

P38570 P26010

Q7Z6G8 P26010

Q9UBP9 P26010

P02751 P26038

P08567 P26038

P16104 P26038

P19320 P26038

P21127 P26038

P43405 P26038

P53779 P26038

Q14324 P26038

Q5S007 P26038

P04626 P26045

P08581 P26045

P11473 P26045

P31946 P26045

P31947 P26045

P61981 P26045

P63104 P26045

P78536 P26045

P02751 P26196

P19320 P26196

P41182 P26196

P46108 P26196

P62993 P26196

Q00005 P26196

Q8IZD4 P26196

Q8WWM7 P26196

Q96F86 P26196

Q99459 P26196

Q99700 P26196

Q9NPI6 P26196

Q9NPJ4 P26196

P00533 P26232

P46937 P26232

O75530 P26358

O95503 P26358

P10276 P26358

P14373 P26358

P19320 P26358

P48552 P26358

P67870 P26358

Q13547 P26358

Q15910 P26358

Q93009 P26358

Q96EB6 P26358

Q96T88 P26358

Q9UBC3 P26358

O94829 P26367

Q96F44 P26367

Q9NSC5 P26367

Q9NYR9 P26367

O43251 P26368

O43395 P26368

O75525 P26368

O95166 P26368

P03372 P26368

P09012 P26368

P15941 P26368

P19320 P26368

P22087 P26368

P52756 P26368

P54253 P26368

P60520 P26368

P61978 P26368

P78362 P26368

P98175 P26368

Q01081 P26368

Q13573 P26368

Q14103 P26368

Q14498 P26368

Q7Z7F0 P26368

Q7Z7J5 P26368

Q8IWX8 P26368

Q8IWZ8 P26368

Q8IX12 P26368

Q8N7W2 P26368

Q96LR2 P26368

Q99459 P26368

Q99590 P26368

Q9BWF3 P26368

Q9BZE0 P26368

Q9GZQ8 P26368

Q9H0R8 P26368

Q9NVV9 P26368

Q9UHX1 P26368

Q9UKJ5 P26368

P84022 P26368

Q15796 P26368

Q02930 P26371

Q6ZNA4 P26371

Q9HB63 P26371

Q9UBR2 P26371

O95166 P26373

P01106 P26373

P03372 P26373

P16333 P26373

P19174 P26373

P19320 P26373

P27986 P26373

P38919 P26373

P43146 P26373

P46781 P26373

P60520 P26373

P61326 P26373

P62993 P26373

Q13573 P26373

Q15843 P26373

Q99459 P26373

Q9H0R8 P26373

Q9H492 P26373

Q9UER7 P26373

Q9UL18 P26373

A8MW92 P26378

Q01844 P26378

O75716 P26441

P40189 P26441

P42702 P26441

P60228 P26441

P04271 P26447

P04637 P26447

P15514 P26447

P16104 P26447

P46379 P26447

Q00987 P26447

Q13432 P26447

Q99075 P26447

P02751 P26583

P17096 P26583

P19320 P26583

P19838 P26583

P48729 P26583

A0JLT2 P26599

O43251 P26599

O95166 P26599

O95758 P26599

P01106 P26599

P09012 P26599

P17096 P26599

P19320 P26599

P23246 P26599

P26599 P26599

P32121 P26599

P60520 P26599

P78362 P26599

P98175 P26599

Q13137 P26599

Q13573 P26599

Q5U5Q3 P26599

Q96PU8 P26599

Q99459 P26599

Q99729 P26599

Q9BTT4 P26599

Q9GZQ8 P26599

Q9H0R8 P26599

Q9H492 P26599

Q9NX70 P26599

P62993 P26599

O00443 P26639

O75323 P26639

P02751 P26639

P19320 P26639

P54619 P26639

Q15843 P26639

Q9BSB4 P26639

Q9BW61 P26639

Q9GZZ9 P26639

Q9UGJ0 P26639

O00555 P26640

P19320 P26640

Q09019 P26640

Q15759 P26640

Q8IY92 P26640

Q8WYK2 P26640

Q96G74 P26640

Q9BSB4 P26640

Q9GZQ8 P26640

O00193 P26641

O15084 P26641

O43261 P26641

O43809 P26641

O75956 P26641

O94762 P26641

O95166 P26641

O95257 P26641

O95989 P26641

P00533 P26641

P01106 P26641

P12757 P26641

P24534 P26641

P26641 P26641

P29692 P26641

P31749 P26641

P43351 P26641

P48039 P26641

P49841 P26641

P60520 P26641

P61956 P26641

P61981 P26641

P62993 P26641

Q00613 P26641

Q12905 P26641

Q13011 P26641

Q13573 P26641

Q16236 P26641

Q8IZ13 P26641

Q8N4N7 P26641

Q8WYK2 P26641

Q969Q1 P26641

Q9BYV6 P26641

Q9GZQ8 P26641

Q9H0R8 P26641

Q9H492 P26641

Q9HAU4 P26641

Q9HCK5 P26641

Q9P0W5 P26641

Q9Y3C7 P26641

Q15834 P26651

Q8IU60 P26651

Q96F86 P26651

P26678 P26678

Q09013 P26678

Q92997 P26678

Q29983 P26718

O15304 P26842

Q12933 P26842

Q96PU4 P26842

P46379 P26885

Q9NRR5 P26885

O60235 P26927

Q7L9L4 P26927

P06241 P26951

P06493 P26951

P19174 P26951

P30304 P26951

P62993 P26951

Q03113 P26951

Q15466 P26951

Q8TBB1 P26951

O15162 P26992

P00519 P26992

Q92624 P26992

Q96RY5 P26992

Q9BQ66 P26992

P05813 P26998

P26998 P26998

P53672 P26998

P02751 P27105

P19320 P27105

P38606 P27105

P40429 P27105

P55854 P27105

P63165 P27105

P29322 P27144

Q14145 P27144

Q96LK0 P27144

P25208 P27338

A6ND36 P27348

O00257 P27348

O00273 P27348

O14744 P27348

O15198 P27348

O15553 P27348

O43491 P27348

O60282 P27348

O75494 P27348

O94921 P27348

O95166 P27348

O96013 P27348

P00533 P27348

P01106 P27348

P02751 P27348

P04049 P27348

P04626 P27348

P04637 P27348

P07359 P27348

P10636 P27348

P15056 P27348

P19838 P27348

P22681 P27348

P23142 P27348

P23945 P27348

P26196 P27348

P27448 P27348

P30304 P27348

P32121 P27348

P33176 P27348

P35527 P27348

P40818 P27348

P42338 P27348

P46527 P27348

P46937 P27348

P49286 P27348

P49407 P27348

P49796 P27348

P49815 P27348

P56524 P27348

P61326 P27348

P62258 P27348

P62993 P27348

P63104 P27348

P78314 P27348

P84103 P27348

Q02241 P27348

Q04912 P27348

Q04917 P27348

Q07955 P27348

Q08AE8 P27348

Q13247 P27348

Q14678 P27348

Q14934 P27348

Q15276 P27348

Q16543 P27348

Q32P44 P27348

Q4FZB7 P27348

Q5PRF9 P27348

Q5S007 P27348

Q5U5Q3 P27348

Q6ICG6 P27348

Q6PJG9 P27348

Q6PKG0 P27348

Q6WCQ1 P27348

Q7Z401 P27348

Q86W92 P27348

Q8IUF1 P27348

Q8IV61 P27348

Q8IXP7 P27348

Q8WUI4 P27348

Q92934 P27348

Q96C45 P27348

Q96CV9 P27348

Q96F86 P27348

Q96T60 P27348

Q9BZ23 P27348

Q9H0B6 P27348

Q9NQU5 P27348

Q9NRI5 P27348

Q9NSK0 P27348

Q9NZT1 P27348

Q9NYF0 P27348

Q9P0K1 P27348

Q9P0K7 P27348

Q9P0V3 P27348

Q9P2K3 P27348

Q9UBF8 P27348

Q9UDY2 P27348

Q9UJM3 P27348

Q9UK53 P27348

Q9UQC2 P27348

Q9UQL6 P27348

Q9Y2D8 P27348

Q9Y2J2 P27348

Q9Y383 P27348

Q9Y4H2 P27348

Q9Y6J0 P27348

O60494 P27352

O75676 P27361

P07948 P27361

P08575 P27361

P21462 P27361

P23467 P27361

P26885 P27361

P28482 P27361

P28562 P27361

P53355 P27361

P67775 P27361

Q02750 P27361

Q04828 P27361

Q05923 P27361

Q12913 P27361

Q14160 P27361

Q15262 P27361

Q16539 P27361

Q8IYT8 P27361

Q9BVL2 P27361

Q9C0H2 P27361

Q9P2Y4 P27361

Q9UK32 P27361

Q9Y508 P27361

O14920 P27448

O75439 P27448

O95639 P27448

P01100 P27448

P02766 P27448

P05060 P27448

P27449 P27448

P31946 P27448

P31947 P27448

P61981 P27448

P63104 P27448

Q04917 P27448

Q7KZI7 P27448

Q8WXK3 P27448

Q92527 P27448

Q96AD5 P27448

Q96ER3 P27448

Q99959 P27448

Q9H270 P27448

Q9H8H0 P27448

Q9NPI8 P27448

Q9UK80 P27448

O00299 P27449

P62888 P27469

O94782 P27482

P31152 P27482

Q8IY92 P27482

Q9H0B3 P27482

Q9HD67 P27482

Q9Y4E8 P27482

O00626 P27487

O14625 P27487

O94907 P27487

P01275 P27487

P01282 P27487

P01286 P27487

P02778 P27487

P09681 P27487

P16860 P27487

P18509 P27487

P19875 P27487

P27487 P27487

P48061 P27487

P51671 P27487

O95777 P27540

P27540 P27540

P35869 P27540

Q13829 P27540

Q16665 P27540

Q99814 P27540

O95166 P27635

O95793 P27635

P03372 P27635

P07947 P27635

P12931 P27635

P19320 P27635

P38919 P27635

P60520 P27635

P61326 P27635

P62380 P27635

P62993 P27635

Q15750 P27635

Q16659 P27635

Q9UL18 P27635

O95967 P27658

P27658 P27658

Q53G59 P27658

Q9BQ66 P27658

Q9NRR5 P27658

P02751 P27694

P19320 P27694

P23025 P27694

P32121 P27694

P35244 P27694

P38398 P27694

P49450 P27694

P51587 P27694

P51858 P27694

P54132 P27694

Q8IW19 P27694

Q8TDY2 P27694

Q9BQS8 P27694

Q9BSB4 P27694

Q9H9A7 P27694

P17096 P27695

P18887 P27695

P78362 P27695

Q09472 P27695

Q96EB6 P27695

Q8TAA9 P27701

O14948 P27707

P27707 P27707

B2Y833 P27708

O95166 P27708

O95758 P27708

P19320 P27708

P29353 P27708

P46781 P27708

P60520 P27708

P62993 P27708

P63104 P27708

Q00005 P27708

Q00653 P27708

Q13418 P27708

Q15796 P27708

Q5U5Q3 P27708

Q6PJG9 P27708

Q96DB2 P27708

Q9GZQ8 P27708

Q9H0R8 P27708

Q9H3D4 P27708

Q9H492 P27708

O95166 P27797

P05067 P27797

P10415 P27797

P15336 P27797

P16104 P27797

P48039 P27797

P60520 P27797

P78504 P27797

Q02750 P27797

Q03518 P27797

Q12824 P27797

Q13087 P27797

Q5T3J3 P27797

Q9H0R8 P27797

P02751 P27816

P16333 P27816

P19320 P27816

P27986 P27816

P49841 P27816

Q8TDR0 P27816

Q8TDY2 P27816

Q9BQS8 P27816

Q9H492 P27816

O15264 P27824

O60831 P27824

P00533 P27824

P04626 P27824

P13569 P27824

P15336 P27824

P16615 P27824

P18124 P27824

P21860 P27824

P22303 P27824

P35610 P27824

P41143 P27824

P43307 P27824

P48039 P27824

P49286 P27824

P60520 P27824

P61619 P27824

P68400 P27824

P78504 P27824

Q6IAM1 P27824

Q8IVT5 P27824

Q92597 P27824

Q99758 P27824

Q9H0R8 P27824

Q9HBA0 P27824

Q9UQF2 P27824

Q9NPH3 P27930

A9UF07 P27986

O00329 P27986

O14490 P27986

O15056 P27986

O15117 P27986

O15357 P27986

O15360 P27986

O15455 P27986

O43150 P27986

O43281 P27986

O43493 P27986

O43561 P27986

O43918 P27986

O60603 P27986

O60721 P27986

O75167 P27986

O75534 P27986

O95886 P27986

P00519 P27986

P00533 P27986

P03372 P27986

P04626 P27986

P06127 P27986

P06213 P27986

P08069 P27986

P08631 P27986

P09564 P27986

P09619 P27986

P10301 P27986

P10721 P27986

P10747 P27986

P10912 P27986

P11362 P27986

P12931 P27986

P15170 P27986

P15311 P27986

P15509 P27986

P16410 P27986

P17948 P27986

P19174 P27986

P19793 P27986

P20231 P27986

P20273 P27986

P20810 P27986

P21145 P27986

P21333 P27986

P21860 P27986

P22681 P27986

P25963 P27986

P27986 P27986

P29350 P27986

P29353 P27986

P29376 P27986

P29692 P27986

P30086 P27986

P30260 P27986

P30530 P27986

P30874 P27986

P32927 P27986

P35568 P27986

P36888 P27986

P38398 P27986

P41970 P27986

P42336 P27986

P42858 P27986

P43405 P27986

P43626 P27986

P45983 P27986

P46108 P27986

P46109 P27986

P47928 P27986

P49023 P27986

P49916 P27986

P58753 P27986

P60953 P27986

P61981 P27986

P62993 P27986

P63104 P27986

Q07687 P27986

Q13087 P27986

Q13094 P27986

Q13111 P27986

Q13480 P27986

Q13746 P27986

Q14118 P27986

Q16627 P27986

Q8IY22 P27986

Q8IZP0 P27986

Q8NFW9 P27986

Q92558 P27986

Q96B97 P27986

Q96EB6 P27986

Q96EV8 P27986

Q96T58 P27986

Q9BYB0 P27986

Q9H1R2 P27986

Q9P1A6 P27986

Q9UQC2 P27986

Q9Y4H2 P27986

Q9Y534 P27986

Q9Y6X6 P27986

P61289 P27987

P01857 P28039

Q03518 P28062

Q03519 P28062

Q99436 P28062

P25787 P28062

Q03518 P28065

Q99436 P28065

Q9Y244 P28065

Q9Y3R0 P28065

Q9Y6Q9 P28065

P25787 P28065

O14818 P28066

P01106 P28066

P02751 P28066

P17861 P28066

P19320 P28066

P25786 P28066

P25787 P28066

P25789 P28066

P55036 P28066

P60900 P28066

Q14696 P28066

Q15051 P28066

Q9BQ83 P28066

Q9Y5K5 P28066

P04229 P28067

P13760 P28067

P04229 P28068

P13760 P28068

P28067 P28068

P28347 P28068

Q13618 P28068

O14818 P28070

P01106 P28070

P16104 P28070

P25786 P28070

P25787 P28070

P28074 P28070

P60900 P28070

Q8IY92 P28070

Q99436 P28070

Q9Y5K5 P28070

O14818 P28072

P19320 P28072

P25786 P28072

P25787 P28072

P60900 P28072

Q00987 P28072

Q15051 P28072

Q9Y244 P28072

Q9Y5K5 P28072

P01106 P28074

P02751 P28074

P08238 P28074

P25786 P28074

P25787 P28074

P27824 P28074

P35998 P28074

P49720 P28074

P54578 P28074

P60900 P28074

Q00532 P28074

Q15051 P28074

Q99436 P28074

Q9BQ83 P28074

Q9H0R8 P28074

Q9H492 P28074

Q9HC24 P28074

Q9Y244 P28074

Q9Y5K5 P28074

P08908 P28222

Q9Y2Q3 P28222

O75970 P28223

O75381 P28288

P25786 P28288

P28288 P28288

P33897 P28288

P40855 P28288

Q13286 P28288

Q13627 P28288

Q70EL3 P28288

Q8NFA0 P28288

Q9Y463 P28288

Q92905 P28289

O15265 P28290

P15336 P28290

P27348 P28290

P62258 P28290

Q07666 P28290

Q9Y383 P28290

O95967 P28300

P01106 P28300

P15502 P28300

P35555 P28300

Q15051 P28300

Q9UBX5 P28300

O75381 P28328

P40855 P28328

P49459 P28328

P63146 P28328

Q04724 P28328

Q9UKR5 P28328

O00716 P28331

O43678 P28331

O60344 P28331

P01106 P28331

P0C862 P28331

P19320 P28331

P49407 P28331

Q06187 P28331

Q14197 P28331

Q8NC60 P28331

Q96KP6 P28331

O75179 P28332

O76021 P28332

P01116 P28332

P49407 P28332

Q15025 P28332

Q8WWW0 P28332

O75970 P28335

O75419 P28340

P00519 P28340

P01106 P28340

P05060 P28340

P06241 P28340

P12004 P28340

P49005 P28340

Q13573 P28340

Q96S55 P28340

Q99459 P28340

Q9HCU8 P28340

Q9P2H0 P28340

O75600 P28347

P06733 P28347

P14859 P28347

P28347 P28347

P46937 P28347

P41161 P28356

Q5T7V8 P28356

O95758 P28360

Q9HAN9 P28360

O00716 P28370

P02686 P28482

P06239 P28482

P07948 P28482

P14921 P28482

P16989 P28482

P19320 P28482

P27361 P28482

P28562 P28482

P32121 P28482

P35236 P28482

P35813 P28482

P43146 P28482

P46109 P28482

P58546 P28482

P62136 P28482

P67775 P28482

P67809 P28482

P84022 P28482

Q02750 P28482

Q05923 P28482

Q12913 P28482

Q13115 P28482

Q14160 P28482

Q14558 P28482

Q14790 P28482

Q15025 P28482

Q15418 P28482

Q16539 P28482

Q16690 P28482

Q16828 P28482

Q16829 P28482

Q3T906 P28482

Q8N8S7 P28482

Q8NFZ5 P28482

Q99759 P28482

Q99956 P28482

Q9BYB0 P28482

Q9H6Z9 P28482

P27361 P28562

P28482 P28562

Q13309 P28562

Q16539 P28562

P17980 P28566

P43686 P28566

O15372 P28676

Q00534 P28698

O14810 P28702

O43791 P28702

O75347 P28702

P02686 P28702

P05455 P28702

P09496 P28702

P10276 P28702

P14625 P28702

P20962 P28702

P29122 P28702

Q00975 P28702

Q07890 P28702

Q14152 P28702

Q15042 P28702

Q8N5G2 P28702

Q8NI22 P28702

Q99459 P28702

Q99666 P28702

Q9NVR0 P28702

Q9P2W9 P28702

Q9UKG1 P28702

P41182 P28715

P50613 P28715

P02751 P28749

P11802 P28749

P22736 P28749

P24941 P28749

P41732 P28749

P62318 P28749

P67775 P28749

Q01094 P28749

Q06278 P28749

Q08379 P28749

Q13574 P28749

Q13627 P28749

Q14653 P28749

Q16254 P28749

Q16659 P28749

Q7L2E3 P28749

Q9BUH8 P28749

Q9Y463 P28749

Q9Y5X4 P28749

Q9Y605 P28749

O00459 P28799

O00555 P28799

O15198 P28799

O15265 P28799

O43741 P28799

P05783 P28799

P13569 P28799

P19438 P28799

P20333 P28799

P25490 P28799

P46109 P28799

P49639 P28799

P51959 P28799

P98160 P28799

Q15418 P28799

Q16526 P28799

Q92837 P28799

Q96AQ9 P28799

Q9NTG7 P28799

P28827 P28827

Q02742 P28838

Q9BW61 P28838

Q9UL33 P28838

P41182 P28908

P61769 P29016

P08571 P29033

P04637 P29034

P15336 P29034

P15514 P29034

P29034 P29034

P52292 P29034

Q00987 P29034

Q02790 P29034

O43149 P29074

P00519 P29074

P04183 P29074

P06858 P29074

P26641 P29074

P27986 P29074

P46108 P29074

P62993 P29074

P68104 P29074

Q92993 P29074

P32780 P29083

P78362 P29083

P29083 P29084

O00555 P29122

O00716 P29218

P29218 P29218

P29274 P29274

P30542 P29274

P43490 P29274

P62158 P29274

Q13107 P29274

Q7Z6G3 P29274

Q99418 P29274

O43399 P29275

Q14586 P29275

O95684 P29279

P02751 P29279

P21802 P29279

P22607 P29279

P48059 P29279

O00505 P29317

P00533 P29317

P08238 P29317

P20827 P29317

P25098 P29317

P28749 P29317

P29317 P29317

Q00537 P29317

Q06124 P29317

Q15375 P29317

Q9UK45 P29317

Q9UL46 P29317

P42771 P29320

Q15375 P29320

Q96H35 P29320

Q96T51 P29320

P62195 P29322

Q13283 P29322

Q6NUN9 P29322

Q6PI48 P29322

P28749 P29323

P49790 P29323

Q12974 P29323

Q16539 P29323

O95866 P29350

P00533 P29350

P03372 P29350

P04629 P29350

P06239 P29350

P08922 P29350

P09619 P29350

P11049 P29350

P16284 P29350

P19022 P29350

P19235 P29350

P20138 P29350

P20273 P29350

P27986 P29350

P31994 P29350

P32927 P29350

P43626 P29350

P43628 P29350

P78324 P29350

Q03135 P29350

Q0P6H9 P29350

Q14790 P29350

Q6GTX8 P29350

Q7Z6A9 P29350

Q8TDQ1 P29350

Q96LC7 P29350

Q9UKJ1 P29350

Q9Y6R4 P29350

P84022 P29350

A9UF07 P29353

O00193 P29353

O00459 P29353

O15357 P29353

P00533 P29353

P04626 P29353

P04629 P29353

P05067 P29353

P05783 P29353

P06213 P29353

P07766 P29353

P07949 P29353

P12314 P29353

P21860 P29353

P27986 P29353

P29353 P29353

P31994 P29353

P32927 P29353

P33151 P29353

P35968 P29353

P42229 P29353

P43403 P29353

P46108 P29353

P62993 P29353

P63104 P29353

Q07889 P29353

Q12774 P29353

Q13137 P29353

Q15303 P29353

Q8TF42 P29353

Q92835 P29353

Q96CW1 P29353

Q99459 P29353

Q9BPY3 P29353

Q9H840 P29353

Q9UJM3 P29353

Q9UHF0 P29371

O00264 P29372

O43809 P29372

P00505 P29372

P04179 P29372

P13804 P29372

P14174 P29372

P18077 P29372

P18887 P29372

P21266 P29372

P23142 P29372

P24752 P29372

P25786 P29372

P25787 P29372

P29372 P29372

P35998 P29372

P43487 P29372

P48047 P29372

P51149 P29372

P51665 P29372

P52907 P29372

P61604 P29372

P62273 P29372

P68402 P29372

Q01105 P29372

Q96AG4 P29372

Q9H8S9 P29372

Q9HB71 P29372

Q9UPN3 P29372

Q9Y237 P29372

Q9Y3B8 P29372

Q13547 P29374

Q9HCU9 P29374

P40227 P29375

Q13547 P29375

P20073 P29400

P38936 P29400

P55055 P29400

Q01955 P29400

Q15038 P29400

Q16637 P29400

Q92876 P29400

P01106 P29401

P02751 P29401

P16104 P29401

Q9H1Y0 P29401

Q9UKP3 P29401

P29460 P29459

P42701 P29459

P29459 P29460

P29460 P29460

P42701 P29460

Q9NPF7 P29460

P09038 P29466

Q9C000 P29466

Q9NPP4 P29466

P01583 P29466

Q8IVI9 P29474

Q08AM6 P29475

P60520 P29508

Q9H0R8 P29508

O95758 P29558

O14746 P29590

O15162 P29590

O95405 P29590

P00325 P29590

P01106 P29590

P04637 P29590

P06730 P29590

P19838 P29590

P25445 P29590

P25788 P29590

P29590 P29590

P55854 P29590

P60228 P29590

P63165 P29590

P81877 P29590

Q00987 P29590

Q05516 P29590

Q08999 P29590

Q13155 P29590

Q13164 P29590

Q13207 P29590

Q13472 P29590

Q13526 P29590

Q13618 P29590

Q15583 P29590

Q16254 P29590

Q5S007 P29590

Q5VVX9 P29590

Q8N726 P29590

Q8N9N5 P29590

Q96DB2 P29590

Q96EQ8 P29590

Q99961 P29590

Q9UBN7 P29590

Q9UER7 P29590

Q9UKL3 P29590

Q9Y2M5 P29590

P06493 P29590

Q00534 P29590

Q96EB6 P29590

P08238 P29597

P17181 P29597

P18031 P29597

O15264 P29692

O43719 P29692

O94886 P29692

O95352 P29692

O95433 P29692

P00441 P29692

P07814 P29692

P15336 P29692

P19320 P29692

P26641 P29692

P29692 P29692

P40337 P29692

P43351 P29692

P46778 P29692

Q08AD1 P29692

Q13011 P29692

Q14194 P29692

Q14204 P29692

Q15424 P29692

Q16659 P29692

Q5C9Z4 P29692

Q5T5U3 P29692

Q7KZ85 P29692

Q86WT1 P29692

Q8IX12 P29692

Q8WYK2 P29692

Q8WYQ5 P29692

Q99832 P29692

Q9BYT3 P29692

Q9GZQ8 P29692

Q9UKB3 P29692

Q9Y572 P29692

P01106 P29803

O15264 P29966

O95758 P29966

P15336 P29966

Q9UFF9 P29966

O43889 P29972

O95967 P29972

Q15654 P29972

Q15834 P29972

Q99750 P29972

Q9BQ66 P29972

P13569 P29992

P30038 P30038

P15336 P30040

P16104 P30040

Q9NRR5 P30040

O15264 P30041

P02751 P30041

P12004 P30041

P16104 P30041

P19320 P30041

P19652 P30041

P22736 P30041

P30041 P30041

P36969 P30041

P43351 P30041

P60520 P30041

Q9H0R8 P30041

Q9H492 P30041

P02763 P30043

P19320 P30043

P15336 P30044

Q14197 P30044

Q6MZP7 P30044

Q9GZT6 P30044

Q9NWV8 P30044

O95166 P30048

P02751 P30048

P15336 P30048

P16104 P30048

P19320 P30048

P30048 P30048

P60520 P30048

Q00005 P30048

Q13162 P30048

Q5S007 P30048

Q86UT6 P30048

Q8TAX7 P30048

Q92731 P30048

Q9GZQ8 P30048

Q9H0R8 P30048

Q9H492 P30048

P24539 P30049

Q9H845 P30049

O95166 P30050

P02751 P30050

P03372 P30050

P17020 P30050

P19320 P30050

P27824 P30050

P32121 P30050

P60520 P30050

P62993 P30050

P68366 P30050

Q13573 P30050

Q15843 P30050

Q5U5Q3 P30050

Q9GZQ8 P30050

Q9H0R8 P30050

Q9H3D4 P30050

Q9HCK5 P30050

Q9UKV8 P30050

Q9UL18 P30050

Q9UQ80 P30050

Q00005 P30050

P01009 P30084

P19532 P30084

P30084 P30084

P40763 P30084

Q8IUF1 P30084

Q9BW61 P30084

Q9HAU4 P30084

Q9H1Y0 P30085

Q9UKE5 P30085

O76061 P30086

P04049 P30086

P40337 P30086

P60520 P30086

Q15051 P30086

O15264 P30101

O15533 P30101

P05067 P30101

P07237 P30101

P07942 P30101

P08582 P30101

P11047 P30101

P12111 P30101

P13667 P30101

P15336 P30101

P17301 P30101

P27797 P30101

P27824 P30101

P60520 P30101

P78536 P30101

Q03518 P30101

Q13162 P30101

Q13586 P30101

Q13751 P30101

Q13753 P30101

Q86YB8 P30101

Q8N8Z6 P30101

Q969S8 P30101

Q96HE7 P30101

Q9H0R8 P30101

Q9UIW2 P30101

Q9Y4K0 P30101

O00506 P30153

O14503 P30153

O43815 P30153

O95166 P30153

O95376 P30153

O95405 P30153

P02751 P30153

P04626 P30153

P04637 P30153

P07900 P30153

P13569 P30153

P19320 P30153

P22392 P30153

P31749 P30153

P32121 P30153

P51449 P30153

P51959 P30153

P53041 P30153

P53816 P30153

P60510 P30153

P60520 P30153

P62714 P30153

P63151 P30153

P67775 P30153

P84022 P30153

Q00005 P30153

Q04206 P30153

Q13033 P30153

Q13155 P30153

Q13362 P30153

Q13469 P30153

Q13573 P30153

Q14738 P30153

Q15172 P30153

Q16659 P30153

Q5S007 P30153

Q5VSL9 P30153

Q66LE6 P30153

Q6PJG9 P30153

Q7L7W2 P30153

Q8TCG1 P30153

Q8WZ74 P30153

Q969G9 P30153

Q96L34 P30153

Q99459 P30153

Q9BRV8 P30153

Q9BUL8 P30153

Q9GZQ8 P30153

Q9H0R8 P30153

Q9H7D0 P30153

Q9NRL3 P30153

Q9P289 P30153

Q9P2B4 P30153

Q9Y228 P30153

Q9Y2T4 P30153

Q9Y3A3 P30153

Q9Y570 P30153

Q9Y6E0 P30153

O00506 P30154

O14503 P30154

P11233 P30154

P30153 P30154

P30154 P30154

P53355 P30154

P62714 P30154

P63151 P30154

P67775 P30154

Q00005 P30154

Q13033 P30154

Q13362 P30154

Q13573 P30154

Q14738 P30154

Q15172 P30154

Q16537 P30154

Q562F6 P30154

Q5VSL9 P30154

Q66LE6 P30154

Q70EK8 P30154

Q8IYT8 P30154

Q8WZ74 P30154

Q96KP6 P30154

Q9BRV8 P30154

Q9BUL8 P30154

Q9H0K1 P30154

Q9H7D0 P30154

Q9NRL3 P30154

Q9P2B4 P30154

Q9UGI0 P30154

Q9Y228 P30154

Q9Y2T4 P30154

Q9Y3A3 P30154

Q9Y6E0 P30154

P46976 P30203

O60566 P30260

P00519 P30260

P12830 P30260

P51784 P30260

P60484 P30260

Q12834 P30260

Q13042 P30260

Q13257 P30260

Q13573 P30260

Q8NHZ8 P30260

Q96C86 P30260

Q96DE5 P30260

Q99459 P30260

Q9BS18 P30260

Q9H1A4 P30260

Q9H2P0 P30260

Q9UI95 P30260

Q9UJX2 P30260

Q9UJX4 P30260

Q9UM11 P30260

Q06187 P30273

Q9HCN6 P30273

P11802 P30279

P24941 P30279

P35227 P30279

P38936 P30279

P46527 P30279

Q00534 P30279

Q00535 P30279

O94921 P30281

O95967 P30281

P02774 P30281

P10242 P30281

P11473 P30281

P11802 P30281

P21127 P30281

P25445 P30281

P38936 P30281

P46527 P30281

P62140 P30281

Q00534 P30281

Q00535 P30281

Q96EV8 P30281

Q9H063 P30281

Q9UBQ5 P30281

P31946 P30291

P32121 P30291

P53350 P30291

P61981 P30291

P63104 P30291

Q9HC98 P30291

Q9UKB1 P30291

Q9Y297 P30291

O14757 P30304

P04049 P30304

P11532 P30304

P14635 P30304

P25791 P30304

P30508 P30304

P31946 P30304

P35711 P30304

P41227 P30304

P62258 P30304

P63104 P30304

P68104 P30304

Q13126 P30304

Q13202 P30304

Q14094 P30304

Q8IU85 P30304

Q96KG9 P30304

Q96PV7 P30304

Q9BV40 P30304

Q9Y297 P30304

P24941 P30305

P31946 P30305

P62258 P30305

P63104 P30305

Q04917 P30305

Q15323 P30305

Q92608 P30305

Q92764 P30305

Q99623 P30305

Q9UKB1 P30305

O14757 P30307

P53350 P30307

P04637 P30405

P15336 P30405

P31689 P30408

P68366 P30408

Q08345 P30408

P01042 P30411

P32121 P30414

P30447 P30450

Q9NYU2 P30450

Q9Y4L1 P30450

O95347 P30457

P04439 P30457

P16188 P30457

P17693 P30457

P30455 P30457

P30464 P30457

P30481 P30457

P51571 P30457

Q07000 P30457

Q5XKP0 P30457

Q860B5 P30457

Q93084 P30457

Q9BUP0 P30457

Q9NYU1 P30457

Q9NYU2 P30457

Q9UEX5 P30457

Q9Y4L1 P30457

O00193 P30480

O00303 P30480

O00442 P30480

O00505 P30480

O14737 P30480

O14818 P30480

O15067 P30480

O15144 P30480

O15372 P30480

O43242 P30480

O43488 P30480

O43765 P30480

O60232 P30480

O60493 P30480

O60506 P30480

O75390 P30480

O75531 P30480

O75534 P30480

O75694 P30480

O75964 P30480

O76094 P30480

O95347 P30480

O95433 P30480

O95757 P30480

O95831 P30480

O96008 P30480

P00403 P30480

P00491 P30480

P00492 P30480

P00505 P30480

P02771 P30480

P02786 P30480

P04080 P30480

P04179 P30480

P04181 P30480

P04843 P30480

P06748 P30480

P06753 P30480

P07355 P30480

P08579 P30480

P09661 P30480

P09960 P30480

P10599 P30480

P10768 P30480

P11586 P30480

P11940 P30480

P12004 P30480

P12532 P30480

P13667 P30480

P13693 P30480

P13797 P30480

P13798 P30480

P14174 P30480

P15104 P30480

P15121 P30480

P15170 P30480

P15311 P30480

P16152 P30480

P16949 P30480

P17174 P30480

P17812 P30480

P17858 P30480

P20290 P30480

P20340 P30480

P20618 P30480

P20700 P30480

P21796 P30480

P22061 P30480

P22234 P30480

P22695 P30480

P23284 P30480

P23526 P30480

P23921 P30480

P24534 P30480

P24752 P30480

P25205 P30480

P25786 P30480

P26038 P30480

P26639 P30480

P26640 P30480

P27695 P30480

P28070 P30480

P28072 P30480

P28331 P30480

P28482 P30480

P29144 P30480

P29401 P30480

P29692 P30480

P30040 P30480

P30046 P30480

P30084 P30480

P30086 P30480

P30520 P30480

P31040 P30480

P31150 P30480

P31689 P30480

P31949 P30480

P33993 P30480

P34897 P30480

P35080 P30480

P35244 P30480

P35637 P30480

P37108 P30480

P37802 P30480

P38606 P30480

P39656 P30480

P40616 P30480

P40939 P30480

P41091 P30480

P41250 P30480

P42704 P30480

P42771 P30480

P43307 P30480

P43487 P30480

P46108 P30480

P46781 P30480

P47914 P30480

P48147 P30480

P49189 P30480

P49321 P30480

P49411 P30480

P49720 P30480

P49773 P30480

P49915 P30480

P51148 P30480

P51149 P30480

P51452 P30480

P51572 P30480

P52209 P30480

P52272 P30480

P52907 P30480

P53007 P30480

P54136 P30480

P55036 P30480

P55072 P30480

P55735 P30480

P55786 P30480

P56192 P30480

P58546 P30480

P60033 P30480

P60660 P30480

P60900 P30480

P61019 P30480

P61077 P30480

P61086 P30480

P61106 P30480

P61160 P30480

P61163 P30480

P61289 P30480

P61513 P30480

P61586 P30480

P61604 P30480

P61803 P30480

P62158 P30480

P62191 P30480

P62263 P30480

P62330 P30480

P62333 P30480

P62714 P30480

P62750 P30480

P62910 P30480

P63167 P30480

P63173 P30480

P67812 P30480

P69905 P30480

P83731 P30480

P99999 P30480

Q00688 P30480

Q01105 P30480

Q01813 P30480

Q02750 P30480

Q06210 P30480

Q08211 P30480

Q09028 P30480

Q12904 P30480

Q12931 P30480

Q13148 P30480

Q13200 P30480

Q13263 P30480

Q13347 P30480

Q13642 P30480

Q14166 P30480

Q14444 P30480

Q14566 P30480

Q14657 P30480

Q14697 P30480

Q15008 P30480

Q15019 P30480

Q15185 P30480

Q15365 P30480

Q15436 P30480

Q15631 P30480

Q15643 P30480

Q15691 P30480

Q15758 P30480

Q15813 P30480

Q16630 P30480

Q16658 P30480

Q16740 P30480

Q3ZAQ7 P30480

Q5SQT9 P30480

Q5T081 P30480

Q6FGD7 P30480

Q6FGH5 P30480

Q6FHQ0 P30480

Q6FHT8 P30480

Q6PKD3 P30480

Q75MH1 P30480

Q7KZF4 P30480

Q86SX6 P30480

Q86YI5 P30480

Q8NC51 P30480

Q8NFH3 P30480

Q8TAF3 P30480

Q8WUM4 P30480

Q96AG4 P30480

Q96CX2 P30480

Q96QK1 P30480

Q99460 P30480

Q9BRA2 P30480

Q9BWD1 P30480

Q9BXJ9 P30480

Q9BZK3 P30480

Q9GZT3 P30480

Q9H0U4 P30480

Q9H773 P30480

Q9H9A6 P30480

Q9H9B4 P30480

Q9HAV7 P30480

Q9HB71 P30480

Q9NP97 P30480

Q9NPD3 P30480

Q9NQ50 P30480

Q9NR28 P30480

Q9NR30 P30480

Q9NRN7 P30480

Q9NSE4 P30480

Q9NZ23 P30480

Q9NZI8 P30480

Q9P0L0 P30480

Q9P258 P30480

Q9UBT2 P30480

Q9UGP8 P30480

Q9UHB9 P30480

Q9UI30 P30480

Q9UKK9 P30480

Q9UL25 P30480

Q9ULC4 P30480

Q9UNM6 P30480

Q9UQ80 P30480

Q9UQE7 P30480

Q9Y230 P30480

Q9Y265 P30480

Q9Y266 P30480

Q9Y277 P30480

Q9Y295 P30480

Q9Y3A5 P30480

Q9Y3F4 P30480

Q9Y5B9 P30480

Q9Y5L4 P30480

Q9Y678 P30480

P21860 P30481

P21860 P30483

O43809 P30504

O60613 P30504

P00505 P30504

P01889 P30504

P09960 P30504

P12004 P30504

P12268 P30504

P16152 P30504

P17812 P30504

P21266 P30504

P24752 P30504

P25205 P30504

P25787 P30504

P26038 P30504

P28070 P30504

P30460 P30504

P49411 P30504

P49591 P30504

P49915 P30504

P60900 P30504

P61289 P30504

P62158 P30504

Q00610 P30504

Q13526 P30504

Q14697 P30504

Q15008 P30504

Q15365 P30504

Q15691 P30504

Q63HN8 P30504

Q6F3D7 P30504

Q6FGD7 P30504

Q8IUC4 P30504

Q99873 P30504

Q9MY50 P30504

Q9NZ23 P30504

Q9NYU2 P30504

Q9Y262 P30504

Q9Y266 P30504

P21860 P30512

O15031 P30519

O94966 P30519

P01023 P30519

P04150 P30519

P26641 P30519

P29353 P30519

P54257 P30519

Q03701 P30519

Q15047 P30519

Q5UIP0 P30519

Q6IE81 P30519

Q7L5N1 P30519

Q8NHY6 P30519

Q8TB96 P30519

Q96AW1 P30519

Q99613 P30519

Q9P2H0 P30519

Q9UNH6 P30519

Q9Y2X7 P30519

Q9Y3C7 P30519

Q9Y5X3 P30519

Q8NB12 P30520

P08238 P30530

P27986 P30530

P62993 P30530

Q14393 P30530

P78362 P30532

O15355 P30533

P49792 P30533

P55854 P30533

Q14181 P30533

Q9NQC3 P30533

P29274 P30542

Q96H20 P30542

P01019 P30556

P19174 P30556

P49407 P30556

P50148 P30556

Q13107 P30566

Q9BW61 P30566

Q9Y4E8 P30566

Q5W0Q7 P30613

Q6UXB4 P30613

Q96G74 P30613

Q9Y6I4 P30613

O14777 P30622

P04150 P30622

P53350 P30622

P68366 P30622

Q15691 P30622

Q8N3C7 P30622

O95758 P30626

P01111 P30626

P02751 P30626

P19320 P30626

P30626 P30626

P40763 P30626

P49810 P30626

P78362 P30626

Q13137 P30626

Q15796 P30626

P00533 P30711

O95433 P30793

P11142 P30793

P30047 P30793

P30793 P30793

P63104 P30793

Q02447 P30793

Q14161 P30793

Q96SU4 P30793

Q99962 P30793

P01106 P30837

P02751 P30837

P12104 P30837

Q14197 P30837

Q9GZQ8 P30837

Q9H492 P30837

P11831 P30838

Q6ZMK1 P30838

P27986 P30874

P32745 P30874

A0JLT2 P30876

O95402 P30876

P01106 P30876

Q13418 P30876

Q8IZL9 P30876

Q96DB2 P30876

Q9BTT4 P30876

Q9NWA0 P30876

Q9NX70 P30876

Q9Y5B0 P30876

P62158 P30988

Q5JY77 P30988

P30989 P30990

O95166 P31040

P01106 P31040

P19320 P31040

P21912 P31040

P32121 P31040

P60520 P31040

Q99643 P31040

Q9H0R8 P31040

Q9H492 P31040

O15160 P31146

P25791 P31146

P40763 P31146

P84022 P31146

Q13643 P31146

Q15750 P31146

Q8TB22 P31146

Q96GM5 P31146

P04183 P31150

P20338 P31150

P36915 P31150

P63104 P31150

Q15051 P31150

Q969Q5 P31150

Q9H492 P31150

P03372 P31151

P09914 P31151

P13569 P31151

P19320 P31151

Q00005 P31151

Q5TKA1 P31151

Q96S59 P31151

P08238 P31152

P46108 P31152

Q9H492 P31153

P01106 P31260

Q06124 P31260

P50542 P31268

O15162 P31269

O95758 P31269

Q13547 P31269

Q15654 P31269

Q93062 P31269

O95758 P31273

P00519 P31273

P06241 P31273

P12931 P31273

P16333 P31273

P62993 P31273

O95758 P31274

Q8IY92 P31274

P32519 P31276

O00470 P31314

P14921 P31314

P40424 P31314

P40426 P31314

P00533 P31321

P01106 P31321

P17612 P31321

P22694 P31321

P17612 P31323

P19438 P31323

P22694 P31323

Q9Y6D5 P31323

P16104 P31327

P63104 P31327

P23921 P31350

P41002 P31350

Q9UM11 P31350

O14908 P31431

P17252 P31431

O95166 P31483

O95758 P31483

P19320 P31483

P46781 P31483

P50616 P31483

P60520 P31483

Q7KZF4 P31483

Q9GZQ8 P31483

Q9H0R8 P31483

Q9H492 P31483

Q9HBD1 P31483

P61981 P31629

P62258 P31629

Q9H4A3 P31629

Q9UKB1 P31629

Q9Y297 P31629

Q13491 P31645

Q9NRD5 P31645

A0JLT2 P31689

O95166 P31689

O95989 P31689

P00533 P31689

P04626 P31689

P07900 P31689

P13569 P31689

P15822 P31689

P21860 P31689

P51636 P31689

P60520 P31689

Q13573 P31689

Q15051 P31689

Q92769 P31689

Q969S8 P31689

Q96DB2 P31689

Q99459 P31689

Q9GZX7 P31689

Q9H492 P31689

Q9H9G7 P31689

Q9HCK5 P31689

Q9NX70 P31689

Q9UBN7 P31689

O14746 P31749

O15530 P31749

O60437 P31749

O94875 P31749

O95999 P31749

P02749 P31749

P04049 P31749

P07738 P31749

P08670 P31749

P10636 P31749

P19174 P31749

P23443 P31749

P25685 P31749

P29474 P31749

P31749 P31749

P31947 P31749

P32927 P31749

P42345 P31749

P42574 P31749

P46527 P31749

P49841 P31749

P62136 P31749

P62140 P31749

P67775 P31749

Q00987 P31749

Q05513 P31749

Q13043 P31749

Q13485 P31749

Q13772 P31749

Q15047 P31749

Q16539 P31749

Q16543 P31749

Q1W6H9 P31749

Q5S007 P31749

Q5T1C6 P31749

Q7Z6J0 P31749

Q8TCG2 P31749

Q92547 P31749

Q92793 P31749

Q96EB6 P31749

Q96S96 P31749

Q99683 P31749

Q9BZQ8 P31749

Q9NPC1 P31749

Q9NUC0 P31749

O94875 P31751

P02647 P31751

P08238 P31751

P08670 P31751

P42574 P31751

P43490 P31751

Q16543 P31751

Q16627 P31751

Q7Z6J0 P31751

P13232 P31785

P24394 P31785

P62993 P31785

P08574 P31930

P15336 P31930

P22695 P31930

P31689 P31930

P62306 P31930

Q14197 P31930

Q56A76 P31930

Q9Y230 P31930

Q9Y5M8 P31930

O14979 P31939

P00533 P31939

P09012 P31939

P27105 P31939

Q96DG6 P31939

Q96JH8 P31939

Q9Y375 P31939

O95166 P31942

P02766 P31942

P09651 P31942

P16104 P31942

P19320 P31942

P26641 P31942

P31942 P31942

P50995 P31942

P52597 P31942

P60520 P31942

P62701 P31942

Q13151 P31942

Q14194 P31942

Q16637 P31942

Q8IWX8 P31942

Q92993 P31942

Q99616 P31942

Q9BVJ6 P31942

Q9GZQ8 P31942

Q9H0R8 P31942

Q9H492 P31942

Q9P1W9 P31942

Q9UBW7 P31942

Q9UKR5 P31942

O75674 P31943

O95166 P31943

O95229 P31943

O95758 P31943

P00533 P31943

P09651 P31943

P15336 P31943

P17844 P31943

P19320 P31943

P31942 P31943

P32121 P31943

P38919 P31943

P49407 P31943

P50616 P31943

P60520 P31943

P60709 P31943

P61326 P31943

P62993 P31943

Q00005 P31943

Q13573 P31943

Q14194 P31943

Q15427 P31943

Q15637 P31943

Q16637 P31943

Q92597 P31943

Q92879 P31943

Q99459 P31943

Q9GZQ8 P31943

Q9H0R8 P31943

Q9H492 P31943

Q9NR56 P31943

P13569 P31944

Q70EK8 P31944

Q96FW1 P31944

Q9Y478 P31944

O00257 P31946

O00273 P31946

O00422 P31946

O00459 P31946

O14649 P31946

O14744 P31946

O15264 P31946

O43143 P31946

O43299 P31946

O43491 P31946

O43524 P31946

O75494 P31946

O75592 P31946

O75643 P31946

O94915 P31946

O94921 P31946

O96013 P31946

P00519 P31946

P01106 P31946

P02751 P31946

P04049 P31946

P06213 P31946

P07437 P31946

P08069 P31946

P08238 P31946

P0CG48 P31946

P11021 P31946

P15056 P31946

P15336 P31946

P16104 P31946

P17812 P31946

P19320 P31946

P22681 P31946

P25705 P31946

P26045 P31946

P27448 P31946

P30291 P31946

P30304 P31946

P30305 P31946

P32121 P31946

P33176 P31946

P40818 P31946

P46937 P31946

P49407 P31946

P49796 P31946

P49815 P31946

P55040 P31946

P56524 P31946

P62258 P31946

P62993 P31946

P63104 P31946

P68400 P31946

P78362 P31946

P84103 P31946

Q02241 P31946

Q02750 P31946

Q04917 P31946

Q07021 P31946

Q07866 P31946

Q12802 P31946

Q13164 P31946

Q13627 P31946

Q14247 P31946

Q14678 P31946

Q14739 P31946

Q14C86 P31946

Q15029 P31946

Q15051 P31946

Q15287 P31946

Q15569 P31946

Q53ET0 P31946

Q5PRF9 P31946

Q5S007 P31946

Q5TCX8 P31946

Q6GQQ9 P31946

Q6ICG6 P31946

Q6PJG9 P31946

Q6PKG0 P31946

Q6R327 P31946

Q6WCQ1 P31946

Q70EL4 P31946

Q7KZI7 P31946

Q7L8J4 P31946

Q7Z401 P31946

Q86W92 P31946

Q86X27 P31946

Q86X29 P31946

Q8IXP7 P31946

Q8N1I0 P31946

Q8N3F8 P31946

Q8NFA0 P31946

Q8NFH8 P31946

Q8TBZ3 P31946

Q8TEW0 P31946

Q8WUI4 P31946

Q8WYL5 P31946

Q92538 P31946

Q92574 P31946

Q96B36 P31946

Q96CV9 P31946

Q96F86 P31946

Q96JH8 P31946

Q96KP6 P31946

Q96NE9 P31946

Q96PX8 P31946

Q96Q42 P31946

Q96Q89 P31946

Q96SB4 P31946

Q96TC7 P31946

Q99570 P31946

Q99683 P31946

Q9BRS2 P31946

Q9BZJ0 P31946

Q9H0H5 P31946

Q9H0R8 P31946

Q9H1K1 P31946

Q9H4L5 P31946

Q9H8N7 P31946

Q9HCG8 P31946

Q9NSK0 P31946

Q9NYF0 P31946

Q9P0K1 P31946

Q9P0K7 P31946

Q9P0V3 P31946

Q9P244 P31946

Q9P2M7 P31946

Q9UBF8 P31946

Q9UDY2 P31946

Q9UJ41 P31946

Q9UK53 P31946

Q9UKV3 P31946

Q9ULT8 P31946

Q9UQ35 P31946

Q9UQC2 P31946

Q9UQL6 P31946

Q9Y2A7 P31946

Q9Y2H1 P31946

Q9Y2J2 P31946

Q9Y2U5 P31946

Q9Y4H2 P31946

Q9Y6M7 P31946

O00257 P31947

O00444 P31947

O00515 P31947

O15151 P31947

O43916 P31947

O60269 P31947

O60292 P31947

O75592 P31947

O94875 P31947

O95171 P31947

P00519 P31947

P00533 P31947

P03372 P31947

P04049 P31947

P04150 P31947

P04637 P31947

P06493 P31947

P10398 P31947

P13861 P31947

P15144 P31947

P15336 P31947

P27448 P31947

P30291 P31947

P30305 P31947

P31947 P31947

P40818 P31947

P56524 P31947

P61587 P31947

P61962 P31947

P63104 P31947

P68400 P31947

P78406 P31947

P78524 P31947

Q00839 P31947

Q02241 P31947

Q07866 P31947

Q12788 P31947

Q13627 P31947

Q14103 P31947

Q14153 P31947

Q14451 P31947

Q14966 P31947

Q16890 P31947

Q3KP66 P31947

Q5VV41 P31947

Q6N089 P31947

Q6NUK4 P31947

Q6P4F7 P31947

Q6ZU52 P31947

Q70EL4 P31947

Q71H61 P31947

Q7KZI7 P31947

Q7L5N1 P31947

Q86UW9 P31947

Q86VP3 P31947

Q86W92 P31947

Q86YV5 P31947

Q8IZW8 P31947

Q8N236 P31947

Q8NEM2 P31947

Q8NFH8 P31947

Q8NHY2 P31947

Q8TEW0 P31947

Q8WU02 P31947

Q8WUI4 P31947

Q92625 P31947

Q92934 P31947

Q96ES7 P31947

Q96F86 P31947

Q96HA8 P31947

Q96IF1 P31947

Q96PE2 P31947

Q96PU5 P31947

Q99418 P31947

Q99959 P31947

Q9H0R8 P31947

Q9H1K1 P31947

Q9H2C0 P31947

Q9H6H4 P31947

Q9H8W4 P31947

Q9NS23 P31947

Q9NYF0 P31947

Q9NYL2 P31947

Q9P0V3 P31947

Q9UBF8 P31947

Q9UJM3 P31947

Q9UQC2 P31947

Q9UQL6 P31947

Q9Y2I8 P31947

Q9Y2U9 P31947

Q9Y446 P31947

Q9Y4H2 P31947

A0AUL9 P31948

O15264 P31948

O95758 P31948

P00533 P31948

P02751 P31948

P07900 P31948

P08107 P31948

P15336 P31948

P19320 P31948

P25791 P31948

P41279 P31948

P50750 P31948

P53041 P31948

P60520 P31948

Q00526 P31948

Q00535 P31948

Q70CQ1 P31948

Q96PU5 P31948

Q9Y4P8 P31948

P00533 P31949

P04271 P31949

P15336 P31949

P31949 P31949

P38606 P31949

Q9H0R8 P31949

P01857 P31994

P12931 P31994

P16333 P31994

P19174 P31994

P29350 P31994

Q06124 P31994

Q92835 P31994

Q9UKR5 P31994

P02741 P31995

P12931 P31995

P16333 P31995

P19174 P31995

P46108 P31995

P13688 P31997

P40199 P31997

Q15121 P32004

O14607 P32119

O95166 P32119

P19320 P32119

P30153 P32119

P30154 P32119

P43351 P32119

P60520 P32119

Q13325 P32119

Q16543 P32119

Q92934 P32119

Q9BQ95 P32119

Q9GZQ8 P32119

Q9H0R8 P32119

Q9H492 P32119

Q9Y2H9 P32119

O00203 P32121

O00505 P32121

O00541 P32121

O00629 P32121

O14964 P32121

O75251 P32121

O75688 P32121

O76061 P32121

O95218 P32121

P06396 P32121

P07550 P32121

P17813 P32121

P22087 P32121

P23528 P32121

P27348 P32121

P32121 P32121

P35813 P32121

P45984 P32121

P49407 P32121

P51148 P32121

P62158 P32121

Q00987 P32121

Q08499 P32121

Q13428 P32121

Q13523 P32121

Q14137 P32121

Q14978 P32121

Q15208 P32121

Q6DKI1 P32121

Q8N983 P32121

Q8TAQ2 P32121

Q96G25 P32121

Q96GQ7 P32121

Q9H422 P32121

Q9H9J2 P32121

Q9HBX9 P32121

O14841 P32189

O94829 P32189

P02743 P32189

P08684 P32189

P19793 P32189

P22736 P32189

Q06278 P32189

Q96SB3 P32238

P00519 P32239

P06307 P32239

P16333 P32239

P62993 P32239

P03989 P32241

P62158 P32241

O76081 P32242

Q6UY14 P32242

Q93062 P32242

Q99750 P32242

Q9BQ66 P32242

Q9C005 P32242

Q9UKJ5 P32242

P11802 P32243

P54253 P32243

Q9NPH3 P32245

O43889 P32246

Q04941 P32246

Q13330 P32249

P04899 P32302

P09471 P32302

P32321 P32321

Q6ZVK8 P32321

Q8TAP6 P32321

Q8TBB1 P32321

Q9GZT8 P32321

P19320 P32322

P32322 P32322

Q7Z465 P32322

Q9P0W5 P32418

P32455 P32455

O60238 P32456

P20073 P32456

P24522 P32456

P49841 P32456

P67870 P32456

P08047 P32519

P31276 P32519

P41182 P32519

O75970 P32745

P30874 P32745

P52758 P32754

O00505 P32780

P04637 P32780

P12814 P32780

P19447 P32780

P29083 P32780

P50613 P32780

P51946 P32780

Q15025 P32780

Q9H7U1 P32780

O60674 P32927

P04141 P32927

P05113 P32927

P05556 P32927

P08700 P32927

P29350 P32927

P29353 P32927

P63104 P32927

P32929 P32929

Q9H190 P32929

P20701 P32942

Q05516 P32942

O95166 P32969

O95758 P32969

O96007 P32969

P03372 P32969

P19320 P32969

P23396 P32969

P60520 P32969

Q15843 P32969

Q9H0R8 P32969

Q9NXU5 P32969

P98168 P33076

Q2QGD7 P33076

P35221 P33151

P35222 P33151

P25054 P33176

P31946 P33176

P31947 P33176

P61457 P33176

P63104 P33176

Q04917 P33176

Q15796 P33176

Q5W0Q7 P33176

Q7KZI7 P33176

Q8N0X7 P33176

Q99459 P33176

Q9BQS8 P33176

Q9P2Y5 P33176

Q9Y4G8 P33176

O95163 P33240

O95453 P33240

P26641 P33240

P38398 P33240

P46379 P33240

P53999 P33240

Q12873 P33240

Q13011 P33240

Q16891 P33240

Q92993 P33240

Q99689 P33240

Q99728 P33240

Q9H6E5 P33240

Q9NRR5 P33240

Q9P2H0 P33240

Q9P2I0 P33240

Q9UKF6 P33240

Q9UKR5 P33240

Q9UMX0 P33240

P01023 P33260

P34741 P33260

Q9BQ95 P33260

O95166 P33316

P60520 P33316

P62745 P33316

P62993 P33316

Q6ZVK8 P33316

Q9H0R8 P33316

Q9UM82 P33316

Q13114 P33527

Q86WV6 P33527

P06493 P33552

P24941 P33552

P62993 P33552

P68104 P33552

Q13432 P33552

Q9H2G9 P33552

Q9Y3Q8 P33552

P08138 P33681

P16410 P33681

Q9NZQ7 P33681

O43918 P33778

O95503 P33778

P83916 P33778

Q9HC52 P33778

P28288 P33897

P33897 P33897

P40855 P33897

P04626 P33947

Q9UGN5 P33947

O95359 P33981

P07437 P33981

P33981 P33981

Q13573 P33981

P01106 P33991

P19320 P33991

P27695 P33991

P33993 P33991

P49736 P33991

P62805 P33991

P78396 P33991

Q08945 P33991

Q09472 P33991

Q12905 P33991

Q14566 P33991

Q15843 P33991

Q8NCN4 P33991

Q93009 P33991

Q96HA7 P33991

Q99459 P33991

Q9BTE3 P33991

Q9Y5B9 P33991

O95989 P33992

P00533 P33992

P19320 P33992

P23508 P33992

P25054 P33992

P25205 P33992

P27695 P33992

P33993 P33992

P49736 P33992

P62805 P33992

Q00005 P33992

Q08945 P33992

Q09472 P33992

Q12905 P33992

Q12906 P33992

Q14566 P33992

Q15843 P33992

Q92878 P33992

Q93009 P33992

Q9BTE3 P33992

Q9H0R8 P33992

P00533 P33993

P01106 P33993

P07948 P33993

P0CG47 P33993

P19320 P33993

P27695 P33993

P33991 P33993

P33992 P33993

P33993 P33993

P46527 P33993

P49736 P33993

P49918 P33993

P53350 P33993

P62136 P33993

P62805 P33993

Q08945 P33993

Q12905 P33993

Q14566 P33993

Q14683 P33993

Q8N163 P33993

Q8NE63 P33993

Q99741 P33993

Q9BTE3 P33993

Q9H765 P33993

Q9NS73 P33993

Q9UBD5 P33993

Q9UFF9 P33993

Q9UL03 P33993

Q9Y5S9 P33993

O00299 P34130

O00560 P34741

P10242 P34741

P19784 P34741

P21359 P34741

P34741 P34741

P48454 P34741

Q12967 P34810

P00519 P34820

P06241 P34820

Q8TEU8 P34820

Q96NZ8 P34820

O43261 P34896

O75618 P34896

P11309 P34896

Q9GZQ8 P34896

O60739 P34897

P15336 P34897

P46736 P34897

P62993 P34897

Q12905 P34897

Q14197 P34897

Q7L0Y3 P34897

Q92597 P34897

Q9BW61 P34897

Q9GZZ9 P34897

Q9NWV8 P34897

Q9NXR7 P34897

P27986 P34903

Q14764 P34913

P13569 P34925

A0JLT2 P34931

O95166 P34931

O95402 P34931

P00533 P34931

P03372 P34931

P04637 P34931

P13569 P34931

P16104 P34931

P32121 P34931

P60520 P34931

P62993 P34931

P63104 P34931

P78380 P34931

Q8IZD4 P34931

Q8TDR0 P34931

Q92905 P34931

Q96EB6 P34931

Q9BTT4 P34931

Q9GZQ8 P34931

Q9H0R8 P34931

Q9H492 P34931

Q9NTG7 P34931

Q9NV56 P34931

Q9NWA0 P34931

Q9NX70 P34931

O95166 P34932

P07900 P34932

P42771 P34932

P60520 P34932

Q6P1J9 P34932

Q9GZQ8 P34932

Q9H0R8 P34932

Q9H492 P34932

P49407 P34947

Q96D09 P34969

P01876 P35030

P02768 P35030

P29353 P35030

P26447 P35070

P42858 P35080

P60520 P35080

Q9H0R8 P35080

Q8NFA0 P35125

P10916 P35125

P35372 P35212

P41145 P35212

O75427 P35219

O00716 P35221

O95758 P35221

P00533 P35221

P12830 P35221

P14923 P35221

P32121 P35221

P33993 P35221

P35222 P35221

P46937 P35221

P55196 P35221

Q13573 P35221

Q5W0B1 P35221

Q8N5A5 P35221

Q99459 P35221

A1Z199 P35222

O00512 P35222

O00716 P35222

O14617 P35222

O15169 P35222

O75564 P35222

O75581 P35222

O75937 P35222

O95405 P35222

O95758 P35222

P00519 P35222

P00533 P35222

P02511 P35222

P08069 P35222

P08575 P35222

P10275 P35222

P11388 P35222

P12830 P35222

P15336 P35222

P15884 P35222

P16284 P35222

P17948 P35222

P18146 P35222

P19022 P35222

P19838 P35222

P23470 P35222

P25054 P35222

P33151 P35222

P33993 P35222

P35813 P35222

P46778 P35222

P46937 P35222

P46940 P35222

P49023 P35222

P49768 P35222

P49841 P35222

P50402 P35222

Q02952 P35222

Q03431 P35222

Q04206 P35222

Q08050 P35222

Q13308 P35222

Q13547 P35222

Q13761 P35222

Q14526 P35222

Q14678 P35222

Q15596 P35222

Q16827 P35222

Q2LD37 P35222

Q6P1J9 P35222

Q7Z6J6 P35222

Q8TAM6 P35222

Q8WVC0 P35222

Q92597 P35222

Q92731 P35222

Q92793 P35222

Q96AC1 P35222

Q96B97 P35222

Q96CW1 P35222

Q96PU4 P35222

Q96RT1 P35222

Q99593 P35222

Q99697 P35222

Q9BZE0 P35222

Q9GZV5 P35222

Q9NQB0 P35222

Q9NSA3 P35222

Q9NYF0 P35222

Q9P287 P35222

Q9UJU2 P35222

Q9UKB1 P35222

Q9UKE5 P35222

Q9UPQ9 P35222

Q9Y297 P35222

P78552 P35225

O00257 P35226

O95503 P35226

O95931 P35226

P60484 P35226

P63279 P35226

Q06587 P35226

Q14781 P35226

Q15022 P35226

Q15910 P35226

Q3KNV8 P35226

Q93009 P35226

Q99496 P35226

Q9H270 P35226

Q9HC52 P35226

Q9Y508 P35226

O00257 P35227

O75461 P35227

O95503 P35227

O95931 P35227

P0CG48 P35227

P30279 P35227

P35226 P35227

P40337 P35227

P51784 P35227

Q06587 P35227

Q16665 P35227

Q5VVX9 P35227

Q8WVN8 P35227

Q93009 P35227

Q99496 P35227

Q9HC52 P35227

Q9UBT7 P35227

A0AUL9 P35232

O14647 P35232

O14672 P35232

O75306 P35232

O75489 P35232

P02751 P35232

P04626 P35232

P04637 P35232

P06454 P35232

P08238 P35232

P0CG48 P35232

P15336 P35232

P19320 P35232

P31946 P35232

P46821 P35232

P55209 P35232

P60520 P35232

P60709 P35232

Q13268 P35232

Q13286 P35232

Q13309 P35232

Q14197 P35232

Q496Y0 P35232

Q86U86 P35232

Q8TDR0 P35232

Q92576 P35232

Q93008 P35232

Q9GZQ8 P35232

Q9H0R8 P35232

Q9H3H9 P35232

Q9H9S3 P35232

Q9NXR7 P35232

Q9P013 P35232

Q9UKA4 P35232

Q9UKV8 P35232

Q9UL18 P35232

Q9UQD0 P35232

Q9UQL6 P35232

P19022 P35236

P28482 P35236

Q16539 P35236

Q96L14 P35237

Q16584 P35240

Q4VCS5 P35240

Q68EM7 P35240

Q8N3R9 P35240

Q8NI35 P35240

Q9BZE4 P35240

Q9H204 P35240

Q9P0W5 P35240

O14745 P35240

P03372 P35241

P05107 P35241

P08567 P35241

P0C862 P35241

P10911 P35241

P52565 P35241

P53779 P35241

Q6ZVD8 P35241

Q9H0R8 P35241

P84022 P35243

Q14161 P35243

P15927 P35244

P27694 P35244

P43351 P35244

P54132 P35244

Q5UIP0 P35244

Q9BQS8 P35244

P01106 P35249

P19320 P35249

P35250 P35249

P35251 P35249

P51858 P35249

P60520 P35249

Q13573 P35249

Q16658 P35249

Q92731 P35249

Q96RL1 P35249

Q99459 P35249

Q9Y2K6 P35249

P01106 P35250

P10644 P35250

P35249 P35250

P35251 P35250

P60520 P35250

Q13573 P35250

Q92731 P35250

Q99459 P35250

Q9Y2K6 P35250

O00716 P35251

P35249 P35251

P51858 P35251

P60520 P35251

Q92731 P35251

O95166 P35268

P03372 P35268

P19320 P35268

P32121 P35268

P49407 P35268

P51858 P35268

P55060 P35268

P60520 P35268

P60709 P35268

P60953 P35268

Q13573 P35268

Q5U5Q3 P35268

Q99459 P35268

Q9GZQ8 P35268

Q9H0R8 P35268

O94763 P35269

P13984 P35269

P21246 P35269

P21675 P35269

P35232 P35269

Q99689 P35269

P00533 P35321

P00519 P35326

P07947 P35326

P12931 P35326

P31751 P35326

Q08881 P35326

P01857 P35368

P02768 P35368

Q96SB3 P35368

P01210 P35372

P21333 P35372

P35212 P35372

Q15942 P35372

Q5JY77 P35372

Q5T9L3 P35372

Q8IUQ4 P35372

Q8TEW6 P35372

Q92905 P35372

Q9P0L0 P35372

Q9P104 P35372

Q9Y679 P35372

P51843 P35398

P35442 P35442

P35443 P35443

Q15843 P35498

Q92731 P35498

O95166 P35520

P10644 P35520

P35520 P35520

P51116 P35520

Q8IV36 P35520

Q9NP79 P35520

Q9Y5P4 P35520

O00716 P35527

O95166 P35527

P01857 P35527

P01871 P35527

P01876 P35527

P02647 P35527

P02768 P35527

P12830 P35527

P13569 P35527

P19320 P35527

P25054 P35527

P38919 P35527

P60520 P35527

Q00005 P35527

Q8TDR0 P35527

Q99963 P35527

Q9GZQ8 P35527

Q9H0R8 P35527

Q9H492 P35527

P01857 P35542

P02751 P35544

P07949 P35544

Q15545 P35544

O15265 P35555

O95967 P35555

P15502 P35555

P28300 P35555

Q9UBX5 P35555

P16118 P35557

P00736 P35558

P04626 P35568

P06213 P35568

P08069 P35568

P11940 P35568

P18031 P35568

P27986 P35568

P31947 P35568

P42336 P35568

P62258 P35568

P62993 P35568

P84022 P35568

Q06124 P35568

Q08345 P35568

Q96EB6 P35568

O43741 P35573

P54646 P35573

P60520 P35573

P83916 P35573

P84022 P35573

Q8WWW0 P35573

Q9H0R8 P35573

Q9P2Y5 P35573

Q9Y371 P35573

Q9Y5W8 P35575

O00255 P35579

O94763 P35579

O94782 P35579

P02751 P35579

P03372 P35579

P05067 P35579

P08238 P35579

P08473 P35579

P13569 P35579

P19320 P35579

P19338 P35579

P24844 P35579

P25815 P35579

P29353 P35579

P32121 P35579

P49407 P35579

P49662 P35579

P62993 P35579

P63000 P35579

P63104 P35579

Q13573 P35579

Q14457 P35579

Q15051 P35579

Q70EL2 P35579

Q8TDR0 P35579

Q8TF42 P35579

Q92905 P35579

Q96QT4 P35579

Q99459 P35579

Q9BX84 P35579

Q9H0R8 P35579

Q9H4A3 P35579

Q9H9G7 P35579

P02751 P35580

P11171 P35580

P19320 P35580

P25054 P35580

P32121 P35580

P62258 P35580

P62993 P35580

Q14457 P35580

Q15334 P35580

Q70EL2 P35580

Q92905 P35580

Q96QT4 P35580

Q9BX84 P35580

P08238 P35590

P08575 P35590

Q15238 P35590

Q99750 P35590

P19838 P35606

P49755 P35606

Q13573 P35606

Q15714 P35606

Q92731 P35606

Q99459 P35606

Q9BVK6 P35606

Q9GZZ9 P35606

Q9Y2Q3 P35606

O15265 P35609

O75386 P35609

O95295 P35609

P03950 P35609

P22459 P35609

P22460 P35609

P29474 P35609

P35609 P35609

P54296 P35609

P62736 P35609

P63104 P35609

P68366 P35609

Q15942 P35609

Q53GG5 P35609

Q6P0Q8 P35609

Q8WZ42 P35609

Q9H8T0 P35609

Q9NRI5 P35609

Q9UKG1 P35609

Q9Y5Z9 P35610

P22670 P35611

Q9GZQ8 P35611

O14593 P35613

P15336 P35613

P49459 P35613

Q70EL3 P35613

Q9H6Z4 P35613

Q9NR12 P35613

Q9UBB4 P35613

P50052 P35625

Q9UNA0 P35625

P25098 P35626

O15264 P35637

O43463 P35637

O75494 P35637

O75554 P35637

O95166 P35637

P02751 P35637

P03372 P35637

P11021 P35637

P11474 P35637

P19320 P35637

P35637 P35637

P40925 P35637

P60520 P35637

P61244 P35637

P62993 P35637

Q01844 P35637

Q13573 P35637

Q14151 P35637

Q15025 P35637

Q15149 P35637

Q15428 P35637

Q92804 P35637

Q969F2 P35637

Q99436 P35637

Q99459 P35637

Q99873 P35637

Q9GZQ8 P35637

Q9H0R8 P35637

Q9H3D4 P35637

Q9H492 P35637

Q9H7E2 P35637

Q9NRR4 P35637

Q9UBW7 P35637

O43791 P35638

P00734 P35638

P35638 P35638

Q09472 P35638

Q10586 P35638

Q16520 P35638

Q9BPX1 P35638

Q9BTE3 P35638

Q9Y2S0 P35638

Q9Y6K9 P35638

P01857 P35658

P19320 P35658

P24386 P35658

P25054 P35658

Q13283 P35658

Q6UXB4 P35658

Q9H0R8 P35658

Q9H492 P35658

Q9UBU9 P35658

Q9UN86 P35658

P03372 P35659

P19784 P35659

P68400 P35659

Q9UER7 P35659

O15105 P35711

O15266 P35711

P02794 P35711

Q00534 P35711

Q99717 P35711

O15266 P35712

Q99676 P35712

P63104 P35749

Q00403 P35749

Q8IY92 P35749

Q9BQ83 P35749

Q9UER7 P35749

P00505 P35754

P55064 P35754

P32121 P35813

P49407 P35813

Q9H6Z4 P35813

P06400 P35869

P27540 P35869

P35900 P35900

Q13057 P35900

Q53FD0 P35900

Q6PJT7 P35900

Q8IYI6 P35900

Q96IX9 P35900

Q9BUW7 P35900

Q9HAU0 P35900

Q9UBB9 P35900

O95166 P35908

P02751 P35908

P19320 P35908

P60520 P35908

Q00005 P35908

Q9GZQ8 P35908

Q9H0R8 P35908

Q9H492 P35908

P31689 P35914

Q14469 P35914

Q9H324 P35914

P08238 P35916

P35968 P35916

P49767 P35916

Q6VMQ6 P35916

O14786 P35968

O75340 P35968

P08581 P35968

P12931 P35968

P15692 P35968

P16333 P35968

P29350 P35968

P33151 P35968

P35916 P35968

P98160 P35968

Q12913 P35968

O00487 P35998

O75832 P35998

P01106 P35998

P12757 P35998

P51665 P35998

P54578 P35998

P62191 P35998

P62195 P35998

P62333 P35998

Q13573 P35998

Q16401 P35998

Q96DT6 P35998

Q99459 P35998

Q9NWV8 P35998

Q9Y5K5 P35998

Q9Y312 P36382

P35222 P36402

O43924 P36404

P02751 P36404

P19320 P36404

Q13432 P36404

Q8WZ55 P36404

Q9BTW9 P36404

O43924 P36405

P04637 P36405

Q04724 P36405

Q13432 P36405

Q5UIP0 P36405

Q8IWV7 P36405

Q8WZ55 P36405

Q9BTW9 P36405

O00716 P36406

P15622 P36406

P25786 P36406

P36406 P36406

P40692 P36406

P55040 P36406

P62256 P36406

Q0VDD7 P36406

Q12815 P36406

Q15637 P36406

Q5VVX9 P36406

Q8NFF5 P36406

Q8TBB1 P36406

Q96HA1 P36406

Q96LR5 P36406

Q99816 P36406

Q99933 P36406

Q9BV68 P36406

Q9BYV6 P36406

Q9H4K1 P36406

Q9NS91 P36406

Q9NTK1 P36406

Q9P0T4 P36406

Q9UIF8 P36406

Q9UJY4 P36406

O75381 P36507

P04049 P36507

P10398 P36507

P15056 P36507

P36507 P36507

Q12959 P36507

Q13573 P36507

Q9Y4F9 P36507

O43570 P36542

O43676 P36542

P00533 P36542

P01009 P36542

P01106 P36542

P02751 P36542

P04626 P36542

P19320 P36542

P19823 P36542

P21246 P36542

P24539 P36542

P30281 P36542

P60002 P36542

Q00013 P36542

Q14181 P36542

Q14197 P36542

Q8N5V2 P36542

Q8NG27 P36542

Q96IZ0 P36542

Q99832 P36542

Q9H0R8 P36542

Q9H845 P36542

Q9HCC8 P36542

Q9HCK5 P36542

Q9NY33 P36542

Q9UKV8 P36542

Q9UL18 P36542

Q9UQN3 P36542

O75348 P36543

O95670 P36543

P12956 P36543

P38606 P36543

Q14696 P36543

P05067 P36544

P54253 P36544

O14641 P36575

O14733 P36575

P04632 P36575

P32121 P36575

P49407 P36575

Q14749 P36575

Q8IZ40 P36575

Q93091 P36575

Q96IT1 P36575

O95166 P36578

P02751 P36578

P03372 P36578

P19320 P36578

P26641 P36578

P32121 P36578

P38919 P36578

P42858 P36578

P60520 P36578

P61326 P36578

P62993 P36578

P84022 P36578

Q00005 P36578

Q13573 P36578

Q9C026 P36578

Q9GZQ8 P36578

Q9H0R8 P36578

Q9H492 P36578

Q9UBU9 P36578

Q9UKV8 P36578

Q9UL18 P36578

Q9UQ80 P36578

O95166 P36639

P60520 P36639

Q9GZQ8 P36639

Q9H0R8 P36639

Q9HC36 P36639

O14879 P36776

O75604 P36776

P01023 P36776

P01106 P36776

P08473 P36776

P19320 P36776

Q13325 P36776

Q13418 P36776

Q14197 P36776

Q92731 P36776

Q96JB6 P36776

Q9BSB4 P36776

Q9H0A6 P36776

O60333 P36871

Q9GZZ9 P36871

O14503 P36873

O94763 P36873

O95166 P36873

P03372 P36873

P19320 P36873

P19525 P36873

P22415 P36873

P41236 P36873

P50750 P36873

P60520 P36873

Q13625 P36873

Q15435 P36873

Q8IZD2 P36873

Q8NG31 P36873

Q8WUF5 P36873

Q96C90 P36873

Q96KQ4 P36873

Q9GZQ8 P36873

Q9H492 P36873

A1DRY3 P36873

O15392 P36873

O60674 P36873

P04637 P36873

P06493 P36873

P11802 P36873

P17028 P36873

P24385 P36873

P24941 P36873

P30281 P36873

P49427 P36873

P61244 P36873

Q07817 P36873

Q13315 P36873

Q4KMQ1 P36873

Q5JR98 P36873

Q5SWA1 P36873

Q5SZB4 P36873

Q6NYC8 P36873

Q8IWU2 P36873

Q8N726 P36873

Q8TEC5 P36873

Q96S59 P36873

O43556 P36873

O95819 P36873

P02745 P36873

P23921 P36873

P51955 P36873

P62993 P36873

Q38J66 P36873

Q6B0I6 P36873

Q6PJW8 P36873

Q6UWU2 P36873

Q7L576 P36873

Q7Z5V6 P36873

Q8N5T2 P36873

Q8TAP8 P36873

Q8WXF2 P36873

Q92905 P36873

Q96GX9 P36873

Q9BS83 P36873

Q9BXJ8 P36873

Q9H0A9 P36873

Q9H0I2 P36873

Q9HBL0 P36873

Q9NR20 P36873

Q9NYF0 P36873

Q9UBL3 P36873

Q9UHY1 P36873

P27986 P36888

Q06124 P36888

Q9Y6K9 P36888

P12643 P36894

P12644 P36894

P43026 P36894

O15105 P36896

P08238 P36896

P22694 P36896

P62942 P36896

P01137 P36897

P10600 P36897

P62942 P36897

P63104 P36897

P98082 P36897

Q9Y3F4 P36897

Q9UDV6 P36915

O75398 P36941

P36941 P36941

Q12933 P36941

Q13114 P36941

Q14469 P36941

Q15326 P36941

Q8IY92 P36952

Q9Y6M0 P36952

A0JLT2 P36954

O95402 P36954

Q8TAF3 P36954

Q9BTT4 P36954

Q9BTW9 P36954

Q9NX70 P36954

P40692 P36955

P22415 P36956

Q96RN5 P36956

P01023 P36957

P35080 P36957

P60520 P36957

P62993 P36957

Q05516 P36957

Q8TDM6 P36957

Q92597 P36957

Q9UKY1 P36957

Q96G74 P36969

P40692 P37058

O00418 P37108

P01106 P37108

P02751 P37108

P19320 P37108

P40337 P37108

P62993 P37108

P67809 P37108

Q9H0R8 P37108

Q9NX58 P37108

Q9NY93 P37108

P01137 P37173

P10600 P37173

P29353 P37173

P35243 P37173

P98082 P37173

Q12841 P37173

Q8IX30 P37173

Q93074 P37173

Q9UER7 P37173

P05413 P37198

P24386 P37198

Q13123 P37198

Q13283 P37198

Q14653 P37198

Q5S007 P37198

Q7Z3B4 P37198

Q9H8Y8 P37198

Q9NVV9 P37198

Q9UBU9 P37198

Q9UN86 P37198

Q9Y3C0 P37198

Q9Y547 P37198

O60869 P37231

O95819 P37231

P04156 P37231

P07437 P37231

P16949 P37231

P21283 P37231

P51532 P37231

P51784 P37231

Q09472 P37231

Q15648 P37231

Q15942 P37231

Q3V6T2 P37231

Q6STE5 P37231

Q96CV9 P37231

Q99460 P37231

Q9BUJ2 P37231

Q9UBK2 P37231

P03372 P37235

Q86UW9 P37235

Q9NP66 P37235

P02751 P37268

Q969S8 P37268

Q9Y265 P37268

Q13363 P37275

O15264 P37802

O94782 P37802

P15336 P37802

P16104 P37802

P19320 P37802

P60709 P37802

Q9H0R8 P37802

Q9NZN4 P37802

P19320 P37837

Q9UKY1 P37837

O75821 P37840

O94811 P37840

P08107 P37840

P10636 P37840

P17600 P37840

P37840 P37840

P42858 P37840

P49841 P37840

P68104 P37840

Q01959 P37840

Q5S007 P37840

Q9UI14 P37840

P13804 P38117

P49789 P38117

O14543 P38159

O14879 P38159

P03372 P38159

P09651 P38159

P10588 P38159

P19320 P38159

P35637 P38159

P61978 P38159

P62995 P38159

Q13573 P38159

Q14011 P38159

Q5VWX1 P38159

Q70EL2 P38159

Q8N5R6 P38159

Q8TBB1 P38159

Q8TDY2 P38159

Q99459 P38159

Q9BTL3 P38159

Q9GZQ8 P38159

Q9H492 P38159

Q9UPE1 P38159

Q9Y580 P38159

O14757 P38398

O15164 P38398

O15360 P38398

O60674 P38398

O76064 P38398

O96017 P38398

P00519 P38398

P03372 P38398

P04637 P38398

P10415 P38398

P10809 P38398

P16104 P38398

P16220 P38398

P16333 P38398

P23458 P38398

P24864 P38398

P31749 P38398

P33240 P38398

P36873 P38398

P38398 P38398

P51948 P38398

P52292 P38398

P54727 P38398

P62140 P38398

P62805 P38398

P78347 P38398

Q12888 P38398

Q13085 P38398

Q14192 P38398

Q14676 P38398

Q14683 P38398

Q16666 P38398

Q17RB8 P38398

Q6NZY4 P38398

Q6UWZ7 P38398

Q7Z569 P38398

Q86YC2 P38398

Q8WX92 P38398

Q92560 P38398

Q92830 P38398

Q96RL1 P38398

Q99708 P38398

Q99728 P38398

Q9BX63 P38398

Q9BXW9 P38398

Q9GZX5 P38398

Q9UBD5 P38398

Q9Y385 P38398

Q9Y4A5 P38398

Q9NWV8 P38405

Q9UM82 P38405

Q9Y6I4 P38405

O00505 P38432

O43707 P38432

O60229 P38432

P12814 P38432

P12956 P38432

P13010 P38432

P19784 P38432

P35609 P38432

P38432 P38432

P54253 P38432

P61289 P38432

P67870 P38432

Q07021 P38432

Q12800 P38432

Q15020 P38432

Q16637 P38432

Q4J6C6 P38432

Q58EX7 P38432

Q5JVS0 P38432

Q5TD97 P38432

Q5W0Q7 P38432

Q674X7 P38432

Q6IAU5 P38432

Q86Y07 P38432

Q8NHQ1 P38432

Q8TBB1 P38432

Q92731 P38432

Q96JN2 P38432

Q96KG9 P38432

Q96RS0 P38432

Q99873 P38432

Q99986 P38432

Q9BQG0 P38432

Q9H0B6 P38432

Q9H2G4 P38432

Q9NR22 P38432

Q9NRD5 P38432

Q9NRM2 P38432

Q9NSK0 P38432

Q9P2G9 P38432

Q9ULV0 P38432

Q9UNH7 P38432

Q9Y3D8 P38432

Q13557 P38484

P01023 P38606

A0JLT2 P38646

O00716 P38646

O15350 P38646

O43504 P38646

O95166 P38646

O95777 P38646

P00533 P38646

P03372 P38646

P04637 P38646

P10415 P38646

P13489 P38646

P13569 P38646

P15056 P38646

P16104 P38646

P19320 P38646

P19438 P38646

P27824 P38646

P31749 P38646

P42771 P38646

P49407 P38646

P60520 P38646

Q00005 P38646

Q14197 P38646

Q15051 P38646

Q5S007 P38646

Q6PJG9 P38646

Q7Z6M2 P38646

Q8N0X7 P38646

Q8TDR0 P38646

Q8WX92 P38646

Q96CV9 P38646

Q99558 P38646

Q9GZQ8 P38646

Q9H0R8 P38646

Q9H492 P38646

Q9H9G7 P38646

Q9NTG7 P38646

Q9NWA0 P38646

Q9UKV8 P38646

O15234 P38919

P19320 P38919

P27348 P38919

P49748 P38919

P61326 P38919

P62993 P38919

P63104 P38919

P78362 P38919

Q09161 P38919

Q13573 P38919

Q6PII3 P38919

Q92900 P38919

Q96Q15 P38919

Q96SB4 P38919

Q99459 P38919

Q9BY77 P38919

Q9BZI7 P38919

Q9H1J1 P38919

Q9HCG8 P38919

Q9UKV8 P38919

Q9UL18 P38919

Q9Y5S9 P38919

O00311 P38936

O75419 P38936

O75496 P38936

O94921 P38936

O95067 P38936

O95741 P38936

O96020 P38936

P01023 P38936

P02675 P38936

P04217 P38936

P04637 P38936

P06493 P38936

P06576 P38936

P07199 P38936

P08670 P38936

P0CG47 P38936

P10074 P38936

P10599 P38936

P11177 P38936

P11802 P38936

P12004 P38936

P13196 P38936

P13378 P38936

P14618 P38936

P14635 P38936

P15880 P38936

P24385 P38936

P24864 P38936

P24941 P38936

P30279 P38936

P30281 P38936

P32754 P38936

P33993 P38936

P38606 P38936

P43246 P38936

P46379 P38936

P47897 P38936

P52742 P38936

P60709 P38936

P61978 P38936

P62328 P38936

P62879 P38936

P63244 P38936

P68104 P38936

P78396 P38936

Q00526 P38936

Q00534 P38936

Q00535 P38936

Q02978 P38936

Q07020 P38936

Q12766 P38936

Q13309 P38936

Q14397 P38936

Q15025 P38936

Q15834 P38936

Q6FI35 P38936

Q6P4I2 P38936

Q6PKC3 P38936

Q6UXH1 P38936

Q71U36 P38936

Q75N90 P38936

Q7L590 P38936

Q7L5D6 P38936

Q7L5N1 P38936

Q7Z699 P38936

Q8IYF3 P38936

Q8NBE8 P38936

Q8NFF5 P38936

Q8NFZ5 P38936

Q8TF32 P38936

Q8WYH8 P38936

Q92934 P38936

Q93063 P38936

Q969F2 P38936

Q96DB2 P38936

Q96FN4 P38936

Q96FS4 P38936

Q96FW1 P38936

Q96N67 P38936

Q99459 P38936

Q99741 P38936

Q9BQ15 P38936

Q9BRK5 P38936

Q9H324 P38936

Q9HB58 P38936

Q9NPI5 P38936

Q9NWV4 P38936

Q9UHC7 P38936

Q9UID3 P38936

Q9Y3C7 P38936

Q9Y600 P38936

P11309 P39019

P19320 P39019

P32121 P39019

P39019 P39019

P43351 P39019

P60520 P39019

P60709 P39019

P62263 P39019

P62277 P39019

P62280 P39019

Q13573 P39019

Q5U5Q3 P39019

Q9H0R8 P39019

O95166 P39023

P03372 P39023

P19320 P39023

P30480 P39023

P32121 P39023

P38919 P39023

P49407 P39023

P49757 P39023

P57059 P39023

P60520 P39023

P61326 P39023

P62993 P39023

P84022 P39023

Q13573 P39023

Q9H0R8 P39023

Q9H3D4 P39023

Q9H492 P39023

Q9UBU9 P39023

Q9UKV8 P39023

P39059 P39059

P39060 P39060

Q7Z6L1 P39060

Q8N157 P39060

P05067 P39060

P07996 P39060

P19338 P39060

P21980 P39060

P01023 P39656

P15336 P39656

P19320 P39656

P46736 P39656

Q16659 P39656

Q86WV6 P39656

Q9H0R8 P39656

Q9H9G7 P39656

Q9UKV8 P39656

Q9UL18 P39656

O15131 P39687

O60684 P39687

P04150 P39687

P35222 P39687

P52294 P39687

P67775 P39687

Q12988 P39687

Q66K89 P39687

P02751 P39748

P12004 P39748

P26641 P39748

P49841 P39748

P54132 P39748

Q13573 P39748

Q14191 P39748

Q99459 P39748

O00499 P39880

O43570 P39880

O94762 P39880

P06400 P39880

Q9BQ52 P39880

Q13573 P40121

Q99459 P40121

P62258 P40123

P13569 P40145

P05231 P40189

P13725 P40189

P26441 P40189

P42224 P40189

Q8N3K9 P40189

P06731 P40199

P40199 P40199

Q16568 P40199

Q8N3L3 P40222

Q9UHD2 P40222

Q09472 P40225

O14732 P40227

O15264 P40227

O15379 P40227

O75381 P40227

P01106 P40227

P04637 P40227

P12830 P40227

P17987 P40227

P19320 P40227

P32121 P40227

P60510 P40227

P60520 P40227

P62714 P40227

P67775 P40227

P78318 P40227

Q00005 P40227

Q00535 P40227

Q13033 P40227

Q13326 P40227

Q13418 P40227

Q13573 P40227

Q15257 P40227

Q15796 P40227

Q66LE6 P40227

Q8WZ42 P40227

Q8WZ74 P40227

Q99459 P40227

Q9GZQ8 P40227

Q9GZX7 P40227

Q9H492 P40227

Q9H7D0 P40227

Q9NRL3 P40227

Q9UH92 P40227

Q9UKK3 P40227

Q9Y2T4 P40227

Q9Y3A3 P40227

Q9Y6E0 P40227

Q8WWM7 P40238

P18850 P40261

O95273 P40306

P20073 P40306

P21246 P40306

P25786 P40306

P25787 P40306

P49720 P40306

Q99436 P40306

A0N4V7 P40337

O00264 P40337

O14737 P40337

O15067 P40337

O43294 P40337

O43809 P40337

O60493 P40337

O60888 P40337

O75396 P40337

O75608 P40337

O75643 P40337

O75694 P40337

O75716 P40337

O75912 P40337

O94888 P40337

O95260 P40337

O95267 P40337

O95340 P40337

P00492 P40337

P01024 P40337

P02751 P40337

P04080 P40337

P04179 P40337

P05455 P40337

P07237 P40337

P07355 P40337

P07741 P40337

P08134 P40337

P08151 P40337

P09661 P40337

P09936 P40337

P10599 P40337

P12268 P40337

P13693 P40337

P13797 P40337

P14174 P40337

P15121 P40337

P15170 P40337

P15311 P40337

P16949 P40337

P17844 P40337

P19105 P40337

P20290 P40337

P20340 P40337

P20618 P40337

P20674 P40337

P20701 P40337

P20930 P40337

P21980 P40337

P22061 P40337

P23284 P40337

P23508 P40337

P24539 P40337

P24666 P40337

P24941 P40337

P25787 P40337

P26441 P40337

P26639 P40337

P26640 P40337

P28074 P40337

P29401 P40337

P30046 P40337

P30084 P40337

P31939 P40337

P31949 P40337

P33176 P40337

P33993 P40337

P35080 P40337

P35227 P40337

P35244 P40337

P35268 P40337

P37802 P40337

P39019 P40337

P42766 P40337

P42771 P40337

P43307 P40337

P43487 P40337

P46781 P40337

P46783 P40337

P47813 P40337

P47914 P40337

P47985 P40337

P48047 P40337

P48739 P40337

P49257 P40337

P49720 P40337

P49755 P40337

P49773 P40337

P50395 P40337

P50748 P40337

P51148 P40337

P51149 P40337

P53396 P40337

P56134 P40337

P58546 P40337

P60660 P40337

P60900 P40337

P61019 P40337

P61077 P40337

P61081 P40337

P61086 P40337

P61088 P40337

P61106 P40337

P61513 P40337

P61604 P40337

P61626 P40337

P62158 P40337

P62249 P40337

P62330 P40337

P62491 P40337

P62750 P40337

P62807 P40337

P62834 P40337

P62877 P40337

P62942 P40337

P63244 P40337

P68036 P40337

P78396 P40337

P99999 P40337

Q00005 P40337

Q01105 P40337

Q01469 P40337

Q02543 P40337

Q04724 P40337

Q04941 P40337

Q05513 P40337

Q12996 P40337

Q13315 P40337

Q13404 P40337

Q13526 P40337

Q13617 P40337

Q14019 P40337

Q14197 P40337

Q14203 P40337

Q15019 P40337

Q15185 P40337

Q15286 P40337

Q15365 P40337

Q15369 P40337

Q15370 P40337

Q15459 P40337

Q15843 P40337

Q16658 P40337

Q16665 P40337

Q3MHD2 P40337

Q3ZAQ7 P40337

Q5SQT9 P40337

Q5XKP0 P40337

Q6FGD7 P40337

Q6FGG2 P40337

Q6FGU2 P40337

Q6FHT8 P40337

Q6P1L8 P40337

Q7Z443 P40337

Q86SX6 P40337

Q8IUC4 P40337

Q8N163 P40337

Q8N4S0 P40337

Q8N6L1 P40337

Q8TBK9 P40337

Q8TBZ2 P40337

Q8TE49 P40337

Q8WU17 P40337

Q92830 P40337

Q92839 P40337

Q92905 P40337

Q92922 P40337

Q969H8 P40337

Q96AG4 P40337

Q96FW1 P40337

Q96KM6 P40337

Q96QK1 P40337

Q99417 P40337

Q99471 P40337

Q99595 P40337

Q99700 P40337

Q99873 P40337

Q9BRA2 P40337

Q9BRT2 P40337

Q9H0U4 P40337

Q9H773 P40337

Q9HAV7 P40337

Q9NR22 P40337

Q9NZ23 P40337

Q9P031 P40337

Q9P258 P40337

Q9UBQ5 P40337

Q9UDY8 P40337

Q9UHV9 P40337

Q9UI30 P40337

Q9ULC4 P40337

Q9UM00 P40337

Q9UNM6 P40337

Q9UQN3 P40337

Q9Y266 P40337

Q9Y376 P40337

Q9Y3A3 P40337

Q9Y3B4 P40337

Q9Y3C8 P40337

Q9Y3D3 P40337

Q9Y3D9 P40337

Q9Y4L1 P40337

Q9Y547 P40337

Q9Y5B9 P40337

Q9Y5M8 P40337

Q9UM11 P40337

O00470 P40424

O14770 P40424

P40425 P40424

Q12948 P40424

P55347 P40424

A6NC98 P40425

O75935 P40425

O95163 P40425

P31314 P40425

P36578 P40425

P37198 P40425

P42285 P40425

P55347 P40425

P78537 P40425

Q7L5N1 P40425

Q96EZ8 P40425

Q96S38 P40425

Q9H7B4 P40425

Q9NXF8 P40425

Q9UQB8 P40425

P31314 P40426

P55347 P40426

P02741 P40429

P02751 P40429

P03372 P40429

P03886 P40429

P05091 P40429

P05181 P40429

P14921 P40429

P19320 P40429

P38919 P40429

P49354 P40429

P60520 P40429

P61326 P40429

P62888 P40429

Q5U5Q3 P40429

Q9H0R8 P40429

Q9H3D4 P40429

Q9UKV8 P40429

Q9UL18 P40429

O95758 P40616

P01106 P40616

P08473 P40616

Q8IWJ2 P40616

O00299 P40692

O15484 P40692

O43929 P40692

O75152 P40692

O94941 P40692

O95831 P40692

P00966 P40692

P01834 P40692

P04075 P40692

P07355 P40692

P07741 P40692

P07858 P40692

P10599 P40692

P13639 P40692

P17661 P40692

P36955 P40692

P38398 P40692

P41222 P40692

P46782 P40692

P52564 P40692

P56470 P40692

P60660 P40692

P61619 P40692

P62249 P40692

P62328 P40692

P63261 P40692

Q02817 P40692

Q0PNE2 P40692

Q13501 P40692

Q13573 P40692

Q13813 P40692

Q14134 P40692

Q15080 P40692

Q16658 P40692

Q5T0D9 P40692

Q6NSI4 P40692

Q7Z7L7 P40692

Q8NA61 P40692

Q92597 P40692

Q96NE9 P40692

Q99757 P40692

Q9BV35 P40692

Q9BV40 P40692

Q9BX63 P40692

Q9H9S4 P40692

Q9HD43 P40692

Q9NUN7 P40692

Q9NV92 P40692

Q9NVD7 P40692

Q9UHV5 P40692

Q9UID3 P40692

O00570 P40763

O14874 P40763

O14920 P40763

O43255 P40763

O43318 P40763

O60232 P40763

O95661 P40763

P00533 P40763

P04626 P40763

P06401 P40763

P07384 P40763

P07949 P40763

P08047 P40763

P11309 P40763

P12931 P40763

P15822 P40763

P22736 P40763

P30084 P40763

P31146 P40763

P40763 P40763

P42224 P40763

P43405 P40763

P45984 P40763

P46781 P40763

P55268 P40763

P58753 P40763

Q06520 P40763

Q13011 P40763

Q13547 P40763

Q14192 P40763

Q15910 P40763

Q8IUQ4 P40763

Q8N960 P40763

Q8NEM7 P40763

Q8TAE8 P40763

Q8TE76 P40763

Q92665 P40763

Q96G01 P40763

Q96RT1 P40763

Q99062 P40763

Q99459 P40763

Q9BVP2 P40763

Q9GZT8 P40763

Q9UBE8 P40763

Q9UER7 P40763

Q9ULD0 P40763

Q9Y5S9 P40763

P27348 P40818

P61981 P40818

P63104 P40818

Q04917 P40818

Q13107 P40818

Q14596 P40818

Q7LBR1 P40818

Q96CF2 P40818

Q9HD42 P40818

Q9UQN3 P40818

Q9Y3E7 P40818

O00623 P40855

O43482 P40855

O60683 P40855

O60739 P40855

O75381 P40855

O96011 P40855

P00533 P40855

P28288 P40855

P28328 P40855

P30304 P40855

P33897 P40855

P40855 P40855

P56589 P40855

Q13188 P40855

Q15796 P40855

Q7Z412 P40855

Q92845 P40855

Q92968 P40855

Q96SB4 P40855

Q9NR77 P40855

Q9P0R6 P40855

Q9UBJ2 P40855

Q9Y5Y5 P40855

P08238 P40925

Q15051 P40925

Q9H1Y0 P40925

O15264 P40926

O95166 P40926

P02751 P40926

P12004 P40926

P15336 P40926

P19320 P40926

P23381 P40926

P49789 P40926

P60520 P40926

Q9H0R8 P40926

Q9H492 P40926

Q13261 P40933

O95166 P40937

P01106 P40937

P35249 P40937

P35250 P40937

P35251 P40937

P51858 P40937

P68104 P40937

Q13432 P40937

Q13573 P40937

Q5RL73 P40937

Q5UIP0 P40937

Q7L5N1 P40937

Q99459 P40937

Q9Y3C7 P40937

P01106 P40938

P35249 P40938

P35250 P40938

P35251 P40938

Q13573 P40938

Q14696 P40938

Q16658 P40938

Q99459 P40938

Q9Y2K6 P40938

O00716 P40939

O75323 P40939

O75815 P40939

O95166 P40939

P02751 P40939

P04233 P40939

P15336 P40939

P19320 P40939

P19784 P40939

P60520 P40939

Q00653 P40939

Q06187 P40939

Q13501 P40939

Q14197 P40939

Q15051 P40939

Q5U5Q3 P40939

Q70CQ1 P40939

Q8N0X7 P40939

Q9BSB4 P40939

Q9BW61 P40939

Q9GZQ8 P40939

Q9H0R8 P40939

Q9H492 P40939

O43303 P41002

P31350 P41002

P02751 P41091

P05198 P41091

P20042 P41091

P43146 P41091

P51858 P41091

Q13573 P41091

Q16659 P41091

Q92597 P41091

Q99459 P41091

O14936 P41134

O43463 P41134

P15923 P41134

Q03135 P41134

P16615 P41143

P27824 P41143

Q5T9L3 P41145

Q8IUQ4 P41145

Q9H0R8 P41145

Q9P0L0 P41145

Q13519 P41146

Q5JY77 P41146

P20815 P41161

P51681 P41161

Q6UXD5 P41161

Q96DB2 P41161

Q9NSE2 P41161

Q9Y394 P41161

P01106 P41162

Q9BZ95 P41162

Q15363 P41180

P56524 P41182

Q9UKV0 P41182

Q9ULU4 P41182

A8K8P3 P41208

O43303 P41208

P54727 P41208

Q70CQ1 P41208

Q9H0E7 P41208

P01106 P41212

Q13573 P41212

Q99459 P41212

Q9UBN7 P41212

Q9UKV0 P41212

Q06609 P41214

Q99459 P41214

Q8TD46 P41217

Q9BXL5 P41218

Q9BZ95 P41218

P08670 P41219

P60520 P41219

Q92731 P41219

Q9H0R8 P41219

O60260 P41220

O95858 P41220

P07858 P41220

P09496 P41220

P0CG48 P41220

P11142 P41220

P12956 P41220

P14314 P41220

P21964 P41220

P60228 P41220

P61224 P41220

P63167 P41220

Q00765 P41220

Q08345 P41220

Q12873 P41220

Q13467 P41220

Q14524 P41220

Q15942 P41220

Q6RFH5 P41220

Q8N6T3 P41220

Q8NAF0 P41220

Q8TB24 P41220

Q9BPW8 P41220

Q9H596 P41220

Q9P244 P41220

Q9P2D0 P41220

Q9P2H0 P41220

Q9UBQ0 P41220

Q9ULJ8 P41220

Q9Y262 P41220

Q9Y2X7 P41220

Q9Y5U9 P41220

O00555 P41222

P00533 P41222

P40692 P41222

P51828 P41223

P05161 P41226

P32121 P41227

P41227 P41227

Q14155 P41227

Q15052 P41227

Q16665 P41227

Q96KQ7 P41227

A8MYZ6 P41235

O75716 P41235

O76070 P41235

Q12772 P41235

Q13310 P41235

Q92878 P41235

Q93063 P41235

Q99967 P41235

P01106 P41236

P36873 P41236

P49840 P41236

P49841 P41236

P62136 P41236

Q8IWU2 P41236

P04626 P41240

P06241 P41240

P08575 P41240

P21860 P41240

P22681 P41240

P23470 P41240

P29353 P41240

P41240 P41240

P49023 P41240

P62993 P41240

Q13283 P41240

Q13573 P41240

Q14108 P41240

Q6GTX8 P41240

Q8NHL6 P41240

Q8TF42 P41240

Q9C0H9 P41240

Q9NWQ8 P41240

P60520 P41250

O95166 P41252

P07814 P41252

P19320 P41252

P63104 P41252

Q9GZQ8 P41252

D3DR86 P41279

O96019 P41279

P04350 P41279

P07437 P41279

P07900 P41279

P08238 P41279

P11021 P41279

P11142 P41279

P15924 P41279

P17858 P41279

P19838 P41279

P34931 P41279

P38646 P41279

P41279 P41279

P58107 P41279

P62158 P41279

Q00610 P41279

Q00653 P41279

Q04206 P41279

Q05639 P41279

Q08380 P41279

Q13748 P41279

Q8NFZ5 P41279

Q96HD9 P41279

Q9UQC1 P41279

Q9Y265 P41279

O00555 P41732

O95166 P41743

P02766 P41743

P60520 P41743

P60953 P41743

P63000 P41743

Q04917 P41743

Q13501 P41743

Q15714 P41743

Q6P1M3 P41743

Q7KZI7 P41743

Q8IY92 P41743

Q8ND90 P41743

Q8TEW0 P41743

Q96L34 P41743

Q9BYG4 P41743

Q9BYG5 P41743

Q9GZQ8 P41743

Q9H0R8 P41743

Q9H492 P41743

Q9NPB6 P41743

Q9UK80 P41743

Q9Y6K9 P41743

P62993 P41970

Q16543 P41970

P60709 P42025

Q12873 P42025

Q14203 P42025

O60344 P42081

P09874 P42081

P16410 P42081

Q14197 P42126

Q9NUX5 P42126

A0JLT2 P42166

O15264 P42166

O95402 P42166

O95758 P42166

P01106 P42166

P13010 P42166

P19320 P42166

P32121 P42166

P49407 P42166

P51858 P42166

Q13573 P42166

Q5S007 P42166

Q99459 P42166

Q9BQS8 P42166

Q9NWA0 P42166

Q9NX70 P42166

P00519 P42167

P35222 P42167

Q00005 P42167

O43707 P42224

P00533 P42224

P03901 P42224

P04626 P42224

P09619 P42224

P14136 P42224

P15260 P42224

P19793 P42224

P32121 P42224

P40189 P42224

P40763 P42224

P42224 P42224

P52630 P42224

P59826 P42224

Q01094 P42224

Q15155 P42224

Q16531 P42224

Q5JPE7 P42224

Q7Z434 P42224

Q8N9N8 P42224

Q969M7 P42224

P24394 P42226

P42226 P42226

P68104 P42226

Q7Z434 P42226

Q86WV6 P42226

Q9UHD2 P42226

A8CG34 P42229

P03372 P42229

P11712 P42229

P14784 P42229

P18031 P42229

P32927 P42229

P38159 P42229

P42229 P42229

P46109 P42229

P60484 P42229

P62879 P42229

P82932 P42229

Q6PKC3 P42229

Q8TC21 P42229

Q92793 P42229

Q99490 P42229

Q99814 P42229

Q9NRG1 P42229

Q9UGK3 P42229

P17612 P42261

P46459 P42262

Q8WUA4 P42262

O95166 P42285

P01876 P42285

P19320 P42285

P30153 P42285

P49760 P42285

Q00005 P42285

Q01780 P42285

Q13868 P42285

Q15759 P42285

Q66LE6 P42285

Q99459 P42285

Q99547 P42285

Q5UIP0 P42330

Q96B02 P42330

Q9UKV3 P42330

Q9UKY1 P42330

O00459 P42336

P02792 P42336

P21860 P42336

P22681 P42336

P27986 P42336

P35568 P42336

P42336 P42336

P60866 P42336

P62993 P42336

Q05397 P42336

Q92569 P42336

O00459 P42338

P08631 P42338

P13196 P42338

P21860 P42338

P22681 P42338

P27348 P42338

P62993 P42338

Q12800 P42338

Q92569 P42338

Q92997 P42338

O43156 P42345

O43504 P42345

P06730 P42345

P42345 P42345

P46781 P42345

P55884 P42345

P61254 P42345

P62942 P42345

P63104 P42345

P63244 P42345

P85299 P42345

Q09161 P42345

Q0VGL1 P42345

Q13541 P42345

Q15019 P42345

Q15382 P42345

Q16543 P42345

Q6IAA8 P42345

Q6MZQ0 P42345

Q6R327 P42345

Q70Z35 P42345

Q8N122 P42345

Q8N6M7 P42345

Q8TB45 P42345

Q8TCU6 P42345

Q96B36 P42345

Q96EB6 P42345

Q9BPZ7 P42345

Q9BVC4 P42345

Q9NWS0 P42345

Q9P2J5 P42345

Q9Y265 P42345

Q9Y2Q5 P42345

Q9Y4R8 P42345

P02751 P42356

P13805 P42356

P19320 P42356

P49286 P42356

Q9H9J2 P42356

A9UF07 P42566

P00519 P42566

P00533 P42566

P02751 P42566

P04626 P42566

P16333 P42566

P19320 P42566

P42566 P42566

P46108 P42566

P49757 P42566

P52594 P42566

P62993 P42566

Q00987 P42566

Q14974 P42566

Q8WXE9 P42566

Q96CW1 P42566

Q9P0V3 P42566

Q9UMX0 P42566

O00472 P42568

O95402 P42568

P21246 P42568

P50750 P42568

P68104 P42568

Q5RL73 P42568

Q5UIP0 P42568

Q8TEK3 P42568

Q9UHB7 P42568

O14727 P42574

O75159 P42574

P15976 P42574

P35754 P42574

P42574 P42574

Q00987 P42574

Q009871 P42574

Q96CA5 P42574

Q9BYP7 P42574

Q9Y243 P42574

P19793 P42575

P42575 P42575

P62888 P42575

P78527 P42575

P78560 P42575

Q9HB75 P42575

P35232 P42658

O15069 P42677

O95758 P42677

P12757 P42677

P12814 P42677

P19320 P42677

P56192 P42677

Q00005 P42677

Q00987 P42677

Q9H6R7 P42677

Q9NQC3 P42677

Q9UHY7 P42677

Q9UKV8 P42677

Q9Y227 P42677

Q9Y328 P42677

P04626 P42679

Q14289 P42679

P08575 P42680

P23467 P42680

P62993 P42680

Q12913 P42680

Q15262 P42680

P00533 P42684

P01112 P42684

P04626 P42684

P12931 P42684

P16333 P42684

P19174 P42684

P21860 P42684

P27986 P42684

P46108 P42684

P62993 P42684

Q02779 P42684

Q13671 P42684

Q15303 P42684

Q16890 P42684

Q6PIJ6 P42684

Q8IZP0 P42684

P06400 P42685

P41240 P42685

P60484 P42685

Q12982 P42685

P55854 P42694

P01106 P42695

Q6IBW4 P42695

Q8IY92 P42695

P60520 P42696

Q96DB2 P42696

Q9H0R8 P42696

Q9UPE1 P42696

Q9Y6K9 P42696

O00716 P42704

O75381 P42704

O94966 P42704

P01106 P42704

P04626 P42704

P06730 P42704

P19320 P42704

P21860 P42704

P60709 P42704

Q14197 P42704

Q15327 P42704

Q5U5Q3 P42704

Q70CQ1 P42704

Q9BSB4 P42704

Q9GZT3 P42704

Q9NXR7 P42704

O43504 P42765

O43586 P42766

P02751 P42766

P19320 P42766

P32121 P42766

P35609 P42766

P38936 P42766

P51858 P42766

P61244 P42766

Q16594 P42766

Q96HU1 P42766

Q9HCK5 P42766

Q9UKV8 P42766

Q9UL18 P42766

Q9Y5S2 P42766

O14641 P42768

O15143 P42768

O43516 P42768

O60861 P42768

O75554 P42768

O94868 P42768

P06241 P42768

P08575 P42768

P08631 P42768

P17081 P42768

P23467 P42768

P23471 P42768

P42768 P42768

P60763 P42768

P60953 P42768

P68400 P42768

Q05209 P42768

Q8TF74 P42768

Q8WV41 P42768

Q8WZ75 P42768

Q9H4E5 P42768

Q9P2A4 P42768

Q9Y2R2 P42768

Q9Y5X1 P42768

O00311 P42771

O43913 P42771

O43929 P42771

O75419 P42771

O75496 P42771

P02730 P42771

P02751 P42771

P11802 P42771

P12004 P42771

P17252 P42771

P33992 P42771

Q00534 P42771

Q00839 P42771

Q14566 P42771

Q6UXH1 P42771

Q7L590 P42771

Q8NHU6 P42771

Q99741 P42771

O95163 P42772

P11802 P42772

P43364 P42772

Q00534 P42772

Q00987 P42772

Q9GZT6 P42772

Q9P2H0 P42772

O60383 P42773

P04637 P42773

P11802 P42773

P51693 P42773

Q00534 P42773

Q04724 P42773

Q13432 P42773

Q14919 P42773

Q5RL73 P42773

Q5UIP0 P42773

Q7L5N1 P42773

Q9GZT6 P42773

Q9Y6Y9 P42773

O60883 P42785

P78362 P42857

Q96SB4 P42857

G3V1X1 P42858

O00268 P42858

O00291 P42858

O00746 P42858

O14545 P42858

O14645 P42858

O14776 P42858

O43312 P42858

O75400 P42858

O75409 P42858

O75410 P42858

O75781 P42858

O75925 P42858

O95163 P42858

O95243 P42858

P02792 P42858

P04637 P42858

P08047 P42858

P09017 P42858

P35080 P42858

P37840 P42858

P42858 P42858

P43268 P42858

P50221 P42858

P61086 P42858

Q13011 P42858

Q13177 P42858

Q13503 P42858

Q14596 P42858

Q2NKX8 P42858

Q5W5X9 P42858

Q6IAI7 P42858

Q8IUH5 P42858

Q8IY31 P42858

Q8IZQ1 P42858

Q8N2W9 P42858

Q8N7X4 P42858

Q8WTP9 P42858

Q8WYH8 P42858

Q92793 P42858

Q92882 P42858

Q969T4 P42858

Q96CV9 P42858

Q96D09 P42858

Q96DZ7 P42858

Q96EE4 P42858

Q96HT8 P42858

Q96K21 P42858

Q96KD3 P42858

Q96M61 P42858

Q96NC0 P42858

Q99689 P42858

Q99963 P42858

Q9BSL1 P42858

Q9BVJ6 P42858

Q9BVL2 P42858

Q9H1J1 P42858

Q9H1Y0 P42858

Q9H4A5 P42858

Q9P2H0 P42858

Q9UI08 P42858

Q9UIS9 P42858

Q9UJ41 P42858

Q9UJY4 P42858

Q9UK73 P42858

Q9Y2X7 P42858

Q9Y3C7 P42858

Q9Y605 P42858

P10636 P43004

O00238 P43026

Q15051 P43034

Q9GZM8 P43034

Q9NRI5 P43034

P21926 P43121

P02768 P43146

P18084 P43146

P46777 P43146

P56270 P43146

Q9HD67 P43146

O00716 P43243

O14686 P43243

O15550 P43243

O75509 P43243

O95166 P43243

O95758 P43243

P00533 P43243

P09651 P43243

P09913 P43243

P16104 P43243

P19320 P43243

P22736 P43243

P33993 P43243

P38919 P43243

P60520 P43243

P61326 P43243

P62993 P43243

Q00005 P43243

Q13573 P43243

Q5S007 P43243

Q6ZW49 P43243

Q99459 P43243

Q9GZQ8 P43243

Q9H0R8 P43243

Q9H492 P43243

Q9NRI5 P43243

Q9UKA9 P43243

Q9UKV8 P43243

Q9UL18 P43243

O00716 P43246

P01106 P43246

P20073 P43246

P20585 P43246

P38398 P43246

P43246 P43246

P52701 P43246

Q13573 P43246

Q8IY92 P43246

Q92830 P43246

Q96FW1 P43246

Q99459 P43246

Q9UQ84 P43246

P08648 P43250

P43250 P43250

O00716 P43304

O60832 P43307

O75365 P43307

P00533 P43307

P01009 P43307

P01106 P43307

P04626 P43307

P08697 P43307

P21246 P43307

P21860 P43307

P27824 P43307

P51571 P43307

P51858 P43307

P61619 P43307

P68104 P43307

Q13418 P43307

Q92731 P43307

Q9BVJ6 P43307

Q9UL18 P43307

P02489 P43320

P02511 P43320

P05813 P43320

P07315 P43320

P43320 P43320

P53673 P43320

P35244 P43351

P43351 P43351

Q14191 P43351

O60313 P43354

P07197 P43354

P62826 P43354

P78347 P43354

Q5H9L2 P43354

Q9BTT0 P43354

P14927 P43355

Q13573 P43355

Q8N140 P43355

Q9BZY9 P43355

Q9NQG6 P43355

Q9NR46 P43355

Q9NXX6 P43355

O15379 P43356

P04637 P43356

P62256 P43356

Q13263 P43356

Q13263 P43357

P14927 P43358

Q13105 P43358

Q86WT6 P43358

Q8WW24 P43358

O14530 P43360

O75688 P43360

O94925 P43360

P04792 P43360

P04844 P43360

P09661 P43360

P28340 P43360

P35606 P43360

P39656 P43360

P42704 P43360

P78344 P43360

P78347 P43360

Q00341 P43360

Q04637 P43360

Q13263 P43360

Q13547 P43360

Q13617 P43360

Q14203 P43360

Q15042 P43360

Q16891 P43360

Q6NXE6 P43360

Q8N163 P43360

Q92797 P43360

Q96A33 P43360

Q96DI7 P43360

Q9BWH6 P43360

Q9H993 P43360

Q9HAV4 P43360

Q9NRZ9 P43360

Q9UBB4 P43360

Q9UGP8 P43360

Q9Y277 P43360

Q9Y3A5 P43360

Q9Y5M8 P43360

Q9Y5Q9 P43360

Q9Y679 P43360

O14548 P43364

O14832 P43364

O94818 P43364

P14373 P43364

P30048 P43364

P42330 P43364

P58340 P43364

Q05516 P43364

Q15007 P43364

Q16206 P43364

Q49A88 P43364

Q52LW3 P43364

Q68CQ4 P43364

Q6ZU52 P43364

Q7Z614 P43364

Q8IYD9 P43364

Q8N131 P43364

Q8N490 P43364

Q8N4C5 P43364

Q8N715 P43364

Q8N720 P43364

Q8NDB6 P43364

Q8NEG5 P43364

Q8TAP6 P43364

Q8TBB1 P43364

Q8TDX5 P43364

Q96AJ1 P43364

Q96CB9 P43364

Q96DC9 P43364

Q96EZ8 P43364

Q96JH8 P43364

Q96LM6 P43364

Q96T60 P43364

Q9BQ70 P43364

Q9BQP7 P43364

Q9BSJ1 P43364

Q9BW11 P43364

Q9BXY8 P43364

Q9GZM8 P43364

Q9GZT8 P43364

Q9H204 P43364

Q9H788 P43364

Q9H875 P43364

Q9NP79 P43364

Q9NP98 P43364

Q9NXH9 P43364

Q9UNH6 P43364

Q9Y2S0 P43364

Q9Y2X8 P43364

Q9Y375 P43364

Q9Y6M9 P43364

P42229 P43365

Q96SB4 P43366

O95197 P43378

P46459 P43378

O43561 P43403

O95169 P43403

P00533 P43403

P06239 P43403

P07766 P43403

P07900 P43403

P15498 P43403

P20963 P43403

P21860 P43403

Q9Y2R2 P43403

O00241 P43405

O00459 P43405

P00533 P43405

P04626 P43405

P05107 P43405

P06239 P43405

P07766 P43405

P09769 P43405

P11049 P43405

P12318 P43405

P19174 P43405

P19320 P43405

P20273 P43405

P21145 P43405

P21860 P43405

P22681 P43405

P26038 P43405

P27986 P43405

P30273 P43405

P52735 P43405

P78314 P43405

Q15303 P43405

Q16633 P43405

Q8WV28 P43405

P19320 P43487

P62826 P43487

Q5S007 P43487

P02792 P43490

P03886 P43490

Q01628 P43490

Q9BW61 P43490

O00231 P43686

O75832 P43686

O95166 P43686

O95816 P43686

P01106 P43686

P17980 P43686

P22234 P43686

P35998 P43686

P51665 P43686

P54578 P43686

P55036 P43686

P62191 P43686

P62195 P43686

P62333 P43686

Q13200 P43686

Q13573 P43686

Q14525 P43686

Q16401 P43686

Q8N3K9 P43686

Q96DT6 P43686

Q99459 P43686

Q99460 P43686

Q9NWV8 P43686

Q9UNM6 P43686

Q9Y5K5 P43686

P49917 P43897

O00401 P45378

O75386 P45378

P54257 P45378

Q8N3C7 P45378

P45381 P45381

P45844 P45844

P15289 P45877

Q9NRR5 P45877

P08473 P45880

P15336 P45880

P15735 P45880

P19320 P45880

P27824 P45880

P37840 P45880

P60709 P45880

Q13268 P45880

Q13286 P45880

Q96A26 P45880

Q9H0R8 P45880

Q9H845 P45880

O94966 P45954

O95347 P45973

P23497 P45973

P45973 P45973

P52565 P45973

P68431 P45973

P83916 P45973

Q13111 P45973

Q13185 P45973

Q14739 P45973

Q15369 P45973

Q7Z3K3 P45973

Q8IYF1 P45973

Q9BWN1 P45973

Q9BZ95 P45973

Q9NQS7 P45973

Q9UIS9 P45973

P0CG48 P45974

P54727 P45974

Q6URH7 P45974

Q9Y3C5 P45974

O14733 P45983

O43318 P45983

O75369 P45983

P05387 P45983

P05412 P45983

P10415 P45983

P11142 P45983

P15822 P45983

P22681 P45983

P41250 P45983

P45984 P45983

P46109 P45983

P49023 P45983

P62714 P45983

P98082 P45983

Q02363 P45983

Q07817 P45983

Q13115 P45983

Q13202 P45983

Q6ZUT1 P45983

Q86Y07 P45983

Q92934 P45983

Q96PV7 P45983

Q9HBH9 P45983

Q9NP77 P45983

Q9NZC7 P45983

Q9P219 P45983

Q9UQF2 P45983

P15336 P45983

O43379 P45984

O75896 P45984

P10909 P45984

P13686 P45984

P24278 P45984

P28562 P45984

P40763 P45984

P45985 P45984

P62714 P45984

Q15427 P45984

Q8N448 P45984

Q9C0F1 P45984

Q9NQW7 P45984

Q9P2H0 P45984

Q9UIA9 P45984

P05412 P45984

P15336 P45984

P32121 P45985

P45983 P45985

P49407 P45985

Q13233 P45985

Q8WV28 P45985

Q99459 P45985

O00257 P46013

O95758 P46013

P00519 P46013

P03372 P46013

P04183 P46013

P11387 P46013

Q5S007 P46013

Q92731 P46013

Q9BYG3 P46013

Q9NS87 P46013

Q9UKV0 P46013

Q00005 P46013

O15198 P46019

P15735 P46019

P32121 P46019

P10276 P46020

P15735 P46020

A0JLT2 P46060

P13501 P46060

P30480 P46060

P32121 P46060

P55854 P46060

P63096 P46060

P63165 P46060

P63279 P46060

Q13573 P46060

Q8IY92 P46060

Q99459 P46060

Q9NWV8 P46060

Q9UBE8 P46060

Q9UBU9 P46060

Q8IW19 P46063

O95166 P46087

P03372 P46087

P11387 P46087

P19525 P46087

P33993 P46087

P51858 P46087

P60520 P46087

P78362 P46087

Q96DB2 P46087

Q9HC52 P46087

Q9UKV0 P46087

Q9UPE1 P46087

O00488 P46089

Q9Y2B1 P46089

P21246 P46100

P29074 P46100

P84243 P46100

Q13573 P46100

Q14240 P46100

Q96PY6 P46100

Q9H7U1 P46100

Q9UER7 P46100

Q9Y383 P46100

A9UF07 P46108

O00254 P46108

O00401 P46108

O00459 P46108

O14490 P46108

O14654 P46108

O15117 P46108

O15162 P46108

O15360 P46108

O15399 P46108

O43281 P46108

O60885 P46108

O75167 P46108

O76081 P46108

O94856 P46108

O95157 P46108

P00519 P46108

P00533 P46108

P04626 P46108

P09619 P46108

P10301 P46108

P10912 P46108

P12931 P46108

P15498 P46108

P15918 P46108

P16234 P46108

P16383 P46108

P17542 P46108

P21333 P46108

P21860 P46108

P22681 P46108

P26992 P46108

P29074 P46108

P29353 P46108

P31994 P46108

P35326 P46108

P35968 P46108

P42566 P46108

P42684 P46108

P43403 P46108

P43699 P46108

P45983 P46108

P49802 P46108

P51813 P46108

P54253 P46108

P56945 P46108

P62333 P46108

P78345 P46108

P80404 P46108

Q05397 P46108

Q07890 P46108

Q07912 P46108

Q13087 P46108

Q13905 P46108

Q14185 P46108

Q14296 P46108

Q7L190 P46108

Q7Z408 P46108

Q86Y01 P46108

Q8IZP0 P46108

Q8ND30 P46108

Q8TF42 P46108

Q92918 P46108

Q96CW1 P46108

Q9BXM0 P46108

Q9C0E4 P46108

Q9H1R2 P46108

Q9HCM9 P46108

Q9HCU4 P46108

Q9NPY3 P46108

Q9NQ76 P46108

Q9UBM4 P46108

Q9UBS5 P46108

Q9UK85 P46108

Q9ULH1 P46108

Q9UN72 P46108

Q9UNH6 P46108

Q9Y2J2 P46108

Q9Y4K4 P46108

Q9Y5H9 P46108

Q9Y5I1 P46108

Q9Y5I2 P46108

Q9Y5I3 P46108

P08069 P46108

O15117 P46109

O75095 P46109

P00519 P46109

P00533 P46109

P09619 P46109

P16234 P46109

P17948 P46109

P19320 P46109

P22681 P46109

P42229 P46109

P62899 P46109

Q05397 P46109

Q06124 P46109

Q07666 P46109

Q12774 P46109

Q13191 P46109

Q13323 P46109

Q13480 P46109

Q13905 P46109

Q15303 P46109

Q68DK7 P46109

Q8IXK0 P46109

Q8ND30 P46109

Q92918 P46109

Q93009 P46109

Q96PD2 P46109

Q9BYB0 P46109

Q9H0R8 P46109

Q9Y4K4 P46109

Q14197 P46199

O43765 P46379

O75400 P46379

O95163 P46379

O95405 P46379

O95967 P46379

P02745 P46379

P02751 P46379

P04637 P46379

P05155 P46379

P10909 P46379

P11441 P46379

P18428 P46379

P20073 P46379

P21246 P46379

P25445 P46379

P34741 P46379

P36896 P46379

P46695 P46379

P46736 P46379

P55345 P46379

Q07002 P46379

Q14257 P46379

Q15047 P46379

Q16891 P46379

Q53G59 P46379

Q5TF85 P46379

Q7Z434 P46379

Q92995 P46379

Q96S82 P46379

Q9BV68 P46379

Q9NPY3 P46379

Q9NRR5 P46379

P36873 P46439

O75899 P46459

O95249 P46459

P05067 P46459

P05386 P46459

P08575 P46459

P23467 P46459

P23470 P46459

P42262 P46459

P46459 P46459

P54920 P46459

P60228 P46459

Q13573 P46459

Q14457 P46459

Q15262 P46459

Q969Q5 P46459

Q99459 P46459

Q9BWH2 P46459

Q9NWV8 P46459

Q9P2H0 P46459

Q9UBS5 P46459

Q9UKR5 P46459

Q9Y383 P46459

Q9Y478 P46459

O00505 P46527

O15111 P46527

O15131 P46527

O60684 P46527

O96020 P46527

P06493 P46527

P07948 P46527

P10914 P46527

P11802 P46527

P14635 P46527

P16949 P46527

P20248 P46527

P24385 P46527

P24864 P46527

P24941 P46527

P27348 P46527

P31749 P46527

P31946 P46527

P33993 P46527

P52292 P46527

P62993 P46527

P78396 P46527

Q00526 P46527

Q00535 P46527

Q12933 P46527

Q13309 P46527

Q92905 P46527

O00555 P46531

P12956 P46531

P19838 P46531

P78504 P46531

P98170 P46531

Q04864 P46531

Q06330 P46531

Q13526 P46531

Q13573 P46531

Q92585 P46531

P46597 P46597

P01042 P46663

P28482 P46695

Q07820 P46695

Q13362 P46695

P26373 P46734

Q13573 P46734

Q16512 P46734

Q16539 P46734

Q5S007 P46734

Q99683 P46734

Q9Y463 P46734

Q93009 P46736

Q96RL1 P46736

Q9NWV8 P46736

Q9NXR7 P46736

Q9P2Y5 P46736

P02751 P46776

P03372 P46776

P19320 P46776

P22694 P46776

P62993 P46776

Q00987 P46776

Q13608 P46776

Q9HCK5 P46776

Q9UKV8 P46776

Q9UL18 P46776

O95166 P46777

P02751 P46777

P03372 P46777

P04637 P46777

P08238 P46777

P19320 P46777

P43146 P46777

P45973 P46777

P60520 P46777

P62266 P46777

P67870 P46777

P68400 P46777

Q00987 P46777

Q15527 P46777

Q15843 P46777

Q9H0R8 P46777

Q9H492 P46777

O95166 P46778

O95793 P46778

P02751 P46778

P03372 P46778

P04179 P46778

P09651 P46778

P19320 P46778

P32121 P46778

P60520 P46778

P61326 P46778

P62993 P46778

Q00005 P46778

Q15605 P46778

Q15843 P46778

Q9GZQ8 P46778

Q9H0R8 P46778

Q9H492 P46778

Q9P1W9 P46778

Q9UKV8 P46778

Q9UL18 P46778

Q9UQ80 P46778

O95166 P46779

O95793 P46779

P02751 P46779

P03372 P46779

P19320 P46779

P32121 P46779

P38919 P46779

P43146 P46779

P51668 P46779

P60520 P46779

P61077 P46779

P61326 P46779

Q13485 P46779

Q9UKV8 P46779

Q9UL18 P46779

Q9UQ80 P46779

O95166 P46781

P03372 P46781

P19320 P46781

P38919 P46781

P40763 P46781

P60520 P46781

P60709 P46781

P61326 P46781

P62993 P46781

Q5U5Q3 P46781

Q92597 P46781

Q92731 P46781

Q9H0R8 P46781

Q9H492 P46781

Q9HCK5 P46781

Q9NY93 P46781

Q9UKV8 P46781

Q9UL18 P46781

O95166 P46782

P19320 P46782

P40692 P46782

P60520 P46782

P62993 P46782

Q5U5Q3 P46782

Q9H0R8 P46782

Q9HCK5 P46782

Q9UKV8 P46782

Q9UL18 P46782

O00291 P46783

O95166 P46783

O95251 P46783

O95758 P46783

P02751 P46783

P08473 P46783

P16104 P46783

P19320 P46783

P35080 P46783

P43146 P46783

P43351 P46783

P54257 P46783

P60520 P46783

P60709 P46783

Q5U5Q3 P46783

Q9H0R8 P46783

Q9UBC3 P46783

O94817 P46821

P04637 P46821

P24386 P46821

P29692 P46821

P32121 P46821

P49407 P46821

P60520 P46821

P61457 P46821

P62136 P46821

Q13043 P46821

Q13188 P46821

Q15051 P46821

Q8WWW0 P46821

Q9C0C7 P46821

Q9GZQ8 P46821

Q9H0Y0 P46821

Q9H2C0 P46821

Q9H492 P46821

Q9NT62 P46821

Q9P0W5 P46821

Q9UGJ0 P46821

O14964 P46934

O15350 P46934

O95166 P46934

O95400 P46934

P0CG48 P46934

P11362 P46934

P32121 P46934

P37088 P46934

P51168 P46934

P51170 P46934

P60484 P46934

P60520 P46934

Q16655 P46934

Q5TBB8 P46934

Q99717 P46934

Q99732 P46934

Q9GZQ8 P46934

Q9H0R8 P46934

Q9H469 P46934

Q9H492 P46934

O14745 P46937

O15350 P46937

P07947 P46937

P27348 P46937

P28347 P46937

P31946 P46937

P35222 P46937

P61981 P46937

Q13625 P46937

Q15797 P46937

Q4VCS5 P46937

Q99593 P46937

Q9H204 P46937

Q9UKB1 P46937

O60941 P46939

O95477 P46939

P08473 P46939

P0C862 P46939

P48736 P46939

Q13425 P46939

Q13884 P46939

Q14118 P46939

Q6PJG9 P46939

Q7KZI7 P46939

Q9NRI5 P46939

Q9UK80 P46939

Q9Y4J8 P46939

O00592 P46940

O15264 P46940

O43707 P46940

O60500 P46940

O60610 P46940

O75381 P46940

O95793 P46940

P00533 P46940

P02751 P46940

P03372 P46940

P04626 P46940

P19320 P46940

P29353 P46940

P35222 P46940

P46937 P46940

P60953 P46940

P62068 P46940

P62993 P46940

P63104 P46940

Q00005 P46940

Q05823 P46940

Q13469 P46940

Q13573 P46940

Q16539 P46940

Q5S007 P46940

Q5U5Q3 P46940

Q8WVZ9 P46940

Q96QZ7 P46940

Q99459 P46940

Q99816 P46940

Q9NP85 P46940

Q9Y5K6 P46940

A0AUL9 P46976

O43741 P46976

O95166 P46976

P13807 P46976

P54646 P46976

Q96HA1 P46976

Q9BQ75 P46976

Q9H0R8 P46976

Q9P2Y5 P46976

O00716 P46977

O43187 P46977

Q86WV6 P46977

O43504 P47224

P61006 P47224

Q9BVJ6 P47224

Q92876 P47710

P01106 P47712

P35080 P47736

P61224 P47736

P63104 P47736

Q16659 P47736

Q6ZN04 P47736

Q9GZQ8 P47736

P25054 P47755

P32121 P47755

P02751 P47756

P03372 P47756

P19320 P47756

P21246 P47756

P60520 P47756

P62993 P47756

Q09019 P47756

Q96RL1 P47756

Q9BYC9 P47756

Q9GZQ8 P47756

Q9H0R8 P47756

Q9P287 P47756

Q00005 P47756

Q96EK5 P47804

O94829 P47813

P55010 P47813

Q00987 P47869

O96015 P47872

P62158 P47872

P02768 P47893

O75131 P47897

P02751 P47897

P02766 P47897

P16104 P47897

P19320 P47897

P20073 P47897

Q13155 P47897

Q13188 P47897

Q7L190 P47897

Q9Y3C5 P47897

Q9Y6H6 P47897

P02751 P47914

P03372 P47914

Q12873 P47914

Q15843 P47914

Q92731 P47914

Q9NX95 P47914

Q9NY93 P47914

Q9UKV8 P47914

Q9UL18 P47914

P12931 P47928

P16333 P47928

P19174 P47928

P46108 P47928

O94782 P47929

P01106 P47929

P03372 P47929

P47929 P47929

Q5MNZ9 P47929

Q8NB14 P47929

Q9H0R8 P47929

Q9Y4E8 P47929

P01106 P47972

Q04917 P47974

P01106 P47985

P08574 P47985

P09669 P47985

P31930 P47985

P35711 P47985

Q01201 P47985

Q14197 P47985

P01106 P47989

A1X283 P48023

O43586 P48023

O43639 P48023

O60504 P48023

O95049 P48023

P02549 P48023

P06241 P48023

P14598 P48023

P16333 P48023

P25445 P48023

P27986 P48023

P42680 P48023

P46108 P48023

P54284 P48023

P98171 P48023

Q06187 P48023

Q08881 P48023

Q13158 P48023

Q14790 P48023

Q15642 P48023

Q15700 P48023

Q16674 P48023

Q5TCZ1 P48023

Q7Z6B7 P48023

Q8WV41 P48023

Q96RU3 P48023

Q9BY11 P48023

Q9NZM3 P48023

Q9UHR4 P48023

Q9UKS6 P48023

Q9UNF0 P48023

Q9Y5X1 P48023

O76081 P48039

P49286 P48039

P01106 P48047

P15336 P48047

P19320 P48047

P46736 P48047

Q8IY92 P48047

Q9H0R8 P48047

Q9H845 P48047

Q9HAP6 P48050

O95758 P48058

P17612 P48058

O43639 P48059

O60711 P48059

P01106 P48059

P20929 P48059

P50461 P48059

P51784 P48059

Q13418 P48059

Q13573 P48059

Q15404 P48059

Q15831 P48059

Q5VZL5 P48059

Q8N2R0 P48059

Q8WZ42 P48059

Q96KQ7 P48059

Q99459 P48059

Q99708 P48059

Q9GZU2 P48059

Q9NS73 P48059

Q9NVD7 P48059

Q9P212 P48059

Q6V1X1 P48061

Q14160 P48065

P01857 P48067

Q70EK8 P48200

P46379 P48304

P08575 P48357

P62993 P48357

Q12913 P48357

Q13526 P48357

Q8HWS3 P48378

Q12933 P48380

Q8HWS3 P48380

Q92769 P48382

Q99459 P48382

P19784 P48426

Q9Y2Q3 P48426

Q9UI12 P48436

P05060 P48443

P10276 P48443

P27707 P48443

Q9NRX1 P48443

O95758 P48444

P19320 P48444

P49755 P48444

Q13573 P48444

Q14145 P48444

Q504Q3 P48444

Q99459 P48444

Q9BVK6 P48444

Q9NTJ5 P48444

O00165 P48454

P67870 P48454

Q96DU7 P48454

Q9BT49 P48454

P62158 P48507

Q6ZVD7 P48507

Q92985 P48507

Q15051 P48539

P01563 P48551

P05000 P48551

P17181 P48551

P23458 P48551

P52630 P48551

P63244 P48551

Q00978 P48551

Q09472 P48551

Q86XI6 P48551

Q92793 P48551

Q9UMW8 P48551

O75386 P48552

O95751 P48552

P03372 P48552

P26358 P48552

Q13363 P48552

Q13642 P48552

Q15834 P48552

Q8IYF3 P48552

Q8N895 P48552

Q8NHQ1 P48552

Q92731 P48552

Q99873 P48552

Q96QF0 P48553

O00487 P48556

P51665 P48556

P54578 P48556

Q16401 P48556

Q9Y5K5 P48556

P00533 P48594

P03372 P48594

Q8IY92 P48594

Q9H0R8 P48594

Q9Y297 P48594

Q96SB4 P48595

O15372 P48634

O15460 P48634

P01106 P48634

P02671 P48634

P05412 P48634

P09651 P48634

P25788 P48634

P46092 P48634

P47897 P48634

P52272 P48634

P55345 P48634

P60228 P48634

P62993 P48634

Q07021 P48634

Q10570 P48634

Q13099 P48634

Q13137 P48634

Q14157 P48634

Q14697 P48634

Q16891 P48634

Q9NTJ4 P48634

Q9UHI6 P48634

Q9UI95 P48634

Q9UNL2 P48634

P48637 P48637

Q14653 P48637

O15264 P48643

O15379 P48643

O75381 P48643

O95163 P48643

P01106 P48643

P02751 P48643

P04637 P48643

P15336 P48643

P16104 P48643

P17987 P48643

P19320 P48643

P60510 P48643

P62714 P48643

P67775 P48643

P78318 P48643

Q00005 P48643

Q00535 P48643

Q13033 P48643

Q13418 P48643

Q13573 P48643

Q15257 P48643

Q16891 P48643

Q5T4F4 P48643

Q66LE6 P48643

Q8WZ74 P48643

Q99459 P48643

Q9H492 P48643

Q9H7D0 P48643

Q9HA65 P48643

Q9HCK5 P48643

Q9NPJ1 P48643

Q9NRL3 P48643

Q9Y228 P48643

Q9Y2T4 P48643

Q9Y3A3 P48643

Q9Y6E0 P48643

O95376 P48664

O95166 P48668

P60520 P48668

Q9H0R8 P48668

Q9H3D4 P48668

P43307 P48723

P46379 P48723

Q9H9A7 P48723

Q9NR33 P48723

Q9NRR5 P48723

Q9UMX0 P48723

O15151 P48729

O15169 P48729

O75689 P48729

O95758 P48729

P07900 P48729

P08238 P48729

P09429 P48729

P18754 P48729

P19320 P48729

P26583 P48729

P50548 P48729

P52294 P48729

Q00987 P48729

Q04917 P48729

Q13546 P48729

Q16625 P48729

Q96A00 P48729

Q9BZL6 P48729

O95758 P48730

P48729 P48730

P49674 P48730

Q00987 P48730

Q8IYT8 P48730

Q92997 P48730

Q96RY5 P48730

P84022 P48730

Q15796 P48730

Q13547 P48735

P01112 P48736

P10276 P48736

P48736 P48736

P54646 P48736

Q13370 P48736

Q70EL2 P48736

Q9H0R8 P48736

P61968 P48739

O43639 P48740

O43809 P48740

P16104 P48740

P21860 P48740

P24522 P48740

P50613 P48740

Q13387 P48740

Q16543 P48740

Q16659 P48740

Q96CW1 P48740

Q9UJM3 P48740

Q9UK32 P48740

O95166 P48741

P13569 P48741

P49407 P48741

P60520 P48741

Q92905 P48741

Q9GZQ8 P48741

Q9H0R8 P48741

Q9H492 P48741

P30566 P48745

O60341 P48775

O95671 P48775

Q96LA8 P48775

P12931 P48960

P28340 P49005

Q15054 P49005

Q8TD31 P49005

Q96S55 P49005

Q9HCU8 P49005

Q16539 P49006

P00533 P49023

P02751 P49023

P05556 P49023

P13612 P49023

P19320 P49023

P21399 P49023

P22102 P49023

P23470 P49023

P23471 P49023

P23526 P49023

P25054 P49023

P35222 P49023

P46108 P49023

P46109 P49023

Q05397 P49023

Q06124 P49023

Q12913 P49023

Q13418 P49023

Q13480 P49023

Q16854 P49023

Q70SY1 P49023

Q99942 P49023

Q9NVD7 P49023

Q9ULH1 P49023

Q9Y2S0 P49023

Q9Y2X7 P49023

O14836 P49069

P46695 P49069

O60341 P49116

Q13573 P49116

Q9Y5K6 P49116

P03372 P49137

P04792 P49137

P30304 P49137

P53667 P49137

Q00613 P49137

Q15759 P49137

Q16539 P49137

P10082 P49146

P62068 P49189

Q96KP6 P49189

Q9BW61 P49189

Q9H0R8 P49189

P62158 P49190

Q9UBQ7 P49190

O43293 P49207

P51858 P49207

P60520 P49207

P61244 P49207

Q9NY93 P49207

P01857 P49221

P01876 P49221

P49247 P49247

P51116 P49247

Q06124 P49247

Q8TBB1 P49247

Q96HA8 P49247

Q9GZT8 P49247

Q9H8Y8 P49247

P17706 P49257

Q9BS26 P49257

Q9Y3A6 P49257

P48039 P49286

O15264 P49321

P62805 P49321

P68431 P49321

Q71DI3 P49321

Q9UBN7 P49321

Q9Y383 P49321

O15264 P49327

O95166 P49327

P01857 P49327

P07900 P49327

P08238 P49327

P15735 P49327

P19320 P49327

P32121 P49327

P49407 P49327

P63104 P49327

Q13418 P49327

Q13573 P49327

Q63ZY3 P49327

Q6PJG9 P49327

Q99459 P49327

Q9GZQ8 P49327

Q9H0R8 P49327

Q9H3D4 P49327

Q9H492 P49327

A0JLT2 P49336

O95402 P49336

P24863 P49336

P36956 P49336

Q12772 P49336

Q15797 P49336

Q9BTT4 P49336

Q9BWU1 P49336

Q9NWA0 P49336

Q9NX70 P49336

Q9Y4A5 P49336

P09529 P49354

P21980 P49354

P49356 P49354

P52630 P49354

P52735 P49354

Q13627 P49354

P49354 P49356

Q13627 P49356

P27361 P49366

P32969 P49366

P62256 P49366

Q6ZVK8 P49366

Q9GZT8 P49366

Q9GZV4 P49366

O15264 P49368

O15379 P49368

O60232 P49368

P01106 P49368

P04637 P49368

P15336 P49368

P17987 P49368

P19320 P49368

P49770 P49368

P60510 P49368

P60520 P49368

P62714 P49368

P67775 P49368

P78318 P49368

Q00005 P49368

Q00535 P49368

Q13033 P49368

Q13151 P49368

Q13418 P49368

Q13573 P49368

Q15257 P49368

Q16659 P49368

Q5T4F4 P49368

Q66LE6 P49368

Q676U5 P49368

Q8IWZ6 P49368

Q8TAM1 P49368

Q8WZ74 P49368

Q96DB2 P49368

Q99459 P49368

Q9GZQ8 P49368

Q9H0R8 P49368

Q9H7D0 P49368

Q9H9G7 P49368

Q9NPJ1 P49368

Q9NRL3 P49368

Q9NWA0 P49368

Q9NX70 P49368

Q9UJM3 P49368

Q9Y228 P49368

Q9Y2T4 P49368

Q9Y3A3 P49368

Q9Y6E0 P49368

O60732 P49406

P19320 P49406

P67809 P49406

Q14197 P49406

Q969Q1 P49406

O00203 P49407

O00459 P49407

O00505 P49407

O00541 P49407

O14964 P49407

O43298 P49407

O95218 P49407

P04731 P49407

P05412 P49407

P06396 P49407

P08069 P49407

P19338 P49407

P23528 P49407

P25490 P49407

P27348 P49407

P32121 P49407

P35813 P49407

P36575 P49407

P45984 P49407

P49407 P49407

P50053 P49407

P50148 P49407

P53779 P49407

P60953 P49407

P62158 P49407

P68400 P49407

Q06187 P49407

Q13428 P49407

Q13523 P49407

Q14137 P49407

Q14749 P49407

Q14978 P49407

Q16627 P49407

Q5JWF2 P49407

Q6DKI1 P49407

Q7Z4V5 P49407

Q92541 P49407

Q96DG6 P49407

Q96GQ7 P49407

Q99683 P49407

Q99933 P49407

Q9HC98 P49407

Q9Y4K3 P49407

O95166 P49411

P00533 P49411

P01106 P49411

P04626 P49411

P13489 P49411

P19320 P49411

P20333 P49411

P21860 P49411

P60520 P49411

P62993 P49411

Q00005 P49411

Q13573 P49411

Q14197 P49411

Q7L0Y3 P49411

Q9GZQ8 P49411

Q9H492 P49411

Q9HCK5 P49411

Q9UQ16 P49418

Q12929 P49419

Q9BW61 P49419

Q9GZZ9 P49419

P28562 P49427

P68400 P49427

Q13616 P49427

Q8ND25 P49427

Q9Y297 P49427

Q9Y5L4 P49427

P18858 P49441

O75674 P49448

P01106 P49448

P15336 P49448

Q7L0Y3 P49448

P00519 P49450

P06241 P49450

P62805 P49450

Q13111 P49450

Q8NCD3 P49450

O00257 P49454

P32121 P49454

P43034 P49454

Q02224 P49454

Q5S007 P49454

Q9GZM8 P49454

Q9NXR1 P49454

O00418 P49458

P01106 P49458

P19320 P49458

P62993 P49458

Q6MZP7 P49458

Q9H492 P49458

Q5VTR2 P49459

Q8IWV7 P49459

Q9NS91 P49459

Q9NVF9 P49459

P02751 P49588

P08238 P49588

P19320 P49588

P63104 P49588

P01375 P49589

Q14197 P49590

P01106 P49591

P19320 P49591

Q9GZZ9 P49591

P54646 P49593

Q13131 P49593

Q9UK73 P49593

Q9UQM7 P49593

P02768 P49619

P49407 P49619

O15162 P49639

O43463 P49639

O60711 P49639

O95967 P49639

P04156 P49639

P27986 P49639

P49639 P49639

Q15654 P49639

Q99750 P49639

Q9BQ66 P49639

O95163 P49641

P08670 P49641

P13929 P49641

Q9Y2X7 P49641

Q9Y383 P49641

P09884 P49642

P24386 P49642

Q14181 P49642

O43463 P49643

P09884 P49643

P24386 P49643

P27694 P49643

Q14181 P49643

O14579 P49662

O75643 P49662

O95347 P49662

P06396 P49662

P09661 P49662

P11172 P49662

P20618 P49662

P21796 P49662

P24941 P49662

P41091 P49662

P51571 P49662

P52701 P49662

P53007 P49662

Q04323 P49662

Q14203 P49662

Q14683 P49662

Q6UB35 P49662

Q86Y56 P49662

Q8N1F7 P49662

Q8TBZ2 P49662

Q92616 P49662

Q93008 P49662

Q969L4 P49662

Q9BVK8 P49662

Q9H936 P49662

Q9NSA3 P49662

Q9NTJ3 P49662

Q9NU22 P49662

Q9P031 P49662

Q9P035 P49662

Q9UQE7 P49662

Q9Y277 P49662

Q9Y5M8 P49662

O14640 P49674

O14641 P49674

O15055 P49674

O15169 P49674

O95251 P49674

O95758 P49674

P08238 P49674

P09467 P49674

P25054 P49674

P35222 P49674

P48729 P49674

P62877 P49674

Q00987 P49674

Q14469 P49674

Q16625 P49674

Q1W6H9 P49674

Q8NFZ8 P49674

Q92888 P49674

Q9BQ89 P49674

Q9NRX1 P49674

Q9ULU4 P49674

Q9Y620 P49674

Q9Y6Q9 P49674

Q99689 P49675

P07814 P49703

P14314 P49703

P18669 P49703

P31689 P49703

P63162 P49703

Q04724 P49703

Q13432 P49703

Q14232 P49703

Q8IWV7 P49703

Q8N129 P49703

Q92597 P49703

Q9HC35 P49703

Q9UPT6 P49703

Q9BQS6 P49711

Q9HCK8 P49711

P09874 P49715

P47902 P49715

Q07021 P49715

Q13972 P49715

O14818 P49720

P01106 P49720

P02751 P49720

P16104 P49720

P25189 P49720

P25786 P49720

P25787 P49720

P28074 P49720

P40306 P49720

P49721 P49720

P55345 P49720

P60900 P49720

P63104 P49720

Q15051 P49720

Q9BQ83 P49720

Q9Y244 P49720

Q9Y5K5 P49720

O14818 P49721

P02751 P49721

P18283 P49721

P19320 P49721

P20618 P49721

P25786 P49721

P25787 P49721

P28074 P49721

P49720 P49721

P60900 P49721

Q15051 P49721

Q8IZL9 P49721

Q99436 P49721

Q9BQ83 P49721

Q9H0R8 P49721

Q9Y5K5 P49721

O43913 P49736

O95067 P49736

P02751 P49736

P19320 P49736

P25205 P49736

P27695 P49736

P33992 P49736

P33993 P49736

P42771 P49736

P46736 P49736

P54619 P49736

P62701 P49736

P62805 P49736

P68400 P49736

Q00534 P49736

Q09472 P49736

Q12905 P49736

Q13416 P49736

Q14566 P49736

Q96H20 P49736

Q96HA7 P49736

Q99741 P49736

Q9BW61 P49736

Q9GZZ9 P49736

P49746 P49746

P49747 P49747

P08865 P49748

P12830 P49748

P15336 P49748

Q14197 P49748

O00555 P49750

P05060 P49750

P13497 P49750

P36873 P49750

Q70EL2 P49750

Q8TAF3 P49750

Q93009 P49750

Q96DB2 P49750

Q99459 P49750

Q99873 P49750

Q9GZQ8 P49750

Q9H0R8 P49750

Q9H492 P49750

Q92731 P49754

Q9H9C1 P49754

O15260 P49755

O95758 P49755

P15336 P49755

P17706 P49755

P49755 P49755

P49768 P49755

Q03135 P49755

Q15363 P49755

Q92542 P49755

Q9H0R8 P49755

Q9NZ42 P49755

Q9Y3B3 P49755

P00533 P49756

Q00005 P49756

Q14498 P49756

Q15428 P49756

Q8WWY3 P49756

Q92731 P49756

Q9UQ88 P49756

Q8IUQ4 P49757

Q8TBB1 P49757

Q96CW1 P49757

O14744 P49759

P18031 P49759

P78362 P49759

Q8N2W9 P49759

Q9BZ29 P49759

P08238 P49760

P08621 P49760

P18031 P49760

P49761 P49760

O14744 P49761

O43463 P49761

P08238 P49761

P49760 P49761

P78362 P49761

Q16543 P49761

Q9BUV0 P49761

P55854 P49761

O43464 P49768

O75369 P49768

O95551 P49768

P04406 P49768

P05067 P49768

P11172 P49768

P12830 P49768

P14923 P49768

P20336 P49768

P35222 P49768

P49755 P49768

P49810 P49768

P49841 P49768

P50502 P49768

P55809 P49768

Q02410 P49768

Q07817 P49768

Q16543 P49768

Q92542 P49768

Q92947 P49768

Q96QH2 P49768

Q96TC7 P49768

Q99569 P49768

Q9BQ95 P49768

Q9NQB0 P49768

Q9UMX0 P49768

P49768 P49768

Q16795 P49768

Q96BI3 P49768

P19784 P49770

P43146 P49770

Q96LT7 P49770

P35080 P49771

P49773 P49773

Q13042 P49773

P10809 P49789

P22570 P49789

P36406 P49789

P49789 P49789

P61604 P49789

Q12829 P49789

P01106 P49790

P02545 P49790

P03372 P49790

P25054 P49790

P29323 P49790

P49790 P49790

P51784 P49790

Q13573 P49790

Q16539 P49790

Q5S007 P49790

Q8IY92 P49790

Q8WUM0 P49790

Q96DB2 P49790

Q9UBU9 P49790

Q9Y2K6 P49790

O15169 P49792

O60260 P49792

P01106 P49792

P02768 P49792

P04626 P49792

P11388 P49792

P21675 P49792

P33176 P49792

P49792 P49792

P52292 P49792

P60520 P49792

P63279 P49792

Q12802 P49792

Q13573 P49792

Q4LE39 P49792

Q5S007 P49792

Q96RY7 P49792

Q99459 P49792

Q9GZQ8 P49792

Q9UBE8 P49792

Q9UBU9 P49792

Q16659 P49795

P49407 P49796

Q13568 P49796

P08238 P49802

P19174 P49802

P33993 P49802

O75369 P49810

P05067 P49810

P21333 P49810

P30626 P49810

Q07817 P49810

Q14192 P49810

Q14204 P49810

Q53EL6 P49810

Q99828 P49810

Q9BQ95 P49810

Q9UMX0 P49810

Q9Y6B2 P49810

Q8WW43 P49810

Q92542 P49810

Q96BI3 P49810

P31946 P49815

P31947 P49815

P61981 P49815

P62993 P49815

P63104 P49815

Q04917 P49815

Q92574 P49815

Q96EB6 P49815

Q96PY6 P49815

O43678 P49821

Q14197 P49821

A5PL33 P49840

O75398 P49840

O75581 P49840

P05067 P49840

P61981 P49840

Q16584 P49840

Q96G01 P49840

Q9NP79 P49840

O14908 P49841

O15169 P49841

O43194 P49841

O43427 P49841

O60502 P49841

O75165 P49841

O75354 P49841

O75398 P49841

O75533 P49841

O75581 P49841

O95163 P49841

O95758 P49841

O95863 P49841

P01106 P49841

P04198 P49841

P04637 P49841

P05386 P49841

P05452 P49841

P07108 P49841

P08621 P49841

P08670 P49841

P10636 P49841

P13807 P49841

P15880 P49841

P16989 P49841

P17612 P49841

P17844 P49841

P19793 P49841

P21246 P49841

P22492 P49841

P23258 P49841

P26358 P49841

P30876 P49841

P31749 P49841

P31751 P49841

P33176 P49841

P35222 P49841

P35637 P49841

P36873 P49841

P37840 P49841

P41236 P49841

P42345 P49841

P47897 P49841

P48740 P49841

P49841 P49841

P51659 P49841

P52742 P49841

P53396 P49841

P57678 P49841

P61960 P49841

P61968 P49841

P63104 P49841

P67775 P49841

P68104 P49841

Q01201 P49841

Q01844 P49841

Q06330 P49841

Q12873 P49841

Q12933 P49841

Q13163 P49841

Q13541 P49841

Q13794 P49841

Q14134 P49841

Q14147 P49841

Q15459 P49841

Q15828 P49841

Q15847 P49841

Q16637 P49841

Q2T9K0 P49841

Q56A76 P49841

Q6J9G0 P49841

Q6NUN9 P49841

Q6UN15 P49841

Q71U36 P49841

Q75N90 P49841

Q7L266 P49841

Q7Z5J4 P49841

Q86WZ6 P49841

Q8IWV7 P49841

Q8IX07 P49841

Q8N0Z3 P49841

Q8N205 P49841

Q8TDZ2 P49841

Q92945 P49841

Q96G01 P49841

Q96GR2 P49841

Q96QU8 P49841

Q9BTE0 P49841

Q9BYZ4 P49841

Q9BZV1 P49841

Q9H2U1 P49841

Q9H4H8 P49841

Q9H7B2 P49841

Q9HBH7 P49841

Q9NQW7 P49841

Q9NXS2 P49841

Q9NZI8 P49841

Q9NYF0 P49841

Q9P0R6 P49841

Q9P1W9 P49841

Q9UHI6 P49841

Q9UHR5 P49841

Q9UII2 P49841

Q9UKY1 P49841

Q9UNZ2 P49841

Q9Y383 P49841

Q9Y3F4 P49841

Q9Y4X0 P49841

Q9Y600 P49841

Q9Y6E2 P49841

Q9Y6I7 P49841

P62487 P49842

P78395 P49842

Q02447 P49842

Q07864 P49842

Q15427 P49842

Q99808 P49842

O95402 P49848

P20226 P49848

P61964 P49848

P84243 P49848

Q12962 P49848

P49917 P49863

Q96T60 P49863

O00204 P49888

P04637 P49888

P68104 P49888

Q13432 P49888

Q15047 P49888

Q5RL73 P49888

Q5UIP0 P49888

Q7L5N1 P49888

Q8TC92 P49888

Q96ID5 P49888

Q9UKR5 P49888

O15162 P49901

Q9BQ66 P49901

P04637 P49902

P49902 P49902

P51116 P49902

Q6ZVK8 P49902

Q86TA1 P49902

Q9BVI4 P49902

Q9Y5B8 P49902

P32456 P49903

P49903 P49903

Q13432 P49903

Q14194 P49903

Q8N357 P49903

Q96E40 P49903

Q96ID5 P49903

Q9BXL8 P49903

Q9UKR5 P49903

P00533 P49908

Q09472 P49908

O43463 P49910

O95251 P49910

P13994 P49910

P15622 P49910

P57086 P49910

Q15834 P49910

Q86V42 P49910

Q8IYF1 P49910

Q96AF5 P49910

Q9BQ66 P49910

Q9NR12 P49910

P45983 P49913

P08069 P49913

Q9Y3Q8 P49914

P02751 P49915

P19320 P49915

Q15170 P49915

Q93009 P49915

Q99459 P49915

Q9BW61 P49915

Q9GZQ8 P49915

Q9H492 P49915

Q9NWV8 P49915

Q9UGJ0 P49915

P00519 P49916

P19174 P49916

P46108 P49916

Q8IW19 P49916

Q9HC52 P49916

Q8IW19 P49917

Q8TBC5 P49917

Q8WUB8 P49917

Q9H9Q4 P49917

P24941 P49918

Q9NXK8 P49918

O60934 P49959

P09874 P49959

P16104 P49959

P27695 P49959

P51784 P49959

Q12905 P49959

Q13573 P49959

Q14676 P49959

Q92878 P49959

Q96EB6 P49959

Q99459 P49959

Q9BXW9 P49959

P35625 P50052

P31025 P50053

P61626 P50053

Q13557 P50135

P32302 P50148

P49407 P50148

P50897 P50148

Q14254 P50148

P01106 P50213

P20338 P50213

Q09019 P50213

Q9BW61 P50213

P15863 P50221

O00295 P50222

P01282 P50222

P54652 P50222

Q9BQ66 P50222

Q9Y512 P50222

P54257 P50238

Q96AW1 P50238

Q99963 P50238

P00533 P50281

Q9BV57 P50281

P54257 P50336

Q16891 P50336

O15264 P50395

P02751 P50395

P20338 P50395

P23416 P50395

Q15051 P50395

Q969Q5 P50395

O43889 P50402

P03372 P50402

P13569 P50402

P19320 P50402

P35222 P50402

P51858 P50402

Q15051 P50402

Q16659 P50402

Q5S007 P50402

Q7L190 P50402

Q7L5N1 P50402

Q8N7W2 P50402

Q969F0 P50402

Q99962 P50402

Q99963 P50402

Q9BQS8 P50402

Q9HCK5 P50402

Q9NYF8 P50402

Q9UH99 P50402

Q9UKV8 P50402

P06241 P50406

O00299 P50416

O00716 P50416

P11021 P50416

Q92731 P50416

Q96DB2 P50416

Q9NQX5 P50416

P05181 P50440

O60383 P50453

O75400 P50453

P04406 P50453

P04637 P50453

P12956 P50453

P32456 P50453

P68104 P50453

Q04724 P50453

Q13011 P50453

Q13432 P50453

Q13885 P50453

Q14194 P50453

Q15047 P50453

Q5RL73 P50453

Q5UIP0 P50453

Q7L5N1 P50453

Q8IWV7 P50453

Q96ID5 P50453

Q9GZT6 P50453

Q9NPI1 P50453

Q9UKR5 P50453

Q9UKY1 P50453

Q9Y3C7 P50453

Q9Y4G2 P50453

P01106 P50454

P03372 P50454

P15336 P50454

P60520 P50454

P62993 P50454

P67809 P50454

Q15051 P50454

Q6UXH1 P50454

Q8N357 P50454

Q99689 P50454

Q9Y328 P50454

O15273 P50461

P01106 P50479

Q93062 P50479

P01375 P50502

P02751 P50502

P02766 P50502

P02768 P50502

P07900 P50502

P08107 P50502

P29474 P50502

P49768 P50502

P60953 P50502

P61457 P50502

O00623 P50542

O75381 P50542

P38398 P50542

Q8IY92 P50542

Q9NTG7 P50542

P30281 P50549

P78527 P50549

O00308 P50552

O60936 P50552

P02751 P50552

P19320 P50552

P22736 P50552

P63104 P50552

Q13573 P50552

Q15418 P50552

Q92636 P50552

Q96MA1 P50552

Q9C026 P50552

Q9HD67 P50552

P15884 P50553

P15923 P50553

A0JLT2 P50570

O14576 P50570

O75381 P50570

P00519 P50570

P01106 P50570

P04626 P50570

P08631 P50570

P15880 P50570

P19320 P50570

P23284 P50570

P29353 P50570

P29597 P50570

P46108 P50570

P49418 P50570

P51149 P50570

P57060 P50570

P60709 P50570

P62993 P50570

Q05516 P50570

Q12965 P50570

Q13573 P50570

Q13838 P50570

Q14247 P50570

Q14847 P50570

Q14974 P50570

Q15811 P50570

Q6FIF0 P50570

Q8IVF5 P50570

Q8N157 P50570

Q96B97 P50570

Q96RU3 P50570

Q99459 P50570

Q99832 P50570

Q99962 P50570

Q9BX66 P50570

Q9GZT6 P50570

Q9NX70 P50570

Q9UBV8 P50570

Q9UKG1 P50570

Q9UKM7 P50570

Q9UNF0 P50570

Q9Y262 P50570

Q9Y5X1 P50570

P17096 P50579

P49748 P50579

O00220 P50591

O14763 P50591

O14798 P50591

P13686 P50591

P46695 P50591

Q13618 P50591

Q14790 P50591

Q5RL73 P50591

P24941 P50613

P32121 P50613

P51946 P50613

P78362 P50613

Q01094 P50613

Q96SB4 P50613

A5YKK6 P50616

O00571 P50616

P22626 P50616

P31942 P50616

P52597 P50616

P61978 P50616

Q13148 P50616

Q15047 P50616

Q17RY0 P50616

Q8IY67 P50616

Q8NE35 P50616

Q92600 P50616

Q96GD3 P50616

Q96LI5 P50616

Q9C0C2 P50616

Q9H9A5 P50616

Q9UIV1 P50616

O00763 P50747

P50747 P50747

Q96L92 P50747

Q9Y2R4 P50747

O43264 P50748

Q13043 P50749

Q13188 P50749

Q8WWW0 P50749

O00472 P50750

O14936 P50750

O60563 P50750

O94992 P50750

O95402 P50750

P17542 P50750

P35813 P50750

P42568 P50750

P51825 P50750

Q03111 P50750

Q03164 P50750

Q13573 P50750

Q14527 P50750

Q16543 P50750

Q16576 P50750

Q4G0J3 P50750

Q7L2E3 P50750

Q8WXE1 P50750

Q99459 P50750

Q9HAW4 P50750

Q9UHB7 P50750

Q9UPN9 P50750

Q9Y5B9 P50750

Q9Y5X4 P50750

P00519 P50851

P02751 P50851

P62256 P50876

P68036 P50876

Q13404 P50876

P01106 P50897

P19320 P50897

O95166 P50914

O95793 P50914

P02751 P50914

P03372 P50914

P19320 P50914

P32121 P50914

P60520 P50914

Q00005 P50914

Q13118 P50914

Q8WV24 P50914

Q969T4 P50914

Q9H0R8 P50914

Q9H492 P50914

Q9UL18 P50914

O15264 P50990

O15379 P50990

O75381 P50990

P01106 P50990

P04637 P50990

P17987 P50990

P19320 P50990

P60510 P50990

P62714 P50990

P67775 P50990

P78318 P50990

Q00005 P50990

Q00535 P50990

Q13033 P50990

Q13418 P50990

Q13573 P50990

Q15257 P50990

Q66LE6 P50990

Q8WZ74 P50990

Q99459 P50990

Q9H0R8 P50990

Q9H3D4 P50990

Q9H492 P50990

Q9H7D0 P50990

Q9NPJ1 P50990

Q9NRL3 P50990

Q9Y228 P50990

Q9Y2T4 P50990

Q9Y3A3 P50990

Q9Y6E0 P50990

O15264 P50991

O15379 P50991

O75381 P50991

P01106 P50991

P02751 P50991

P04637 P50991

P15336 P50991

P17987 P50991

P19320 P50991

P53041 P50991

P60510 P50991

P62714 P50991

P63104 P50991

P67775 P50991

P78318 P50991

Q00005 P50991

Q00535 P50991

Q13033 P50991

Q13418 P50991

Q13573 P50991

Q15257 P50991

Q15418 P50991

Q5T4F4 P50991

Q66LE6 P50991

Q8IWZ6 P50991

Q96DB2 P50991

Q99459 P50991

Q9GZX7 P50991

Q9H0R8 P50991

Q9H492 P50991

Q9H7D0 P50991

Q9NPJ1 P50991

Q9NRL3 P50991

Q9NWA0 P50991

Q9Y2T4 P50991

Q9Y6E0 P50991

O95166 P50993

O15162 P50995

O75340 P50995

Q99459 P50995

P06748 P51003

Q10570 P51003

Q15366 P51003

Q15796 P51003

P32456 P51114

P51114 P51114

Q14194 P51114

Q5UIP0 P51114

Q96F07 P51114

Q9P2H0 P51114

Q9UKR5 P51114

Q9UKV8 P51114

Q9Y383 P51114

A7MCY6 P51116

O60232 P51116

O95197 P51116

O95751 P51116

P22234 P51116

P35900 P51116

P36406 P51116

P38159 P51116

P38432 P51116

P49902 P51116

P51116 P51116

P61289 P51116

Q01804 P51116

Q13131 P51116

Q13137 P51116

Q14134 P51116

Q15041 P51116

Q15834 P51116

Q5T5P2 P51116

Q5T681 P51116

Q5VSY0 P51116

Q7Z7J5 P51116

Q86SE5 P51116

Q8N5I3 P51116

Q8N5R6 P51116

Q8WVF5 P51116

Q92731 P51116

Q93062 P51116

Q96BR9 P51116

Q96EZ8 P51116

Q96F07 P51116

Q96HA1 P51116

Q9BQD3 P51116

Q9HAF1 P51116

Q9NQC3 P51116

Q9NS73 P51116

Q9NVV9 P51116

Q9UHD2 P51116

Q9UI14 P51116

Q9UIC8 P51116

Q9UL18 P51116

Q9Y2J4 P51116

Q9Y3Q8 P51116

Q9Y4E5 P51116

A0AUL9 P51148

P02751 P51148

P19320 P51148

P20339 P51148

Q9UH99 P51148

Q9Y4K3 P51148

O14964 P51149

P02751 P51149

P15336 P51149

P30153 P51149

P30154 P51149

Q9H0R8 P51149

O00716 P51153

O60383 P51159

P68104 P51159

Q05516 P51159

Q5RL73 P51159

Q5UIP0 P51159

Q7L5N1 P51159

Q9BV36 P51159

Q9P2H0 P51159

Q9UKR5 P51159

P46934 P51168

Q96PU5 P51168

Q8N668 P51172

P21980 P51178

Q14974 P51178

Q15047 P51397

O95166 P51398

P03372 P51398

P60520 P51398

P67809 P51398

Q14197 P51398

Q5U5Q3 P51398

Q8NC60 P51398

Q9BYC9 P51398

Q9GZQ8 P51398

Q9H492 P51398

Q9Y6K9 P51398

O14503 P51449

P30154 P51449

P36873 P51449

P62136 P51449

P67775 P51449

Q14738 P51449

Q14995 P51449

Q92753 P51449

Q9H469 P51449

O60238 P51452

P27361 P51452

P28482 P51452

Q13562 P51452

P04070 P51511

Q8TAQ2 P51513

Q96SB4 P51513

O15198 P51522

Q13153 P51522

A4PIW0 P51531

O14497 P51531

P04150 P51531

P16104 P51531

P45973 P51531

Q13573 P51531

Q7Z7N5 P51531

Q8NFD5 P51531

Q9UPN9 P51531

A4PIV7 P51532

O14497 P51532

O75376 P51532

O96019 P51532

P03372 P51532

P04150 P51532

P04637 P51532

P16104 P51532

P17844 P51532

P19320 P51532

P45973 P51532

P51532 P51532

P60520 P51532

P83916 P51532

Q12824 P51532

Q13127 P51532

Q13185 P51532

Q15532 P51532

Q15831 P51532

Q68CP9 P51532

Q86U42 P51532

Q86U86 P51532

Q8NFD5 P51532

Q8TAD8 P51532

Q92922 P51532

Q96GM5 P51532

Q96L34 P51532

Q9BQA1 P51532

Q9C0C9 P51532

Q9UKL0 P51532

Q9UPN9 P51532

P01106 P51553

P50213 P51553

P21926 P51570

Q01804 P51570

Q09019 P51570

Q9HCK5 P51570

O95166 P51571

P01106 P51571

P08473 P51571

P60520 P51571

Q8IZL9 P51571

Q8NE63 P51571

Q9H0R8 P51571

Q9H492 P51571

Q9P2S5 P51571

Q9UKV8 P51571

O43240 P51572

P08697 P51572

P13569 P51572

P15336 P51572

P60468 P51572

P63104 P51572

Q07817 P51572

Q13563 P51572

Q14790 P51572

Q15629 P51572

Q6Y1H2 P51572

Q9Y3D6 P51572

Q15363 P51582

O94776 P51587

P00519 P51587

P06241 P51587

P38398 P51587

P51587 P51587

P54725 P51587

P60896 P51587

Q06609 P51587

Q14565 P51587

Q86YC2 P51587

Q8IZU3 P51587

Q9BXW9 P51587

Q9NTI5 P51587

Q9P0W2 P51587

O96006 P51606

P35713 P51608

P51531 P51608

Q9H2X6 P51608

A0JLT2 P51610

O15047 P51610

O15294 P51610

O43889 P51610

O75182 P51610

P14316 P51610

P14859 P51610

P51610 P51610

P61964 P51610

Q03164 P51610

Q06546 P51610

Q06547 P51610

Q13105 P51610

Q13547 P51610

Q70EK8 P51610

Q8IY92 P51610

Q8IZD2 P51610

Q92560 P51610

Q92731 P51610

Q92769 P51610

Q96EK4 P51610

Q96ST3 P51610

Q9H7L9 P51610

Q9NS37 P51610

Q9UBL3 P51610

Q00005 P51610

O14836 P51617

O43187 P51617

P01106 P51617

P51617 P51617

P58753 P51617

Q13286 P51617

Q13526 P51617

Q15306 P51617

Q86WV6 P51617

Q92985 P51617

Q96FA3 P51617

Q9H0E2 P51617

Q9NWZ3 P51617

Q9Y4K3 P51617

Q9Y616 P51617

Q9Y6K9 P51617

Q8N2H9 P51617

P00533 P51636

O95166 P51648

P08473 P51648

P60520 P51648

Q70EL3 P51648

Q9H0R8 P51648

Q9H492 P51648

P04150 P51649

P51649 P51649

Q9UKG1 P51654

O75381 P51659

O95166 P51659

O95758 P51659

P01106 P51659

P08567 P51659

P45983 P51659

P50613 P51659

P51659 P51659

Q01201 P51659

Q2T9J0 P51659

Q92597 P51659

Q9GZZ9 P51659

Q9H0R8 P51659

O00487 P51665

P54252 P51665

P54578 P51665

P62195 P51665

P62333 P51665

Q16401 P51665

Q9NWV8 P51665

Q9Y2K6 P51665

Q9Y5K5 P51665

O00237 P51668

O14681 P51668

O60291 P51668

O94874 P51668

O94941 P51668

O95376 P51668

P04083 P51668

P13805 P51668

P24385 P51668

P24864 P51668

P38936 P51668

P49841 P51668

P55055 P51668

P57078 P51668

P78317 P51668

P78545 P51668

Q06587 P51668

Q12899 P51668

Q13489 P51668

Q13490 P51668

Q14258 P51668

Q14790 P51668

Q15714 P51668

Q68DV7 P51668

Q6ZNA4 P51668

Q6ZSG1 P51668

Q86YT6 P51668

Q8N6T7 P51668

Q8ND25 P51668

Q8TDB6 P51668

Q969V5 P51668

Q96A37 P51668

Q96BH1 P51668

Q96EP1 P51668

Q96FW1 P51668

Q99496 P51668

Q99714 P51668

Q9BUB5 P51668

Q9BV68 P51668

Q9BZR9 P51668

Q9BZY9 P51668

Q9H0F5 P51668

Q9H5J8 P51668

Q9HCM9 P51668

Q9NVW2 P51668

Q9P0P0 P51668

Q9UBF6 P51668

Q9UKV5 P51668

Q9ULK6 P51668

Q9Y3C5 P51668

Q9Y4K3 P51668

Q9Y4L5 P51668

Q9Y577 P51668

Q9Y675 P51668

P27487 P51671

P51677 P51671

O43889 P51681

O60831 P51681

O75915 P51681

P13236 P51681

P13501 P51681

P35579 P51681

P61073 P51681

Q13007 P51681

P55084 P51687

Q9H609 P51687

Q4ZIN3 P51690

Q99608 P51690

Q9Y2Z9 P51690

O00165 P51692

P00533 P51692

P14784 P51692

P18031 P51692

P19320 P51692

Q13287 P51692

Q15303 P51692

Q5VZB9 P51692

Q6FGX7 P51692

Q6ULP2 P51692

Q92793 P51692

Q96EY1 P51692

Q9UGK3 P51692

O94779 P51693

P05067 P51693

P17028 P51693

P18754 P51693

P37840 P51693

P38936 P51693

P49407 P51693

P51693 P51693

P61224 P51693

Q06481 P51693

Q15714 P51693

Q16637 P51693

Q8IWV2 P51693

Q8WUB8 P51693

Q93074 P51693

Q9BVJ6 P51693

Q9NPJ6 P51693

O00257 P51784

O95503 P51784

P35226 P51784

P35227 P51784

P48059 P51784

P51784 P51784

Q06587 P51784

Q12788 P51784

Q15047 P51784

Q15170 P51784

Q15831 P51784

Q16539 P51784

Q8NEB9 P51784

Q93009 P51784

Q96DT6 P51784

Q96EI5 P51784

Q96G74 P51784

Q99496 P51784

Q9BXW4 P51784

Q9H0Y0 P51784

Q9HC52 P51784

Q9NR20 P51784

Q9P0W5 P51784

Q9P2N7 P51784

Q9Y463 P51784

P15382 P51787

P62158 P51787

P54253 P51788

P62136 P51788

O14745 P51790

P51808 P51808

Q96BS2 P51808

P38606 P51809

P52594 P51809

Q16655 P51810

P08238 P51812

P28482 P51812

P48740 P51812

P52788 P51812

P67870 P51812

Q15418 P51812

Q16543 P51812

Q99613 P51812

P08238 P51813

P11309 P51813

Q8N5I9 P51813

P43354 P51814

Q15796 P51814

P19174 P51816

P46108 P51816

O43541 P51817

P08238 P51817

P10644 P51817

O00472 P51825

O95402 P51825

P42568 P51825

P50750 P51825

Q03164 P51825

Q96JC9 P51825

Q9UHB7 P51825

P50750 P51826

O15162 P51828

P60520 P51841

P35398 P51843

P50148 P51884

P62995 P51884

Q92731 P51911

O60232 P51946

P10826 P51946

P25786 P51946

P50613 P51946

P51948 P51946

P56545 P51946

P67870 P51946

Q08379 P51946

Q14697 P51946

Q96JH8 P51946

Q9BZR9 P51946

P19474 P51948

P24941 P51948

P50613 P51948

P51946 P51948

P68104 P51948

Q12899 P51948

Q13064 P51948

Q5RL73 P51948

Q5UIP0 P51948

Q7L5N1 P51948

Q86YV9 P51948

Q9BYJ4 P51948

O14777 P51955

P06753 P51955

P10645 P51955

P23508 P51955

P30260 P51955

P36873 P51955

P51955 P51955

P62136 P51955

P62745 P51955

Q13042 P51955

Q14203 P51955

Q14240 P51955

Q16891 P51955

Q3B7T1 P51955

Q8NG66 P51955

Q8NHZ8 P51955

Q96K30 P51955

Q96PX6 P51955

Q9BQ70 P51955

Q9UJX5 P51955

Q9Y2X7 P51955

P16471 P51956

Q15025 P51959

Q8TBC3 P51959

Q9H4M7 P51959

Q9NS15 P51959

O43567 P51965

O95376 P51965

P09466 P51965

Q01860 P51965

Q06587 P51965

Q5JTY5 P51965

Q5VTR2 P51965

Q6ZNA4 P51965

Q86UD3 P51965

Q8N5I2 P51965

Q8ND25 P51965

Q969V5 P51965

Q96A37 P51965

Q96EP1 P51965

Q96FW1 P51965

Q96G75 P51965

Q9BY78 P51965

Q9C040 P51965

Q9H6Y7 P51965

Q9NVP2 P51965

Q9P0P0 P51965

Q9ULK6 P51965

Q9Y3C5 P51965

Q9Y4K3 P51965

Q9Y508 P51965

O43678 P51970

P01106 P51970

O14879 P51991

O95166 P51991

P03372 P51991

P11021 P51991

P11387 P51991

P19320 P51991

P49407 P51991

P51858 P51991

P60520 P51991

Q13573 P51991

Q9BXL5 P51991

Q9GZQ8 P51991

Q9H0R8 P51991

Q9H3D4 P51991

Q9H492 P51991

O15084 P52179

O75923 P52179

P0C862 P52179

P52179 P52179

Q8WZ42 P52179

P01106 P52209

Q04446 P52209

Q15051 P52209

Q5W0Q7 P52209

A0JLT2 P52272

B2Y833 P52272

O43670 P52272

O95166 P52272

O95402 P52272

O95816 P52272

P03372 P52272

P19320 P52272

P25791 P52272

P32121 P52272

P38919 P52272

P40763 P52272

P49407 P52272

P49760 P52272

P60520 P52272

P61326 P52272

P78362 P52272

Q00653 P52272

Q13573 P52272

Q8TAP4 P52272

Q99459 P52272

Q99558 P52272

Q9BQ04 P52272

Q9GZQ8 P52272

Q9H0R8 P52272

Q9H492 P52272

Q9NX70 P52272

Q9UPE1 P52272

A0JLT2 P52292

O15211 P52292

O43707 P52292

O96017 P52292

P01106 P52292

P02751 P52292

P05412 P52292

P05787 P52292

P06703 P52292

P07910 P52292

P19320 P52292

P35638 P52292

P38398 P52292

P45973 P52292

P46063 P52292

P51858 P52292

P55268 P52292

P62993 P52292

Q04206 P52292

Q13561 P52292

Q13573 P52292

Q5D1E8 P52292

Q68CJ9 P52292

Q92844 P52292

Q96EB6 P52292

Q96JB5 P52292

Q96NA2 P52292

Q96SB4 P52292

Q99459 P52292

Q9H492 P52292

Q9NSB8 P52292

Q9NWA0 P52292

Q9P2H0 P52292

Q9UH99 P52292

Q9UHY8 P52292

Q9UQ88 P52292

O43929 P52294

O75815 P52294

O94782 P52294

P00533 P52294

P15407 P52294

P15918 P52294

P29692 P52294

P38398 P52294

P40763 P52294

P49761 P52294

P63104 P52294

P78560 P52294

Q13255 P52294

Q13547 P52294

Q13573 P52294

Q8IY92 P52294

Q92688 P52294

Q92769 P52294

Q9HD26 P52294

Q9NPH3 P52294

Q04637 P52298

Q09161 P52298

Q15428 P52298

Q92900 P52298

Q96Q15 P52298

Q9Y5S9 P52298

P63000 P52306

Q9NS73 P52306

O95644 P52333

P21860 P52333

P48357 P52333

Q00403 P52333

P08559 P52429

P22234 P52429

P32121 P52429

P49407 P52429

Q01105 P52429

Q9H9J2 P52429

Q9Y266 P52429

A0JLT2 P52434

O95402 P52434

O95602 P52434

Q9BTT4 P52434

Q9NX70 P52434

A0JLT2 P52435

O15160 P52435

O95402 P52435

P08571 P52435

P46379 P52435

Q9BTT4 P52435

Q9NWA0 P52435

Q9NX70 P52435

Q9NY61 P52435

O75293 P52564

O95257 P52564

P24522 P52564

P40692 P52564

Q16539 P52564

Q5S007 P52564

Q9Y6R4 P52564

P08134 P52565

P14923 P52565

P15311 P52565

P35241 P52565

P35637 P52565

P39748 P52565

P46527 P52565

P46952 P52565

P51991 P52565

P52565 P52565

P60763 P52565

P60953 P52565

P61586 P52565

P61960 P52565

P63000 P52565

Q01105 P52565

Q01844 P52565

Q08211 P52565

Q14232 P52565

Q15011 P52565

Q6FI97 P52565

Q6IBE6 P52565

Q96PU5 P52565

Q99729 P52565

Q99963 P52565

Q9NZI8 P52565

P60763 P52566

P19320 P52594

O95166 P52597

O95229 P52597

O95758 P52597

P00533 P52597

P01106 P52597

P02751 P52597

P03372 P52597

P19320 P52597

P21980 P52597

P32121 P52597

P38919 P52597

P49336 P52597

P60520 P52597

P61326 P52597

P62993 P52597

Q00005 P52597

Q13151 P52597

Q13573 P52597

Q15051 P52597

Q15637 P52597

Q92597 P52597

Q96GM8 P52597

Q99459 P52597

Q9BWF3 P52597

Q9GZQ8 P52597

Q9H0R8 P52597

Q9H492 P52597

Q9NWB1 P52597

Q9UKV8 P52597

Q9UL18 P52597

P08238 P52630

P17181 P52630

P40763 P52630

P42224 P52630

P52630 P52630

Q00978 P52630

Q96ST2 P52630

P16220 P52655

P52657 P52655

Q9P0W5 P52655

P20226 P52655

P20226 P52657

P52657 P52657

O00716 P52701

O95166 P52701

O95758 P52701

P01106 P52701

P38398 P52701

P43246 P52701

Q13573 P52701

Q63ZY3 P52701

Q96DB2 P52701

Q99459 P52701

Q9H492 P52701

Q9NWA0 P52701

O75381 P52732

Q13573 P52732

Q8IYT8 P52732

Q96KP6 P52732

Q99459 P52732

O14513 P52735

O43432 P52735

O75575 P52735

O75674 P52735

O95155 P52735

P00533 P52735

P04626 P52735

P16471 P52735

P49368 P52735

P50502 P52735

P51956 P52735

P52597 P52735

P54725 P52735

P62993 P52735

P78314 P52735

P78362 P52735

P78371 P52735

P84022 P52735

Q13503 P52735

Q13573 P52735

Q15633 P52735

Q58F21 P52735

Q5F1R6 P52735

Q5SW79 P52735

Q7L1Q6 P52735

Q7Z3K3 P52735

Q7Z6E9 P52735

Q8IZP0 P52735

Q8NB14 P52735

Q8WUB8 P52735

Q8WWU5 P52735

Q92564 P52735

Q92598 P52735

Q96JH7 P52735

Q9NV56 P52735

Q9Y3E7 P52735

Q9Y4X5 P52735

Q9Y6E0 P52735

P01100 P52736

Q00987 P52736

Q13418 P52736

Q16659 P52736

Q99750 P52737

P18754 P52742

Q16539 P52746
[truncated: 1,402,677 more chars]
