# Supplementary material for: Identifying diabetes-related important protein targets with few interacting partners with the PageRank algorithm
Source: R Soc Open Sci. 2015 Apr 29;2(4):140252. doi: 10.1098/rsos.140252 (PMC4448867; doi:10.1098/rsos.140252)
Supplement: Table S2 gives the starting/seeding list of diabetes related proteins, originated from annotations of the UniProt database; [file rsos140252supp2.docx]

# Table S2

The list of diabetes-related human proteins from the curated Swiss-Prot subset of the UniProt database:

Entry Entry name

P04229 2B11_HUMAN

P36639 8ODP_HUMAN

O43741 AAKB2_HUMAN

Q13131 AAPK1_HUMAN

P54646 AAPK2_HUMAN

Q09428 ABCC8_HUMAN

P12821 ACE_HUMAN

Q15848 ADIPO_HUMAN

P13945 ADRB3_HUMAN

P31749 AKT1_HUMAN

P31751 AKT2_HUMAN

Q8TCU4 ALMS1_HUMAN

Q86SG2 ANR23_HUMAN

Q9BUR5 APOO_HUMAN

P41181 AQP2_HUMAN

O14520 AQP7_HUMAN

Q6UXH0 BETAT_HUMAN

P51451 BLK_HUMAN

Q96G97 BSCL2_HUMAN

Q01668 CAC1D_HUMAN

Q9HC96 CAN10_HUMAN

Q16568 CART_HUMAN

P14384 CBPM_HUMAN

P51681 CCR5_HUMAN

P28907 CD38_HUMAN

P13987 CD59_HUMAN

Q00534 CDK6_HUMAN

Q5VV42 CDKAL_HUMAN

P19835 CEL_HUMAN

Q96AQ7 CIDEC_HUMAN

Q9NZ45 CISD1_HUMAN

Q8N5K1 CISD2_HUMAN

Q2KHT3 CL16A_HUMAN

Q96KN2 CNDP1_HUMAN

P01024 CO3_HUMAN

P27658 CO8A1_HUMAN

P01189 COLI_HUMAN

P04118 COL_HUMAN

Q53ET0 CRTC2_HUMAN

P16410 CTLA4_HUMAN

Q5H9S7 DCA17_HUMAN

Q05329 DCE2_HUMAN

O95822 DCMC_HUMAN

O75398 DEAF1_HUMAN

P04440 DPB1_HUMAN

P01909 DQA1_HUMAN

P01920 DQB1_HUMAN

Q9NZJ5 E2AK3_HUMAN

O42043 ENK7_HUMAN

P22413 ENPP1_HUMAN

O43768 ENSA_HUMAN

P01588 EPO_HUMAN

P15090 FABP4_HUMAN

Q9H479 FN3K_HUMAN

Q9Y261 FOXA2_HUMAN

Q9BZS1 FOXP3_HUMAN

Q9NQR9 G6PC2_HUMAN

P22466 GALA_HUMAN

Q92908 GATA6_HUMAN

Q14397 GCKR_HUMAN

P04150 GCR_HUMAN

Q06210 GFPT1_HUMAN

Q6P9H5 GIMA6_HUMAN

P48546 GIPR_HUMAN

Q8NEA6 GLIS3_HUMAN

P43220 GLP1R_HUMAN

P47871 GLR_HUMAN

P01275 GLUC_HUMAN

A8MTJ3 GNAT3_HUMAN

P43304 GPDM_HUMAN

Q9HC97 GPR35_HUMAN

P07203 GPX1_HUMAN

P49840 GSK3A_HUMAN

P49841 GSK3B_HUMAN

O95528 GTR10_HUMAN

P11166 GTR1_HUMAN

P11168 GTR2_HUMAN

P14672 GTR4_HUMAN

P13807 GYS1_HUMAN

P68871 HBB_HUMAN

Q9UQL6 HDAC5_HUMAN

Q30201 HFE_HUMAN

P52926 HMGA2_HUMAN

P20823 HNF1A_HUMAN

P35680 HNF1B_HUMAN

P41235 HNF4A_HUMAN

Q14541 HNF4G_HUMAN

Q9UBC0 HNF6_HUMAN

P52789 HXK2_HUMAN

P35557 HXK4_HUMAN

P10997 IAPP_HUMAN

Q05084 ICA69_HUMAN

Q9Y6M1 IF2B2_HUMAN

Q9BYX4 IFIH1_HUMAN

P18510 IL1RA_HUMAN

P01589 IL2RA_HUMAN

P06213 INSR_HUMAN

P01308 INS_HUMAN

Q9Y5U9 IR3IP_HUMAN

Q14654 IRK11_HUMAN

P48051 IRK6_HUMAN

P35568 IRS1_HUMAN

P00995 ISK1_HUMAN

P61371 ISL1_HUMAN

Q9UQF2 JIP1_HUMAN

Q13555 KCC2G_HUMAN

O14901 KLF11_HUMAN

P05771 KPCB_HUMAN

P51812 KS6A3_HUMAN

Q9HA64 KT3K_HUMAN

P06239 LCK_HUMAN

P48357 LEPR_HUMAN

P41159 LEP_HUMAN

P11150 LIPC_HUMAN

P06858 LIPL_HUMAN

P02545 LMNA_HUMAN

O75581 LRP6_HUMAN

Q86YR7 MF2L2_HUMAN

Q7L1T6 NB5R4_HUMAN

Q13562 NDF1_HUMAN

Q9HD90 NDF4_HUMAN

P29120 NEC1_HUMAN

P01185 NEU2_HUMAN

Q9Y4Z2 NGN3_HUMAN

O95096 NKX22_HUMAN

P15559 NQO1_HUMAN

P03886 NU1M_HUMAN

O15294 OGT1_HUMAN

P78380 OLR1_HUMAN

O43612 OREX_HUMAN

O00443 P3C2A_HUMAN

P27986 P85A_HUMAN

P18509 PACA_HUMAN

Q96RG2 PASK_HUMAN

O43316 PAX4_HUMAN

P40424 PBX1_HUMAN

P35558 PCKGC_HUMAN

Q16654 PDK4_HUMAN

P52945 PDX1_HUMAN

Q15121 PEA15_HUMAN

P42338 PK3CB_HUMAN

Q9UM63 PLAL1_HUMAN

O15120 PLCB_HUMAN

Q96Q06 PLIN4_HUMAN

Q96AD5 PLPL2_HUMAN

P27169 PON1_HUMAN

Q15165 PON2_HUMAN

Q07869 PPARA_HUMAN

P37231 PPARG_HUMAN

Q16821 PPR3A_HUMAN

Q86XI6 PPR3B_HUMAN

Q86YN6 PRGC2_HUMAN

Q7RTS3 PTF1A_HUMAN

P41222 PTGDS_HUMAN

Q9Y2R2 PTN22_HUMAN

Q92932 PTPR2_HUMAN

Q16849 PTPRN_HUMAN

Q15256 PTPRR_HUMAN

Q6NZI2 PTRF_HUMAN

P55042 RAD_HUMAN

Q15109 RAGE_HUMAN

Q9HD89 RETN_HUMAN

P07949 RET_HUMAN

Q8HWS3 RFX6_HUMAN

P50914 RL14_HUMAN

Q9HAU8 RNPL1_HUMAN

O60779 S19A2_HUMAN

Q9BZD2 S29A3_HUMAN

O00141 SGK1_HUMAN

Q9UQQ2 SH2B3_HUMAN

O15357 SHIP2_HUMAN

P04179 SODM_HUMAN

Q9UN79 SOX13_HUMAN

Q9BX66 SRBS1_HUMAN

Q6EEV6 SUMO4_HUMAN

Q8IXH6 T53I2_HUMAN

Q03518 TAP1_HUMAN

O60343 TBCD4_HUMAN

Q9NQB0 TF7L2_HUMAN

P21980 TGM2_HUMAN

P51854 TKTL1_HUMAN

P25874 UCP1_HUMAN

P55851 UCP2_HUMAN

P55916 UCP3_HUMAN

Q96IX5 USMG5_HUMAN

Q13336 UT1_HUMAN

P30518 V2R_HUMAN

P50552 VASP_HUMAN

P15692 VEGFA_HUMAN

P17948 VGFR1_HUMAN

Q9H598 VIAAT_HUMAN

O76024 WFS1_HUMAN

Q9NU63 ZFP57_HUMAN

Q15649 ZNHI3_HUMAN

Q8IWU4 ZNT8_HUMAN
